# Synthesis of Optically Active Spirocycles by a Sequence of Decarboxylative Asymmetric Allylic Alkylation and Heck Reaction

**DOI:** 10.1021/acs.orglett.4c04079

**Published:** 2024-12-04

**Authors:** Lukas Fliegel, Marc Schmidtmann, Jens Christoffers

**Affiliations:** † Institut für Chemie, Universität Oldenburg, D-26129 Oldenburg, Germany

## Abstract

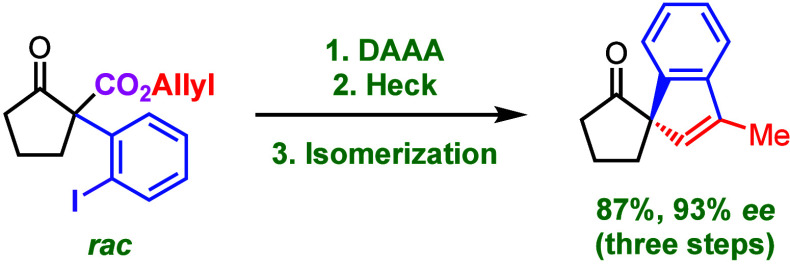

Optically active spirocycles were prepared in a sequence of two palladium-catalyzed reactions. In the first step, racemic α-(*ortho*-iodophenyl)-β-oxo allyl esters were submitted to the palladium-catalyzed decarboxylative asymmetric allylic alkylation reaction, furnishing the α-allylated products with a quaternary stereocenter with good yields and enantioselectivities. Subsequently, these intermediate products were converted in a Heck reaction yielding the spirocyclic structures as a mixture of *exo*- and *endo*-cyclic regioisomers. These mixtures could be isomerized with an acidic ion-exchange resin to give the *endo*-products with quantitative yield and selectivity. The target structure of this study could be further submitted to Friedel–Crafts reactions with electron-rich aromatic compounds.

Spirocyclic compounds possess a fascinating and synthetically challenging structural motif. Examples of naturally occurring compounds with a spirocyclic scaffold are (−)-cannabispirenones A and B (**1a**, **1b**) and fredericamycin A (**2**) (Figure 1). In both cases, the spirocarbon atom defines a quaternary stereocenter. Compounds **1a** and **1b** bear a [4,5]-spirocyclic unit and can be isolated from *Cannabis sativa*. Though they themselves are nonhallucinogenic natural products, they possess various other biological activities.^1^ A [4,4]-spirocyclic motif is found in fredericamycin A (**2**). This compound was isolated from *Streptomyces griseus* and exhibits potent antitumor activities.^2^ The synthesis of compounds like spirocycle **3** was accomplished by palladium-catalyzed cyclization of (*ortho*-bromophenyl)-functionalized ketones,^3^ even in an optically active form.^4^ Stoltz and co-workers reported the preparation of spirocyclic lactones (for example, product **4**) from (*ortho*-cyanophenyl)-functionalized lactones by applying nickel-catalysis with optically active ferrocene-based diphosphane ligands.^5^ The construction of quaternary stereocenters in α-allyl-α-arylcycloalkanones like compound **5** can be conveniently achieved by palladium-catalyzed decarboxylative asymmetric allylic alkylation (DAAA)^6^ as was recently shown by Guiry and co-workers, who used the ANDEN-Trost ligand for the preparation of product **5**.^7^

**Figure 1:**
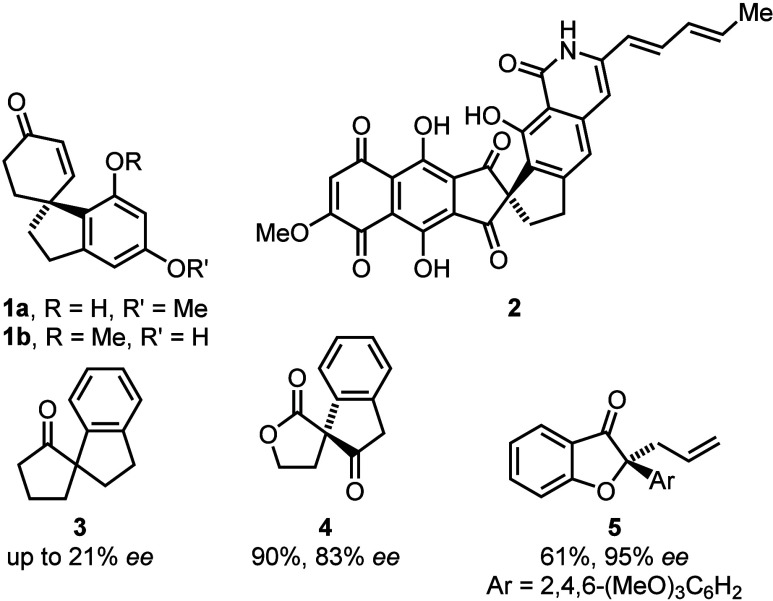
Natural products with a spirocarbocyclic scaffold: (−)-cannabispirenones A and B (**1a**, **1b**) and fredericamycin A (**2**), as well as synthetic compounds **3**–**5**.

The synthesis of β-oxoesters with an α-(*ortho*-iodophenyl)-moiety like compound **8** was first reported by Shafir and co-workers by conversion of the parent oxoesters with PhI(OCOCF_3_)_2_ [PIFA, phenyliodobis(trifluoroacetate)].^8^ We utilized compounds of type **8** for the preparation of annulated benzofuran derivatives like **6**.^9^ The latter compounds could be ring-transformed after the addition of water, furnishing lactones **7** (Scheme 1).^10^ Furthermore, copper-catalyzed aryl-amination of iodoarene **8** gave an aniline derivative that instantly cyclized to tricyclic *N*,*O*-acetal **9**. Its subsequent ring-transformation in retro-Claisen-type ring cleavage gave the medium-sized ring lactam **10**.^11^ We moreover used starting material **8** in an electrochemical transformation to benzocycloheptanone **13**.^12^ This sequence proceeded by umpolung of the iodo-carbon bond to generate carbanion **11**, which underwent ring-transformation via tricyclic intermediate **12** to furnish cyclic ketone **13**.

**Scheme 1:**
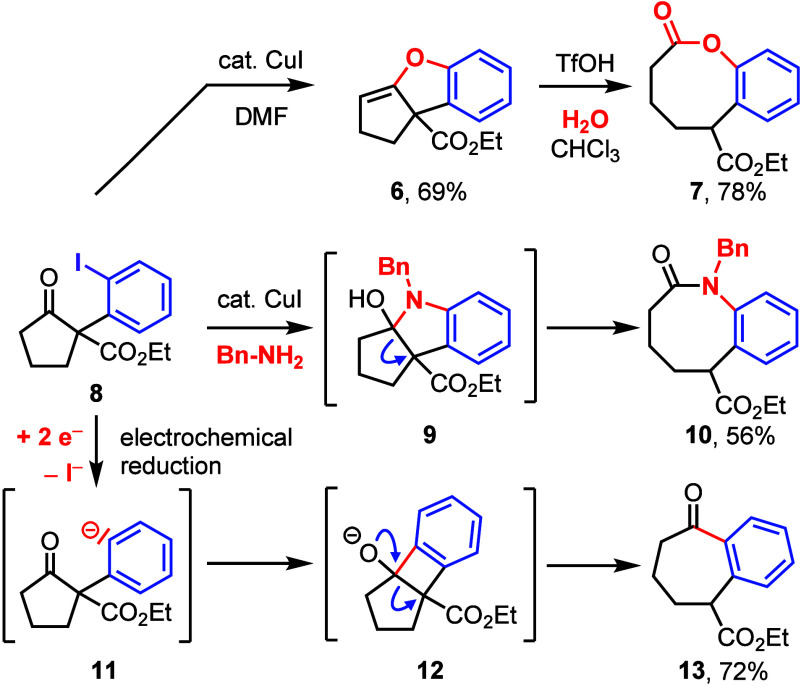
Ring-Transformations Starting from α-(*ortho*-Iodophenyl)-β-oxoester **8**: Formation of Benzofuranes, Lactones, Lactams, And Benzocycloheptanones

For the project presented herein, we have proposed to prepare α-(*ortho*-iodophenyl)-β-oxo allyl esters **14** and submit them to the DAAA (Table 1). The optically active intermediate products **15** could then be submitted to an intramolecular Heck reaction furnishing spirocyclic products. Compound **14a** (as well as the other starting materials **14b**–**14g**) were prepared from the respective β-oxo allyl esters and PIFA according to the protocols published by Shafir^8^ and us^9^ (see the Supporting Information). Compound **14a** was then converted with Pd_2_(dba)_3_ and the (*R*)-*i*Pr-PHOX-ligand **L1** under standard conditions suggested by Stoltz and co-workers (Table 1, entry 1).^13^ The product **15a** was formed with only 39% ee. We therefore investigated the DACH-Trost ligand **L2** as recommended by Guiry and co-workers^14^ and obtained 80% ee at 23 °C (entry 2). Toluene (entry 3) and 1,4-dioxane (entry 4) were also used as solvents, but the ee’s were lower. We then investigated the influence of the temperature (entries 5–9), and the selectivity increased up to 94% ee at −30 °C. The experiment was repeated on a 1 mmol scale, and product was isolated with 94% yield (entry 8). Use of the ANDEN-Trost ligand **L3** at −30 °C in THF^14^ also gave high selectivity, but the yield of the isolated product (42%) was significantly lower (entry 10). The (*S*)-configuration of compound **15a** was established by X-ray crystallography of subsequent spirocyclic product **17a** (Figure 2).

**Table 1:**
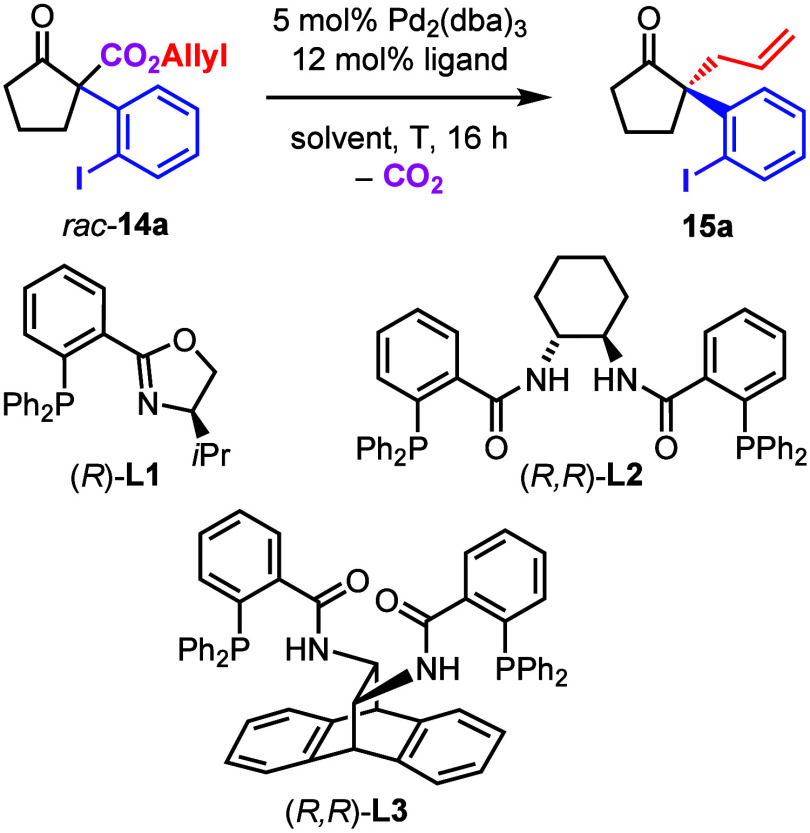
Screening Program for Suitable Reaction Conditions for the DAAA

entry	ligand	solvent	T (°C)	yield	ee
1	**L1**	THF	23	-a	39%
2	**L2**	THF	23	-a	80%
3	**L2**	toluene	23	-a	76%
4	**L2**	dioxane	23	-a	70%
5	**L2**	THF	0	-a	84%
6	**L2**	THF	–10	-a	87%
7	**L2**	THF	–20	-a	89%
8	**L2**	THF	–30	97%^b^	94%
9	**L2**	THF	–37	-a	90%
10	**L3**	THF	–30	42%^b^	93%

a The yield was not determined.

b isolated product.

**Figure 2:**
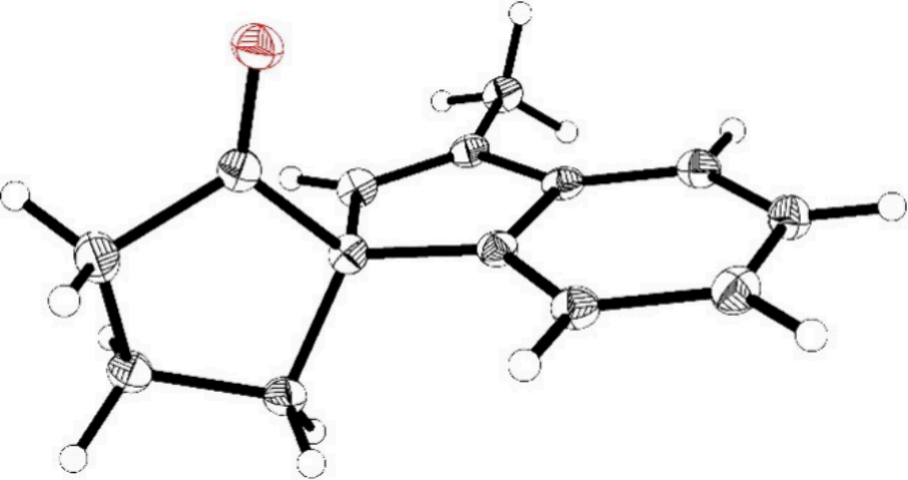
ORTEP representation of the molecular structure of compound **17a** in the solid state. Ellipsoids are located at the 50% probability level.

With the optimized reaction conditions in hand, we investigated the scope of the transformation (Table 2). First of all, the influence of the oxoester’s ring size was investigated. Compound **15a** with a five-membered ring was, as mentioned above, obtained in 97% yield and 94% ee. The product with the six-membered ring **15b** was formed only in traces at −20 °C (14% yield) with poor selectivity (24% ee; at −30 °C the results were worse). The yield of the cycloheptanone derivative **15c** was again better (53%) and the enantioselectivity was good (91% ee). Benzannulated starting materials **14d** and **14e** gave good results in the case of the five-membered ring (95% yield and 91% *ee* of product **15d**). The results for tetralone derivative **15e** were again poor (13% yield and 55% ee).

**Table 2:**
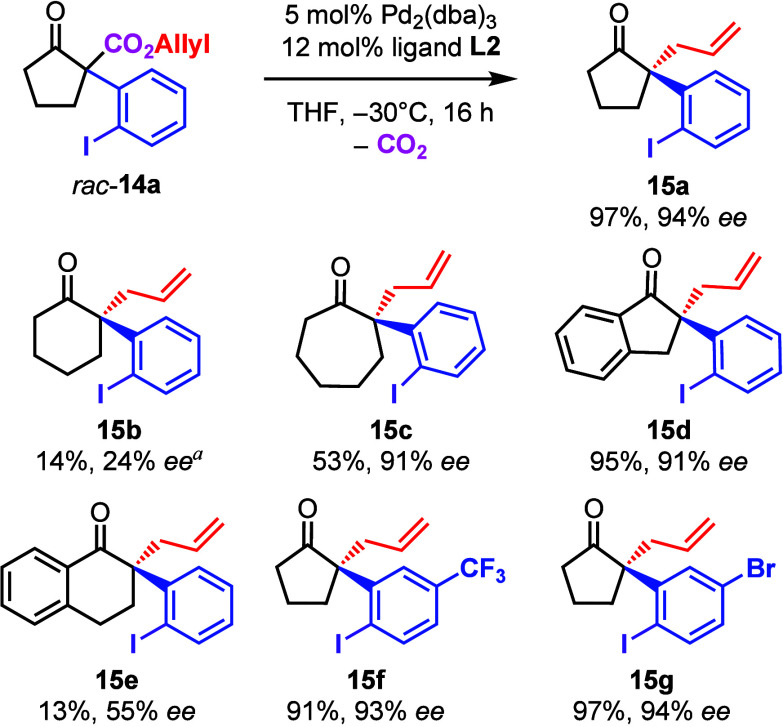
Scope of the DAAA Reaction

a At −20 °C, 48% starting material recovered.

We furthermore accessed *para*-trifluoromethyl and *para*-bromo substituted PIFA congeners and prepared the corresponding allyl esters **14f** and **14g**. As these compounds are cyclopentanone derivatives, the DAAA proceeded with good results (91% yield and 93% ee for CF_3_-substituted product **15f** and 97% yield and 94% ee for bromo-compound **15g**).

Compound **15a** was then submitted to the Heck reaction under standard conditions: cat. Pd(OAc)_2_, PPh_3_ with NEt_3_ in NMP at 65 °C (Table 3).^15^ As planned, spirocyclic compound **16a** was obtained, albeit as a mixture with its *endo*-cyclic isomer **17a** (*exo*/*endo* 5:1, total yield 90%). Attempts to obtain only *exo*-cyclic isomer **16a** by variation of the reaction conditions failed. Therefore, we aimed at the unification of the *exo*-*endo*-mixture via the application of a Brønsted acid. While homogeneous solutions of dilute H_2_SO_4_ led to unspecified decomposition, *exo*-isomer **16a** could be selectively and quantitatively isomerized to *endo*-cyclic isomer **17a** by application of the cationic ion-exchange resin Amberlyst15 at 50 °C in CH_2_Cl_2_. At this stage, we confirmed the enantiopurity of the product by GLC on a chiral phase as 93% ee. Furthermore, a single crystal of compound **17a** was suitable for an X-ray structure analysis. An ORTEP representation of the molecular structure in the solid state is depicted in Figure 2 (see the Supporting Information for details). The absolute configuration is (*S*) [Flack parameter −0.05(3)], thus, the initial (*S*)-configuration of compound **15a** was confirmed.

**Table 3:**
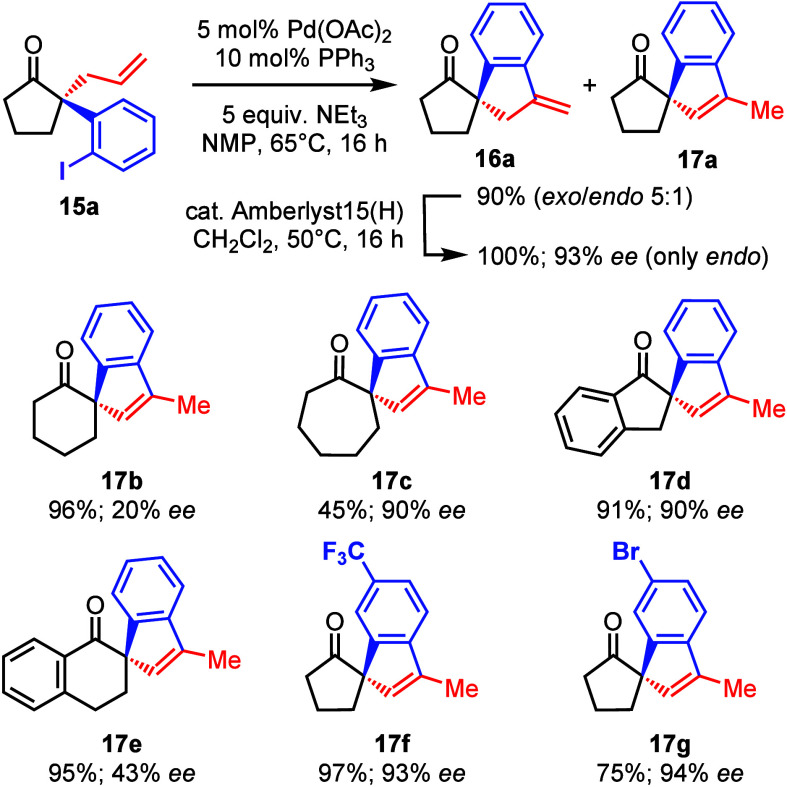
Heck Reaction and Double Bond Isomerization

With suitable conditions for the Heck reaction and isomerization to the respective *endo*-isomer in hand, we submitted all other compounds **15b**–**15g** to this sequence and also checked the enantiopurity by GLC on a chiral phase. With the exception of products **17c** and **17g**, the yields were very good (see Table 3).

Finally, we addressed the C=C double bond of racemic spirocycle **17a** for further diversification of the structure (Table 4). Thus, we performed an electrophilic aromatic substitution in neat 1,3-dimethoxybenzene after the protonation of compound **17a** with triflic acid at 0 °C. The arylated product **18a** was obtained with good yield (86%), but as a mixture of diastereoisomers (dr 5:1). Also, furan, thiophene, and benzofuran could be converted accordingly (at 0 or 23 °C), yielding the compounds **18b**–**18d** in good yields and reasonable diastereoselectivities. Benzofuran derivative **18d** was obtained as a single diastereoisomer (54% yield).

**Table 4:**
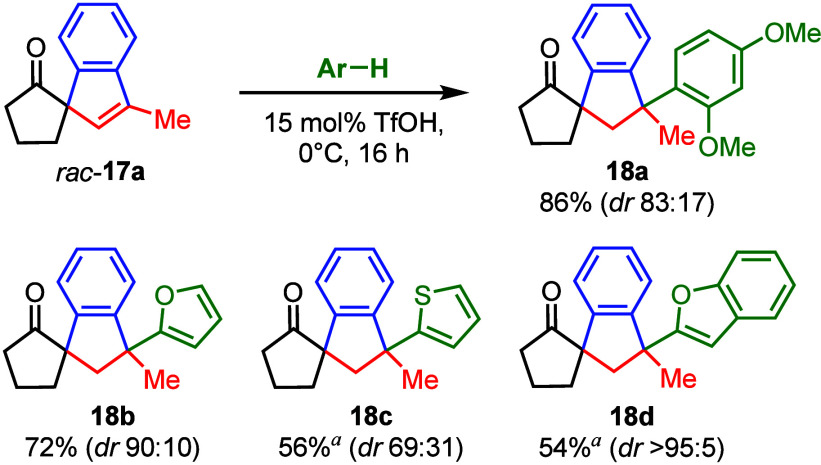
Friedel-Crafts Reaction of Racemic Spirocycle **17a** with Electron-Rich Aromatic Compounds

a Conversion at 23 °C.

In summary, we have reported a novel synthesis of optically active spirocyclic compounds **17** in three steps from α-(*ortho*-iodophenyl)-β-oxo allyl esters **14**. The latter are the products of the conversion of β-oxo allyl esters with PhI(OCOCF_3_)_2_ (PIFA). The first of two palladium-catalyzed steps was the decarboxylative asymmetric allylic alkylation with the DACH-Trost ligand **L2** yielding the α-allylated compounds **15** with a quaternary stereocenter in yields up to 97% and enantioselectivities up to 94%, which were achieved for cyclopentanone derivatives. For substrates with six-membered rings, yields and enantioselectivities were significantly lower. The second palladium-catalyzed step was a Heck reaction, leading to the spirocyclic motif. Mixtures of *exo*- and *endo*-cyclic products **16** and **17** were obtained in up to 97% yield. The mixtures of regioisomers could be submitted to acid-catalyzed isomerization with a cation exchange resin, furnishing the endocyclic isomers **17** with quantitative yield and selectivity. Finally, compound **17a** was exemplarily submitted to further diversification by Friedel–Crafts reaction with electron-rich aromatic and heteroaromatic compounds.

## Data Availability

The data underlying this study are available in the published article and its Supporting Information.

## Supporting Information Available

Synthesis, analytical data, crystallographic data, NMR spectra, and GLCs on a chiral phase (PDF)
