# Supplementary material for: Synthesis of Optically Active Spirocycles by a Sequence of Decarboxylative Asymmetric Allylic Alkylation and Heck Reaction
Source: Org Lett. 2024 Dec 4;26(49):10600–3. doi: 10.1021/acs.orglett.4c04079 (PMC11650767; doi:10.1021/acs.orglett.4c04079)
Supplement: Supplementary file 1 — ol4c04079_si_001.pdf [file ol4c04079_si_001.pdf]

Synthesis of Optically Active Spirocycles by a Sequence of Decarboxylative Asymmetric Allylic Alkylation and Heck Reaction

Lukas Fliegel, Marc Schmidtmann, and Jens Christoffers*

Institut für Chemie, Universität Oldenburg, D-26111 Oldenburg, Germany;
Email: jens.christoffers@uol.de

Supporting Information

1. General Information S2
2. Decarboxylative Asymmetric Allylic Alkylations S2
3. Heck Reactions and Isomerizations S13
4. Friedel-Crafts Reactions S22
5. α-(*ortho*-Iodophenylation) of β-Oxoesters S26
6. Synthesis of β-Oxo Allyl Esters S33
7. Crystal structure of compound **17a** S39
8. References S41
9. NMR-Spectra of all Reported Compounds S42
10. GLC on a Chiral Phase S89

1. General Information

Heating of reaction mixtures was always performed with an oil bath. Preparative column chromatography was carried out using Merck SiO_2 (35–70 µm, type 60A) with hexanes (mixture of isomers, bp. 64–71°C), *tert*-butyl methyl ether (MTBE), MeOH, ethyl acetate and acetone as eluents. TLC was performed on aluminum plates coated with SiO_2 F_{254}. ^1H, ^{13}C, and ^{19}F NMR spectra were recorded on JEOL 500, Bruker Avance III 500, Bruker Fourier 300 or Magritek Spinsolve 60 (only for ^{19}F NMR) instruments at 32°C (for 500 MHz measurements) or 23°C (for 300 MHz or 60 MHz measurements). Broad signals are abbreviated with br. Multiplicities of proton signals were abbreviated as follows: s = singlet, d = doublet, t = triplet, q = quartet, p = pentet. Combinations of these abbreviations were used accordingly, e.g. dd = double doublet, dq = doublet of quartets; ddt = doublet of doublet of triplets, etc. Multiplicities of carbon signals were determined with DEPT experiments. MS and HRMS spectra were obtained with Waters Q-TOF Premier (ESI, positive mode) or Thermo Scientific DFS (EI) spectrometers. IR spectra were recorded on a Shimadzu IR Spirit T spectrometer equipped with diamond ATR units. Optical rotations were determined with a Schmidt + Haensch Polartronic M polarimeter. GLC analyses were performed on a Shimadzu GC-2030 on a chiral Hydrodex β6-TBDM capillary column (Machery-Nagel, 25 m, 0.25 mm) with H_2 as carrier gas. The PIFA derivatives 4-(trifluoromethyl)phenyliodinebis(trifluoroacetate) and 4-bromophenyliodinebis(trifluoroacetate) were prepared according to the literature.[S1] All other starting materials were commercially available. CAUTION: NaH reacts vigorously with water.

2. Decarboxylative Asymmetric Allylic Alkylations

2.1 *rac*- or (*S*)-2-Allyl-2-(2-iodophenyl)-1-cyclopentanone (15a)

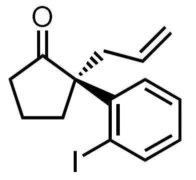

(*S*)-**15a**

Racemic: Based on a literature procedure,[S2] [$Pd(PPh_3)_4$] (0.235 g, 0.203 mmol) was added to a Schlenk tube under nitrogen atmosphere and the latter was evacuated and refilled with nitrogen three times. The α-arylated β-oxoester **14a** (0.750 g, 2.03 mmol) was then dissolved in anhydrous THF (4.5 mL) and transferred to the Schlenk tube. After rinsing with additional anhydrous THF (3 × 1 mL) the resulting mixture was stirred at ambient temperature for 30 min. The solvent was removed under reduced pressure and the residue submitted to column chromatography (SiO_2, hexanes/MTBE 20:1, R_f = 0.19) to yield the product *rac*-**15a** (0.561 g, 1.72 mmol, 85%) as a colorless oil.

Optically active: Based on a literature procedure,[S3] $[Pd_2(dba)_3]$ (49 mg, 54 µmol) and the (*R*,*R*)-DACH-phenyl Trost ligand (90 mg, 0.13 mmol) were added to a Schlenk tube under nitrogen atmosphere and the latter was evacuated and refilled with nitrogen three times. Anhydrous THF (2.8 mL) was added and the resulting mixture was stirred at ambient temperature for 1 h. The mixture was then cooled to –30°C. The α-arylated β-oxoester **14a** (0.400 g, 1.08 mmol) was dissolved in anhydrous THF (1 mL) and transferred to the cooled Schlenk tube. After rinsing with additional anhydrous THF (3 × 0.6 mL) the resulting mixture was stirred at –30°C for 16 h. The solvent was removed under reduced pressure and the residue submitted to column chromatography (SiO_2, hexanes/MTBE 20:1, R_f = 0.19) to yield the product (*S*)-**15a** (0.344 g, 1.05 mmol, 97%, 94% *ee*) as a colorless oil. ^{1}H NMR (500 MHz, $CDCl_3$): δ = 7.97 (dd, *J* = 7.9 Hz, *J* = 1.4 Hz, 1 H), 7.29–7.26 (m, 1 H), 7.23 (dd, *J* = 8.0 Hz, *J* = 1.9 Hz, 1 H), 6.90 (ddd, *J* = 7.9 Hz, *J* = 7.1 Hz, *J* = 1.9 Hz, 1 H), 5.77 (ddt, *J* = 17.2 Hz, *J* = 10.2 Hz, *J* = 7.0 Hz, 1 H), 5.18 (dq, *J* = 17.1 Hz, *J* = 1.5 Hz, 1 H), 5.10 (ddt, *J* = 10.1 Hz, *J* = 2.2 Hz, *J* = 1.1 Hz, 1 H), 2.88–2.79 (m, 3 H), 2.62 (dt, *J* = 19.1 Hz, *J* = 8.6 Hz, 1 H), 2.37 (dt, *J* = 19.1 Hz, *J* = 7.5 Hz, 1 H), 2.23 (dt, *J* = 13.4 Hz, *J* = 6.5 Hz, 1 H), 1.98–1.92 (m, 2 H) ppm. $^{13}C\{^1H\}$ NMR (125 MHz, $CDCl_3$): δ = 219.4 (C), 143.4 (C), 143.1 (CH), 133.5 (CH), 129.9 (CH), 128.5 (CH), 127.9 (CH), 118.6 (CH_2), 97.0 (C), 59.4 (C), 38.8 (CH_2), 38.6 (CH_2), 35.1 (CH_2), 18.6 (CH_2) ppm. IR (ATR): 3073 (w), 2963 (m), 2884 (w), 1733 (vs), 1637 (w), 1582 (w), 1560 (w), 1463 (s), 1430 (m), 1404 (m), 1159 (m), 1120 (m), 1006 (s), 917 (m), 753 (s), 720 (m), 699 (w), 640 (m), 556 (w), 541 (m) cm^{-1}. HRMS (EI, 70 eV) *m/z*: [M^+] calcd. for $C_{14}H_{15}IO^+$ 326.0162; found 326.0167. GLC (Hydrodex β6-TBDM; 100°C, then 5.0 K min^{-1} to 140°C, then 0.3 K min^{-1} to 172°C, 5 min hold, then 5 K min^{-1} to 200°C, 5 min hold): $t_R(S)$ = 69.35 min (major), $t_R(R)$ = 69.75 min (minor), 94% *ee*. $[\alpha]_D^{20}$ = +27.32° (CH_2Cl_2, 0.0085 g/100 mL). $C_{14}H_{15}IO$ (326.18 g mol^{-1}).

2.2 *rac*- or (*S*)-2-Allyl-2-(2-iodophenyl)-1-cyclohexanone (15b)

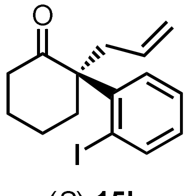

(*S*)-**15b**

Racemic: Based on a literature procedure,[S2] $[Pd(PPh_3)_4]$ (31 mg, 27 µmol) was added to a Schlenk tube under nitrogen atmosphere and the latter was evacuated and refilled with nitrogen three times. The α-arylated β-oxoester **14b** (0.104 g, 0.271 mmol) was then dissolved in anhydrous THF (0.4 mL) and transferred to the Schlenk tube. After rinsing with additional anhydrous THF (3 × 0.2 mL) the resulting mixture was stirred at 0°C for 90 min. The solvent was removed under reduced pressure and the residue submitted to column chromatography (SiO_2, hexanes/MTBE 10:1) to yield the *O*-allylated byproduct **S1** (0.66 g, 0.19 mmol, 72%, R_f

= 0.48) as a colorless oil in the first fraction. In a second fraction the product *rac*-**15b** (25 mg, 73 µmol, 27%, R_f = 0.26) could be obtained as a colorless oil.

Optically active: Based on a literature procedure,[S3] [$Pd_2(dba)_3$] (12 mg, 14 µmol) and the (*R*,*R*)-DACH-phenyl Trost ligand (22 mg, 32 µmol) were added to a Schlenk tube under nitrogen atmosphere and the latter was evacuated and refilled with nitrogen three times. Anhydrous THF (0.7 mL) was added and the resulting mixture was stirred at ambient temperature for 1 h. The mixture was then cooled to –20°C. The α-arylated β-oxoester **14b** (0.104 g, 0.271 mmol) was dissolved in anhydrous THF (0.4 mL) and transferred to the cooled Schlenk tube. After rinsing with additional anhydrous THF (3 × 0.1 mL) the resulting mixture was stirred at –20°C for 16 h. The solvent was removed under reduced pressure and the residue submitted to column chromatography (SiO_2, hexanes/MTBE 10:1) to yield the product (*S*)-**15b** (13 mg, 38 µmol, 14%, 24% *ee*, R_f = 0.29) as a colorless oil in a first fraction. In a second fraction the starting material **14b** (51 mg, 0.13 mmol, 48%, R_f = 0.14) was partially recovered. ^{1}H NMR (500 MHz, $CDCl_3$): δ = 7.94 (dd, *J* = 7.9 Hz, *J* = 1.4 Hz, 1 H), 7.38 (td, *J* = 7.7 Hz, *J* = 1.4 Hz, 1 H), 7.30 (dd, *J* = 8.0 Hz, *J* = 1.7 Hz, 1 H), 6.93 (td, *J* = 7.5 Hz, *J* = 1.7 Hz, 1 H), 5.34 (dddd, *J* = 16.7 Hz, *J* = 10.7 Hz, *J* = 8.6 Hz, *J* = 6.4 Hz, 1 H), 4.94–4.93 (m, 1 H), 4.92–4.89 (m, 1 H), 2.86 (ddt, *J* = 14.5 Hz, *J* = 6.3 Hz, *J* = 1.4 Hz, 1 H), 2.79 (dq, *J* = 14.5 Hz, *J* = 3.2 Hz, 1 H), 2.61 (dd, *J* = 14.5 Hz, *J* = 8.6 Hz, 1 H), 2.54 (td, *J* = 11.9 Hz, *J* = 5.7 Hz, 1 H), 2.40 (dtd, *J* = 11.6 Hz, *J* = 3.9 Hz, *J* = 1.1 Hz, 1 H), 2.07–2.01 (m, 1 H), 1.85–1.72 (m, 2 H), 1.66–1.63 (m, 1 H), 1.58 (ddd, *J* = 15.0 Hz, *J* = 12.4 Hz, *J* = 3.7 Hz, 1 H) ppm. $^{13}C\{^1H\}$ NMR (125 MHz, $CDCl_3$): δ = 215.0 (C), 142.7 (CH), 142.5 (C), 133.5 (CH), 130.4 (CH), 128.5 (CH), 127.7 (CH), 118.1 (CH_2), 98.5 (C), 61.3 (C), 41.6 (CH_2), 39.9 (CH_2), 39.7 (CH_2), 30.9 (CH_2), 21.5 (CH_2) ppm. IR (ATR): 3072 (w), 2937 (m), 2863 (m), 1706 (vs), 1637 (w), 1582 (w), 1560 (w), 1460 (m), 1450 (m), 1434 (m), 1330 (w), 1302 (w), 1234 (w), 1212 (w), 1114 (w), 1103 (w), 1044 (w), 1027 (w), 1007 (m), 914 (m), 833 (w), 803 (w), 754 (s), 731 (m), 713 (w), 703 (w), 659 (w), 636 (w), 589 (w), 553 (w) cm^{-1}. HRMS (ESI, pos. mode) *m/z*: [M + H^+] calcd. for $C_{15}H_{18}IO^+$ 341.0397; found 341.0396. GLC (Hydrodex β6-TBDM; 100°C, then 1.8 K min^{-1} to 130°C, then 0.06 K min^{-1} to 150°C, then 5 K min^{-1} to 200°C, 5 min hold): t_R(*R*) = 222.67 min (minor), t_R(*S*) = 225.65 min (major), 24% *ee*. $[\alpha]_D^{20}$ = –10.34° (CH_2Cl_2, 0.097 g/100 mL). $C_{15}H_{17}IO$ (340.20 g mol^{-1}).

2.2.1 2-(Allyloxy)-2'-iodo-3,4,5,6-tetrahydro-1,1'-biphenyl (S1)

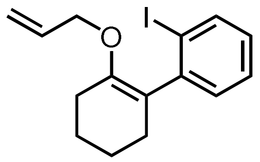

S1

The product was obtained as colorless oil and was a byproduct in the racemic procedure for compound **15b** (see above). ^{1}H NMR (300 MHz, $CDCl_3$): δ = 7.83 (dd, *J* = 8.0 Hz, *J* = 1.2 Hz, 1 H), 7.30 (td, *J* = 7.4 Hz, *J* = 1.2 Hz, 1 H), 7.14 (dd, *J* = 7.6 Hz, *J* = 1.8 Hz, 1 H), 6.90 (td, *J* = 7.6 Hz, *J* = 1.8 Hz, 1 H), 5.74 (ddt, *J* = 17.4 Hz, *J* = 10.5 Hz, *J* = 5.3 Hz, 1 H), 5.10–4.99 (m, 2 H), 4.12 (dt, *J* = 5.3 Hz, *J* = 1.6 Hz, 2 H), 2.40–2.16 (m, 3 H), 2.13–2.00 (m, 1 H), 1.89–1.66 (m, 4 H) ppm. GCMS (EI, 70 eV): m/z (%) 340 (48) [M^+], 299 (8), 259 (8), 246 (8), 231 (86), 213 (78), 203 (20), 187 (36), 171 (96), 169 (24), 156 (18), 144 (72), 129 (90), 115 (100), 102 (32), 91 (34), 77 (34), 76 (24), 63 (12), 55 (32), 41 (40). HRMS (EI, 70 eV) *m/z*: [M^+] calcd. for $C_{15}H_{17}IO^+$ 340.0319; found 340.0309. $C_{15}H_{17}IO$ (340.20 g mol^{-1}).

2.3 *rac*- or (*S*)-2-Allyl-2-(2-iodophenyl)-1-cycloheptanone (15c)

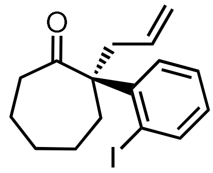

(*S*)-**15c**

Racemic: Based on a literature procedure,[S2] [$Pd(PPh_3)_4$] (29 mg, 25 µmol) was added to a Schlenk tube under nitrogen atmosphere and the latter was evacuated and refilled with nitrogen three times. The α-arylated β-oxoester **14c** (0.101 g, 0.254 mmol) was then dissolved in anhydrous THF (0.4 mL) and transferred to the Schlenk tube. After rinsing with additional anhydrous THF (3 × 0.2 mL) the resulting mixture was stirred at ambient temperature for 30 min. The solvent was removed under reduced pressure and the residue submitted to column chromatography (SiO_2, hexanes/MTBE 50:1) to yield the product *rac*-**15c** (36 mg, 0.10 mmol, 40%, R_f = 0.15) as a colorless oil in the first fraction. In a second fraction the deallylated byproduct **S2** (32 mg, 0.10 mmol, 40%, R_f = 0.06) could be obtained as a colorless solid.

Optically active: Based on a literature procedure,[S3] [$Pd_2(dba)_3$] (17 mg, 19 µmol) and the (*R*,*R*)-DACH-phenyl Trost ligand (31 mg, 45 µmol) were added to a Schlenk tube under nitrogen atmosphere and the latter was evacuated and refilled with nitrogen three times. Anhydrous THF (1 mL) was added and the resulting mixture was stirred at ambient temperature for 1 h. The mixture was then cooled to –30°C. The α-arylated β-oxoester **14c** (0.149 g, 0.374 mmol) was dissolved in anhydrous THF (0.4 mL) and transferred to the cooled Schlenk tube. After rinsing with additional anhydrous THF (3 × 0.2 mL) the resulting mixture was stirred at –30°C

for 16 h. The solvent was removed under reduced pressure and the residue submitted to column chromatography (SiO_2, hexanes/MTBE 25:1) to yield the product (*S*)-**15c** (70 mg, 20 µmol, 53%, 91% *ee*, R_f = 0.16) as a colorless oil in a first fraction. In a second fraction the deallylated byproduct **S2** (23 mg, 73 µmol, 20%, R_f = 0.07) could be obtained as a colorless solid. ^{1}H NMR (500 MHz, $CDCl_3$): δ = 7.95 (dd, *J* = 7.8 Hz, *J* = 1.4 Hz, 1 H), 7.35 (td, *J* = 7.6 Hz, *J* = 1.4 Hz, 1 H), 7.19 (dd, *J* = 8.0 Hz, *J* = 1.6 Hz, 1 H), 6.93 (td, *J* = 7.6 Hz, *J* = 1.7 Hz, 1 H), 5.27 (dddd, *J* = 16.3 Hz, *J* = 10.1 Hz, *J* = 8.9 Hz, *J* = 6.0 Hz, 1 H), 4.95–4.87 (m, 2 H), 3.09 (ddt, *J* = 14.3 Hz, *J* = 6.0 Hz, *J* = 1.5 Hz, 1 H), 2.66–2.59 (m, 2 H), 2.55 (ddd, *J* = 12.6 Hz, *J* = 6.3 Hz, *J* = 4.7 Hz, 1 H), 2.37–2.30 (m, 1 H), 1.76–1.65 (m, 3 H), 1.63–1.57 (m, 2 H), 1.48–1.40 (m, 2 H) ppm. ^{13}C{^{1}H} NMR (125 MHz, $CDCl_3$): δ = 212.9 (C), 143.5 (C), 142.8 (CH), 134.3 (CH), 130.3 (CH), 128.5 (CH), 127.7 (CH), 117.8 (CH_2), 97.7 (C), 62.1 (C), 40.4 (CH_2), 40.0 (CH_2), 36.1 (CH_2), 27.7 (CH_2), 23.4 (CH_2), 23.0 (CH_2) ppm. IR (ATR): 3072 (w), 2973 (m), 2932 (s), 2860 (m), 1703 (vs), 1637 (m), 1582 (w), 1560 (w), 1456 (s), 1430 (m), 1417 (m), 1336 (w), 1323 (m), 1263 (w), 1234 (w), 1162 (m), 1131 (m), 1053 (w), 1006 (s), 914 (s), 757 (s), 731 (s), 691 (m), 634 (w), 594 (w), 554 (w) cm^{-1}. HRMS (ESI, pos. mode) *m/z*: [M + H^+] calcd. for $C_{16}H_{20}IO^+$ 355.0553; found 355.0556. GLC (Hydrodex β6-TBDM; 100°C, then 0.04 K min^{-1} to 126°C, then 0.01 K min^{-1} to 130°C, then 5 K min^{-1} to 200°C, 5 min hold): t_R(*S*) = 960.89 min (major), t_R(*R*) = 964.76 min (minor), 91% *ee*. $[\alpha]_D^{20}$ = +69.44° (CH_2Cl_2, 0.12 g/100 mL). $C_{16}H_{19}IO$ (354.23 g mol^{-1}).

2.3.1 2-(2-Iodophenyl)cycloheptan-1-one (S2)

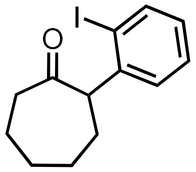

S2

The product was obtained as colorless solid and was a byproduct in the racemic and enantioselective procedure for compound **15c** (see above); mp. 67.6–69.4°C. ^{1}H NMR (500 MHz, $CDCl_3$): δ = 7.81 (dd, *J* = 8.0 Hz, *J* = 1.3 Hz, 1 H), 7.34 (td, *J* = 7.6 Hz, *J* = 1.3 Hz, 1 H), 7.20 (dd, *J* = 7.8 Hz, *J* = 1.7 Hz, 1 H), 6.93 (td, *J* = 7.6 Hz, *J* = 1.7 Hz, 1 H), 4.28 (dd, *J* = 10.9 Hz, *J* = 2.5 Hz, 1 H), 2.83 (dtd, *J* = 16.0 Hz, *J* = 4.5 Hz, *J* = 1.2 Hz, 1 H), 2.61 (ddd, *J* = 16.0 Hz, *J* = 11.6 Hz, *J* = 4.2 Hz, 1 H), 2.11–1.97 (m, 4 H), 1.92–1.84 (m, 2 H), 1.70–1.61 (m, 1 H), 1.43–1.34 (m, 1 H) ppm. ^{13}C{^{1}H} NMR (125 MHz, $CDCl_3$): δ = 212.5 (C), 144.2 (C), 139.2 (CH), 129.0 (CH), 128.4 (CH), 128.3 (CH), 101.7 (C), 61.5 (CH), 44.7 (CH_2), 32.7 (CH_2), 29.7 (CH_2), 29.1 (CH_2), 23.7 (CH_2) ppm. IR (ATR): 3059 (w), 2926 (s), 2854 (m), 1702 (vs), 1584 (w), 1563 (w), 1464 (m), 1453 (m), 1434 (m), 1330 (w), 1302 (w), 1217 (w), 1156 (m), 1132 (m), 1097 (w), 1050 (w), 1009 (s), 976 (w), 933 (m), 900 (w), 801 (w), 743 (s), 661 (w), 641 (w), 539 (w)

cm^{-1}. HRMS (ESI, pos. mode) *m/z*: [M + H$^+$] calcd. for $C_{13}H_{16}IO^+$ 315.0240; found 315.0241. $C_{13}H_{15}IO$ (314.17 g mol^{-1}).

2.4 *rac*- or (*S*)-2-Allyl-2-(2-iodophenyl)-1-indanone (15d)

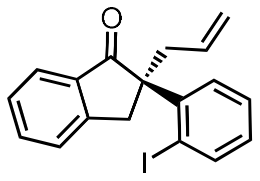

(*S*)-**15d**

Racemic: Based on a literature procedure,[S2] [Pd(PPh$_3$)$_4$] (31 mg, 27 µmol) was added to a Schlenk tube under nitrogen atmosphere and the latter was evacuated and refilled with nitrogen three times. The α-arylated β-oxoester **14d** (0.113 g, 0.270 mmol) was then dissolved in anhydrous THF (0.4 mL) and transferred to the Schlenk tube. After rinsing with additional anhydrous THF (3 × 0.2 mL) the resulting mixture was stirred at ambient temperature for 30 min. The solvent was removed under reduced pressure and the residue submitted to column chromatography (SiO$_2$, hexanes/MTBE 20:1, R$_f$ = 0.17) to yield the product *rac*-**15d** (76 mg, 0.20 mmol, 74%) as a colorless oil.

Optically active: Based on a literature procedure,[S3] [Pd$_2$(dba)$_3$] (49 mg, 53 µmol) and the (*R*,*R*)-DACH-phenyl Trost ligand (88 mg, 0.13 mmol) were added to a Schlenk tube under nitrogen atmosphere and the latter was evacuated and refilled with nitrogen three times. Anhydrous THF (2.7 mL) was added and the resulting mixture was stirred at ambient temperature for 1 h. The mixture was then cooled to –30°C. The α-arylated β-oxoester **14d** (0.442 g, 1.06 mmol) was dissolved in anhydrous THF (1.8 mL) and transferred to the cooled Schlenk tube. After rinsing with additional anhydrous THF (3 × 0.3 mL) the resulting mixture was stirred at –30°C for 16 h. The solvent was removed under reduced pressure and the residue submitted to column chromatography (SiO$_2$, hexanes/MTBE 20:1, R$_f$ = 0.17) to yield the product (*S*)-**15d** (0.378 g, 1.01 mmol, 95%, 91% *ee*) as a colorless oil. ^{1}H NMR (500 MHz, CDCl$_3$): δ = 7.96 (dd, *J* = 7.8 Hz, *J* = 1.4 Hz, 1 H), 7.84 (d, *J* = 7.6 Hz, 1 H), 7.61 (td, *J* = 7.5 Hz, *J* = 1.2 Hz, 1 H), 7.51 (dd, *J* = 8.0 Hz, *J* = 1.6 Hz, 1 H), 7.44–7.39 (m, 2 H), 7.33 (td, *J* = 7.7 Hz, *J* = 1.4 Hz, 1 H), 6.93 (td, *J* = 7.6 Hz, *J* = 1.6 Hz, 1 H), 5.56 (ddt, *J* = 17.1 Hz, *J* = 10.1 Hz, *J* = 7.1 Hz, 1 H), 5.11 (dq, *J* = 16.9 Hz, *J* = 1.6 Hz, 1 H), 4.94 (ddt, *J* = 10.2 Hz, *J* = 2.2 Hz, *J* = 1.1 Hz, 1 H), 3.66 (d, *J* = 17.3 Hz, 1 H), 3.47 (d, *J* = 17.4 Hz, 1 H), 3.01–2.96 (m, 2 H) ppm. ^{13}C{^{1}H} NMR (125 MHz, CDCl$_3$): δ = 207.0 (C), 152.1 (C), 144.1 (C), 142.5 (CH), 138.0 (C), 134.9 (CH), 133.0 (CH), 129.9 (CH), 128.6 (CH), 127.9 (CH), 127.6 (CH), 126.1 (CH), 123.9 (CH), 118.7 (CH$_2$), 97.6 (C), 58.8 (C), 41.9 (CH$_2$), 41.2 (CH$_2$) ppm. IR (ATR): 3072 (w), 2919 (w), 2846 (w), 1704 (vs), 1637 (w), 1607 (m), 1462 (s), 1429 (m), 1327 (m), 1293 (m), 1256 (m), 1184 (m), 1152 (m), 1092 (w), 1004 (m), 919 (m), 783 (m), 753 (s), 739 (s), 720 (s), 640 (m), 569 (w)

cm^{-1}. HRMS (EI, 70 eV) *m/z*: [M$^+$] calcd. for $C_{18}H_{15}IO^+$ 374.0162; found 374.0166. GLC (Hydrodex β6-TBDM; 100°C, then 5 K min^{-1} to 160°C, then 0.5 K min^{-1} to 180°C, then 0.1 K min^{-1} to 188°C, then 5 K min^{-1} to 200°C, 5 min hold): t_R(*S*) = 114.85 min (major), t_R(*R*) = 115.94 min (minor), 91% *ee*. $[\alpha]_D^{20}$ = +63.33° (CH_2Cl_2, 0.10 g/100 mL). $C_{18}H_{15}IO$ (374.22 g mol^{-1}).

2.5 *rac*- or (*S*)-2-Allyl-2-(2-iodophenyl)-1-oxo-1,2,3,4-tetrahydronaphthalene (15e)

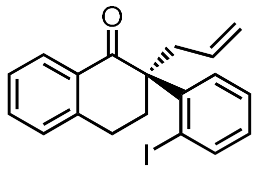

(*S*)-**15e**

Racemic: Based on a literature procedure,[S2] [Pd(PPh$_3$)$_4$] (31 mg, 27 μmol) was added to a Schlenk tube under nitrogen atmosphere and the latter was evacuated and refilled with nitrogen three times. The α-arylated β-oxoester **14e** (0.117 g, 0.271 mmol) was then dissolved in anhydrous THF (0.4 mL) and transferred to the Schlenk tube. After rinsing with additional anhydrous THF (3 × 0.2 mL) the resulting mixture was stirred at ambient temperature for 30 min. The solvent was removed under reduced pressure and the residue submitted to column chromatography (SiO_2, hexanes/ethyl acetate 50:1) to obtain the *O*-allylated byproduct **S3** (67 mg, 0.17 mmol, 64%, R_f = 0.18) in a first fraction as a colorless oil. In a second fraction the product *rac*-**15e** (9.0 mg, 23 μmol, 9%, R_f = 0.10) was received as a colorless resin. In a third fraction the deallylated byproduct **S4** (7 mg, 0.02 mmol, 7%, R_f = 0.07) was obtained as a colorless solid.

Optically active: Based on a literature procedure,[S3] [Pd$_2$(dba)$_3$] (12 mg, 14 μmol) and the (*R*,*R*)-DACH-phenyl Trost ligand (22 mg, 32 μmol) were added to a Schlenk tube under nitrogen atmosphere and the latter was evacuated and refilled with nitrogen three times. Anhydrous THF (0.7 mL) was added and the resulting mixture was stirred at ambient temperature for 1 h. The mixture was then cooled to –30°C. The α-arylated β-oxoester **14e** (0.117 g, 0.271 mmol) was dissolved in anhydrous THF (0.4 mL) and transferred to the cooled Schlenk tube. After rinsing with additional anhydrous THF (3 × 0.1 mL) the resulting mixture was stirred at –30°C for 16 h. The solvent was removed under reduced pressure and the residue submitted to column chromatography (SiO_2, hexanes/ethyl acetate 50:1) to obtain the *O*-allylated byproduct **S3** (5 mg, 0.01 mmol, 5%, R_f = 0.18) in a first fraction as a colorless oil. In a second fraction the product (*S*)-**15e** (14 mg, 36 μmol, 13%, 55% *ee*, R_f = 0.10) was received as a colorless resin. In a third fraction the deallylated byproduct **S4** (27 mg, 78 μmol, 29%, R_f = 0.07) was obtained as a colorless solid. ^{1}H NMR (500 MHz, $CDCl_3$): δ = 8.16 (dd, *J* = 7.9 Hz, *J* = 1.4 Hz, 1 H), 7.95 (dd, *J* = 7.8 Hz, *J* = 1.3 Hz, 1 H), 7.45 (td, *J* = 7.5 Hz, *J* = 1.5 Hz, 1 H), 7.35–7.32 (m, 1 H), 7.24–7.20 (m, 1 H), 7.19–7.15 (m, 2 H), 6.88 (td, *J* = 7.5 Hz, *J* = 1.8 Hz, 1 H), 5.92

(ddt, J = 17.0 Hz, J = 10.2 Hz, J = 6.9 Hz, 1 H), 5.17 (dq, J = 17.1 Hz, J = 1.7 Hz, 1 H), 5.07 (dd, J = 10.2 Hz, J = 1.9 Hz, 1 H), 3.45 (ddd, J = 13.2 Hz, J = 7.5 Hz, J = 4.6 Hz, 1 H), 3.05 (ddd, J = 17.1 Hz, J = 7.9 Hz, J = 4.8 Hz, 1 H), 3.01–2.94 (m, 2 H), 2.84 (ddd, J = 17.3 Hz, J = 7.5 Hz, J = 4.8 Hz, 1 H), 1.99 (ddd, J = 14.5 Hz, J = 7.4 Hz, J = 4.8 Hz, 1 H) ppm. $^{13}C\{^{1}H\}$ NMR (125 MHz, $CDCl_3$): δ = 199.0 (C), 143.1 (CH), 143.0 (C), 142.2 (C), 134.3 (CH), 133.6 (C), 133.0 (CH), 130.8 (CH), 128.6 (CH), 128.4 (CH), 128.2 (CH), 127.8 (CH), 126.7 (CH), 118.0 (CH_2), 96.7 (C), 56.4 (C), 38.3 (CH_2), 32.3 (CH_2), 25.8 (CH_2) ppm. IR (ATR): 3070 (w), 3022 (w), 2976 (w), 2924 (m), 2854 (w), 1679 (vs), 1637 (w), 1600 (s), 1560 (w), 1484 (w), 1464 (s), 1453 (s), 1430 (s), 1352 (w), 1304 (m), 1292 (m), 1267 (m), 1219 (s), 1156 (w), 1120 (w), 1066 (w), 1004 (s), 919 (s), 813 (w), 789 (w), 743 (vs), 720 (m), 699 (w), 669 (w), 647 (w), 637 (w), 620 (w), 544 (m) cm^{-1}. HRMS (ESI, pos. mode) *m/z*: [M + H^+] calcd. for $C_{19}H_{18}IO^+$ 389.0397; found 389.0396. GLC (Hydrodex β6-TBDM; 100°C, then 1 K min^{-1} to 150°C, 5 min hold, then 0.01 K min^{-1} to 160°C, then 5 K min^{-1} to 200°C): $t_R(S)$ = 643.06 min (major), $t_R(R)$ = 649.29 min (minor), 55% *ee*. $[\alpha]_D^{20}$ = +43.46° (CH_2Cl_2, 0.077 g/100 mL). $C_{19}H_{17}IO$ (388.25 g mol^{-1}).

2.5.1 1-(Allyloxy)-2-(2-iodophenyl)-3,4-dihydronaphthalene (S3)

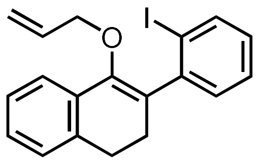

S3

The product was obtained as colorless oil and was a byproduct in the racemic and enantioselective procedure for compound **15e** (see above). ^{1}H NMR (500 MHz, $CDCl_3$): δ = 7.91 (dd, J = 7.9 Hz, J = 1.1 Hz, 1 H), 7.51 (dd, J = 7.3 Hz, J = 1.5 Hz, 1 H), 7.36–7.33 (m, 2 H), 7.28 (dd, J = 7.6 Hz, J = 1.8 Hz, 1 H), 7.23–7.18 (m, 2 H), 6.99 (td, J = 7.7 Hz, J = 1.8 Hz, 1 H), 5.74 (ddt, J = 17.2 Hz, J = 10.8 Hz, J = 5.5 Hz, 1 H), 5.12 (dq, J = 17.1 Hz, J = 1.7 Hz, 1 H), 5.04 (dq, J = 10.4 Hz, J = 1.5 Hz, 1 H), 4.17–4.10 (m, 1 H), 4.06–3.99 (m, 1 H), 3.22–3.12 (m, 1 H), 2.91–2.84 (m, 1 H), 2.74–2.66 (m, 1 H), 2.57–2.49 (m, 1 H) ppm. $^{13}C\{^{1}H\}$ NMR (125 MHz, $CDCl_3$): δ = 148.8 (C), 144.9 (C), 139.0 (CH), 137.1 (C), 134.1 (CH), 132.2 (C), 130.5 (CH), 128.4 (CH), 128.0 (CH), 127.6 (CH), 127.3 (CH), 126.9 (C), 126.4 (CH), 122.4 (CH), 116.9 (CH_2), 99.6 (C), 72.5 (CH_2), 28.9 (CH_2), 28.4 (CH_2) ppm. IR (ATR): 3057 (w), 3019 (w), 2930 (w), 2882 (w), 2829 (w), 1683 (m), 1640 (w), 1600 (w), 1556 (w), 1483 (m), 1463 (m), 1436 (m), 1426 (m), 1352 (w), 1330 (w), 1294 (s), 1270 (m), 1256 (w), 1223 (w), 1196 (m), 1157 (w), 1140 (m), 1119 (m), 1096 (m), 1063 (m), 1033 (w), 1011 (s), 980 (m), 924 (m), 896 (w), 770 (s), 750 (s), 737 (s), 720 (s), 694 (s), 643 (w), 601 (w), 540 (s), 504 (w) cm^{-1}. HRMS (ESI, pos. mode) *m/z*: [M + H^+] calcd. for $C_{19}H_{18}IO^+$ 389.0397; found 389.0394. $C_{19}H_{17}IO$ (388.25 g mol^{-1}).

2.5.2 2-(2-Iodophenyl)-1,2,3,4-tetrahydronaphthalen-1-one (S4)

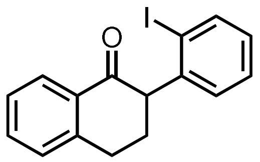

S4

The product was obtained as colorless solid and was a byproduct in the racemic and enantioselective procedure for compound **15e** (see above); mp. 107.4–109.8°C. ^{1}H NMR (500 MHz, $CDCl_3$): δ = 8.12 (dd, *J* =7.8 Hz, *J* = 1.4 Hz, 1 H), 7.90 (dd, *J* = 8.0 Hz, *J* = 1.2 Hz, 1 H), 7.53 (td, *J* = 7.5 Hz, *J* = 1.4 Hz, 1 H), 7.38–7.34 (m, 1 H), 7.34–7.30 (m, 2 H), 7.14 (dd, *J* = 7.7 Hz, *J* = 1.6 Hz, 1 H), 6.97 (td, *J* = 7.7 Hz, *J* = 1.7 Hz, 1 H), 4.24 (dd, *J* = 9.5 Hz, *J* = 7.5 Hz, 1 H), 3.28–3.20 (m, 1 H), 3.07 (dt, *J* = 16.7 Hz, *J* = 4.0 Hz, 1 H), 2.42–2.36 (m, 2 H) ppm. ^{13}C{^{1}H} NMR (125 MHz, $CDCl_3$): δ = 196.9 (C), 143.9 (C), 143.0 (C), 139.6 (CH), 133.5 (CH), 132.9 (C), 128.8 (CH), 128.70 (CH), 128.67 (CH), 128.5 (CH), 127.8 (CH), 126.8 (CH), 102.2 (C), 59.1 (CH), 31.0 (CH_2), 29.3 (CH_2) ppm. IR (ATR): 3060 (w), 3024 (w), 2926 (m), 2860 (w), 1680 (vs), 1600 (s), 1562 (w), 1467 (m), 1454 (m), 1433 (m), 1354 (m), 1313 (m), 1296 (m), 1279 (w), 1222 (s), 1192 (w), 1156 (w), 1106 (w), 1053 (w), 1027 (w), 1010 (s), 961 (w), 939 (w), 897 (m), 823 (w), 743 (vs), 724 (m), 680 (m), 646 (w), 601 (m), 554 (w), 530 (w), 487 (w) cm^{-1}. HRMS (ESI, pos. mode) *m/z*: [M + H^+] calcd. for $C_{16}H_{14}IO^+$ 349.0084; found 349.0081. $C_{16}H_{13}IO$ (348.18 g mol^{-1}).

2.6 *rac*- or (*S*)-2-Allyl-2-[2-iodo-5-(trifluoromethyl)phenyl]-1-cyclopentanone (15f)

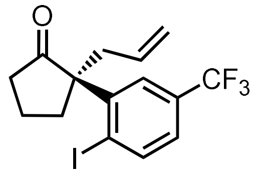

(*S*)-**15f**

Racemic: Based on a literature procedure,[S2] [$Pd(PPh_3)_4$] (55 mg, 48 µmol) was added to a Schlenk tube under nitrogen atmosphere and the latter was evacuated and refilled with nitrogen three times. The α-arylated β-oxoester **14f** (0.208 g, 0.475 mmol) was then dissolved in anhydrous THF (1.1 mL) and transferred to the Schlenk tube. After rinsing with additional anhydrous THF (3 × 0.3 mL) the resulting mixture was stirred at ambient temperature for 30 min. The solvent was removed under reduced pressure and the residue submitted to column chromatography (SiO_2, hexanes/MTBE 20:1, R_f = 0.16) to yield the product *rac*-**15f** (0.140 g, 0.355 mmol, 75%) as a colorless oil.

Optically active: Based on a literature procedure,[S3] [$Pd_2(dba)_3$] (33 mg, 37 µmol) and the (*R*,*R*)-DACH-phenyl Trost ligand (61 mg, 88 µmol) were added to a Schlenk tube under nitrogen atmosphere and the latter was evacuated and refilled with nitrogen three times. Anhydrous

THF (2 mL) was added and the resulting mixture was stirred at ambient temperature for 1 h. The mixture was then cooled to –30°C. The α-arylated β-oxoester **14f** (0.320 g, 0.730 mmol) was dissolved in anhydrous THF (1.1 mL) and transferred to the cooled Schlenk tube. After rinsing with additional anhydrous THF (3 × 0.3 mL) the resulting mixture was stirred at –30°C for 16 h. The solvent was removed under reduced pressure and the residue submitted to column chromatography (SiO_2, hexanes/MTBE 20:1, R_f = 0.16) to yield the product (*S*)-**15f** (0.261 g, 0.662 mmol, 91%, 93% *ee*) as a colorless oil. ^{1}H NMR (500 MHz, $CDCl_3$): δ = 8.07 (d, *J* = 8.2 Hz, 1 H), 7.54 (d, *J* = 2.1 Hz, 1 H), 7.14 (dd, *J* = 8.2 Hz, *J* = 2.1 Hz, 1 H), 5.74 (ddt, *J* = 17.1 Hz, *J* = 10.2 Hz, *J* = 7.0 Hz, 1 H), 5.21 (dq, *J* = 17.0 Hz, *J* = 1.6 Hz, 1 H), 5.14 (dd, *J* = 10.2 Hz, *J* = 1.6 Hz, 1 H), 2.84–2.78 (m, 2 H), 2.74 (dt, *J* = 13.7 Hz, *J* = 8.7 Hz, 1 H), 2.66 (ddd, *J* = 18.8 Hz, *J* = 9.9 Hz, *J* = 8.5 Hz, 1 H), 2.44 (ddd, *J* = 19.3 Hz, *J* = 9.4 Hz, *J* = 4.8 Hz, 1 H), 2.25 (ddd, *J* = 13.6 Hz, *J* = 7.9 Hz, *J* = 3.9 Hz, 1 H), 2.10–1.97 (m, 2 H) ppm. ^{13}C{^{1}H} NMR (125 MHz, $CDCl_3$): δ = 218.1 (C), 144.8 (C), 143.3 (CH), 132.6 (CH), 130.2 (q, *J* = 32.7 Hz, C), 126.6 (q, *J* = 3.7 Hz, CH), 124.9 (q, *J* = 3.6 Hz, CH), 123.8 (q, *J* = 273 Hz, CF_3), 119.2 (CH_2), 101.5 (C), 59.3 (C), 38.8 (CH_2), 38.4 (CH_2), 35.4 (CH_2), 18.5 (CH_2) ppm. ^{19}F{^{1}H} NMR (470 MHz, $CDCl_3$): δ = –62.96 (s) ppm. IR (ATR): 3077 (w), 2969 (w), 2924 (w), 2893 (w), 1732 (s), 1639 (w), 1603 (m), 1572 (w), 1464 (m), 1403 (m), 1327 (s), 1283 (m), 1230 (w), 1170 (s), 1120 (vs), 1089 (vs), 1009 (s), 919 (m), 890 (m), 824 (s), 773 (m), 723 (m), 706 (w), 687 (w), 667 (w), 647 (w), 627 (w), 590 (w), 544 (m) cm^{-1}. HRMS (EI, 70 eV) *m/z*: [M^+] calcd. for $C_{15}H_{14}F_3IO^+$ 394.0036; found 394.0028. GLC (Hydrodex β6-TBDM; 100°C, then 5 K min^{-1} to 140°C, then 0.3 K min^{-1} to 172°C, 5 min hold, then 5 K min^{-1} to 200°C, 5 min hold): $t_R(S)$ = 60.71 min (major), $t_R(R)$ = 62.33 min (minor), 93% *ee*. $[\alpha]_D^{20}$ = +31.94° (CH_2Cl_2, 0.12 g/100 mL). $C_{15}H_{14}F_3IO$ (394.18 g mol^{-1}).

2.7 *rac*- or (*S*)-2-Allyl-2-(5-bromo-2-iodophenyl)-1-cyclopentanone (15g)

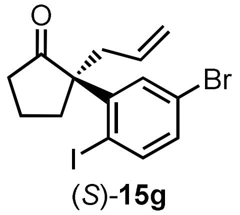

(*S*)-**15g**

Racemic: Based on a literature procedure,[S2] [$Pd(PPh_3)_4$] (49 mg, 42 µmol) was added to a Schlenk tube under nitrogen atmosphere and the latter was evacuated and refilled with nitrogen three times. The α-arylated β-oxoester **14g** (0.189 g, 0.421 mmol) was then dissolved in anhydrous THF (1 mL) and transferred to the Schlenk tube. After rinsing with additional anhydrous THF (3 × 0.2 mL) the resulting mixture was stirred at ambient temperature for 30 min. The solvent was removed under reduced pressure and the residue submitted to column chromatography (SiO_2, hexanes/MTBE 20:1, R_f = 0.22) to yield the product *rac*-**15g** (0.138 g, 0.341 mmol, 81%) as a colorless oil.

Optically active: Based on a literature procedure,[S3] [$Pd_2(dba)_3$] (35 mg, 38 μmol) and the (*R*,*R*)-DACH-phenyl Trost ligand (63 mg, 91 μmol) were added to a Schlenk tube under nitrogen atmosphere and the latter was evacuated and refilled with nitrogen three times. Anhydrous THF (2 mL) was added and the resulting mixture was stirred at ambient temperature for 1 h. The mixture was then cooled to –30°C. The α-arylated β-oxoester **14g** (0.340 g, 0.757 mmol) was dissolved in anhydrous THF (1.1 mL) and transferred to the cooled Schlenk tube. After rinsing with additional anhydrous THF (3 × 0.3 mL) the resulting mixture was stirred at –30°C for 16 h. The solvent was removed under reduced pressure and the residue submitted to column chromatography (SiO_2, hexanes/MTBE 20:1, R_f = 0.22) to yield the product (*S*)-**15g** (0.296 g, 0.731 mmol, 97%, 94% *ee*) as a colorless oil. ^1H NMR (500 MHz, $CDCl_3$): δ = 7.78 (d, *J* = 8.4 Hz, 1 H), 7.37 (d, *J* = 2.4 Hz, 1 H), 7.03 (dd, *J* = 8.3 Hz, *J* = 2.4 Hz, 1 H), 5.73 (ddt, *J* = 17.1 Hz, *J* = 10.1 Hz, *J* = 7.0 Hz, 1 H), 5.18 (dq, *J* = 17.1 Hz, *J* = 1.6 Hz, 1 H), 5.12 (dd, *J* = 10.2 Hz, *J* = 1.7 Hz, 1 H), 2.84–2.75 (m, 2 H), 2.72 (dt, *J* = 13.6 Hz, *J* = 8.3 Hz, 1 H), 2.62 (ddd, *J* = 19.3 Hz, *J* = 9.6 Hz, *J* = 8.2 Hz, 1 H), 2.38 (ddd, *J* = 19.3 Hz, *J* = 9.0 Hz, *J* = 5.5 Hz, 1 H), 2.23 (ddd, *J* = 13.2 Hz, *J* = 7.6 Hz, *J* = 4.8 Hz, 1 H), 2.02–1.92 (m, 2 H) ppm. $^{13}C\{^1H\}$ NMR (125 MHz, $CDCl_3$): δ = 218.5 (C), 145.7 (C), 144.0 (CH), 133.1 (CH), 132.8 (CH), 131.5 (CH), 122.6 (C), 119.1 (CH_2), 95.0 (C), 59.1 (C), 38.8 (CH_2), 38.5 (CH_2), 35.1 (CH_2), 18.6 (CH_2) ppm. IR (ATR): 3076 (w), 2963 (m), 2922 (m), 2889 (m), 1732 (vs), 1637 (m), 1567 (m), 1542 (m), 1452 (s), 1442 (s), 1403 (m), 1366 (m), 1272 (w), 1203 (w), 1142 (m), 1100 (s), 1063 (w), 1003 (vs), 963 (m), 916 (s), 873 (m), 807 (s), 729 (w), 711 (w), 573 (m), 553 (w), 517 (w) cm^{-1}. HRMS (ESI, pos. mode) *m/z*: [M + H^+] calcd. for $C_{14}H_{15}BrIO^+$ 404.9345; found 404.9345. GLC (Hydrodex β6-TBDM; 100°C, then 5 K min^{-1} to 140°C, then 0.3 K min^{-1} to 172°C, 5 min hold, then 5 K min^{-1} to 200°C, 5 min hold): t_R(*S*) = 125.65 min (major), t_R(*R*) = 126.05 min (minor), 94% *ee*. $[\alpha]_D^{20}$ = +14.47° (CH_2Cl_2, 0.13 g/100 mL). $C_{14}H_{14}BrIO$ (405.07 g mol^{-1}).

3. Heck Reactions and Isomerizations

3.1 *rac*- or (*S*)-3'-Methylspiro[cyclopentane-1,1'-indene]-2-one (17a)

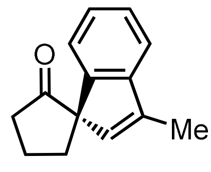

(*S*)-**17a**

Heck reaction: Based on a literature procedure,[S4] $Pd(OAc)_2$ (9 mg, 0.04 mmol) and PPh_3 (20 mg, 77 µmol) were added to a Schlenk tube under nitrogen atmosphere and the latter was evacuated and refilled with nitrogen three times. The α-allyl ketone (*S*)-**15a** (0.250 g, 0.766 mmol) was dissolved in anhydrous NMP (1.4 mL) and transferred to the Schlenk tube. After rinsing with additional anhydrous NMP (3 × 0.2 mL), NEt_3 (0.388 g, 3.83 mmol) was added and the resulting mixture was stirred at 65°C for 16 h. Subsequently, the mixture was diluted with H_2O (10 mL) and the aqueous layer extracted with MTBE (3 × 20 mL). The combined organic layers were dried over $MgSO_4$, filtered and the solvent was removed under reduced pressure. The residue was submitted to column chromatography (SiO_2, hexanes/MTBE 4:1, R_f = 0.29) to furnish *exo*- and *endo*-isomers **16a** and **17a** (0.137 g, 0.691 mmol, 90%, *exo*/*endo* 5:1) in an inseparable mixture. The ratio of *exo*- and *endo*-isomer was determined by integration and comparison of the olefin signals in the ^1H NMR spectrum. ^1H NMR (300 MHz, $CDCl_3$): δ = 7.54–7.49 (m, 1 H; *exo*), 7.32–7.20 (m, 2.8 H), 7.11–7.05 (m, 1 H; *exo*), 6.06 (q, *J* = 1.6 Hz, 0.2 H; *endo*), 5.51 (t, *J* = 2.4 Hz, 1 H; *exo*), 5.07 (t, *J* = 2.1 Hz, 1 H; *exo*), 3.03 (dt, *J* = 16.2 Hz, *J* = 2.3 Hz, 1 H; *exo*), 2.66 (dt, *J* = 16.2 Hz, *J* = 2.2 Hz, 1 H; *exo*), 2.59–2.52 (m, 0.4 H; *endo*), 2.52–2.41 (m, 2 H; *exo*), 2.41–2.36 (m, 0.2 H; *endo*), 2.34–2.12 (m, 4.2 H), 2.07–1.92 (m, 1 H; *exo*) ppm.

Isomerization: Ion-exchange resin Amberlyst15(H) (26 mg, 20% w/w) was placed in a thick-walled reaction vial. The mixture of isomers **16a** and **17a** (0.137 g, 0.691 mmol) was dissolved in CH_2Cl_2 (2.4 mL) and transferred to the vial. After rinsing with additional CH_2Cl_2 (3 × 0.2 mL) the vial was screwed tightly and the resulting mixture was stirred at 50°C for 16 h. The mixture was then filtered over SiO_2 (1.5 cm) and the solvent was removed under reduced pressure to furnish the product (*S*)-**17a** (0.137 g, 0.691 mmol, 100%, 93% *ee*) as a light orange solid and exclusively as the *endo*-isomer; mp. 57.0–59.0°C. ^1H NMR (300 MHz, $CDCl_3$): δ = 7.36–7.28 (m, 2 H), 7.26–7.19 (m, 2 H), 6.07 (q, *J* = 1.6 Hz, 1 H), 2.61–2.52 (m, 2 H), 2.46–2.37 (m, 1 H), 2.36–2.27 (m, 2 H), 2.27–2.18 (m, 1 H), 2.17 (d, *J* = 1.6 Hz, 3 H) ppm. $^{13}C\{^1H\}$ NMR (125 MHz, $CDCl_3$): δ = 218.0 (C), 147.7 (C), 145.9 (C), 141.4 (C), 132.0 (CH), 127.4 (CH), 125.7 (CH), 121.8 (CH), 119.6 (CH), 67.0 (C), 38.9 (CH_2), 33.4 (CH_2), 20.3 (CH_2), 12.9 (CH_3) ppm. IR (ATR): 3063 (w), 3016 (w), 2962 (m), 2913 (w), 2863 (w), 1733 (vs), 1613 (w), 1577 (w), 1464 (m), 1456 (m), 1447 (m), 1437 (m), 1404 (w), 1380 (w), 1343 (w), 1310 (m), 1264 (m), 1230

(w), 1159 (m), 1123 (m), 1093 (w), 1051 (w), 1020 (w), 1000 (m), 979 (w), 921 (m), 896 (w), 843 (w), 801 (m), 764 (m), 751 (s), 724 (w), 693 (w), 621 (w), 589 (w), 561 (m), 541 (w), 520 (w), 510 (w), 496 (w) cm^{-1}. HRMS (EI, 70 eV) *m/z*: [M^+] calcd. for $C_{14}H_{14}O^+$ 198.1039; found 198.1039. GLC (Hydrodex β6-TBDM; 100°C, then 5 K min^{-1} to 140°C, then 0.35 K min^{-1} to 158°C, then 5 K min^{-1} to 200°C, 5 min hold): $t_R(S)$ = 44.46 min (major), $t_R(R)$ = 45.10 min (minor), 93% *ee*. $[\alpha]_D^{20}$ = +329.19° (CH_2Cl_2, 0.14 g/100 mL). $C_{14}H_{14}O$ (198.27 g mol^{-1}).
The racemic product *rac*-**17a** was obtained by the same procedure using *rac*-**15a** as starting material (0.92 mmol-scale; *Heck reaction*: 100% yield, *exo*/*endo* 6:1; *isomerization*: 100% yield, only *endo*).

3.2 *rac*- or (*S*)-3'-Methylspiro[cyclohexane-1,1'-indene]-2-one (17b)

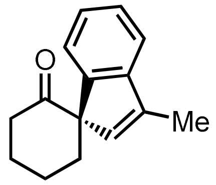

(*S*)-**17b**

Heck reaction: Based on a literature procedure,[S4] $Pd(OAc)_2$ (3 mg, 0.01 mmol) and PPh_3 (7 mg, 0.03 mmol) were added to a Schlenk tube under nitrogen atmosphere and the latter was evacuated and refilled with nitrogen three times. The α-allyl ketone (*S*)-**15b** (95 mg, 0.28 mmol) was dissolved in anhydrous NMP (0.4 mL) and transferred to the Schlenk tube. After rinsing with additional anhydrous NMP (3 × 0.2 mL), NEt_3 (0.142 g, 1.40 mmol) was added and the resulting mixture was stirred at 65°C for 16 h. Subsequently, the mixture was diluted with H_2O (10 mL) and the aqueous layer extracted with MTBE (3 × 20 mL). The combined organic layers were dried over $MgSO_4$, filtered and the solvent was removed under reduced pressure. The residue was submitted to column chromatography (SiO_2, hexanes/MTBE 10:1, R_f = 0.19) to furnish *exo*- and *endo*-isomers **16b** and **17b** (57 mg, 0.27 mmol, 96%, *exo*/*endo* 6:1) in an inseparable mixture. The ratio of *exo*- and *endo*-isomer was determined by integration and comparison of the olefin signals in the ^1H NMR spectrum. ^1H NMR (300 MHz, $CDCl_3$): δ = 7.52–7.45 (m, 1 H; *exo*), 7.41–7.38 (m, 0.16 H; *endo*), 7.35–7.32 (m, 0.16 H; *endo*), 7.31–7.24 (m, 3.32 H), 6.37–6.34 (m, 0.16 H; *endo*), 5.48 (t, *J* = 2.4 Hz, 1 H; *exo*), 5.06 (t, *J* = 2.2 Hz, 1 H; *exo*), 3.02 (dt, *J* = 16.2 Hz, *J* = 2.4 Hz, 1 H; *exo*), 2.91 (dt, *J* = 16.3 Hz, *J* = 2.2 Hz, 1 H; *exo*), 2.74–2.48 (m, 2.32 H), 2.27–1.79 (m, 7.44 H) ppm.
Isomerization: Ion-exchange resin Amberlyst15(H) (11 mg, 20% w/w) was placed in a thick-walled reaction vial. The mixture of isomers **16b** and **17b** (57 mg, 0.27 mmol) was dissolved in CH_2Cl_2 (0.4 mL) and transferred to the vial. After rinsing with additional CH_2Cl_2 (3 × 0.2 mL) the vial was screwed tightly and the resulting mixture was stirred at 50°C for 16 h. The mixture was then filtered over SiO_2 (1.5 cm) and the solvent was removed under reduced pressure to furnish the product (*S*)-**17b** (57 mg, 0.27 mmol, 100%, 20% *ee*) as a light orange solid and

exclusively as the *endo*-isomer; mp. 57.1–61.3°C. ^{1}H NMR (500 MHz, $CDCl_3$): δ = 7.40 (dd, J = 7.3 Hz, J = 1.2 Hz, 1 H), 7.32 (td, J = 7.3 Hz, J = 1.2 Hz, 1 H), 7.28–7.27 (m, 1 H), 7.27–7.24 (m, 1 H), 6.36 (q, J = 1.6 Hz, 1 H), 2.73–2.61 (m, 2 H), 2.26–2.18 (m, 2 H), 2.13 (d, J = 1.5 Hz, 3 H), 2.04–1.92 (m, 3 H), 1.84–1.79 (m, 1 H) ppm. ^{13}C{^{1}H} NMR (125 MHz, $CDCl_3$): δ = 209.0 (C), 147.2 (C), 144.5 (C), 140.6 (C), 132.2 (CH), 127.3 (CH), 125.5 (CH), 123.8 (CH), 119.8 (CH), 68.0 (C), 41.6 (CH_2), 38.0 (CH_2), 27.7 (CH_2), 23.8 (CH_2), 13.0 (CH_3) ppm. IR (ATR): 3064 (w), 3020 (w), 2933 (m), 2860 (m), 1709 (vs), 1467 (m), 1459 (m), 1449 (m), 1440 (m), 1380 (w), 1332 (m), 1309 (w), 1283 (w), 1250 (w), 1217 (m), 1177 (w), 1157 (w), 1123 (s), 1093 (w), 1069 (m), 1050 (w), 1021 (w), 994 (w), 947 (w), 933 (w), 909 (w), 876 (w), 839 (w), 809 (m), 764 (m), 751 (s), 744 (s), 727 (w), 616 (w), 574 (m), 551 (w), 524 (w), 481 (w) cm^{-1}. HRMS (ESI, pos. mode) *m/z*: [M + H^+] calcd. for $C_{15}H_{17}O^+$ 213.1274; found 213.1274. GLC (Hydrodex β6-TBDM; 100°C, then 0.05 K min^{-1} to 132°C, then 2.5 K min^{-1} to 160°C, then 5 K min^{-1} to 200°C, 10 min hold): $t_R(R)$ = 456.47 min (minor), $t_R(S)$ = 460.18 min (major), 20% *ee*. $[\alpha]_D^{20}$ = –48.72° (CH_2Cl_2, 0.13 g/100 mL). $C_{15}H_{16}O$ (212.29 g mol^{-1}).

The racemic product *rac*-**17b** was obtained by the same procedure using *rac*-**15b** as starting material (71 µmol-scale; *Heck reaction*: 86% yield, *exo*/*endo* 7:1; *isomerization*: 100% yield, only *endo*).

3.3 *rac*- or (*S*)-3'-Methylspiro[cycloheptane-1,1'-indene]-2-one (17c)

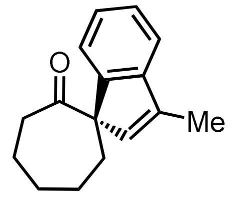

(*S*)-**17c**

Heck reaction: Based on a literature procedure,[S4] $Pd(OAc)_2$ (6 mg, 0.03 mmol) and PPh_3 (13 mg, 50 µmol) were added to a Schlenk tube under nitrogen atmosphere and the latter was evacuated and refilled with nitrogen three times. The α-allyl ketone (*S*)-**15c** (0.178 g, 0.502 mmol) was dissolved in anhydrous NMP (0.9 mL) and transferred to the Schlenk tube. After rinsing with additional anhydrous NMP (3 × 0.2 mL), NEt_3 (0.254 g, 2.51 mmol) was added and the resulting mixture was stirred at 65°C for 16 h. Subsequently, the mixture was diluted with H_2O (10 mL) and the aqueous layer extracted with MTBE (3 × 20 mL). The combined organic layers were dried over $MgSO_4$, filtered and the solvent was removed under reduced pressure. The residue was submitted to column chromatography (SiO_2, hexanes/MTBE 20:1, R_f = 0.13) to furnish *exo*- and *endo*-isomers **16c** and **17c** (total yield: 0.109 g, 0.482 mmol, 96%, *exo*/*endo* 10:1) in three fractions with varying *exo*/*endo* ratios which were later combined. The ratio of *exo*- and *endo*-isomer was determined by integration and comparison of the olefin signals in the ^{1}H NMR spectrum. In the following example ^{1}H NMR spectrum the ratio of isomers

(*exo*/*endo* 1.8:1) is different than mentioned before since several fractions with different ratios were obtained. These were later combined to give a total ratio of all fractions of *exo*/*endo* 10:1. ^{1}H NMR (300 MHz, $CDCl_3$): δ = 7.53–7.47 (m, 1 H; *exo*), 7.42–7.38 (m, 0.55 H; *endo*), 7.33–7.17 (m, 4.65 H), 6.25 (q, *J* = 1.3 Hz, 0.55 H; *endo*), 5.50 (t, *J* = 2.3 Hz, 1 H; *exo*), 5.10 (t, *J* = 1.9 Hz, 1 H; *exo*), 3.50 (dt, *J* = 16.1 Hz, *J* = 2.2 Hz, 1 H; *exo*), 3.03–2.90 (m, 1.55 H), 2.76–2.68 (m, 0.55 H; *endo*), 2.62–2.49 (m, 2 H; *exo*), 2.21–2.07 (m, 3.20 H), 2.04–1.83 (m, 6.30 H), 1.78–1.60 (m, 2.55 H), 1.44–1.28 (m, 2 H; *exo*) ppm.

Isomerization: Ion-exchange resin Amberlyst15(H) (21 mg, 20% w/w) was placed in a thick-walled reaction vial. The combined mixtures of isomers **16c** and **17c** (0.107 g, 0.473 mmol) were dissolved in CH_2Cl_2 (1.4 mL) and transferred to the vial. After rinsing with additional CH_2Cl_2 (3 × 0.2 mL) the vial was screwed tightly and the resulting mixture was stirred at 50°C for 16 h. The mixture was then filtered over SiO_2 (1.5 cm) and the solvent was removed under reduced pressure to furnish the product (*S*)-**17c** (49 mg, 0.22 mmol, 47%, 90% *ee*) as a colorless solid and exclusively as the *endo*-isomer; mp. 107.7–109.9°C. ^{1}H NMR (500 MHz, $CDCl_3$): δ = 7.42–7.40 (m, 1 H), 7.30 (td, *J* = 7.4 Hz, *J* = 1.1 Hz, 1 H), 7.25–7.23 (m, 1 H), 7.21 (td, *J* = 7.4 Hz, *J* = 1.2 Hz, 1 H), 6.26 (q, *J* = 1.7 Hz, 1 H), 2.96 (td, *J* = 11.0 Hz, *J* = 3.1 Hz, 1 H), 2.72 (ddd, *J* = 11.3 Hz, *J* = 8.2 Hz, *J* = 2.7 Hz, 1 H), 2.19–2.14 (m, 1 H), 2.15 (d, *J* = 1.6 Hz, 3 H), 2.02–1.95 (m, 2 H), 1.94–1.88 (m, 1 H), 1.85 (ddd, *J* = 14.4 Hz, *J* = 8.5 Hz, *J* = 1.2 Hz, 1 H), 1.79–1.65 (m, 3 H) ppm. ^{13}C{^{1}H} NMR (125 MHz, $CDCl_3$): δ = 211.4 (C), 147.8 (C), 144.7 (C), 140.2 (C), 134.6 (CH), 127.4 (CH), 125.5 (CH), 122.3 (CH), 119.7 (CH), 69.2 (C), 43.1 (CH_2), 35.0 (CH_2), 30.5 (CH_2), 27.1 (CH_2), 26.6 (CH_2), 13.0 (CH_3) ppm. IR (ATR): 3060 (w), 3037 (w), 3016 (w), 2970 (w), 2923 (s), 2847 (m), 1687 (s), 1622 (w), 1466 (m), 1456 (m), 1439 (m), 1382 (m), 1333 (m), 1320 (m), 1262 (w), 1250 (w), 1192 (m), 1152 (s), 1107 (w), 1076 (w), 1060 (w), 1029 (m), 1020 (m), 1009 (w), 987 (w), 950 (m), 943 (m), 919 (w), 881 (m), 849 (m), 839 (w), 799 (m), 789 (m), 766 (m), 753 (s), 744 (s), 716 (m), 596 (m), 579 (w), 544 (w), 520 (m) cm^{-1}. HRMS (ESI, pos. mode) *m/z*: [M + H^+] calcd. for $C_{16}H_{19}O^+$ 227.1430; found 227.1430. GLC (Hydrodex β6-TBDM; 100°C, then 0.5 K min^{-1} to 110°C, then 0.03 K min^{-1} to 126.7°C, then 5 K min^{-1} to 200°C, 5 min hold): $t_R(S)$ = 520.28 min (major), $t_R(R)$ = 536.06 min (minor), 90% *ee*. $[\alpha]_D^{20}$ = +165.62° (CH_2Cl_2, 0.11 g/100 mL). $C_{16}H_{18}O$ (226.32 g mol^{-1}).

The racemic product *rac*-**17c** was obtained by the same procedure using *rac*-**15c** as starting material (90 µmol-scale; *Heck reaction*: 79% yield, *exo*/*endo* 13:1; *isomerization*: 49% yield, only *endo*).

3.4 *rac*- or (*S*)-3-Methyl-1,2'-spirobi[indene]-1'(3'*H*)-one (17d)

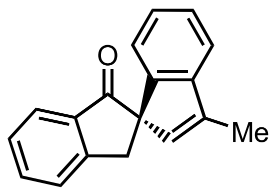

(*S*)-**17d**

Heck reaction: Based on a literature procedure,[S4] $Pd(OAc)_2$ (11 mg, 50 µmol) and PPh_3 (26 mg, 99 µmol) were added to a Schlenk tube under nitrogen atmosphere and the latter was evacuated and refilled with nitrogen three times. The α-allyl ketone (*S*)-**15d** (0.371 g, 0.991 mmol) was dissolved in anhydrous NMP (2 mL) and transferred to the Schlenk tube. After rinsing with additional anhydrous NMP (3 × 0.2 mL), NEt_3 (0.501 g, 4.96 mmol) was added and the resulting mixture was stirred at 65°C for 16 h. Subsequently, the mixture was diluted with H_2O (15 mL) and the aqueous layer extracted with MTBE (3 × 25 mL). The combined organic layers were dried over $MgSO_4$, filtered and the solvent was removed under reduced pressure. The residue was submitted to column chromatography (SiO_2, hexanes/MTBE 20:1, R_f = 0.11) to furnish *exo*- and *endo*-isomers **16d** and **17d** (0.222 g, 0.901 mmol, 91%, *exo*/*endo* 1:3) in an inseparable mixture. The ratio of *exo*- and *endo*-isomer was determined by integration and comparison of the olefin signals in the ^{1}H NMR spectrum. ^{1}H NMR (300 MHz, $CDCl_3$): δ = 7.86–7.80 (m, 1.33 H), 7.74–7.64 (m, 1.33 H), 7.62–7.51 (m, 1.66 H), 7.49–7.41 (m, 1.33 H), 7.35–7.23 (m, 2.33 H), 7.17–7.07 (m, 1.33 H), 6.92 (dt, *J* = 7.5 Hz, *J* = 1.0 Hz, 1 H; *endo*), 6.79 (dt, *J* = 7.6 Hz, *J* = 0.9 Hz, 0.33 H; *exo*), 6.03 (q, *J* = 1.6 Hz, 1 H; *endo*), 5.59 (t, *J* = 2.4 Hz, 0.33 H; *exo*), 5.17 (t, *J* = 2.1 Hz, 0.33 H; *exo*), 3.61 (d, *J* = 17.3 Hz, 1 H; *endo*), 3.55–3.44 (m, 1.33 H), 3.44–3.35 (m, 0.66 H; *exo*), 2.86 (dt, *J* = 16.4 Hz, *J* = 2.3 Hz, 0.33 H; *exo*), 2.24 (d, *J* = 1.6 Hz, 3 H; *endo*) ppm.

Isomerization: Ion-exchange resin Amberlyst15(H) (44 mg, 20% w/w) was placed in a thick-walled reaction vial. The mixture of isomers **16d** and **17d** (0.222 g, 0.901 mmol) was dissolved in CH_2Cl_2 (2.9 mL) and transferred to the vial. After rinsing with additional CH_2Cl_2 (3 × 0.2 mL) the vial was screwed tightly and the resulting mixture was stirred at 50°C for 16 h. The mixture was then filtered over SiO_2 (2 cm) and the solvent was removed under reduced pressure to furnish the product (*S*)-**17d** (0.222 g, 0.901 mmol, 100%, 90% *ee*) as a colorless solid and exclusively as the *endo*-isomer; mp. 117.5–120.7°C. ^{1}H NMR (500 MHz, $CDCl_3$): δ = 7.85 (d, *J* = 7.7 Hz, 1 H), 7.70 (td, *J* = 7.5 Hz, *J* = 1.2 Hz, 1 H), 7.60 (dt, *J* = 7.7 Hz, *J* = 1.0 Hz, 1 H), 7.48–7.44 (m, 1 H), 7.36–7.31 (m, 2 H), 7.13 (ddd, *J* = 8.6 Hz, *J* = 6.5 Hz, *J* = 2.1 Hz, 1 H), 6.94 (dd, *J* = 7.4 Hz, *J* = 0.9 Hz, 1 H), 6.05 (q, *J* = 1.6 Hz, 1 H), 3.61 (d, *J* = 17.3 Hz, 1 H), 3.51 (d, *J* = 17.2 Hz, 1 H), 2.26 (d, *J* = 1.6 Hz, 3 H) ppm. $^{13}C\{^1H\}$ NMR (125 MHz, $CDCl_3$): δ = 204.7 (C), 152.8 (C), 147.8 (C), 145.6 (C), 142.5 (C), 137.0 (C), 135.1 (CH), 132.8 (CH), 127.5 (CH), 127.3 (CH), 126.7 (CH), 125.7 (CH), 124.7 (CH), 120.6 (CH), 119.5 (CH), 66.4 (C), 35.8 (CH_2), 13.0 (CH_3) ppm. IR (ATR): 3064 (w), 3016 (w), 2912 (w), 2853 (w), 1710 (s), 1606 (m), 1589

(m), 1463 (m), 1433 (m), 1380 (w), 1342 (w), 1322 (m), 1270 (m), 1244 (m), 1207 (m), 1184 (m), 1153 (m), 1110 (m), 1037 (w), 1017 (m), 1009 (m), 954 (w), 910 (m), 880 (m), 866 (m), 816 (m), 799 (m), 754 (s), 746 (s), 734 (s), 684 (s), 649 (s), 574 (w), 524 (w), 504 (w) cm^{-1}. HRMS (ESI, pos. mode) *m/z*: [M + H^+] calcd. for $C_{18}H_{15}O^+$ 247.1117; found 247.1118. GLC (Hydrodex β6-TBDM; 100°C, then 2 K min^{-1} to 144°C, then 0.01 K min^{-1} to 148.5°C, then 5 K min^{-1} to 200°C, 5 min hold): $t_R(R)$ = 368.26 min (minor), $t_R(S)$ = 373.83 min (major), 90% *ee*. $[\alpha]_D^{20}$ = +102.67° (CH_2Cl_2, 0.50 g/100 mL). $C_{18}H_{14}O$ (246.31 g mol^{-1}).

The racemic product *rac*-**17d** was obtained by the same procedure using *rac*-**15d** as starting material (0.1 mmol-scale; *Heck reaction*: 95% yield, *exo*/*endo* 1:1; *isomerization*: 100% yield, only *endo*).

3.5 *rac*- or (*S*)-3-Methyl-3',4'-dihydro-1'*H*-spiro[indene-1,2'-naphthalene]-1'-one (17e)

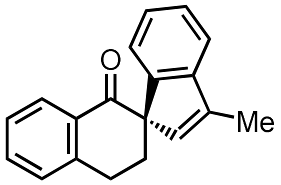

(*S*)-**17e**

Heck reaction: Based on a literature procedure,[S4] $Pd(OAc)_2$ (1 mg, 4 µmol) and PPh_3 (2 mg, 9 µmol) were added to a Schlenk tube under nitrogen atmosphere and the latter was evacuated and refilled with nitrogen three times. The α-allyl ketone (*S*)-**15e** (33 mg, 85 µmol) was dissolved in anhydrous NMP (0.2 mL) and transferred to the Schlenk tube. After rinsing with additional anhydrous NMP (3 × 0.1 mL), NEt_3 (43 mg, 0.43 mmol) was added and the resulting mixture was stirred at 65°C for 16 h. Subsequently, the mixture was diluted with H_2O (5 mL) and the aqueous layer extracted with MTBE (3 × 10 mL). The combined organic layers were dried over $MgSO_4$, filtered and the solvent was removed under reduced pressure. The residue was submitted to column chromatography (SiO_2, hexanes/MTBE 20:1, R_f = 0.16) to furnish *exo*- and *endo*-isomers **16e** and **17e** (22 mg, 85 µmol, 100%, *exo*/*endo* 10:1) in an inseparable mixture. The ratio of *exo*- and *endo*-isomer was determined by integration and comparison of the olefin signals in the ^{1}H NMR spectrum. ^{1}H NMR (300 MHz, $CDCl_3$): δ = 8.11 (dd, *J* = 7.9 Hz, *J* = 1.4 Hz, 1 H; *exo*), 8.05–8.02 (m, 0.1 H; *endo*), 7.59–7.50 (m, 2.1 H), 7.40–7.27 (m, 3.4 H), 7.23–7.15 (m, 1.2 H), 7.01 (d, *J* = 7.6 Hz, 1 H; *exo*), 6.29 (q, *J* = 1.6 Hz, 0.1 H; *endo*), 5.55 (t, *J* = 2.4 Hz, 1 H; *exo*), 5.12 (t, *J* = 2.1 Hz, 1 H; *exo*), 3.44 (dt, *J* = 16.1 Hz, *J* = 2.5 Hz, 1 H; *exo*), 3.28–3.24 (m, 0.2 H; *endo*), 3.16–3.04 (m, 2 H; *exo*), 2.78 (dt, *J* = 16.2 Hz, *J* = 2.0 Hz, 2 H; *exo*), 2.54–2.45 (m, 0.1 H; *endo*), 2.44–2.34 (m, 1 H; *exo*), 2.29–2.19 (m, 1.1 H), 2.16 (d, *J* = 1.6 Hz, 0.3 H; *endo*) ppm.

Isomerization: Ion-exchange resin Amberlyst15(H) (4 mg, 20% w/w) was placed in a thick-walled reaction vial. The mixture of isomers **16e** and **17e** (22 mg, 85 µmol) was dissolved in CH_2Cl_2 (0.2 mL) and transferred to the vial. After rinsing with additional CH_2Cl_2 (3 × 0.1 mL) the vial was screwed tightly and the resulting mixture was stirred at 50°C for 16 h. The mixture was then filtered over SiO_2 (1 cm) and the solvent was removed under reduced pressure to furnish the product (*S*)-**17e** (21 mg, 81 µmol, 95%, 43% *ee*) as a light yellow solid and exclusively as the *endo*-isomer; mp. 78.2–85.8°C. ^{1}H NMR (500 MHz, $CDCl_3$): δ = 8.05–8.02 (m, 1 H), 7.55 (td, *J* = 7.5 Hz, *J* = 1.4 Hz, 1 H), 7.36–7.31 (m, 4 H), 7.22–7.18 (m, 2 H), 6.29 (q, *J* = 1.4 Hz, 1 H), 3.28–3.25 (m, 2 H), 2.50 (ddd, *J* = 13.9 Hz, *J* = 7.7 Hz, *J* = 6.5 Hz, 1 H), 2.22 (dt, *J* = 13.4 Hz, *J* = 5.5 Hz, 1 H), 2.17 (d, *J* = 1.5 Hz, 3 H) ppm. $^{13}C\{^1H\}$ NMR (125 MHz, $CDCl_3$): δ = 197.1 (C), 147.4 (C), 145.5 (C), 143.6 (C), 141.4 (C), 133.4 (CH), 133.0 (C), 132.3 (CH), 129.0 (CH), 128.1 (CH), 127.5 (CH), 126.7 (CH), 125.6 (CH), 122.9 (CH), 119.9 (CH), 63.9 (C), 32.4 (CH_2), 28.0 (CH_2), 13.1 (CH_3) ppm. IR (ATR): 3063 (w), 3020 (w), 2924 (m), 2853 (m), 1680 (s), 1599 (m), 1483 (w), 1467 (m), 1453 (m), 1434 (m), 1380 (w), 1349 (m), 1333 (m), 1299 (m), 1224 (s), 1157 (w), 1110 (w), 1080 (w), 1023 (m), 964 (w), 944 (m), 896 (m), 871 (w), 847 (w), 810 (m), 794 (m), 777 (m), 753 (s), 740 (s), 717 (m), 687 (w), 650 (m), 560 (w), 550 (w), 531 (w), 501 (w), 484 (m) cm^{-1}. HRMS (ESI, pos. mode) *m/z*: [M + H^+] calcd. for $C_{19}H_{17}O^+$ 261.1274; found 261.1273. GLC (Hydrodex β6-TBDM; 100°C, then 0.5 K min^{-1} to 150°C, 5 min hold, then 0.01 K min^{-1} to 154.2°C, then 5 K min^{-1} to 200°C, 5 min hold): $t_R(S)$ = 485.53 min (major), $t_R(R)$ = 491.11 min (minor), 43% *ee*. $[\alpha]_D^{20}$ = +80.11° (CH_2Cl_2, 0.15 g/100 mL). $C_{19}H_{16}O$ (260.34 g mol^{-1}).

The racemic product *rac*-**17e** was obtained by the same procedure using *rac*-**15e** as starting material (98 µmol-scale; *Heck reaction*: 79% yield, *exo*/*endo* 1:1; *isomerization*: 100% yield, only *endo*).

3.6 *rac*- or (*S*)-3'-Methyl-6'-(trifluoromethyl)spiro[cyclopentane-1,1'-indene]-2-one (17f)

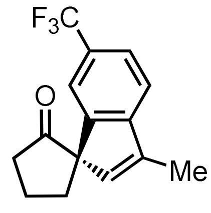

(*S*)-**17f**

Heck reaction: Based on a literature procedure,[S4] $Pd(OAc)_2$ (7 mg, 0.03 mmol) and PPh_3 (17 mg, 65 µmol) were added to a Schlenk tube under nitrogen atmosphere and the latter was evacuated and refilled with nitrogen three times. The α-allyl ketone (*S*)-**15f** (0.256 g, 0.649 mmol) was dissolved in anhydrous NMP (1.4 mL) and transferred to the Schlenk tube. After rinsing with additional anhydrous NMP (3 × 0.2 mL), NEt_3 (0.328 g, 3.25 mmol) was added and

the resulting mixture was stirred at 65°C for 16 h. Subsequently, the mixture was diluted with H_2O (10 mL) and the aqueous layer extracted with MTBE (3 × 20 mL). The combined organic layers were dried over $MgSO_4$, filtered and the solvent was removed under reduced pressure. The residue was submitted to column chromatography (SiO_2, hexanes/MTBE 5:1, R_f = 0.21) to furnish *exo*- and *endo*-isomers **16f** and **17f** (0.167 g, 0.627 mmol, 97%, *exo*/*endo* 5:1) in an inseparable mixture. The ratio of *exo*- and *endo*-isomer was determined by integration and comparison of the olefin signals in the ^{1}H NMR spectrum. ^{1}H NMR (300 MHz, $CDCl_3$): δ = 7.60–7.57 (m, 1.2 H), 7.51 (dd, *J* = 8.2 Hz, *J* = 1.5 Hz, 1 H; *exo*), 7.45 (d, *J* = 1.5 Hz, 0.2 H; *endo*), 7.36 (d, *J* = 7.9 Hz, 0.2 H; *endo*), 7.32 (d, *J* = 1.6 Hz, 1 H; *exo*), 6.21 (q, *J* = 1.6 Hz, 0.2 H; *endo*), 5.61 (t, *J* = 2.5 Hz, 1 H; *exo*), 5.19 (t, *J* = 2.1 Hz, 1 H; *exo*), 3.05 (dt, *J* = 16.3 Hz, *J* = 2.3 Hz, 1 H; *exo*), 2.71 (dt, *J* = 16.2 Hz, *J* = 2.3 Hz, 1 H; *exo*), 2.61–2.56 (m, 0.4 H; *endo*), 2.53 (ddd, *J* = 8.8 Hz, *J* = 4.2 Hz, *J* = 1.5 Hz, 0.2 H; *endo*), 2.52–2.40 (m, 2 H; *exo*), 2.35–2.25 (m, 1.4 H), 2.23–2.15 (m, 2.8 H), 2.07–1.96 (m, 1 H; *exo*) ppm.

Isomerization: Ion-exchange resin Amberlyst15(H) (33 mg, 20% w/w) was placed in a thick-walled reaction vial. The mixture of isomers **16f** and **17f** (0.167 g, 0.627 mmol) was dissolved in CH_2Cl_2 (1.9 mL) and transferred to the vial. After rinsing with additional CH_2Cl_2 (3 × 0.2 mL) the vial was screwed tightly and the resulting mixture was stirred at 50°C for 3 d. The mixture was then filtered over SiO_2 (1.5 cm) and the solvent was removed under reduced pressure to furnish the product (*S*)-**17f** (0.167 g, 0.627 mmol, 100%, 93% *ee*) as a colorless solid and exclusively as the *endo*-isomer; mp. 91.0–94.2°C. ^{1}H NMR (500 MHz, $CDCl_3$): δ = 7.59 (dd, *J* = 8.0 Hz, *J* = 1.5 Hz, 1 H), 7.45 (d, *J* = 1.5 Hz, 1 H), 7.36 (d, *J* = 7.9 Hz, 1 H), 6.22 (q, *J* = 1.5 Hz, 1 H), 2.64–2.56 (m, 2 H), 2.51–2.45 (m, 1 H), 2.38–2.31 (m, 2 H), 2.27–2.20 (m, 1 H), 2.18 (d, *J* = 1.5 Hz, 3 H) ppm. $^{13}C\{^1H\}$ NMR (125 MHz, $CDCl_3$): δ = 216.8 (C), 149.5 (C), 148.0 (C), 140.6 (C), 134.6 (CH), 127.7 (q, *J* = 32.0 Hz, C), 124.9 (q, *J* = 3.8 Hz, CH), 124.6 (q, *J* = 272 Hz, CF_3), 119.6 (CH), 118.6 (q, *J* = 3.8 Hz, CH), 67.2 (C), 39.0 (CH_2), 33.0 (CH_2), 20.3 (CH_2), 12.8 (CH_3) ppm. $^{19}F\{^1H\}$ NMR (58 MHz, $CDCl_3$): δ = –59.33 (s) ppm. IR (ATR): 2966 (w), 2917 (w), 2867 (w), 1740 (s), 1617 (w), 1579 (w), 1483 (w), 1470 (w), 1449 (w), 1430 (m), 1407 (w), 1384 (w), 1357 (w), 1324 (vs), 1269 (m), 1257 (s), 1193 (w), 1162 (s), 1110 (vs), 1063 (s), 1000 (m), 936 (w), 916 (w), 890 (m), 834 (s), 813 (m), 800 (m), 766 (w), 746 (w), 721 (s), 661 (w), 636 (w), 621 (w), 597 (w), 576 (m), 569 (m), 544 (w), 493 (w), 487 (w) cm^{-1}. HRMS (ESI, pos. mode) *m/z*: [M + Na^+] calcd. for $C_{15}H_{13}F_3NaO^+$ 289.0811; found 289.0811. GLC (Hydrodex β6-TBDM; 100°C, then 5 K min^{-1} to 140°C, then 0.35 K min^{-1} to 158°C, then 5 K min^{-1} to 200°C, 5 min hold): t_R(*R*) = 39.69 min (minor), t_R(*S*) = 42.87 min (major), 93% *ee*. $[\alpha]_D^{20}$ = +268.49° (CH_2Cl_2, 0.12 g/100 mL). $C_{15}H_{13}F_3O$ (266.26 g mol^{-1}).

The racemic product *rac*-**17f** was obtained by the same procedure using *rac*-**15f** as starting material (0.355 mmol-scale; *Heck reaction*: 91% yield, *exo*/*endo* 4.4:1; *isomerization*: 92% yield, only *endo*).

3.7 *rac*- or (*S*)-6'-Bromo-3'-methylspiro[cyclopentane-1,1'-indene]-2-one (17g)

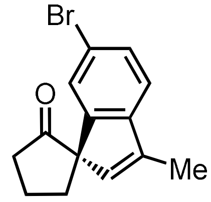

(*S*)-**17g**

Heck reaction: Based on a literature procedure,[S4] $Pd(OAc)_2$ (8 mg, 0.04 mmol) and PPh_3 (18 mg, 70 µmol) were added to a Schlenk tube under nitrogen atmosphere and the latter was evacuated and refilled with nitrogen three times. The α-allyl ketone (*S*)-**15g** (0.285 g, 0.704 mmol) was dissolved in anhydrous NMP (1.4 mL) and transferred to the Schlenk tube. After rinsing with additional anhydrous NMP (3 × 0.2 mL), NEt_3 (0.356 g, 3.52 mmol) was added and the resulting mixture was stirred at 65°C for 16 h. Subsequently, the mixture was diluted with H_2O (10 mL) and the aqueous layer extracted with MTBE (3 × 20 mL). The combined organic layers were dried over $MgSO_4$, filtered and the solvent was removed under reduced pressure. The residue was submitted to column chromatography (SiO_2, hexanes/MTBE 5:1, R_f = 0.22) to furnish *exo*- and *endo*-isomers **16g** and **17g** (0.151 g, 0.545 mmol, 77%, *exo*/*endo* 4:1) in an inseparable mixture. The ratio of *exo*- and *endo*-isomer was determined by integration and comparison of the olefin signals in the ^1H NMR spectrum. ^1H NMR (300 MHz, $CDCl_3$): δ = 7.43 (dd, *J* = 8.0 Hz, *J* = 1.8 Hz, 0.25 H; *endo*), 7.40–7.32 (m, 2.25 H), 7.22–7.18 (m, 1 H; *exo*), 7.13 (d, *J* = 8.0 Hz, 0.25 H; *endo*), 6.04 (q, *J* = 1.8 Hz, 0.25 H; *endo*), 5.49 (t, *J* = 2.5 Hz, 1 H; *exo*), 5.08 (t, *J* = 2.2 Hz, 1 H; *exo*), 3.01 (dt, *J* = 16.3 Hz, *J* = 2.4 Hz, 1 H; *exo*), 2.65 (dt, *J* = 16.2 Hz, *J* = 2.3 Hz, 1 H; *exo*), 2.59–2.09 (m, 7.25 H), 2.06–1.92 (m, 1 H; *exo*) ppm.

Isomerization: Ion-exchange resin Amberlyst15(H) (30 mg, 20% w/w) was placed in a thick-walled reaction vial. The mixture of isomers **16g** and **17g** (0.151 g, 0.545 mmol) was dissolved in CH_2Cl_2 (1.4 mL) and transferred to the vial. After rinsing with additional CH_2Cl_2 (3 × 0.2 mL) the vial was screwed tightly and the resulting mixture was stirred at 50°C for 16 h. The mixture was then filtered over SiO_2 (1.5 cm) and the solvent was removed under reduced pressure. The residue was submitted to column chromatography (SiO_2, hexanes/MTBE 5:1, R_f = 0.22) to furnish the product (*S*)-**17g** (0.146 g, 0.527 mmol, 97%, 94% *ee*) as a colorless solid and exclusively as the *endo*-isomer; mp. 134.9–139.6°C. ^1H NMR (500 MHz, $CDCl_3$): δ = 7.44 (dd, *J* = 8.0 Hz, *J* = 1.7 Hz, 1 H), 7.35 (d, *J* = 1.8 Hz, 1 H), 7.13 (d, *J* = 8.0 Hz, 1 H), 6.04 (q, *J* = 1.6 Hz, 1 H), 2.61–2.52 (m, 2 H), 2.44–2.37 (m, 1 H), 2.35–2.27 (m, 2 H), 2.24–2.16 (m, 1 H), 2.13 (d, *J* = 1.6 Hz, 3 H) ppm. $^{13}C\{^1H\}$ NMR (125 MHz, $CDCl_3$): δ = 217.1 (C), 149.6 (C), 144.9 (C), 140.7 (C), 132.3 (CH), 130.4 (CH), 125.3 (CH), 120.9 (CH), 119.7 (C), 67.0 (C), 38.8 (CH_2), 33.2 (CH_2), 20.3 (CH_2), 12.9 (CH_3) ppm. IR (ATR): 3049 (w), 2966 (m), 2946 (m), 2913 (m), 2882 (m), 1724 (vs), 1612 (w), 1594 (w), 1566 (m), 1460 (s), 1446 (m), 1403 (s), 1379 (m),

1344 (w), 1309 (m), 1276 (m), 1262 (m), 1234 (w), 1190 (w), 1176 (w), 1160 (s), 1132 (s), 1119 (s), 1089 (m), 1061 (s), 1047 (m), 1000 (m), 981 (w), 947 (w), 931 (m), 886 (m), 866 (w), 844 (m), 820 (vs), 794 (s), 769 (s), 751 (w), 740 (w), 699 (w), 639 (w), 626 (m), 593 (m), 581 (m), 577 (m), 567 (m), 543 (w), 493 (w) cm^{-1}. HRMS (ESI, pos. mode) *m/z*: [M + H^+] calcd. for $C_{14}H_{14}BrO^+$ 277.0223; found 277.0222. GLC (Hydrodex β6-TBDM; 100°C, then 5 K min^{-1} to 140°C, then 0.35 K min^{-1} to 158°C, then 5 K min^{-1} to 200°C, 7.5 min hold): $t_R(S)$ = 44.42 min (major), $t_R(R)$ = 45.04 min (minor), 94% *ee*. $[\alpha]_D^{20}$ = +369.13° (CH_2Cl_2, 0.11 g/100 mL). $C_{14}H_{13}BrO$ (277.16 g mol^{-1}).

The racemic product *rac*-**17g** was obtained by the same procedure using *rac*-**15g** as starting material (0.328 mmol-scale; *Heck reaction*: 74% yield, *exo*/*endo* 4:1; *isomerization*: 97% yield, only *endo*).

4. Friedel-Crafts Reactions

4.1 *rac*-3'-(2,4-Dimethoxyphenyl)-3'-methyl-2',3'-dihydrospiro[cyclopentane-1,1'-indene]-2-one (18a)

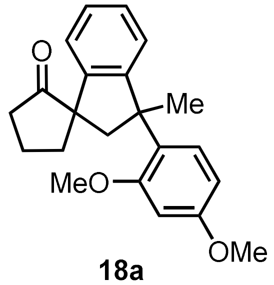

18a

Spiro compound *rac*-**17a** (20 mg, 0.10 mmol) was placed in a thick-walled reaction vial and 1,3-dimethoxybenzene (0.5 mL) was added. TfOH (2 mg, 0.02 mmol) was then added and the vial was screwed tightly. The resulting mixture was stirred at 0°C for 16 h and then submitted to column chromatography (SiO_2, hexanes/MTBE 8:1, R_f = 0.14) to furnish the product **18a** (29 mg, 86 µmol, 86%, *dr* 5:1) as a mixture of diastereomers as a light orange solid; mp. 94.7–97.1°C. ^{1}H NMR (500 MHz, $CDCl_3$): δ = 7.27–7.22 (m, 2 H; major), 7.21–7.18 (m, 0.4 H; minor), 7.15 (d, *J* = 8.5 Hz, 0.2 H; minor), 7.10 (dd, *J* = 7.5 Hz, *J* = 1.4 Hz, 1 H; major), 7.03–7.01 (m, 0.2 H; minor), 6.99 (dd, *J* = 7.6 Hz, *J* = 1.4 Hz, 1 H; major), 6.97–6.94 (m, 0.2 H; minor), 6.72 (d, *J* = 8.6 Hz, 1 H), 6.47 (d, *J* = 2.5 Hz, 1 H; major), 6.42 (d, *J* = 2.5 Hz, 0.2 H; minor), 6.39 (dd, *J* = 8.5 Hz, *J* = 2.5 Hz, 0.2 H; minor), 6.30 (dd, *J* = 8.6 Hz, *J* = 2.5 Hz, 1 H; major), 3.78 (s, 0.6 H; minor), 3.77 (s, 3 H; major), 3.70 (s, 3 H; major), 3.60 (s, 0.6 H; minor), 2.93 (d, *J* = 13.1 Hz, 0.2 H; minor), 2.65 (d, *J* = 12.9 Hz, 1 H; major), 2.43–2.37 (m, 2.2 H), 2.36–2.34 (m, 0.2 H; minor), 2.34 (d, *J* = 12.9 Hz, 1 H; major), 2.30–2.27 (m, 0.4 H; minor), 2.16–2.11 (m, 0.2 H; minor), 2.05–1.99 (m, 1.2 H), 1.98–1.89 (m, 1.2 H), 1.88–1.81 (m, 2 H; major), 1.74 (s, 3 H; major), 1.68 (s, 0.6 H; minor) ppm. $^{13}C\{^1H\}$ NMR (125 MHz, $CDCl_3$): δ = 222.3 (C; major), 220.4

(C; minor), 159.52 (C; minor), 159.48 (C; major), 158.6 (C; minor), 158.4 (C; major), 153.0 (C; minor), 151.8 (C; major), 146.1 (C; major), 145.1 (C; minor), 129.7 (C; major), 128.7 (C; minor), 128.6 (CH; major), 128.2 (CH; minor), 127.2 (CH; major), 127.1 (CH; major, CH; minor), 126.7 (CH; minor), 124.6 (CH; major), 123.8 (CH; minor), 123.4 (CH; minor), 123.3 (CH; major), 103.1 (CH; major), 103.0 (CH; minor), 100.0 (CH; major), 99.8 (CH; minor), 61.2 (C; major), 61.0 (C; minor), 55.2 (CH_3; major, CH_3; minor), 55.1 (CH_3; major), 54.9 (CH_3; minor), 51.2 (CH_2; minor), 51.1 (CH_2; major), 50.22 (C, major), 50.18 (C, minor), 40.7 (CH_2; minor), 38.9 (CH_2; major), 38.1 (CH_2; major), 37.3 (CH_2; minor), 30.0 (CH_3; minor), 27.6 (CH_3; major), 19.7 (CH_2; major), 19.5 (CH_2; minor) ppm. IR (ATR): 2959 (m), 2926 (m), 2856 (m), 2836 (w), 1734 (s), 1610 (s),1582 (m), 1502 (s), 1464 (m), 1439 (m), 1414 (m), 1369 (w), 1303 (m), 1289 (m), 1272 (m), 1256 (m), 1207 (s), 1160 (s), 1147 (m), 1127 (m), 1082 (w), 1034 (s), 937 (w), 926 (w), 834 (m), 797 (w), 763 (m), 754 (m), 731 (w), 637 (w), 543 (m) cm^{-1}. HRMS (EI, 70 eV) *m/z*: [M^+] calcd. for $C_{22}H_{24}O_3^+$ 336.1720; found 336.1714. $C_{22}H_{24}O_3$ (336.43 g mol^{-1}).

4.2 *rac*-3'-(2-Furyl)-3'-methyl-2',3'-dihydrospiro[cyclopentane-1,1'-indene]-2-one (18b)

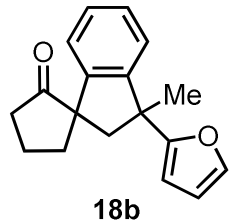

18b

Spiro compound *rac*-**17a** (50 mg, 0.25 mmol) was placed in a thick-walled reaction vial and furan (1.25 mL) was added. TfOH (6 mg, 0.04 mmol) was then added and the vial was screwed tightly. The resulting mixture was stirred at 0°C for 16 h and then submitted to column chromatography (SiO_2, hexanes/MTBE 8:1) to furnish the product **18ba** (22 mg, 83 µmol, 33%, R_f = 0.21) as a colorless oil and as single diastereomer in the first fraction. In a second fraction a mixture of diastereomers **18b** (27 mg, 0.10 mmol, 40%, *dr* 4.5:1, R_f = 0.15) was obtained as a colorless oil. Overall yield: 49 mg, 0.18 mmol, 72%, *dr* 9:1. Isomer **18ba** (first fraction): ^1H NMR (500 MHz, $CDCl_3$): δ = 7.33–7.31 (m, 1 H), 7.28–7.24 (m, 2 H), 7.21–7.18 (m, 1 H), 7.03–6.99 (m, 1 H), 6.23 (dd, *J* = 3.3 Hz, *J* = 1.8 Hz, 1 H), 5.84 (dd, *J* = 3.2 Hz, *J* = 0.8 Hz, 1 H), 2.50 (d, *J* = 13.1 Hz, 1 H), 2.47–2.43 (m, 2 H), 2.39 (d, *J* = 13.1 Hz, 1 H), 2.18–2.06 (m, 3 H), 1.98–1.91 (m, 1 H), 1.71 (s, 3 H) ppm. $^{13}C\{^1H\}$ NMR (125 MHz, $CDCl_3$): δ = 221.4 (C), 161.4 (C), 148.7 (C), 145.5 (C), 141.4 (CH), 127.9 (CH), 127.8 (CH), 124.0 (CH), 123.4 (CH), 109.8 (CH), 104.7 (CH), 60.8 (C), 49.9 (CH_2), 48.2 (C), 39.3 (CH_2), 37.9 (CH_2), 27.3 (CH_3), 19.6 (CH_2) ppm. IR (ATR): 2964 (m), 2930 (m), 2867 (w), 1797 (m), 1762 (m), 1734 (s), 1504 (w), 1480 (w), 1453 (w), 1406 (w), 1312 (w), 1156 (m), 1132 (w), 1077 (w), 1011 (m), 929 (w), 759 (m), 736 (m), 494 (w) cm^{-1}. HRMS (ESI, pos. mode) *m/z*: [M + H^+] calcd. for $C_{18}H_{19}O_2^+$ 267.1380; found 267.1380. $C_{18}H_{18}O_2$ (266.34 g mol^{-1}). Mixture of diastereomers 4.5:1 (second fraction): ^1H

NMR (500 MHz, $CDCl_3$): δ = 7.34–7.33 (m, 0.22 H; minor), 7.32–7.31 (m, 1 H; major), 7.27–7.25 (m, 2 H; major), 7.24–7.22 (m, 0.44 H; minor), 7.21–7.18 (m, 1 H; major), 7.12–7.09 (m, 0.22 H; minor), 7.03–6.99 (m, 1.22 H), 6.29 (dd, *J* = 3.2 Hz, *J* = 1.8 Hz, 0.22 H; minor), 6.23 (dd, *J* = 3.2 Hz, *J* = 1.8 Hz, 1 H; major), 6.13 (dd, *J* = 3.3 Hz, *J* = 0.8 Hz, 0.22 H; minor), 5.84 (dd, *J* = 3.3 Hz, *J* = 0.8 Hz, 1 H; major), 2.96 (d, *J* = 13.1 Hz, 0.22 H; minor), 2.50 (d, *J* = 13.1 Hz, 1 H; major), 2.47–2.43 (m, 2.22 H), 2.39 (d, *J* = 13.1 Hz, 1 H; major), 2.33–2.30 (m, 0.44 H; minor), 2.22–2.03 (m, 3.44 H), 2.02 (d, *J* = 13.1 Hz, 0.22 H; minor), 2.00–1.89 (m, 1.22 H), 1.71 (s, 3 H; major), 1.66 (s, 0.66 H; minor) ppm. $^{13}C\{^1H\}$ NMR (125 MHz, $CDCl_3$): δ = 221.8 (C; major), 221.0 (C; minor), 161.3 (C; major), 160.4 (C; minor), 149.4 (C; minor), 148.7 (C; major), 145.4 (C; major), 144.9 (C; minor), 141.6 (CH; minor), 141.4 (CH; major), 127.9 (CH; major), 127.84 (CH; minor), 127.82 (CH; minor), 127.77 (CH; major), 123.93 (CH; major), 123.87 (CH; minor), 123.41 (CH; minor), 123.35 (CH; major), 109.83 (CH; minor), 109.80 (CH; major), 104.8 (CH; minor), 104.7 (CH; major), 60.8 (C; major), 60.5 (C; minor), 49.8 (CH_2; major), 49.7 (CH_2; minor), 48.4 (C; minor), 48.1 (C; major), 40.3 (CH_2; minor), 39.2 (CH_2; major), 37.9 (CH_2; major), 37.7 (CH_2; minor), 28.5 (CH_3; minor), 27.3 (CH_3; major), 19.6 (CH_2; major, CH_2; minor) ppm.

4.3 *rac*-3'-Methyl-3'-(2-thienyl)-2',3'-dihydrospiro[cyclopentane-1,1'-indene]-2-one (18c)

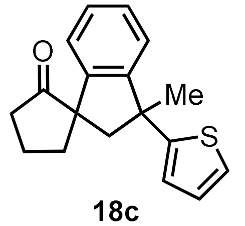

18c

Spiro compound *rac*-**17a** (99 mg, 0.50 mmol) was placed in a thick-walled reaction vial and thiophene (2.5 mL) was added. TfOH (11 mg, 75 μmol) was then added and the vial was screwed tightly. The resulting mixture was stirred at ambient temperature for 16 h and then submitted to column chromatography (SiO_2, hexanes/MTBE 10:1) to furnish the product **18ca** (32 mg, 0.11 mmol, 22%, R_f = 0.17) as a colorless solid and as single diastereomer in the first fraction. In a second fraction a mixture of diastereomers **18c** (47 mg, 0.17 mmol, 34%, *dr* 9:8, R_f = 0.16) with unknown impurities was obtained as a colorless oil. Overall yield: 79 mg, 0.28 mmol, 56%, *dr* 18:8. Isomer **18ca** (first fraction): Mp. 88.9–93.1°C. ^1H NMR (500 MHz, $CDCl_3$): δ = 7.29–7.22 (m, 3 H), 7.11 (dd, *J* = 5.1 Hz, *J* = 1.2 Hz, 1 H), 6.98 (dd, *J* = 6.3 Hz, *J* = 1.6 Hz, 1 H), 6.83 (dd, *J* = 5.1 Hz, *J* = 3.5 Hz, 1 H), 6.57 (dd, *J* = 3.6 Hz, *J* = 1.2 Hz, 1 H), 2.56 (d, *J* = 13.0 Hz, 1 H), 2.43–2.39 (m, 2 H), 2.38 (d, *J* = 13.0 Hz, 1 H), 2.09–1.96 (m, 3 H), 1.91–1.84 (m, 1 H), 1.78 (s, 3 H) ppm. $^{13}C\{^1H\}$ NMR (125 MHz, $CDCl_3$): δ = 221.7 (C), 155.8 (C), 150.0 (C), 145.4 (C), 128.0 (CH), 127.8 (CH), 126.3 (CH), 124.2 (CH), 123.54 (CH), 123.51 (CH),

123.4 (CH), 60.8 (C), 54.4 (CH_2), 50.0 (C), 39.1 (CH_2), 38.0 (CH_2), 30.8 (CH_3), 19.7 (CH_2) ppm. IR (ATR): 3067 (w), 3020 (w), 2959 (m), 2924 (m), 2864 (w), 1734 (vs), 1600 (w), 1479 (m), 1454 (m), 1404 (w), 1373 (w), 1350 (w), 1310 (w), 1267 (w), 1232 (w), 1156 (m), 1134 (w), 1084 (w), 1050 (w), 1016 (w), 1001 (w), 923 (w), 850 (w), 830 (w), 780 (w), 759 (s), 696 (s), 606 (w), 563 (w), 536 (m), 509 (w) cm^{-1}. HRMS (ESI, pos. mode) *m/z*: [M + Na^+] calcd. for $C_{18}H_{18}NaOS^+$ 305.0971; found 305.0968. $C_{18}H_{18}OS$ (282.40 g mol^{-1}). Mixture of diastereomers 9:8 (second fraction): ^1H NMR (500 MHz, $CDCl_3$): δ = 7.32–7.23 (m, 4.64 H), 7.18–7.12 (m, 2.88 H), 7.03–7.01 (m, 1.88 H), 6.94 (dd, *J* = 5.1 Hz, *J* = 3.6 Hz, 1 H; major), 6.90 (dd, *J* = 3.5 Hz, *J* = 1.2 Hz, 1 H; major), 6.86 (dd, *J* = 5.1 Hz, *J* = 3.5 Hz, 0.88 H; minor), 6.61 (dd, *J* = 3.6 Hz, *J* = 1.2 Hz, 0.88 H; minor), 2.86 (d, *J* = 13.2 Hz, 1 H; major), 2.72–2.62 (m, 1 H; major), 2.60 (d, *J* = 12.9 Hz, 0.88 H; minor), 2.51–2.38 (m, 4.64 H), 2.24–2.19 (m, 1 H; major), 2.15–2.00 (m, 4.64 H), 1.97–1.86 (m, 1.88 H), 1.82 (3 H; major), 1.78 (s, 2.64 H; minor) ppm. $^{13}C\{^1H\}$ NMR (125 MHz, $CDCl_3$): δ = 221.6 (C; minor), 220.7 (C; major), 155.8 (C; minor), 154.6 (C; major), 151.3 (C; major), 150.0 (C; minor), 145.4 (C; minor), 144.9 (C; major), 128.0 (CH; minor), 127.9 (CH; major), 127.84 (CH; major), 127.80 (CH; minor), 126.6 (CH; major), 126.3 (CH; minor), 124.23 (CH; major), 124.20 (CH; minor), 123.8 (CH; major), 123.53 (CH; minor), 123.50 (CH; minor), 123.46 (CH; major), 123.4 (CH; major & minor), 60.8 (C; minor), 60.6 (C; major), 54.8 (CH_2; major), 54.4 (CH_2; minor), 50.0 (C; major), 49.8 (C; minor), 40.2 (CH_2; major), 39.1 (CH_2; minor), 37.9 (CH_2; minor), 37.6 (CH_2; major), 31.3 (CH_3; major), 30.8 (CH_3; minor), 19.7 (CH_2; minor), 19.6 (CH_2; major) ppm.

4.4 *trans*-3'-(Benzofuran-2-yl)-3'-methyl-2',3'-dihydrospiro[cyclopentane-1,1'-indene]-2-one (18d)

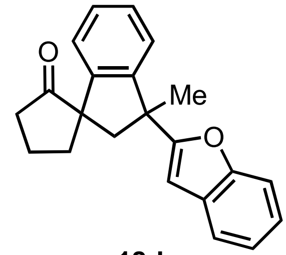

18d

Spiro compound *rac*-**17a** (20 mg, 0.10 mmol) was placed in a thick-walled reaction vial and benzofuran (0.5 mL) was added. TfOH (2 mg, 0.02 mmol) was then added and the vial was screwed tightly. The resulting mixture was stirred at ambient temperature for 16 h and then submitted to column chromatography (SiO_2, hexanes/MTBE 8:1, R_f = 0.17) to furnish the product **18d** (17 mg, 54 µmol, 54%) as *trans*-isomer and as a light-yellow resin. The relative configuration was established by a NOE effect of the benzofuran-3H atom to one of the H-atoms (δ = 2.63 ppm) in α-position to the carbonyl group. ^1H NMR (500 MHz, $CDCl_3$): δ = 7.44 (dd, *J* = 7.5 Hz, *J* = 1.4 Hz, 1 H), 7.41 (d, *J* = 8.1 Hz, 1 H), 7.31–7.27 (m, 3 H), 7.22 (td, *J* = 8.2

Hz, J = 1.5 Hz, 1 H), 7.17 (td, J = 7.5 Hz, J = 1.1 Hz, 1 H), 7.06–7.03 (m, 1 H), 6.20 (s, 1 H), 2.63 (d, J = 13.1 Hz, 1 H), 2.49–2.44 (m, 3 H), 2.21–2.14 (m, 1 H), 2.14–2.06 (m, 2 H), 1.98–1.89 (m, 1 H), 1.81 (s, 3 H) ppm. $^{13}C\{^1H\}$ NMR (125 MHz, $CDCl_3$): δ = 221.6 (C), 164.4 (C), 154.9 (C), 148.0 (C), 145.8 (C), 128.5 (C), 128.2 (CH), 127.9 (CH), 124.1 (CH), 123.5 (2 CH), 122.5 (CH), 120.5 (CH), 111.0 (CH), 101.9 (CH), 60.9 (C), 49.7 (CH_2), 48.5 (C), 39.3 (CH_2), 38.0 (CH_2), 27.0 (CH_3), 19.7 (CH_2) ppm. IR (ATR): 3064 (w), 2963 (m), 2929 (m), 2869 (w), 1812 (w), 1734 (s), 1613 (w), 1596 (m), 1577 (m), 1479 (s), 1453 (s), 1404 (w), 1374 (w), 1312 (w), 1292 (w), 1254 (s), 1239 (m), 1164 (m), 1137 (w), 1110 (w), 1077 (m), 1057 (w), 1004 (w), 970 (w), 941 (m), 910 (w), 880 (w), 836 (w), 807 (m), 773 (m), 750 (vs), 690 (w), 649 (w), 614 (w), 566 (w), 547 (w), 534 (w) cm^{-1}. HRMS (ESI, pos. mode) *m/z*: [M + Na^+] calcd. for $C_{22}H_{20}NaO_2^+$ 339.1356; found 339.1354. $C_{22}H_{20}O_2$ (316.40 g mol^{-1}).

5. α-(*ortho*-Iodophenylation) of β-Oxoesters

5.1 *rac*-Allyl 1-(2-iodophenyl)-2-oxocyclopentane-1-carboxylate (14a)

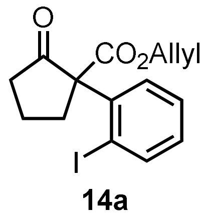

14a

Based on a literature procedure,[S5] anhydrous MeCN (15 mL) and TFA (15 mL) were added to a mixture of K_2SO_4 (10.4 g, 59.4 mmol) and phenyliodobis(trifluoroacetate) (14.1 g, 32.7 mmol) under nitrogen atmosphere. Subsequently, the β-oxoester **S6** (5.0 g, 30 mmol) was added and the resulting mixture was stirred at ambient temperature for 16 h. All volatiles were removed under reduced pressure, the residue was slurried in CH_2Cl_2 (ca. 100 mL), filtered through a glass frit and the residue rinsed with additional CH_2Cl_2 (3 × 30 mL). The filtrate was evaporated and the residue was submitted to column chromatography (SiO_2, hexanes/MTBE 10:1, R_f = 0.15) to furnish the α-arylated β-oxoester **14a** (3.48 g, 9.40 mmol, 31%) as a colorless oil. ^1H NMR (300 MHz, $CDCl_3$): δ = 7.95–7.89 (m, 1 H), 7.27 (td, J = 7.7 Hz, J = 1.4 Hz, 1 H), 6.99–6.91 (m, 2 H), 5.87 (ddt, J = 17.3 Hz, J = 10.8 Hz, J = 5.6 Hz, 1 H), 5.22 (dq, J = 17.2 Hz, J = 1.6 Hz, 1 H), 5.15 (dq, J = 10.5 Hz, J = 1.4 Hz, 1 H), 4.70 (ddt, J = 13.3 Hz, J = 5.6 Hz, J = 1.5 Hz, 1 H), 4.60 (ddt, J = 13.3 Hz, J = 5.6 Hz, J = 1.5 Hz, 1 H), 3.20 (ddd, J = 13.7 Hz, J = 9.8 Hz, J = 6.9 Hz, 1 H), 2.61–2.46 (m, 3 H), 2.15–2.03 (m, 1 H), 1.71 (dddd, J = 18.8 Hz, J = 13.0 Hz, J = 9.1 Hz, J = 6.7 Hz, 1 H) ppm. $^{13}C\{^1H\}$ NMR (75 MHz, $CDCl_3$): δ = 213.4 (C), 169.2 (C), 141.9 (CH), 141.3 (C), 131.4 (CH), 128.8 (CH), 128.6 (CH), 128.0 (CH), 118.2 (CH_2), 98.7 (C), 70.0 (C), 66.7 (CH_2), 39.4 (CH_2), 36.1 (CH_2), 19.2 (CH_2) ppm. IR (ATR): 2980 (w), 2954 (w), 2884 (w), 1750 (s), 1720 (vs), 1647 (w), 1464 (m), 1403 (m), 1256 (m), 1223 (s), 1177 (m), 1127 (m), 1080 (m), 1010 (m), 984 (m), 926 (m), 843 (w), 753 (m), 720 (m), 640 (m), 537 (w)

cm^{-1}. HRMS (EI, 70 eV) *m/z*: [M$^+$] calcd. for $C_{15}H_{15}IO_3^+$ 370.0060; found 370.0055. $C_{15}H_{15}IO_3$ (370.19 g mol^{-1}).

5.2 *rac*-Allyl 1-(2-iodophenyl)-2-oxocyclohexane-1-carboxylate (14b)

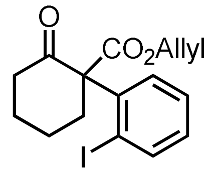

14b

Based on a literature procedure,[S5] anhydrous MeCN (12 mL) and TFA (12 mL) were added to a mixture of K_2SO_4 (8.16 g, 46.8 mmol) and phenyliodobis(trifluoroacetate) (11.1 g, 25.8 mmol) under nitrogen atmosphere. Subsequently, the β-oxoester **S8** (4.27 g, 23.4 mmol) was added and the resulting mixture was stirred at ambient temperature for 16 h. All volatiles were removed under reduced pressure, the residue was slurried in CH_2Cl_2 (ca. 100 mL), filtered through a glass frit and the residue rinsed with additional CH_2Cl_2 (3 × 30 mL). The filtrate was evaporated and the residue was submitted to column chromatography (SiO_2, hexanes/MTBE 8:1, R_f = 0.18) to furnish the α-arylated β-oxoester **14b** (3.61 g, 9.40 mmol, 40%) as a colorless oil. ^{1}H NMR (500 MHz, $CDCl_3$): δ = 7.96 (dd, *J* = 7.9 Hz, *J* = 1.3 Hz, 1 H), 7.32 (td, *J* = 7.6 Hz, *J* = 1.4 Hz, 1 H), 7.07 (dd, *J* = 8.0 Hz, *J* = 1.6 Hz, 1 H), 6.96 (td, *J* = 7.6 Hz, *J* = 1.6 Hz, 1 H), 5.89 (ddt, *J* = 17.1 Hz, *J* = 10.5 Hz, *J* = 5.7 Hz, 1 H), 5.24 (dq, *J* = 17.3 Hz, *J* = 1.6 Hz, 1 H), 5.21 (dq, *J* = 11.8 Hz, *J* = 1.3 Hz, 1 H), 4.73 (ddt, *J* = 13.3 Hz, *J* = 5.8 Hz, *J* = 1.5 Hz, 1 H), 4.68 (ddt, *J* = 13.2 Hz, *J* = 5.8 Hz, *J* = 1.5 Hz, 1 H), 2.83 (ddd, *J* = 14.8 Hz, *J* = 9.9 Hz, *J* = 7.6 Hz, 1 H), 2.76 (ddd, *J* = 14.8 Hz, *J* = 10.9 Hz, *J* = 4.1 Hz, 1 H), 2.69 (dtd, *J* = 14.8 Hz, *J* = 4.9 Hz, *J* = 1.2 Hz, 1 H), 2.62 (dtd, *J* = 13.9 Hz, *J* = 3.5 Hz, *J* = 1.3 Hz, 1 H), 2.04–1.97 (m, 2 H), 1.89–1.74 (m, 2 H) ppm. ^{13}C{^{1}H} NMR (125 MHz, $CDCl_3$): δ = 204.9 (C), 170.0 (C), 142.3 (CH), 141.1 (C), 131.2 (CH), 129.2 (CH), 128.9 (CH), 128.0 (CH), 119.0 (CH_2), 99.4 (C), 70.0 (C), 66.5 (CH_2), 41.7 (CH_2), 36.3 (CH_2), 26.1 (CH_2), 22.1 (CH_2) ppm. IR (ATR): 2943 (m), 2867 (w), 1732 (s), 1716 (vs), 1452 (m), 1234 (m), 1206 (s), 1136 (m), 1082 (m), 1011 (m), 933 (m), 749 (s), 554 (w) cm^{-1}. HRMS (EI, 70 eV) *m/z*: [M$^+$] calcd. for $C_{16}H_{17}IO_3^+$ 384.0217; found 384.0217. $C_{16}H_{17}IO_3$ (384.21 g mol^{-1}).

5.3 *rac*-Allyl 1-(2-iodophenyl)-2-oxocycloheptane-1-carboxylate (14c)

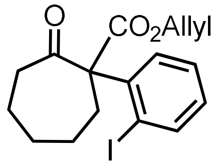

14c

Based on a literature procedure,[S5] anhydrous MeCN (15 mL) and TFA (15 mL) were added to a mixture of K_2SO_4 (5.29 g, 30.4 mmol) and phenyliodobis(trifluoroacetate) (7.19 g, 16.7 mmol) under nitrogen atmosphere. Subsequently, the β-oxoester **S10** (2.98 g, 15.2 mmol) was added and the resulting mixture was stirred at ambient temperature for 16 h. All volatiles were removed under reduced pressure, the residue was slurried in CH_2Cl_2 (ca. 100 mL), filtered through a glass frit and the residue rinsed with additional CH_2Cl_2 (3 × 30 mL). The filtrate was evaporated and the residue was submitted to column chromatography (SiO_2, hexanes/acetone 50:1) to partially reisolate the starting material **S10** (0.798 g, 4.07 mmol, 27%, R_f = 0.14) in the first fraction. In a second fraction the α-arylated β-oxoester **14c** (0.832 g, 2.09 mmol, 14%, R_f = 0.07) was obtained as a colorless solid; mp. 94.5–95.7°C. ^{1}H NMR (500 MHz, $CDCl_3$): δ = 7.94 (dd, *J* = 7.9 Hz, *J* = 1.4 Hz, 1 H), 7.31 (td, *J* = 7.7 Hz, *J* = 1.4 Hz, 1 H), 7.03 (dd, *J* = 7.9 Hz, *J* = 1.6 Hz, 1 H), 6.95 (td, *J* = 7.6 Hz, *J* = 1.6 Hz, 1 H), 5.88 (ddt, *J* = 17.3 Hz, *J* = 10.8 Hz, *J* = 5.6 Hz, 1 H), 5.18 (dq, *J* = 17.0 Hz, *J* = 1.5 Hz, 1 H), 5.14 (dq, *J* = 10.5 Hz, *J* = 1.4 Hz, 1 H), 4.68 (ddt, *J* = 13.4 Hz, *J* = 5.5 Hz, *J* = 1.4 Hz, 1 H), 4.64 (ddt, *J* = 13.5 Hz, *J* = 5.6 Hz, *J* = 1.4 Hz, 1 H), 3.23–3.18 (m, 1 H), 2.99 (ddd, *J* = 11.9 Hz, *J* = 7.0 Hz, *J* = 4.7 Hz, 1 H), 2.79–2.74 (m, 1 H), 2.20–2.14 (m, 1 H), 1.82–1.71 (m, 5 H), 1.53–1.46 (m, 1 H) ppm. $^{13}C\{^1H\}$ NMR (125 MHz, $CDCl_3$): δ = 208.1 (C), 170.9 (C), 142.5 (C), 142.3 (CH), 131.7 (CH), 129.7 (CH), 128.7 (CH), 127.7 (CH), 118.0 (CH_2), 98.3 (C), 71.7 (C), 66.3 (CH_2), 43.6 (CH_2), 34.1 (CH_2), 30.4 (CH_2), 26.8 (CH_2), 25.6 (CH_2) ppm. IR (ATR): 3064 (w), 2930 (m), 2860 (w), 1736 (s), 1704 (s), 1647 (w), 1582 (w), 1563 (w), 1463 (m), 1454 (m), 1432 (m), 1337 (w), 1322 (w), 1299 (w), 1249 (m), 1217 (s), 1184 (s), 1153 (s), 1139 (m), 1122 (w), 1097 (w), 1069 (w), 1056 (w), 1009 (s), 993 (m), 937 (m), 884 (w), 836 (w), 767 (w), 744 (s), 713 (w), 647 (w), 637 (w), 629 (w), 576 (w), 536 (w), 500 (w), 483 (w) cm^{-1}. HRMS (EI, 70 eV) *m/z*: [M^+] calcd. for $C_{17}H_{19}IO_3^+$ 398.0373; found 398.0375. $C_{17}H_{19}IO_3$ (398.24 g mol^{-1}).

5.4 *rac*-Allyl 2-(2-iodophenyl)-1-oxoindane-2-carboxylate (14d)

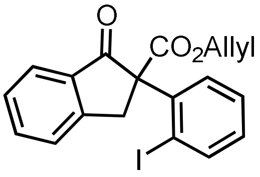

14d

Based on a literature procedure,[S5] anhydrous MeCN (10 mL) and TFA (10 mL) were added to a mixture of K_2SO_4 (3.22 g, 18.5 mmol) and phenyliodobis(trifluoroacetate) (4.39 g, 10.2 mmol) under nitrogen atmosphere. Subsequently, the β-oxoester **S12** (2.01 g, 9.27 mmol) was added and the resulting mixture was stirred at ambient temperature for 16 h. All volatiles were removed under reduced pressure, the residue was slurried in CH_2Cl_2 (ca. 80 mL), filtered through a glass frit and the residue rinsed with additional CH_2Cl_2 (3 × 25 mL). The filtrate was evaporated and the residue was submitted to column chromatography (SiO_2, hexanes/MTBE 20:1, R_f = 0.08) to obtain the α-arylated β-oxoester **14d** (0.723 g) in an impure form as light purple crystals. After carefully washing with a mixture of CH_2Cl_2 and MTBE (1:1, 5 × 2 mL) the α-arylated β-oxoester **14d** (0.561 g, 1.34 mmol, 14%) could be furnished as colorless crystals; mp. 81.0–83.0°C. ^{1}H NMR (500 MHz, $CDCl_3$): δ = 7.95 (dd, *J* = 7.9 Hz, *J* = 1.3 Hz, 1 H), 7.88 (d, *J* = 7.7 Hz, 1 H), 7.64 (td, *J* = 7.5 Hz, *J* = 1.2 Hz, 1 H), 7.47 (d, *J* = 7.7 Hz, 1 H), 7.42 (t, *J* = 7.5 Hz, 1 H), 7.26–7.23 (m, 1 H), 7.19 (dd, *J* = 8.0 Hz, *J* = 1.7 Hz, 1 H), 6.94 (td, *J* = 7.6 Hz, *J* = 1.7 Hz, 1 H), 5.89 (ddt, *J* = 16.2 Hz, *J* = 10.9 Hz, *J* = 5.7 Hz, 1 H), 5.24 (dq, *J* = 17.2 Hz, *J* = 1.6 Hz, 1 H), 5.16 (dq, *J* = 10.5 Hz, *J* = 1.4 Hz, 1 H), 4.73 (ddt, *J* = 13.3 Hz, *J* = 5.6 Hz, *J* = 1.5 Hz, 1 H), 4.67 (d, *J* = 17.7 Hz, 1 H), 4.64 (ddt, *J* = 13.3 Hz, *J* = 5.5 Hz, *J* = 1.6 Hz, 1 H), 3.37 (d, *J* = 17.4 Hz, 1 H) ppm. ^{13}C{^{1}H} NMR (125 MHz, $CDCl_3$): δ = 200.3 (C), 168.9 (C), 153.0 (C), 143.0 (C), 141.4 (CH), 136.1 (CH), 134.9 (C), 131.4 (CH), 129.0 (CH), 128.8 (CH), 128.1 (CH), 127.9 (CH), 126.4 (CH), 125.1 (CH), 118.4 (CH_2), 99.8 (C), 69.8 (C), 67.0 (CH_2), 42.0 (CH_2) ppm. IR (ATR): 3057 (w), 3020 (w), 2933 (w), 2880 (w), 1744 (s), 1726 (s), 1709 (vs), 1647 (w), 1606 (m), 1590 (m), 1563 (w), 1477 (w), 1463 (s), 1430 (m), 1420 (m), 1374 (w), 1359 (w), 1324 (w), 1289 (m), 1273 (s), 1246 (s), 1222 (s), 1209 (vs), 1166 (s), 1096 (w), 1079 (m), 1057 (m), 1007 (s), 966 (m), 930 (s), 893 (s), 860 (w), 821 (w), 797 (m), 769 (s), 754 (s), 737 (s), 720 (vs), 689 (m), 674 (m), 643 (m), 614 (m), 574 (w), 553 (w), 504 (w) cm^{-1}. HRMS (EI, 70 eV) *m/z*: [M^+] calcd. for $C_{19}H_{15}IO_3^+$ 418.0060; found 418.0058. $C_{19}H_{15}IO_3$ (418.23 g mol^{-1}).

5.5 *rac*-Allyl 2-(2-iodophenyl)-1-oxo-1,2,3,4-tetrahydronaphthalene-2-carboxylate (14e)

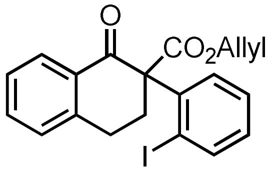

14e

Based on a literature procedure,[S5] anhydrous MeCN (6.5 mL) and TFA (6.5 mL) were added to a mixture of K_2SO_4 (2.18 g, 12.5 mmol) and phenyliodobis(trifluoroacetate) (2.96 g, 6.88 mmol) under nitrogen atmosphere. Subsequently, the β-oxoester **S15** (1.44 g, 6.25 mmol) was added and the resulting mixture was stirred at ambient temperature for 16 h. All volatiles were removed under reduced pressure, the residue was slurried in CH_2Cl_2 (ca. 70 mL), filtered through a glass frit and the residue rinsed with additional CH_2Cl_2 (3 × 25 mL). The filtrate was evaporated and the residue was submitted to a first column chromatography (SiO_2, hexanes/MTBE 10:1, R_f = 0.13) to obtain the α-arylated β-oxoester **14e** (0.652 g) with impurities as a light-yellow solid. After a second column chromatography (SiO_2, hexanes/MTBE 20:1, R_f = 0.04) the α-arylated β-oxoester **14e** (0.415 g, 0.960 mmol, 15%) could be furnished as colorless solid; mp. 124.5–127.3°C. ^{1}H NMR (500 MHz, $CDCl_3$): δ = 8.17 (dd, *J* = 7.9 Hz, *J* = 1.4 Hz, 1 H), 7.95 (dd, *J* = 7.9 Hz, *J* = 1.4 Hz, 1 H), 7.48 (td, *J* = 7.5 Hz, *J* = 1.5 Hz, 1 H), 7.36 (t, *J* = 7.6 Hz, 1 H), 7.16 (d, *J* = 7.7 Hz, 1 H), 7.10 (td, *J* = 7.6 Hz, *J* = 1.4 Hz, 1 H), 6.91 (td, *J* = 7.6 Hz, *J* = 1.6 Hz, 1 H), 6.85 (dd, *J* = 7.9 Hz, *J* = 1.6 Hz, 1 H), 5.94 (ddt, *J* = 17.3 Hz, *J* = 10.8 Hz, *J* = 5.6 Hz, 1 H), 5.25 (dq, *J* = 17.2 Hz, *J* = 1.6 Hz, 1 H), 5.17 (dq, *J* = 10.5 Hz, *J* = 1.4 Hz, 1 H), 4.81 (ddt, *J* = 13.3 Hz, *J* = 5.5 Hz, *J* = 1.5 Hz, 1 H), 4.69 (ddt, *J* = 13.4 Hz, *J* = 5.6 Hz, *J* = 1.5 Hz, 1 H), 3.50–3.45 (m, 1 H), 2.89–2.79 (m, 2 H), 2.48 (ddd, *J* = 17.4 Hz, *J* = 12.1 Hz, *J* = 3.9 Hz, 1 H) ppm. $^{13}C\{^1H\}$ NMR (125 MHz, $CDCl_3$): δ = 194.4 (C), 170.1 (C), 143.3 (C), 142.3 (CH), 139.0 (C), 133.8 (CH), 132.5 (C), 131.6 (CH), 129.9 (CH), 129.0 (CH), 128.6 (CH), 128.0 (CH), 127.8 (CH), 127.0 (CH), 118.1 (CH_2), 97.4 (C), 67.0 (C), 66.5 (CH_2), 31.0 (CH_2), 25.6 (CH_2) ppm. IR (ATR): 3066 (w), 2942 (w), 1732 (m), 1683 (m), 1647 (w), 1599 (m), 1563 (w), 1466 (m), 1454 (m), 1432 (w), 1352 (w), 1332 (w), 1296 (m), 1226 (m), 1192 (m), 1157 (m), 1124 (w), 1114 (w), 1096 (w), 1057 (w), 1029 (w), 1009 (m), 991 (m), 960 (w), 904 (s), 801 (w), 784 (w), 776 (w), 724 (s), 681 (m), 647 (m), 640 (m), 597 (m), 576 (m), 553 (m), 507 (w), 494 (w) cm^{-1}. HRMS (ESI, pos. mode) *m/z*: [M + H^+] calcd. for $C_{20}H_{18}IO_3^+$ 433.0295; found 433.0294. $C_{20}H_{17}IO_3$ (432.26 g mol^{-1}).

5.6 *rac*-Allyl 1-[2-iodo-5-(trifluoromethyl)phenyl]-2-oxocyclopentane-1-carboxylate (14f)

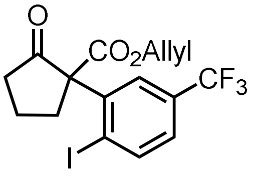

14f

Based on a literature procedure,[S5] anhydrous MeCN (9 mL) and TFA (9 mL) were added to a mixture of K_2SO_4 (3.18 g, 18.2 mmol) and 4-(trifluoromethyl)phenyliodinebis(trifluoroacetate)[S1] (5.00 g, 10.0 mmol) under nitrogen atmosphere. Subsequently, the β-oxoester **S6** (1.53 g, 9.12 mmol) was added and the resulting mixture was stirred at ambient temperature for 16 h. All volatiles were removed under reduced pressure, the residue was slurried in CH_2Cl_2 (ca. 70 mL), filtered through a glass frit and the residue rinsed with additional CH_2Cl_2 (3 × 25 mL). The filtrate was evaporated and the residue was submitted to column chromatography (SiO_2, hexanes/MTBE 10:1, R_f = 0.18) to furnish the α-arylated β-oxoester **14f** (0.371 g, 0.847 mmol, 9%) as a colorless oil. ^{1}H NMR (500 MHz, $CDCl_3$): δ = 8.07 (d, *J* = 8.0 Hz, 1 H), 7.23–7.19 (m, 2 H), 5.87 (ddt, *J* = 17.3 Hz, *J* = 10.6 Hz, *J* = 5.7 Hz, 1 H), 5.24 (dq, *J* = 17.2 Hz, *J* = 1.5 Hz, 1 H), 5.18 (dq, *J* = 10.3 Hz, *J* = 1.3 Hz, 1 H), 4.69 (ddt, *J* = 13.2 Hz, *J* = 5.7 Hz, *J* = 1.5 Hz, 1 H), 4.62 (ddt, *J* = 13.3 Hz, *J* = 5.7 Hz, *J* = 1.4 Hz, 1 H), 3.27 (ddd, *J* = 13.6 Hz, *J* = 8.7 Hz, *J* = 7.0 Hz, 1 H), 2.63–2.57 (m, 2 H), 2.45–2.51 (m, 1 H), 2.16 (dtt, *J* = 11.6 Hz, *J* = 7.6 Hz, *J* = 6.0 Hz, 1 H), 1.76 (dqd, *J* = 13.0 Hz, *J* = 8.6 Hz, *J* = 6.8 Hz, 1 H) ppm. $^{13}C\{^1H\}$ NMR (125 MHz, $CDCl_3$): δ = 212.3 (C), 168.5 (C), 142.7 (C), 142.5 (CH), 131.2 (CH), 130.5 (q, *J* = 32.9 Hz, C), 125.4 (q, *J* = 3.6 Hz, CH), 125.0 (q, *J* = 3.9 Hz, CH), 123.6 (q, *J* = 273 Hz, CF_3), 118.7 (CH_2), 103.2 (C), 69.7 (C), 67.0 (CH_2), 39.4 (CH_2), 36.1 (CH_2), 19.3 (CH_2) ppm. $^{19}F\{^1H\}$ NMR (470 MHz, $CDCl_3$): δ = –62.85 (s) ppm. IR (ATR): 3087 (w), 2982 (w), 2959 (w), 2887 (w), 1753 (s), 1722 (vs), 1649 (w), 1604 (w), 1464 (w), 1453 (w), 1399 (m), 1327 (vs), 1289 (m), 1276 (w), 1256 (w), 1214 (m), 1169 (s), 1123 (vs), 1089 (s), 1074 (s), 1036 (w), 1011 (s), 984 (m), 954 (m), 927 (m), 890 (m), 826 (s), 809 (m), 747 (w), 724 (m), 683 (w), 644 (w), 594 (w), 537 (m), 521 (w) cm^{-1}. HRMS (ESI, pos. mode) *m/z*: [M + H^+] calcd. for $C_{16}H_{15}F_3IO_3^+$ 439.0013; found 439.0009. $C_{16}H_{14}F_3IO_3$ (438.18 g mol^{-1}).

5.7 *rac*-Allyl 1-(5-bromo-2-iodophenyl)-2-oxocyclopentane-1-carboxylate (14g)

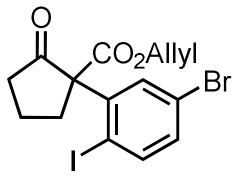

14g

Based on a literature procedure,[S5] anhydrous MeCN (10 mL) and TFA (10 mL) were added to a mixture of K_2SO_4 (3.49 g, 20.0 mmol) and 4-bromophenyliodinebis(trifluoroacetate)[S1] (5.60 g, 11.0 mmol) under nitrogen atmosphere. Subsequently, the β-oxoester **S6** (1.68 g, 10.0 mmol) was added and the resulting mixture was stirred at ambient temperature for 16 h. All volatiles were removed under reduced pressure, the residue was slurried in CH_2Cl_2 (ca. 80 mL), filtered through a glass frit and the residue rinsed with additional CH_2Cl_2 (3 × 25 mL). The filtrate was evaporated and the residue was submitted to a first column chromatography (SiO_2, hexanes/MTBE 10:1, R_f = 0.24) to obtain the α-arylated β-oxoester **14g** (0.72 g) with impurities as a light-yellow oil. After a second column chromatography (SiO_2, hexanes/MTBE 10:1, R_f = 0.24) the α-arylated β-oxoester **14g** (0.660 g, 1.47 mmol, 15%) could be furnished as colorless solid; mp. 62.7–64.5°C. ^{1}H NMR (500 MHz, $CDCl_3$): δ = 7.77 (d, *J* = 8.0 Hz, 1 H), 7.12–7.09 (m, 2 H), 5.88 (ddt, *J* = 17.3 Hz, *J* = 10.5 Hz, *J* = 5.7 Hz, 1 H), 5.25 (dq, *J* = 17.2 Hz, *J* = 1.6 Hz, 1 H), 5.19 (dq, *J* = 10.4 Hz, *J* = 1.3 Hz, 1 H), 4.70 (ddt, *J* = 13.2 Hz, *J* = 5.7 Hz, *J* = 1.4 Hz, 1 H), 4.61 (ddt, *J* = 13.2 Hz, *J* = 5.7 Hz, *J* = 1.4 Hz, 1 H), 3.24 (ddd, *J* = 13.7 Hz, *J* = 9.2 Hz, *J* = 7.0 Hz, 1 H), 2.64–2.52 (m, 2 H), 2.48 (dddd, *J* = 15.0 Hz, *J* = 6.5 Hz, *J* = 4.3 Hz, *J* = 1.8 Hz, 1 H), 2.17–2.09 (m, 1 H), 1.77 (dqd, *J* = 13.0 Hz, *J* = 8.8 Hz, *J* = 6.8 Hz, 1 H) ppm. $^{13}C\{^1H\}$ NMR (125 MHz, $CDCl_3$): δ = 212.6 (C), 168.6 (C), 143.5 (C), 143.0 (CH), 132.0 (CH), 131.8 (CH), 131.3 (CH), 122.6 (C), 118.7 (CH_2), 96.7 (C), 69.6 (C), 67.0 (CH_2), 39.5 (CH_2), 36.1 (CH_2), 19.3 (CH_2) ppm. IR (ATR): 3084 (w), 2953 (w), 2883 (w), 1752 (s), 1720 (vs), 1647 (w), 1569 (w), 1544 (w), 1453 (m), 1423 (w), 1403 (w), 1370 (m), 1316 (w), 1273 (m), 1246 (m), 1216 (s), 1177 (m), 1132 (m), 1112 (m), 1099 (m), 1077 (m), 1034 (w), 1009 (s), 984 (m), 949 (m), 931 (m), 869 (w), 841 (w), 811 (m), 784 (w), 714 (w), 689 (w), 669 (w), 650 (w), 556 (w), 530 (w) cm^{-1}. HRMS (ESI, pos. mode) *m/z*: [M + H$^+$] calcd. for $C_{15}H_{15}BrIO_3^+$ 448.9244; found 448.9243. $C_{15}H_{14}BrIO_3$ (449.08 g mol^{-1}).

6. Synthesis of β-Oxo Allyl Esters

6.1.1 Diallyl adipate (S5)

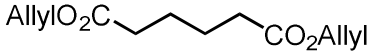

S5

Based on a literature procedure,[S6] adipic acid (30.0 g, 205 mmol), toluene (90 mL), allylic alcohol (35.7 g, 615 mmol) and *p*-toluenesulfonic acid monohydrate (1.95 g, 10.3 mmol) were placed in round-bottomed flask equipped with a Dean-Stark trap and a reflux condenser. The mixture was heated to reflux for 16 h. After cooling to ambient temperature, the organic solution was washed with saturated aqueous sodium bicarbonate (3 × 100 mL) and brine (3 × 50 mL), dried over $MgSO_4$, filtered and the solvent was removed under reduced pressure to furnish the ester **S5** (46.4 g, 205 mmol, 100%) as a yellow liquid. ^{1}H NMR (300 MHz, $CDCl_3$): δ = 5.91 (ddt, *J* = 17.4 Hz, *J* = 10.4 Hz, *J* = 5.7 Hz, 2 H), 5.31 (dq, *J* = 17.2 Hz, *J* = 1.6 Hz, 2 H), 5.23 (dq, *J* = 10.4 Hz, *J* = 1.3 Hz, 2 H), 4.57 (dt, *J* = 5.8 Hz, *J* = 1.4 Hz, 4 H), 2.40–2.30 (m, 4 H), 1.73–1.63 (m, 4 H) ppm. $C_{12}H_{18}O_4$ (226.27 g mol^{-1}).

The spectroscopic data are in accordance with the literature.[S7]

6.1.2 Allyl 1-(2-iodophenyl)-2-oxocyclopentane-1-carboxylate (S6)

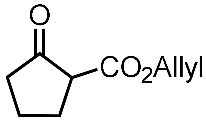

S6

Based on a literature procedure,[S8] NaH (60% in mineral oil, 10.7 g, 267 mmol) and anhydrous THF (120 mL) were added to a three-necked round-bottomed flask equipped with a reflux condenser under nitrogen atmosphere. The ester **S5** (46.4 g, 205 mmol) was dissolved in anhydrous THF (40 mL) and slowly added to the reaction vessel. The resulting mixture was stirred at 40°C for 16 h. After cooling down to ambient temperature the mixture was acidified to pH = 1 with hydrochloric acid (ca. 150 mL, 1 mol L^{-1}) and extracted with ethyl acetate (3 × 100 mL). The combined organic layers were washed with brine (2 × 100 mL), dried over $MgSO_4$, filtered and the solvent was evaporated. The residue was submitted to column chromatography (SiO_2, hexanes/MTBE 5:1, R_f = 0.25) to furnish the β-oxoester **S6** (29.7 g, 176 mmol, 86%) as a pink liquid. Mixture of keto/enol tautomers 95:5. ^{1}H NMR (300 MHz, $CDCl_3$): δ = 10.35 (s, 0.05 H, enol), 5.92 (ddt, *J* = 16.2 Hz, *J* = 10.7 Hz, *J* = 5.7 Hz, 1 H), 5.34 (dq, *J* = 17.2 Hz, *J* = 1.6 Hz, 1 H), 5.25 (dq, *J* = 10.4 Hz, *J* = 1.5 Hz, 1 H), 4.71–4.58 (m, 2 H), 3.19 (t, *J* = 9.0 Hz, 1 H), 2.58–2.48 (m, 0.2 H, enol), 2.40–2.25 (m, 4 H), 2.22–2.08 (m, 1 H), 1.95–1.79 (m, 1 H) ppm. $C_9H_{12}O_3$ (168.19 g mol^{-1}).

The spectroscopic data are in accordance with the literature.[S9]

6.2.1 Diallyl pimelate (S7)

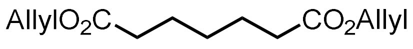

S7

Based on a literature procedure,[S6] pimelic acid (5.00 g, 31.2 mmol), toluene (15 mL), allylic alcohol (5.43 g, 93.6 mmol) and *p*-toluenesulfonic acid monohydrate (30.0 mg, 160 µmol) were placed in round-bottomed flask equipped with a Dean-Stark trap and a reflux condenser. The mixture was heated to reflux for 16 h. After cooling down to ambient temperature, the organic solution was washed with saturated aqueous sodium bicarbonate (3 × 15 mL) and brine (3 × 15 mL), dried over $MgSO_4$, filtered and the solvent was removed under reduced pressure to furnish the ester **S7** (7.19 g, 29.9 mmol, 96%) as a colorless oil. ^{1}H NMR (300 MHz, $CDCl_3$): δ = 5.92 (ddt, *J* = 17.1 Hz, *J* = 10.4 Hz, *J* = 5.7 Hz, 2 H), 5.31 (dq, *J* = 17.2 Hz, *J* = 1.5 Hz, 2 H), 5.23 (dq, *J* = 10.6 Hz, *J* = 1.2 Hz, 2 H), 4.57 (dt, *J* = 5.7 Hz, *J* = 1.2 Hz, 4 H), 2.34 (t, *J* = 7.5 Hz, 4 H), 1.66 (pent, *J* = 7.5 Hz, 4 H), 1.42–1.34 (m, 2 H) ppm. $C_{13}H_{20}O_4$ (240.30 g mol^{-1}).

The spectroscopic data are in accordance with the literature.[S6]

6.2.2 Allyl 2-oxocyclohexane-1-carboxylate (S8)

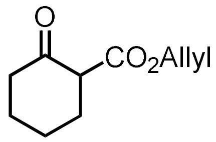

S8

Based on a literature procedure,[S8] NaH (60% in mineral oil, 780 mg, 32.5 mmol) and anhydrous THF (25 mL) were added to a three-necked round-bottomed flask equipped with a reflux condenser under nitrogen atmosphere. The ester **S7** (7.09 g, 29.5 mmol) was dissolved in anhydrous THF (10 mL) and slowly added to the reaction vessel. The resulting mixture was stirred at 40°C for 16 h. After cooling down to ambient temperature the mixture was acidified to pH = 1 with hydrochloric acid (ca. 40 mL, 1 mol L^{-1}) and extracted with ethyl acetate (3 × 25 mL). The combined organic layers were washed with brine (2 × 15 mL), dried over $MgSO_4$, filtered and the solvent was evaporated. The residue was submitted to column chromatography (SiO_2, hexanes/MTBE 8:1, R_f = 0.40) to furnish the β-oxoester **S8** (4.96 g, 27.2 mmol, 92%) in its enol form as a yellow oil. ^{1}H NMR (300 MHz, $CDCl_3$): δ = 12.15 (s, 1 H), 5.95 (ddt, *J* = 17.1 Hz, *J* = 10.7 Hz, *J* = 5.5 Hz, 1 H), 5.33 (dq, *J* = 17.2 Hz, *J* = 1.5 Hz, 1 H), 5.24 (dq, *J* = 10.4 Hz, *J* = 1.3 Hz, 1 H), 4.66 (dt, *J* = 5.4 Hz, *J* = 1.4 Hz, 2 H), 2.34–2.17 (m, 4 H), 1.78–1.45 (m, 4 H) ppm. $C_{10}H_{14}O_3$ (182.22 g mol^{-1}).

The spectroscopic data are in accordance with the literature.[S10]

6.3.1 2-Oxocycloheptane-1-carboxylic acid (S9)

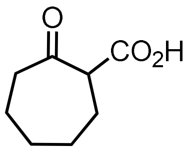

S9

Based on a literature procedure,[S1] an aqueous NaOH solution (14.4 mL, 2 mol L^{-1}) was added to a solution of methyl 2-oxocycloheptane-1-carboxylate (500 mg, 2.94 mmol) in MeOH (1.5 mL) and the resulting mixture was stirred at ambient temperature for 16 h. Subsequently, while cooling with an ice bath, the mixture was acidified to pH = 1 with hydrochloric acid (ca. 35 mL, 1 mol L^{-1}) and extracted with CH_2Cl_2 (3 × 50 mL). The combined organic layers were dried over $MgSO_4$, filtered and the solvents were evaporated at ambient temperature to minimize decarboxylation. The carboxylic acid **S9** (0.415 g, 2.66 mmol, 90%) was furnished as a light-yellow oil with traces of the decarboxylation product in the ^{1}H NMR spectrum. Mixture of keto/enol tautomers 3:1. ^{1}H NMR (300 MHz, $CDCl_3$): δ = 12.40 (s, 0.33 H, enol), 10.39 (br s, 1.33 H), 3.58 (dd, *J* = 9.9 Hz, *J* = 3.8 Hz, 1 H, keto), 2.70–2.59 (m, 2 H), 2.52–2.39 (m, 2.33 H), 2.23–2.12 (m, 1 H), 1.94–1.82 (m, 3 H), 1.74–1.62 (m, 4 H), 1.54–1.42 (m, 2 H) ppm. $C_8H_{12}O_3$ (156.18 g mol^{-1}).

The spectroscopic data are in accordance with the literature.[S11]

It is advised to immediately use the product if handled at ambient temperatures, since decarboxylation takes place (fully decarboxylated after storing at 5°C for approximately seven days).

6.3.2 Allyl 2-oxocycloheptane-1-carboxylate (S10)

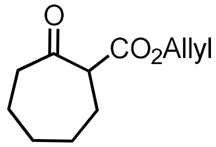

S10

Based on a literature procedure,[S12] *N*,*N'*-dicyclohexylcarbodiimide (7.69 g, 37.3 mmol) and 4-dimethylaminopyridine (455 mg, 3.73 mmol) were added to a solution of carboxylic acid **S9** (4.48 g, 28.7 mmol) and allylic alcohol (3.33 g, 57.4 mmol) in CH_2Cl_2 (60 mL) and the resulting mixture was stirred at ambient temperature for 16 h. Subsequently, the mixture was filtered over filter paper (cellulose, type 600P) and the filtrate was evaporated. The residue was submitted to column chromatography (SiO_2, hexanes/MTBE 20:1, R_f = 0.10) to furnish the β-oxo-ester **S10** (3.00 g, 15.3 mmol, 53%) as a colorless oil. Mixture of keto/enol tautomers 10:1. ^{1}H NMR (300 MHz, $CDCl_3$): δ = 12.66 (s, 0.1 H; enol), 5.90 (ddt, *J* = 16.4 Hz, *J* = 10.9 Hz, *J* = 5.8 Hz, 1.1 H), 5.32 (dq, *J* = 17.2 Hz, *J* = 1.6 Hz, 1.1 H), 5.24 (dq, *J* = 10.4 Hz, *J* = 1.5 Hz, 1.1 H), 4.68–4.57 (m, 2.2 H), 3.58 (dd, *J* = 10.4 Hz, *J* = 3.9 Hz, 1 H; keto), 2.64–2.40 (m, 2.2 H), 2.16–

2.05 (m, 1 H), 1.99–1.79 (m, 3.4 H), 1.74–1.55 (m, 2.2 H), 1.53–1.37 (m, 2.2 H) ppm. $C_{11}H_{16}O_3$ (196.25 g mol^{-1}).

The spectroscopic data are in accordance with the literature.[S13]

6.4.1 Methyl 1-oxoindane-2-carboxylate (S11)

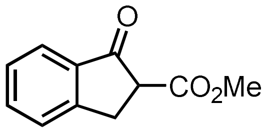

S11

Based on a literature procedure,[S14] NaH (60% in mineral oil, 4.23 g, 106 mmol) and anhydrous THF (100 mL) were added to a three-necked round-bottomed flask equipped with a reflux condenser and a dropping funnel under nitrogen atmosphere. Dimethyl carbonate (6.81 g, 75.6 mmol) was added and the mixture was heated to reflux. A solution of 1-indanone (5.00 g, 37.8 mmol) in anhydrous THF (40 mL) was added to the vigorously stirred reaction mixture via dropping funnel over the course of approx. 20 min. After complete addition the resulting mixture was heated to reflux for additional 2 h and subsequently stirred at ambient temperature for 16 h. The mixture was then acidified to pH = 1 with hydrochloric acid (ca. 100 mL, 1 mol L^{-1}) and extracted with MTBE (3 × 50 mL). The combined organic layers were dried over $MgSO_4$, filtered and the solvent was removed under reduced pressure. The residue was submitted to column chromatography (SiO_2, hexanes/MTBE 4:1, R_f = 0.34) to furnish the ester **S11** (6.65 g, 35.0 mmol, 93%) as a red liquid. Mixture of keto/enol tautomers 6.5:1. ^{1}H NMR (300 MHz, $CDCl_3$): δ = 10.37 (s, 0.15 H; enol), 7.78 (d, J = 7.7 Hz, 1 H; keto), 7.68–7.60 (m, 1.15 H), 7.51 (d, J = 7.7 Hz, 1 H; keto), 7.48–7.45 (m, 0.15 H; enol), 7.44–7.37 (m, 1.3 H), 3.86 (s, 0.45 H; enol), 3.80 (s, 3 H; keto), 3.75 (dd, J = 8.3 Hz, J = 4.1 Hz, 1 H; keto), 3.62–3.59 (m, 0.3 H; enol), 3.56–3.52 (m, 1 H; keto), 3.38 (dd, J = 17.3 Hz, J = 8.3 Hz, 1 H; keto) ppm. $C_{11}H_{10}O_3$ (190.20 g mol^{-1}).

The spectroscopic data are in accordance with the literature.[S14]

6.4.2 Allyl 1-oxoindane-2-carboxylate (S12)

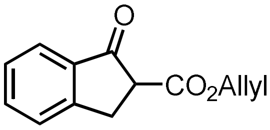

S12

Based on a literature procedure,[S15] ester **S11** (2.12 g, 11.2 mmol), allylic alcohol (713 mg, 12.3 mmol), 4-dimethylaminopyridine (68 mg, 56 µmol) and cyclohexane (11 mL) were placed in round-bottomed flask equipped with a Dean-Stark trap and a reflux condenser. The mixture was heated to reflux for 16 h. Subsequently, all volatiles were removed under reduced pressure

and the residue was submitted to column chromatography (SiO_2, hexanes/MTBE 3:1, R_f = 0.32) to furnish the β-oxoester **S12** (1.74 g, 8.07 mmol, 72%) as a red oil. Mixture of keto/enol tautomers 5:1. ^{1}H NMR (300 MHz, $CDCl_3$): δ = 10.35 (br s, 0.2 H; enol), 7.78 (d, *J* = 7.7 Hz, 1 H; keto), 7.68–7.60 (m, 1.2 H), 7.53–7.48 (m, 1 H; keto), 7.48–7.45 (m, 0.2 H; enol), 7.45–7.36 (m, 1.4 H), 5.95 (ddt, *J* = 17.3 Hz, *J* = 10.5 Hz, *J* = 5.6 Hz, 1.2 H), 5.37 (dq, *J* = 17.2 Hz, *J* = 1.5 Hz, 1.2 H), 5.26 (dq, *J* = 10.3 Hz, *J* = 1.3 Hz, 1.2 H), 4.80–4.75 (m, 0.4 H; enol), 4.74–4.63 (m, 2 H; keto), 3.76 (dd, *J* = 8.3 Hz, *J* = 4.1 Hz, 1 H; keto), 3.61 (d, *J* = 4.1 Hz, 0.4 H; enol), 3.55 (d, *J* = 4.3 Hz, 1 H; keto), 3.39 (dd, *J* = 17.2 Hz, *J* = 8.3 Hz, 1 H; keto) ppm. $C_{13}H_{12}O_3$ (216.24 g mol^{-1}).

The spectroscopic data are in accordance with the literature.[S14]

6.5.1 Methyl 1-oxo-1,2,3,4-tetrahydronaphthalene-2-carboxylate (S13)

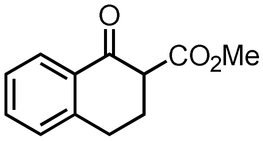

S13

Based on a literature procedure,[S14] NaH (60% in mineral oil, 7.66 g, 192 mmol) and anhydrous THF (100 mL) were added to a three-necked round-bottomed flask equipped with a reflux condenser and a dropping funnel under nitrogen atmosphere. Dimethyl carbonate (12.3 g, 137 mmol) was added and the mixture was heated to reflux. A solution of 1-tetralone (10.0 g, 68.4 mmol) in anhydrous THF (50 mL) was added to the vigorously stirred reaction mixture via dropping funnel over the course of approx. 20 min. After complete addition, the resulting mixture was heated to reflux for additional 2 h and subsequently stirred at ambient temperature for 16 h. The mixture was then acidified to pH = 1 with hydrochloric acid (ca. 200 mL, 1 mol L^{-1}) and extracted with MTBE (3 × 100 mL). The combined organic layers were dried over $MgSO_4$, filtered and the solvent was removed under reduced pressure. The residue was submitted to column chromatography (SiO_2, hexanes/MTBE 10:1, R_f = 0.32) to furnish the ester **S13** (13.16 g, 64.44 mmol, 94%) as a yellow oil. Mixture of keto/enol tautomers 1.2:1. ^{1}H NMR (300 MHz, $CDCl_3$): δ = 12.41 (s, 1 H; enol), 8.05 (dd, *J* = 7.9 Hz, *J* = 1.5 Hz, 1.2 H; keto), 7.80 (dd, *J* = 7.3 Hz, *J* = 1.9 Hz, 1 H; enol), 7.50 (td, *J* = 7.5 Hz, *J* = 1.5 Hz, 1.2 H; keto), 7.36–7.28 (m, 3.2 H), 7.27–7.24 (m, 1.2 H; keto), 7.17 (dd, *J* = 7.2 Hz, *J* = 1.5 Hz, 1 H; enol), 3.83 (s, 3 H; enol), 3.79 (s, 3.6 H; keto), 3.63 (dd, *J* = 10.3 Hz, *J* = 4.8 Hz, 1.2 H; keto), 3.13–2.94 (m, 2.4 H; keto), 2.81 (dd, *J* = 8.9 Hz, *J* = 6.6 Hz, 2 H; enol), 2.60–2.54 (m, 2 H; enol), 2.49 (ddd, *J* = 10.3 Hz, *J* = 8.9 Hz, *J* = 5.2 Hz, 1.2 H; keto), 2.42–2.31 (m, 1.2 H; keto) ppm. $C_{12}H_{12}O_3$ (204.23 g mol^{-1}).

The spectroscopic data are in accordance with the literature.[S14]

6.5.2 1-Oxo-1,2,3,4-tetrahydronaphthalene-2-carboxylic acid (S14)

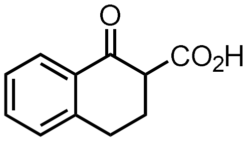

S14

Based on a literature procedure,[S1] an aqueous NaOH solution (140 mL, 2 mol L^{-1}) was added to a solution of ester **S13** (6.00 g, 29.4 mmol) in MeOH (14 mL) and the resulting mixture was stirred at ambient temperature for 16 h. Subsequently, while cooling with an ice bath the mixture was acidified to pH = 1 with hydrochloric acid (ca. 300 mL, 1 mol L^{-1}) and extracted with CH_2Cl_2 (3 × 150 mL). The combined organic layers were dried over $MgSO_4$, filtered and the solvents were evaporated at ambient temperature to minimize decarboxylation. The carboxylic acid **S14** (5.36 g, 28.2 mmol, 96%) was furnished as a yellow solid with traces of the decarboxylation product in the ^{1}H NMR spectrum and was directly used in the next step. Mixture of keto/enol tautomers 1:2. ^{1}H NMR (300 MHz, $CDCl_3$): δ = 12.17 (s, 1 H; enol) 11.38 (br s, 1.5 H), 8.11 (dd, *J* = 8.0 Hz, *J* = 1.4 Hz, 0.5 H; keto), 7.83 (dd, *J* = 7.5 Hz, *J* = 1.6 Hz, 1 H; enol), 7.56 (td, *J* = 7.5 Hz, *J* = 1.5 Hz, 0.5 H; keto), 7.40–7.25 (m, 3 H), 7.20 (dd, *J* = 7.4 Hz, *J* = 1.4 Hz, 1 H; enol), 3.57 (dd, *J* = 11.7 Hz, *J* = 4.8 Hz, 0.5 H; keto), 3.12–3.04 (m, 1 H; keto), 2.85 (dd, *J* = 9.0 Hz, *J* = 6.5 Hz, 2 H; enol), 2.69–2.59 (m, 2.5 H), 2.39 (dddd, *J* = 13.7 Hz, *J* = 11.8 Hz, *J* = 9.9 Hz, *J* = 5.7 Hz, 0.5 H; keto) ppm. $C_{11}H_{10}O_3$ (190.20 g mol^{-1}).

The spectroscopic data are in accordance with the literature.[S16]

6.5.3 Allyl 1-oxo-1,2,3,4-tetrahydronaphthalene-2-carboxylate (S15)

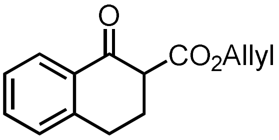

S15

Based on a literature procedure,[S12] *N*,*N'*-dicyclohexylcarbodiimide (7.51 g, 36.4 mmol) and 4-dimethylaminopyridine (445 mg, 3.64 mmol) were added to a solution of carboxylic acid **S14** (5.33 g, 28.0 mmol) and allylic alcohol (3.25 g, 56.0 mmol) in CH_2Cl_2 (60 mL) and the resulting mixture was stirred at ambient temperature for 16 h. Subsequently, the mixture was filtered over filter paper (cellulose, type 600P) and the filtrate was evaporated. The residue was submitted to column chromatography (SiO_2, hexanes/MTBE 40:1, R_f = 0.12) to furnish the β-oxo-ester **S15** (5.36 g, 23.3 mmol, 83%) as a colorless oil. Mixture of keto/enol tautomers 1:1. ^{1}H NMR (300 MHz, $CDCl_3$): δ = 12.40 (s, 1 H; enol), 8.05 (dd, *J* = 7.9 Hz, *J* = 1.4 Hz, 1 H; keto), 7.80 (dd, *J* = 7.3 Hz, *J* = 1.8 Hz, 1 H; enol), 7.50 (td, *J* = 7.5 Hz, *J* = 1.5 Hz, 1 H; keto), 7.36–7.23 (m, 4 H), 7.18 (dt, *J* = 6.9 Hz, *J* = 1.3 Hz, 1 H; enol), 6.07–5.87 (m, 2 H), 5.42–5.21 (m, 4 H), 4.76–4.63 (m, 4 H), 3.65 (dd, *J* = 10.5 Hz, *J* = 4.8 Hz, 1 H; keto), 3.13–2.95 (m, 2 H; keto),

2.82 (dd, *J* = 8.9 Hz, *J* = 6.5 Hz, 2 H; enol), 2.64–2.58 (m, 2 H; enol), 2.52 (dtd, *J* = 10.4 Hz, *J* = 7.1 Hz, *J* = 4.5 Hz, 1 H; keto), 2.38 (ddd, *J* = 13.5 Hz, *J* = 10.3 Hz, *J* = 4.9 Hz, 1 H; keto) ppm. $C_{14}H_{14}O_3$ (230.26 g mol^{-1}).

The spectroscopic data are in accordance with the literature.[S14]

7. Crystal Structure of Compound 17a

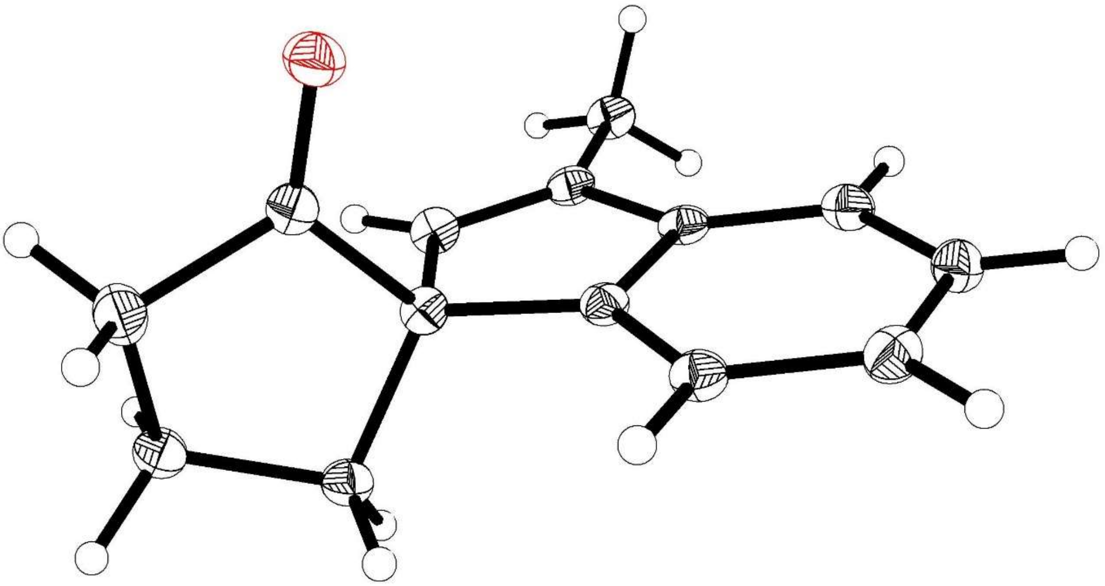

Crystals of compound **17a** suitable for X-ray analysis were obtained within one day from a saturated solution in CH_2Cl_2-hexanes at 25°C. Single crystal X-ray data for **17a** were measured on a Bruker AXS D8 Venture diffractometer (multilayer optics, Cu-Kα radiation with λ = 1.54178 Å, Kappa 4-circle goniometer, Photon III C14 CPAD detector) at a temperature of 100 K. An absorption correction using equivalent reflections was performed with the program SADABS.[S17] The structure was solved with the program SHELXS[S18] and refined with SHELXL[S19] using the OLEX2[S20] GUI. All non-H atoms were refined using anisotropic atomic displacement parameters. H atoms bonded to C were located in the difference Fourier maps and placed on idealized geometric positions with idealized atomic displacement parameters using the riding model. The absolute structure was determined by anomalous dispersion effects using Cu-Kα radiation. The crystallographic data can be obtained free of charge from https://www.ccdc.cam.ac.uk/structures/ quoting the CCDC number 2380538.

Table S1. Crystal data and structure refinement for compound **17a**.

Empirical formula	$C_{14}H_{14}O$	
Formula weight	198.25 g mol^{-1}	
Temperature	100(2) K	
Wavelength	1.54178 Å	
Crystal system	orthorhombic	
Space group	$P2_12_12_1$	
Unit cell dimensions	a = 7.1979(3) Å	α = 90°.
	b = 9.5011(5) Å	β = 90°.
	c = 15.3268(7) Å	γ = 90°.
Volume	1048.17(9) Å^3	
Z	4	
Density (calculated)	1.256 Mg/m^3	
Absorption coefficient	0.600 mm^{-1}	
F(000)	424	
Crystal size	0.120 x 0.080 x 0.040 mm^3	
Theta range for data collection	5.478 to 74.368°	
Index ranges	$-8 \le h \le 8$, $-11 \le k \le 11$, $-19 \le l \le 19$	
Reflections collected	20767	
Independent reflections	2131 [R(int) = 0.0206]	
Observed reflections [I > 2(I)]	2112	
Completeness to theta = 74.368°	100.0%	
Absorption correction	Semi-empirical from equivalents	
Max. and min. transmission	1.0000 and 0.9236	
Refinement method	Full-matrix least-squares on F^2	
Data / restraints / parameters	2131 / 0 / 137	
Goodness-of-fit on F^2	1.058	
Final R indices [I>2sigma(I)]	R1 = 0.0293, wR2 = 0.0844	
R indices (all data)	R1 = 0.0295, wR2 = 0.0848	
Absolute structure parameter	–0.05(3)	
Extinction coefficient	n/a	
Largest diff. peak and hole	0.306 and –0.133 e^- x Å^{-3}	

8. References

(S1) Fliegel, L.; Christoffers, J. *Org. Lett.* **2022**, *24*, 8526–8530.

(S2) Tsuda, T.; Chujo, Y.; Nishi, S.; Tawara, K.; Saegusa, T. *J. Am. Chem. Soc.* **1980**, *102*, 6381–6384.

(S3) Akula, R.; Doran, R.; Guiry, P. J. *Chem. Eur. J.* **2016**, *22*, 9938–9942.

(S4) Penning, M.; Aeissen, E.; Christoffers, J. *Synthesis* **2015**, *47*, 1007–1015.

(S5) a) Jia, Z.; Galvez, E.; Sebastian, R. M.; Pleixats, R.; Alvarez-Larena, A.; Martin, E.; Vallribera, A.; Shafir, A. *Angew. Chem. Int. Ed.* **2014**, *53*, 11298–11301; b) Wu, Y.; Arenas, I.; Broomfield, L. M.; Martin, E.; Shafir, A. *Chem. Eur. J.* **2015**, *21*, 18779–18784.

(S6) Mohr, J. T.; Krout, M. R.; Stoltz, B. M. *Org. Synth.* **2009**, *86*, 194–211.

(S7) Dagoneau, D.; Xu, Z.; Wang, Q.; Zhu, J. *Angew. Chem. Int. Ed.* **2016**, *55*, 760–763.

(S8) Doran, R.; Guiry, P. J. *J. Org. Chem.* **2014**, *79*, 9112–9124.

(S9) Back, T. G.; Gladstone, P. L.; Parvez, M. *J. Org. Chem.* **1996**, *61*, 3806–3814.

(S10) Boddaert, T.; Coquerel, Y.; Rodriguez, J. *Eur. J. Org. Chem.* **2011**, 5061–5070.

(S11) Senboku, H.; Fujimura, Y.; Kamekawa, H.; Tokuda, M. *Electrochim. Acta* **2000**, *45*, 2995–3003.

(S12) Ding, L.; Song, H.; Zheng, C.; You, S.-L. *J. Am. Chem. Soc.* **2022**, *144*, 4770–4775.

(S13) Boudreault, J.; Lévesque, F.; Bélanger, G. *J. Org. Chem.* **2016**, *81*, 9247–9268.

(S14) Ding, T.; Jiang, L.; Xu, Y.; Wang, G.; Yi, W. *Org. Lett.* **2019**, *21*, 6025–6028.

(S15) Christoffers, J.; Önal, N. *Eur. J. Org. Chem.* **2000**, 1633–1635.

(S16) Shibatomi, K.; Kitahara, K.; Sasaki, N.; Kawasaki, Y.; Fujisawa, I.; Iwasa, S. *Nat. Commun.* **2017**, *8*, 15600.

(S17) Krause, L.; Herbst-Irmer, R.; Sheldrick, G. M.; Stalke, D. *J. Appl. Cryst.* **2015**, *48*, 3–10.

(S18) Sheldrick, G. M. *Acta Cryst.* **2008**, *A64*, 112–122.

(S19) Sheldrick, G. M. *Acta Cryst.* **2015**, *C71*, 3–8.

(S20) Dolomanov, O. V.; Bourhis, L. J.; Gildea, R. J.; Howard, J. A. K.; Puschmann, H. *J. Appl. Cryst.* **2009**, *42*, 339–341.

9. NMR-Spectra of all Reported Compounds

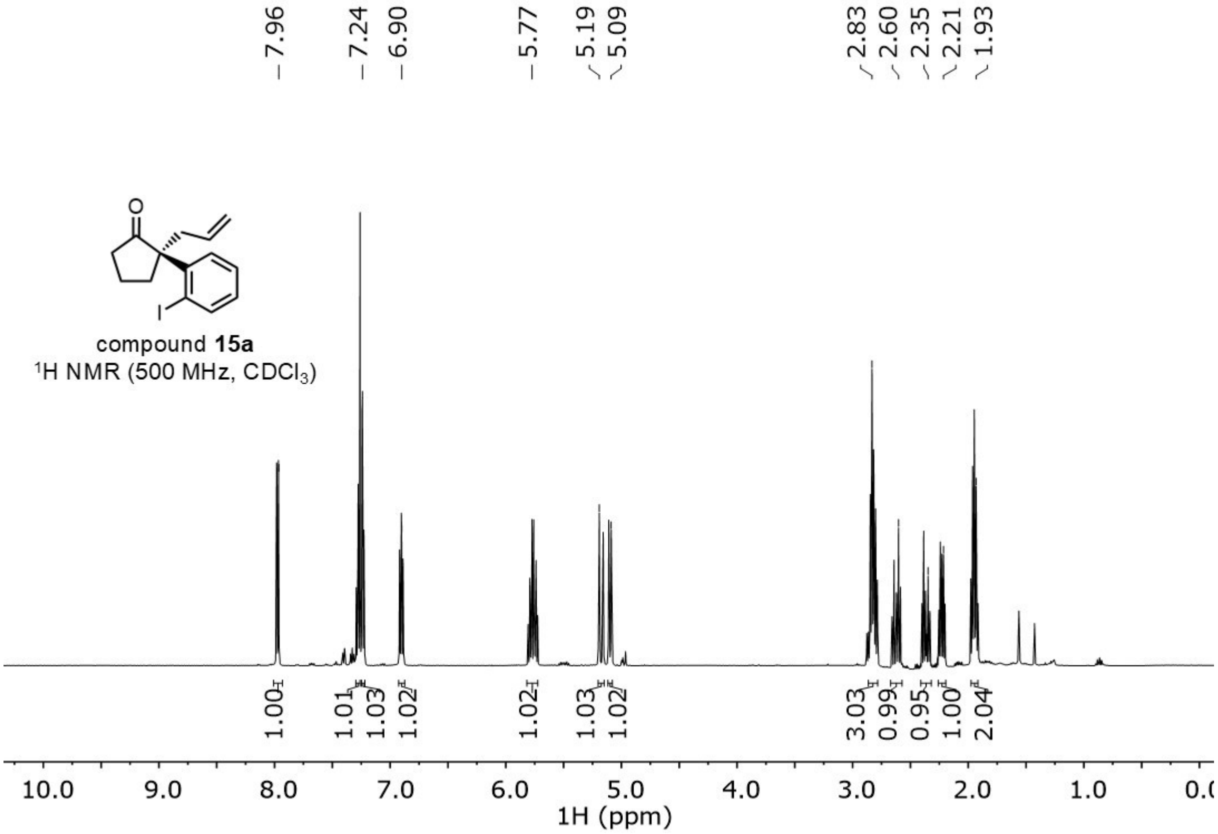

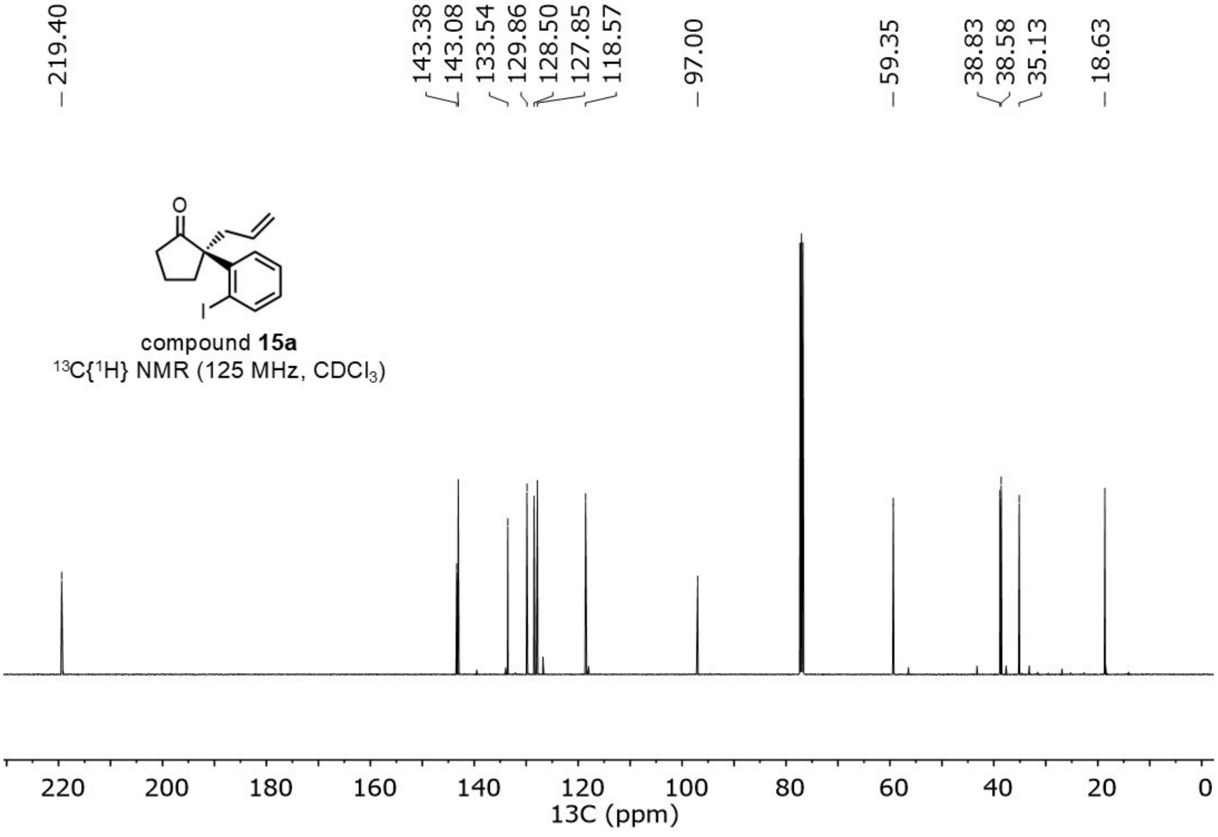

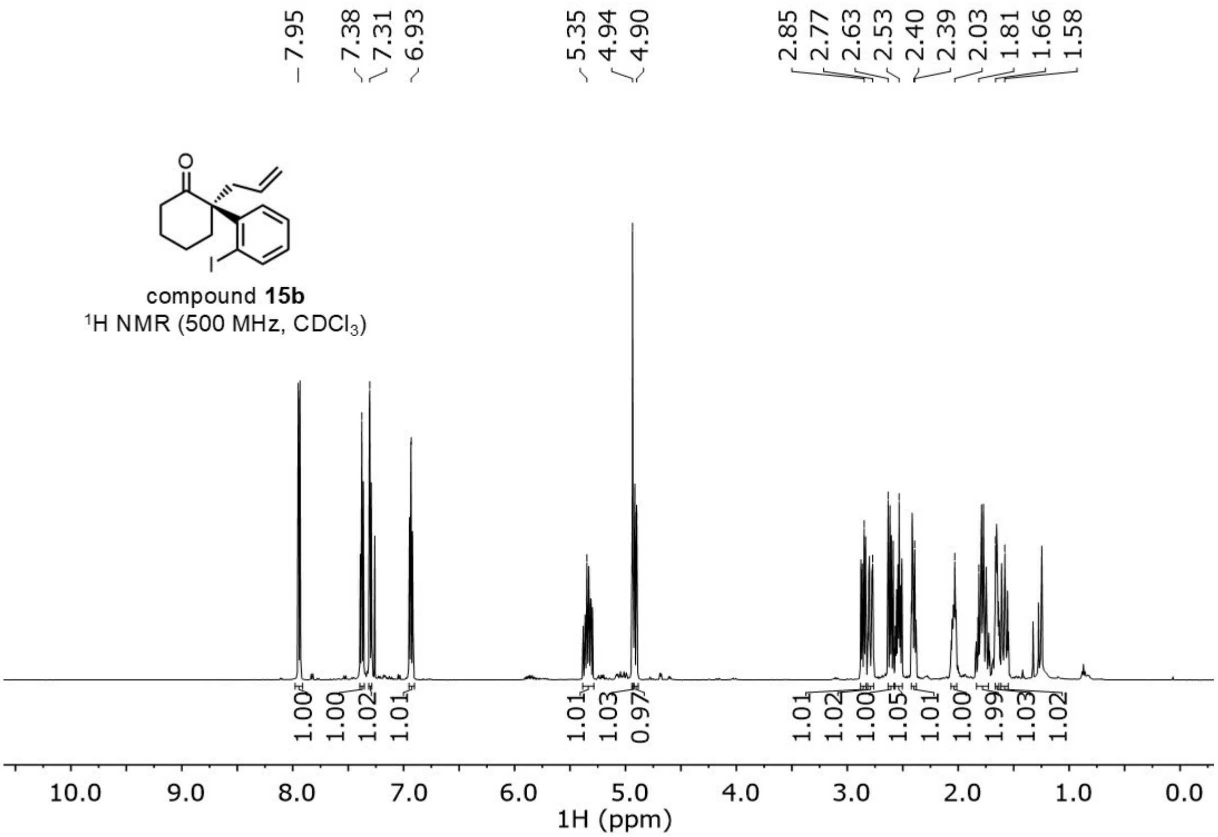
7.95
7.38
7.31
6.93
5.35
4.94
4.90
2.85
2.77
2.63
2.53
2.40
2.39
2.03
1.81
1.66
1.58
O
I
compound 15b
1H NMR (500 MHz, CDCl3)
1.00
1.00
1.02
1.01
1.01
1.03
0.97
1.01
1.02
1.00
1.05
1.01
1.00
1.99
1.03
1.02
10.0
9.0
8.0
7.0
6.0
5.0
4.0
3.0
2.0
1.0
0.0
1H (ppm)

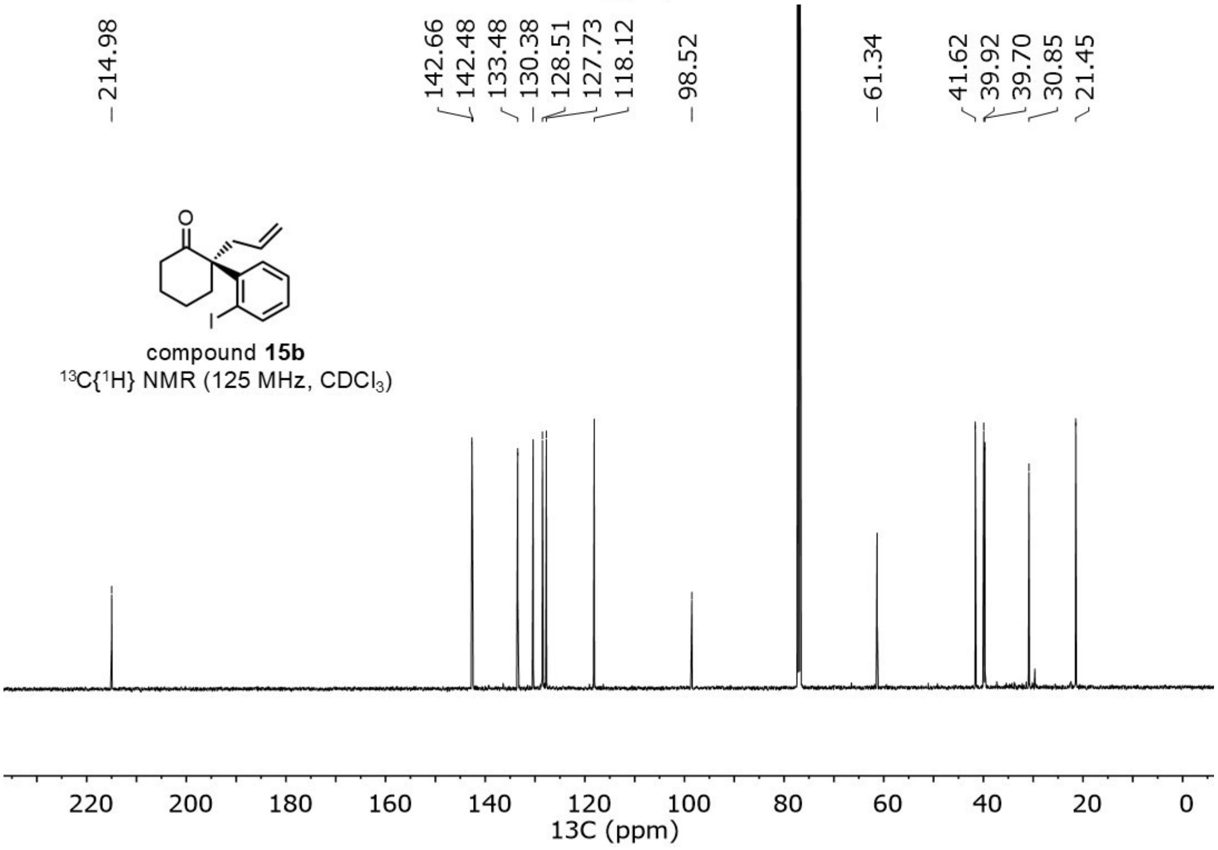
214.98
142.66
142.48
133.48
130.38
128.51
127.73
118.12
98.52
61.34
41.62
39.92
39.70
30.85
21.45
O
I
compound 15b
13C{1H} NMR (125 MHz, CDCl3)
220
200
180
160
140
120
100
80
60
40
20
0
13C (ppm)

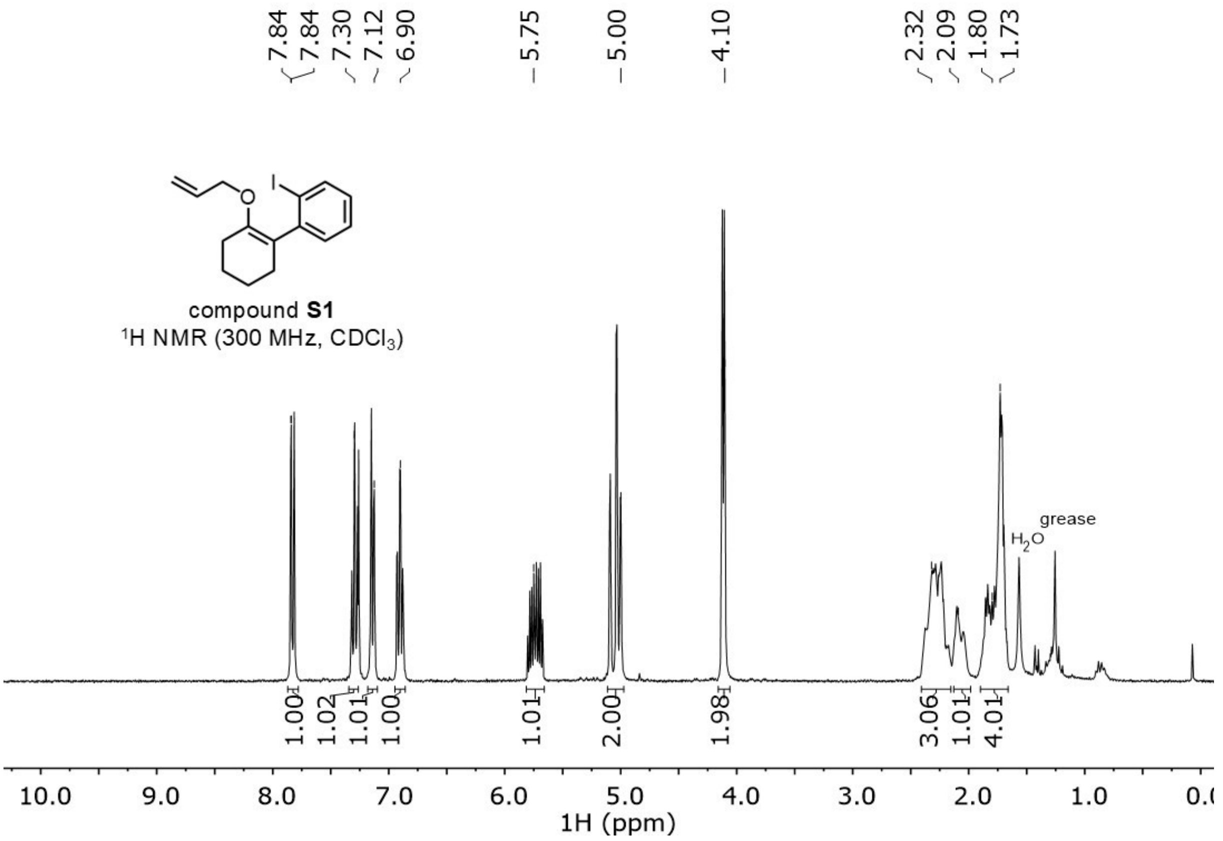

7.84
7.84
7.30
7.12
6.90
5.75
5.00
4.10
2.32
2.09
1.80
1.73
compound S1
1H NMR (300 MHz, CDCl3)
grease
H2O
1.00
1.02
1.01
1.00
1.01
2.00
1.98
3.06
1.01
4.01
10.0
9.0
8.0
7.0
6.0
5.0
4.0
3.0
2.0
1.0
1H (ppm)

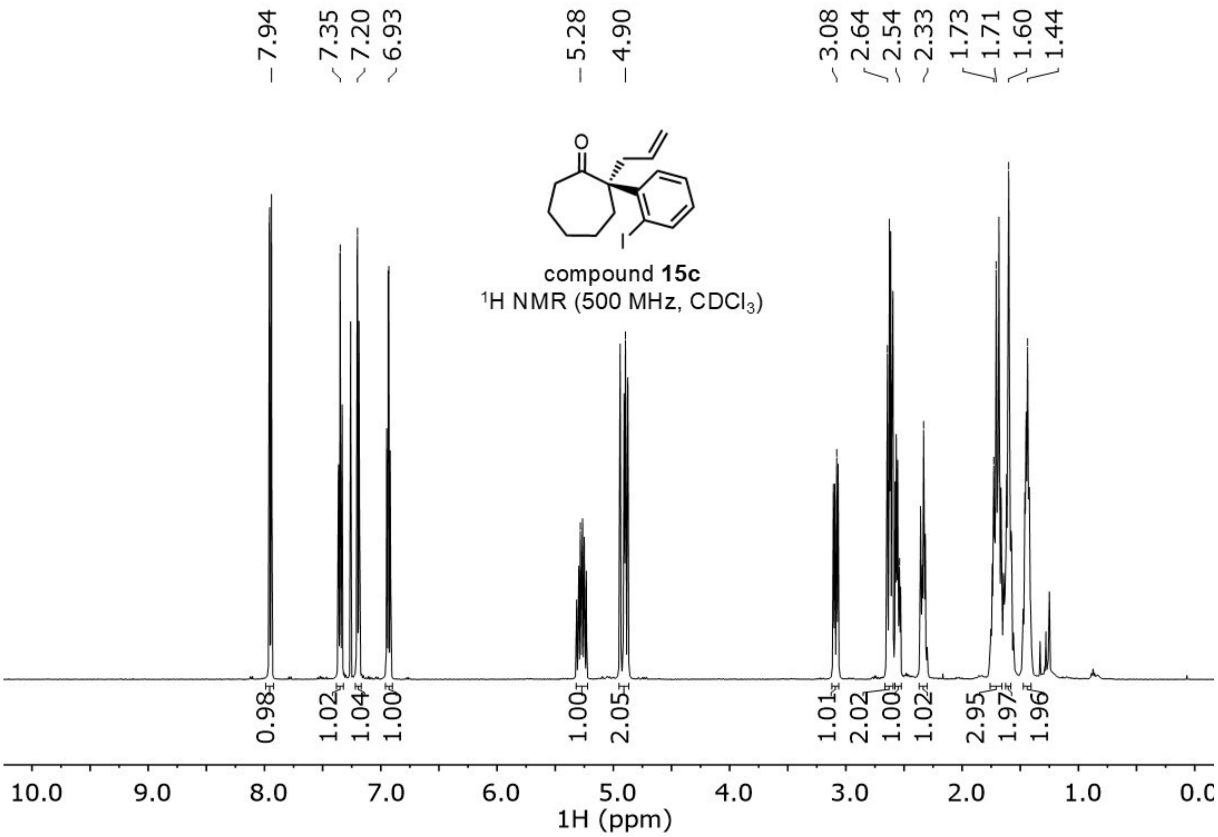
7.94
7.35
7.20
6.93
5.28
4.90
3.08
2.64
2.54
2.33
1.73
1.71
1.60
1.44
O
I
compound 15c
^{1}H NMR (500 MHz, $CDCl_3$)
0.98
1.02
1.04
1.00
1.00
2.05
1.01
2.02
1.00
1.02
2.95
1.97
1.96
10.0 9.0 8.0 7.0 6.0 5.0 4.0 3.0 2.0 1.0 0.0
1H (ppm)

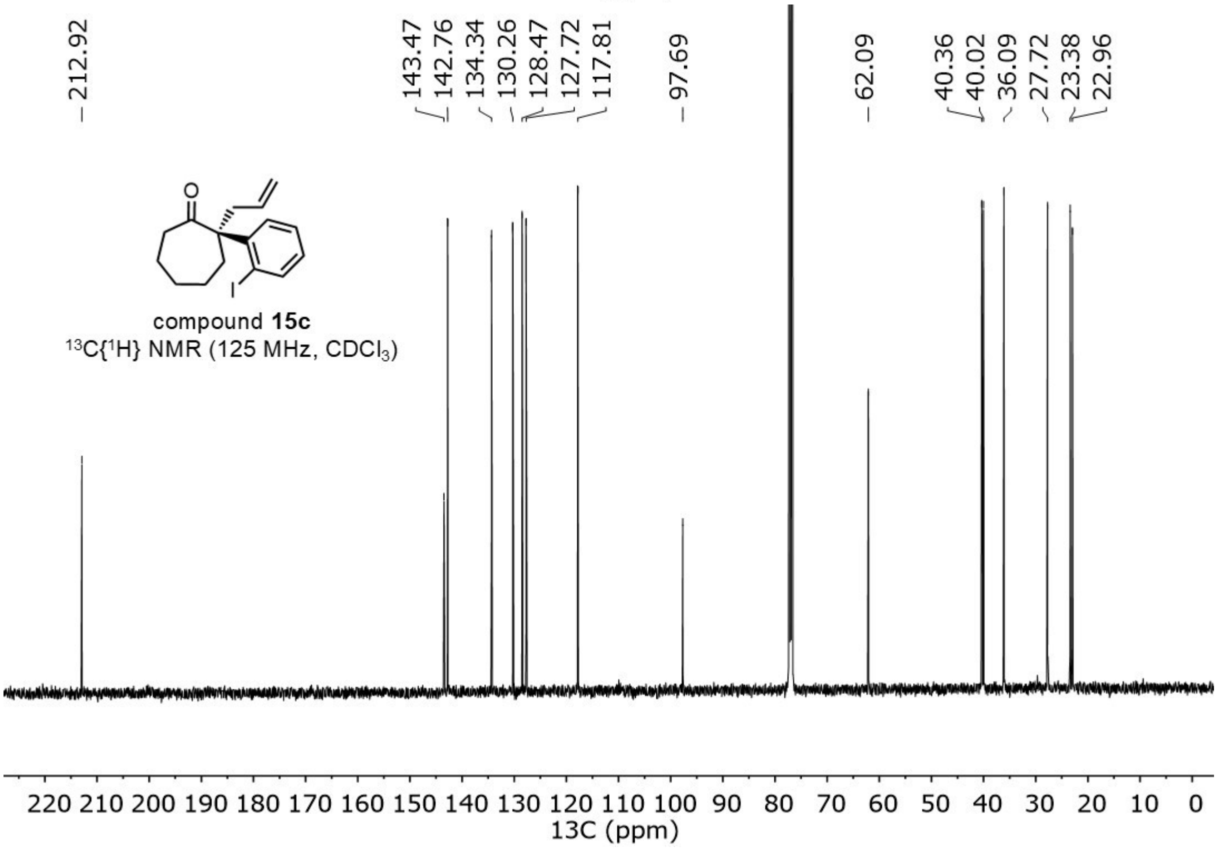
212.92
143.47
142.76
134.34
130.26
128.47
127.72
117.81
97.69
62.09
40.36
40.02
36.09
27.72
23.38
22.96
O
I
compound 15c
^{13}C{^{1}H} NMR (125 MHz, $CDCl_3$)
220 210 200 190 180 170 160 150 140 130 120 110 100 90 80 70 60 50 40 30 20 10 0
13C (ppm)

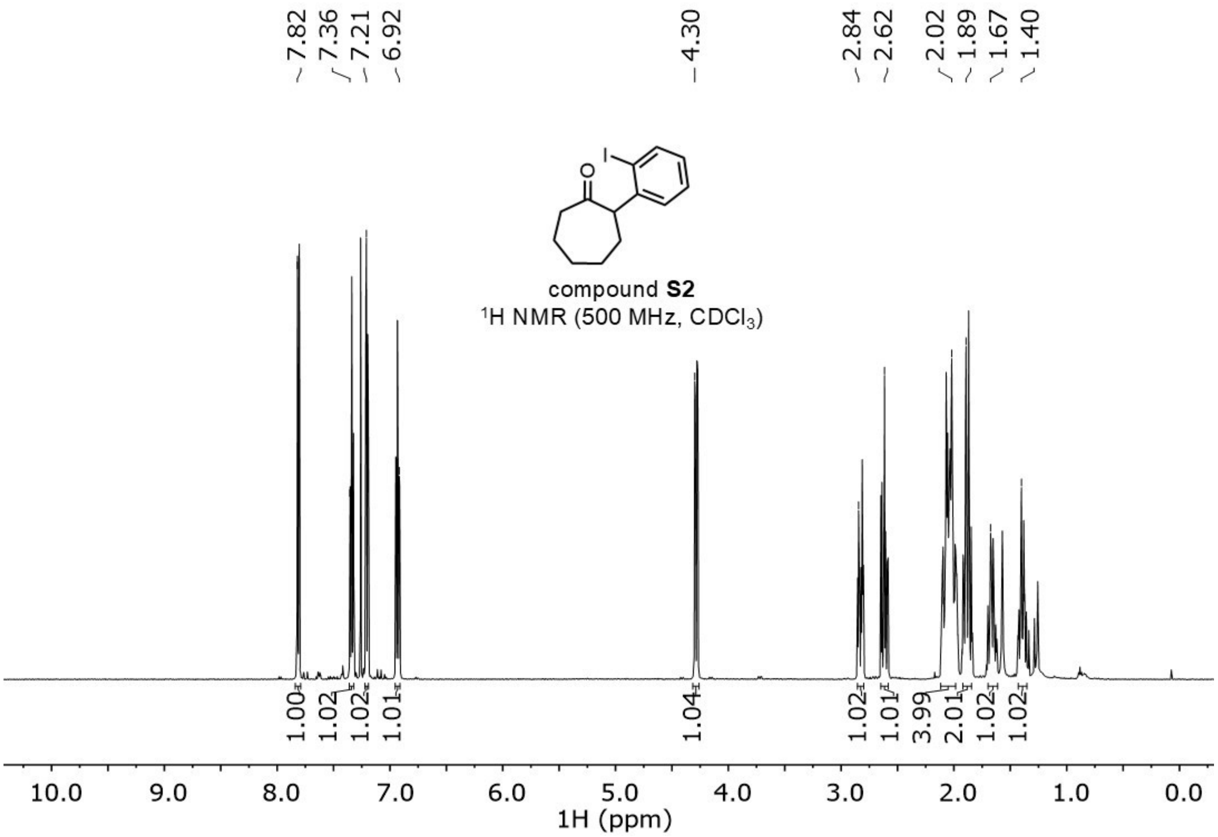
7.82
7.36
7.21
6.92
4.30
2.84
2.62
2.02
1.89
1.67
1.40
compound **S2**
^{1}H NMR (500 MHz, $CDCl_3$)
1.00
1.02
1.02
1.01
1.04
1.02
1.01
3.99
2.01
1.02
1.02
10.0
9.0
8.0
7.0
6.0
5.0
4.0
3.0
2.0
1.0
0.0
1H (ppm)

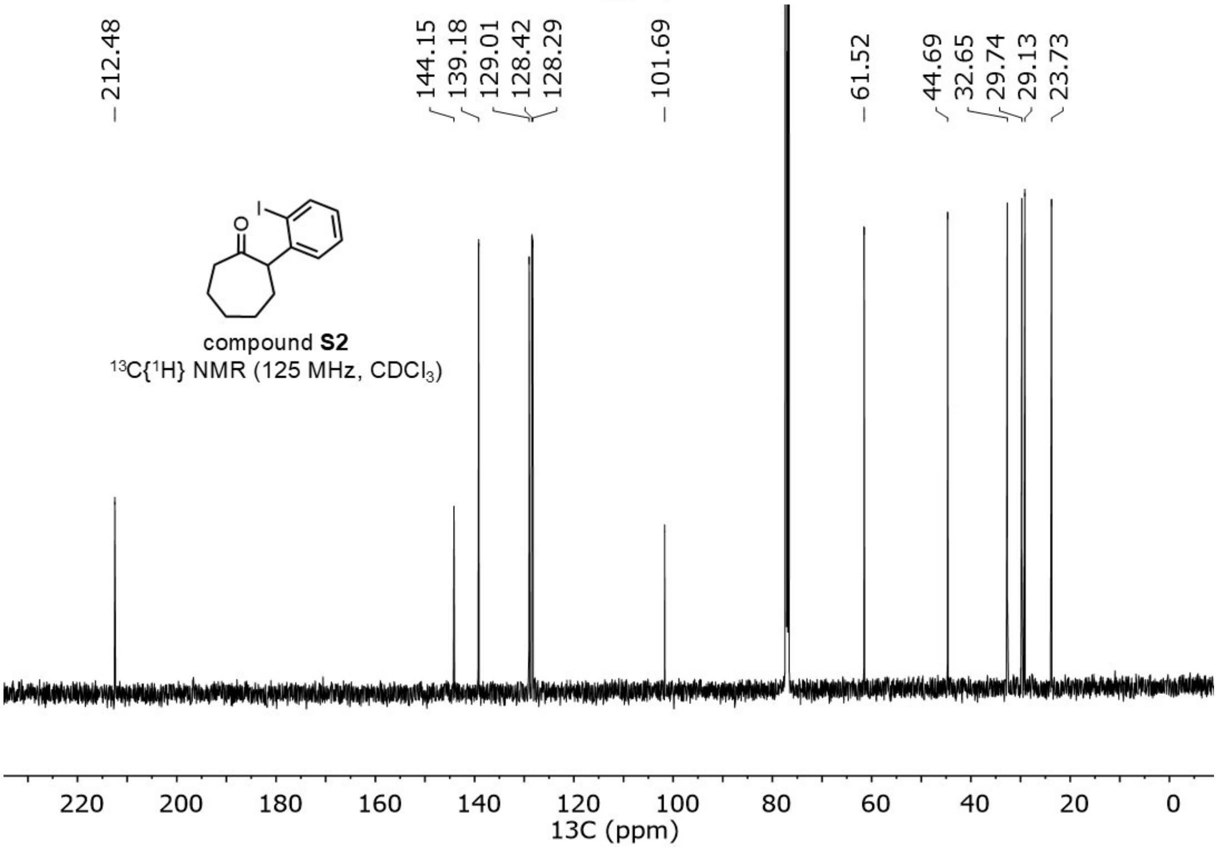
212.48
144.15
139.18
129.01
128.42
128.29
101.69
61.52
44.69
32.65
29.74
29.13
23.73
compound **S2**
^{13}C{^{1}H} NMR (125 MHz, $CDCl_3$)
220
200
180
160
140
120
100
80
60
40
20
0
13C (ppm)

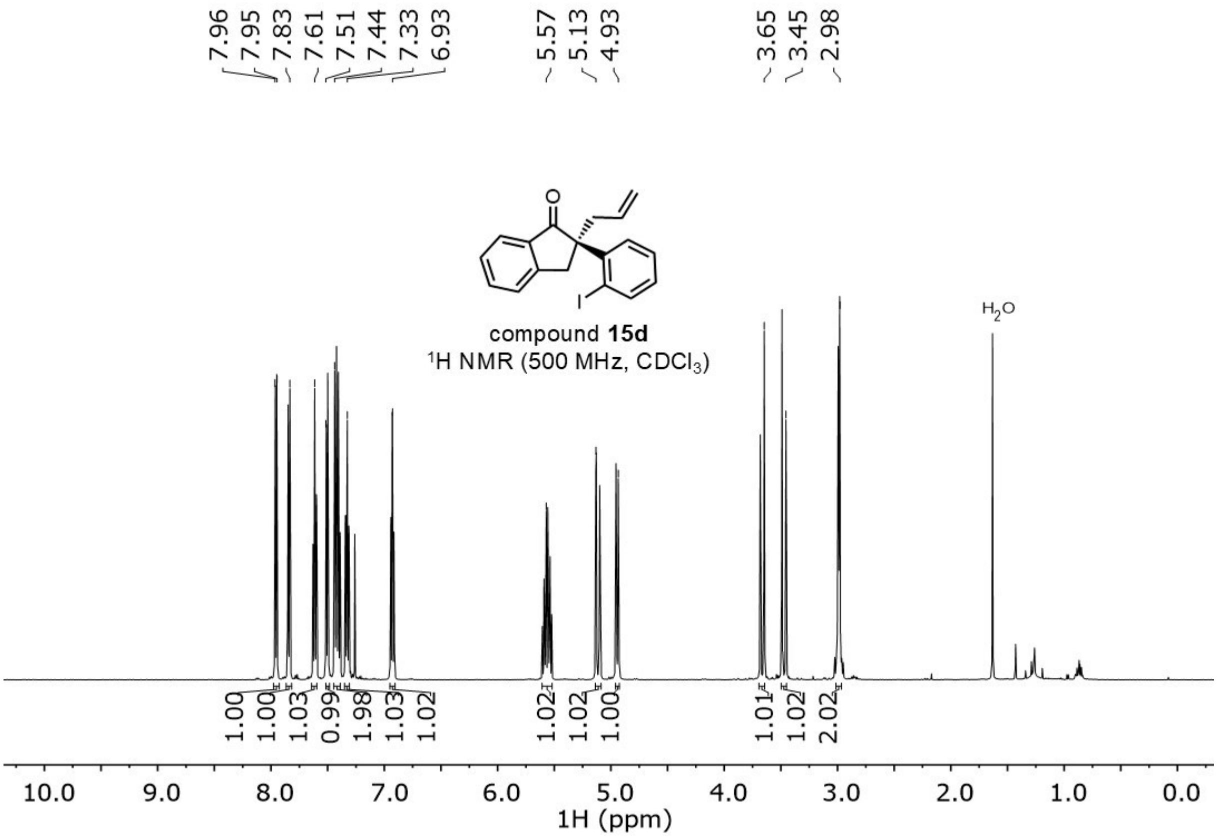
7.96
7.95
7.83
7.61
7.51
7.44
7.33
6.93
5.57
5.13
4.93
3.65
3.45
2.98
O
I
compound **15d**
^{1}H NMR (500 MHz, $CDCl_3$)
H_2O
1.00
1.00
1.03
0.99
1.98
1.03
1.02
1.02
1.02
1.00
1.01
1.02
2.02
10.0 9.0 8.0 7.0 6.0 5.0 4.0 3.0 2.0 1.0 0.0
1H (ppm)

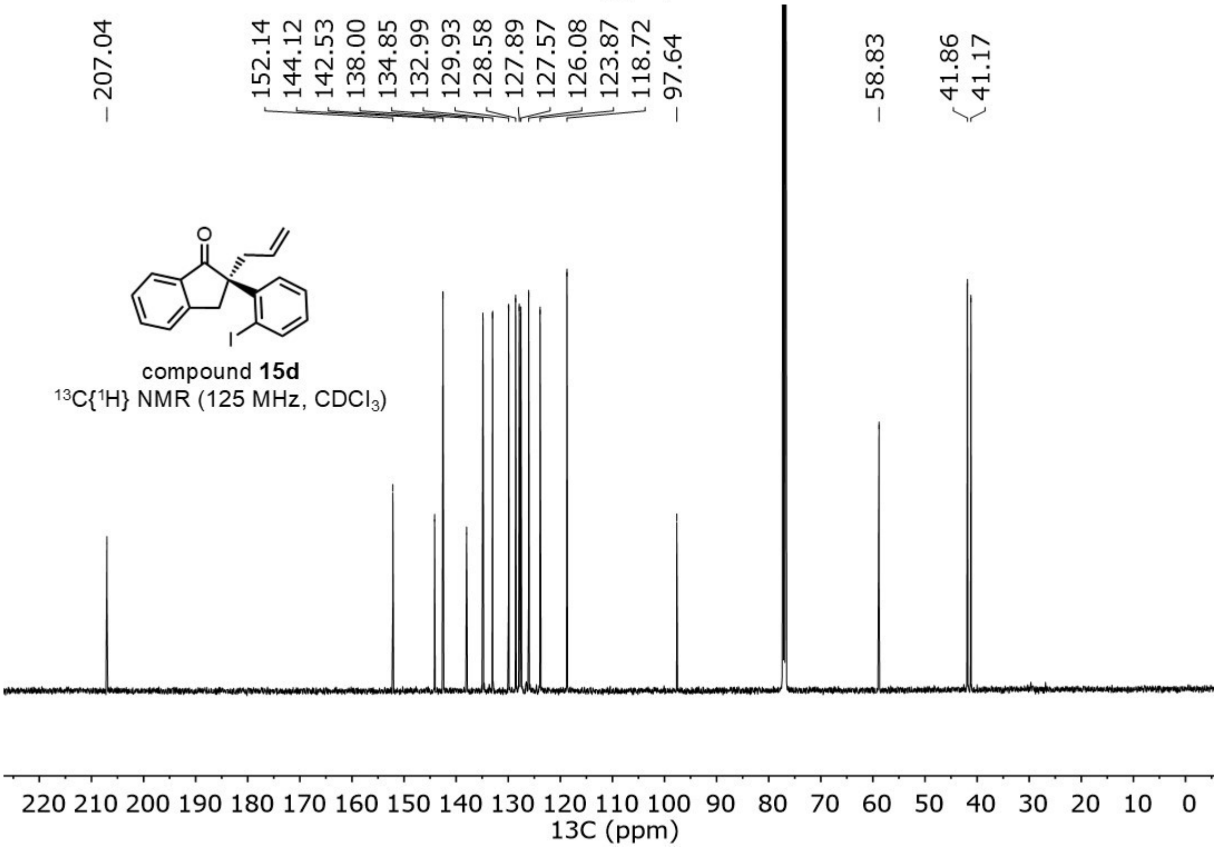
207.04
152.14
144.12
142.53
138.00
134.85
132.99
129.93
128.58
127.89
127.57
126.08
123.87
118.72
97.64
58.83
41.86
41.17
O
I
compound **15d**
$^{13}C\{^{1}H\}$ NMR (125 MHz, $CDCl_3$)
220 210 200 190 180 170 160 150 140 130 120 110 100 90 80 70 60 50 40 30 20 10 0
13C (ppm)

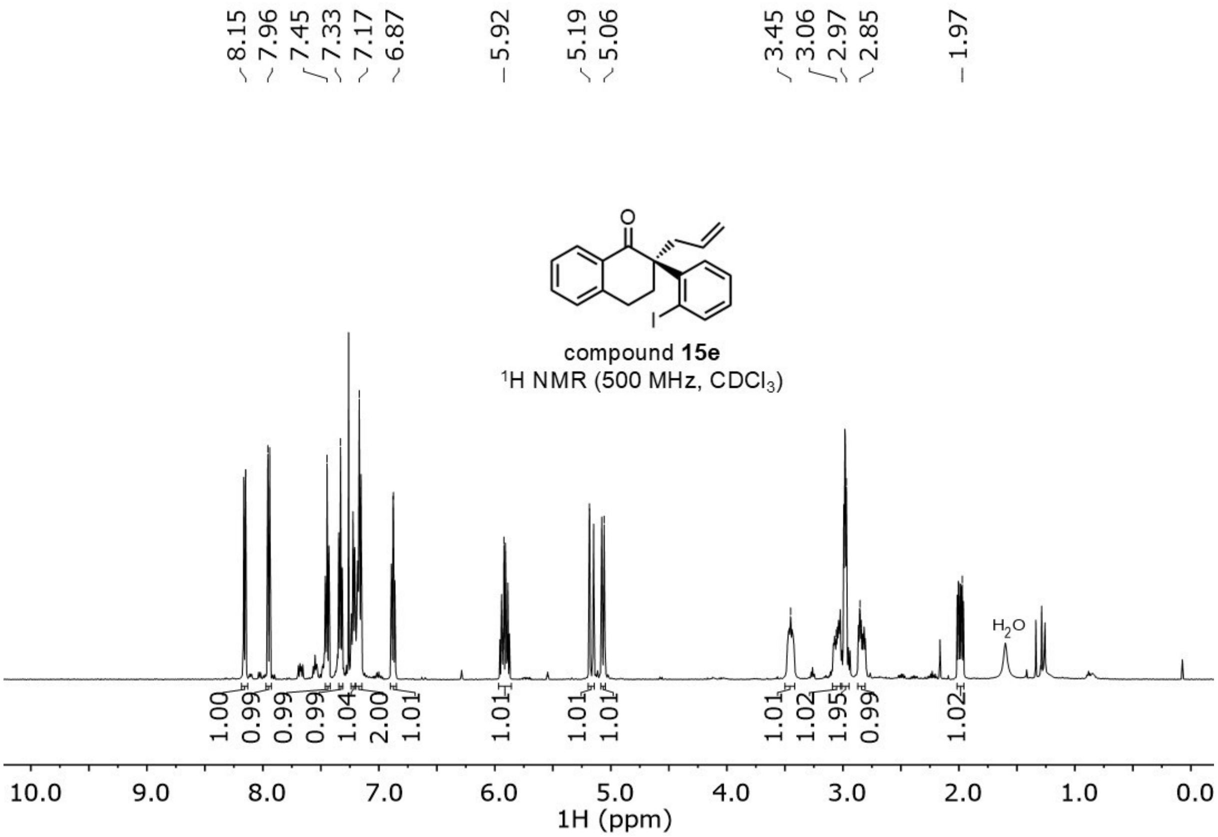
8.15
7.96
7.45
7.33
7.17
6.87
5.92
5.19
5.06
3.45
3.06
2.97
2.85
1.97
O
I
compound **15e**
^{1}H NMR (500 MHz, $CDCl_3$)
H_2O
1.00
0.99
0.99
0.99
1.04
2.00
1.01
1.01
1.01
1.01
1.01
1.02
1.95
0.99
1.02
10.0 9.0 8.0 7.0 6.0 5.0 4.0 3.0 2.0 1.0 0.0
1H (ppm)

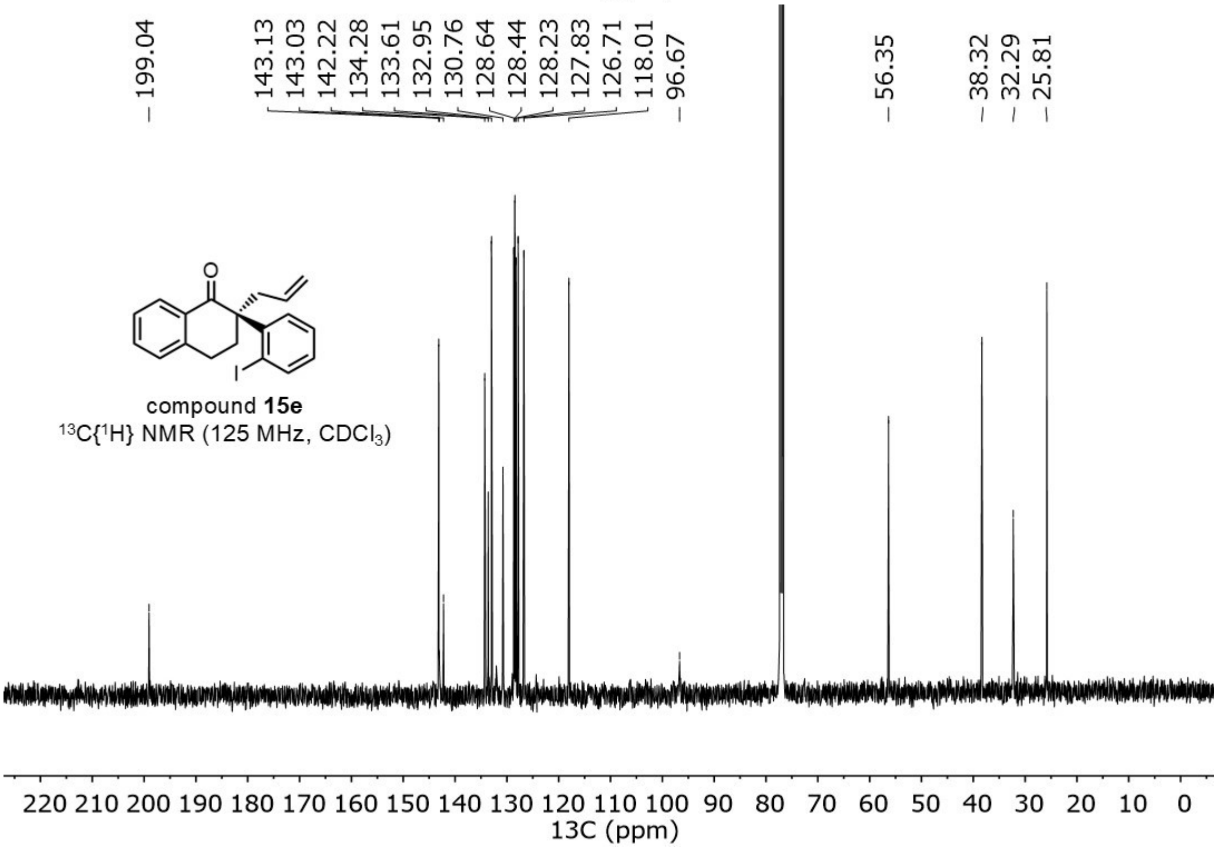
199.04
143.13
143.03
142.22
134.28
133.61
132.95
130.76
128.64
128.44
128.23
127.83
126.71
118.01
96.67
56.35
38.32
32.29
25.81
O
I
compound **15e**
$^{13}C\{^{1}H\}$ NMR (125 MHz, $CDCl_3$)
220 210 200 190 180 170 160 150 140 130 120 110 100 90 80 70 60 50 40 30 20 10 0
13C (ppm)

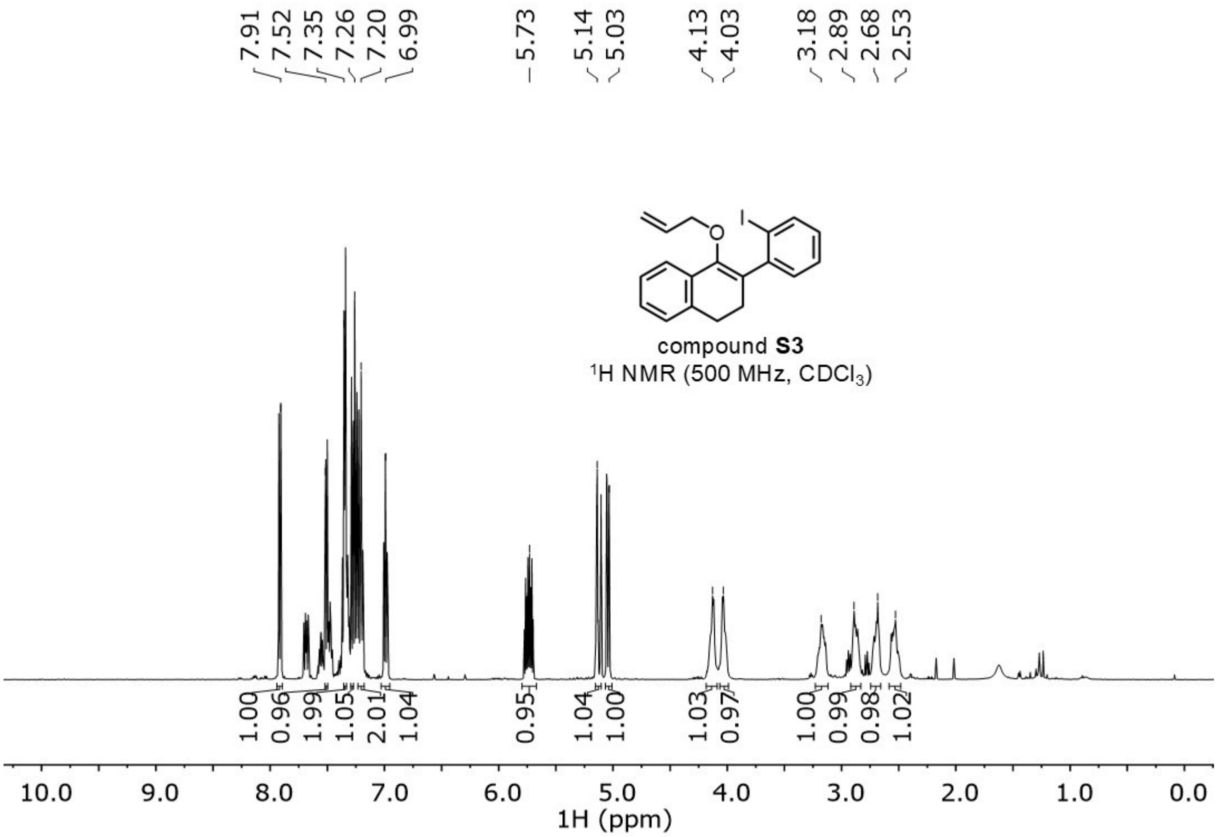
7.91
7.52
7.35
7.26
7.20
6.99
5.73
5.14
5.03
4.13
4.03
3.18
2.89
2.68
2.53
I
O
compound S3
1H NMR (500 MHz, CDCl3)
1.00
0.96
1.99
1.05
2.01
1.04
0.95
1.04
1.00
1.03
0.97
1.00
0.99
0.98
1.02
10.0 9.0 8.0 7.0 6.0 5.0 4.0 3.0 2.0 1.0 0.0
1H (ppm)

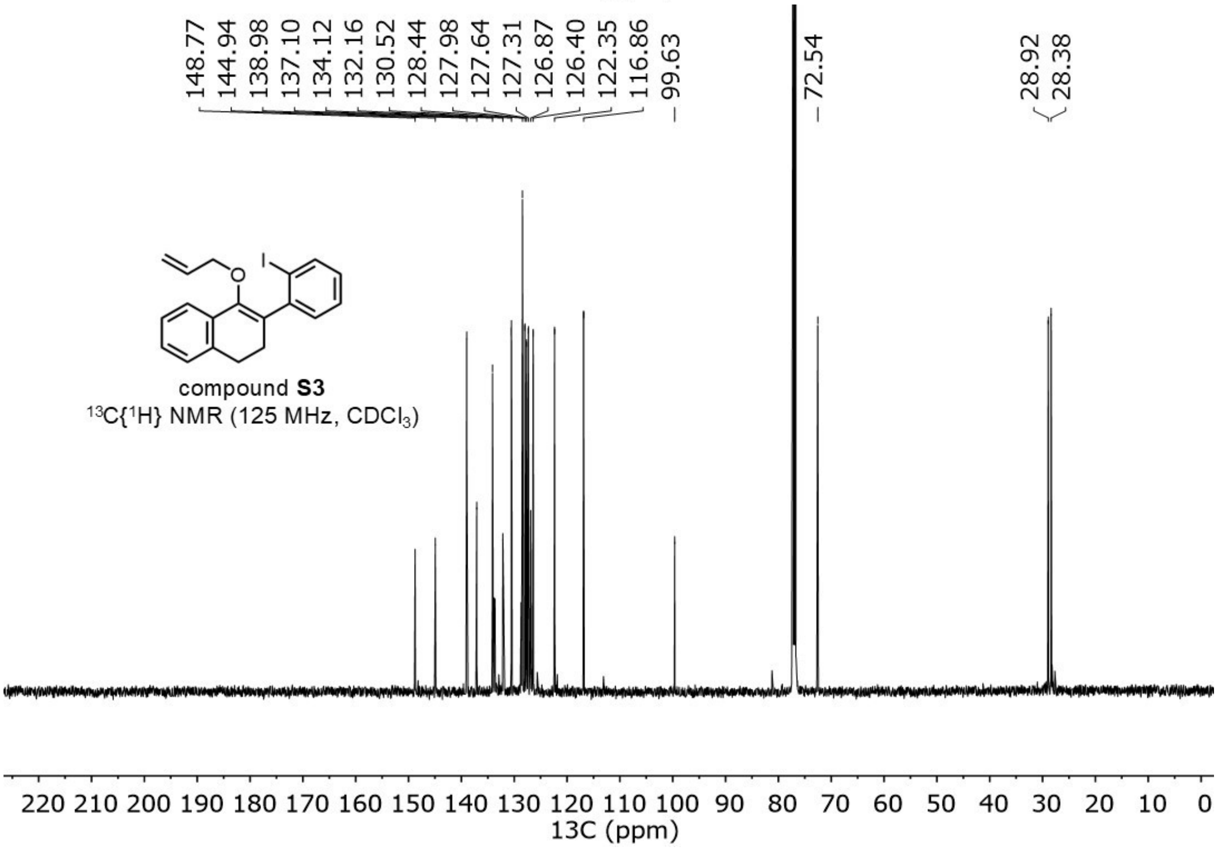
148.77
144.94
138.98
137.10
134.12
132.16
130.52
128.44
127.98
127.64
127.31
126.87
126.40
122.35
116.86
99.63
72.54
28.92
28.38
I
O
compound S3
13C{1H} NMR (125 MHz, CDCl3)
220 210 200 190 180 170 160 150 140 130 120 110 100 90 80 70 60 50 40 30 20 10 0
13C (ppm)

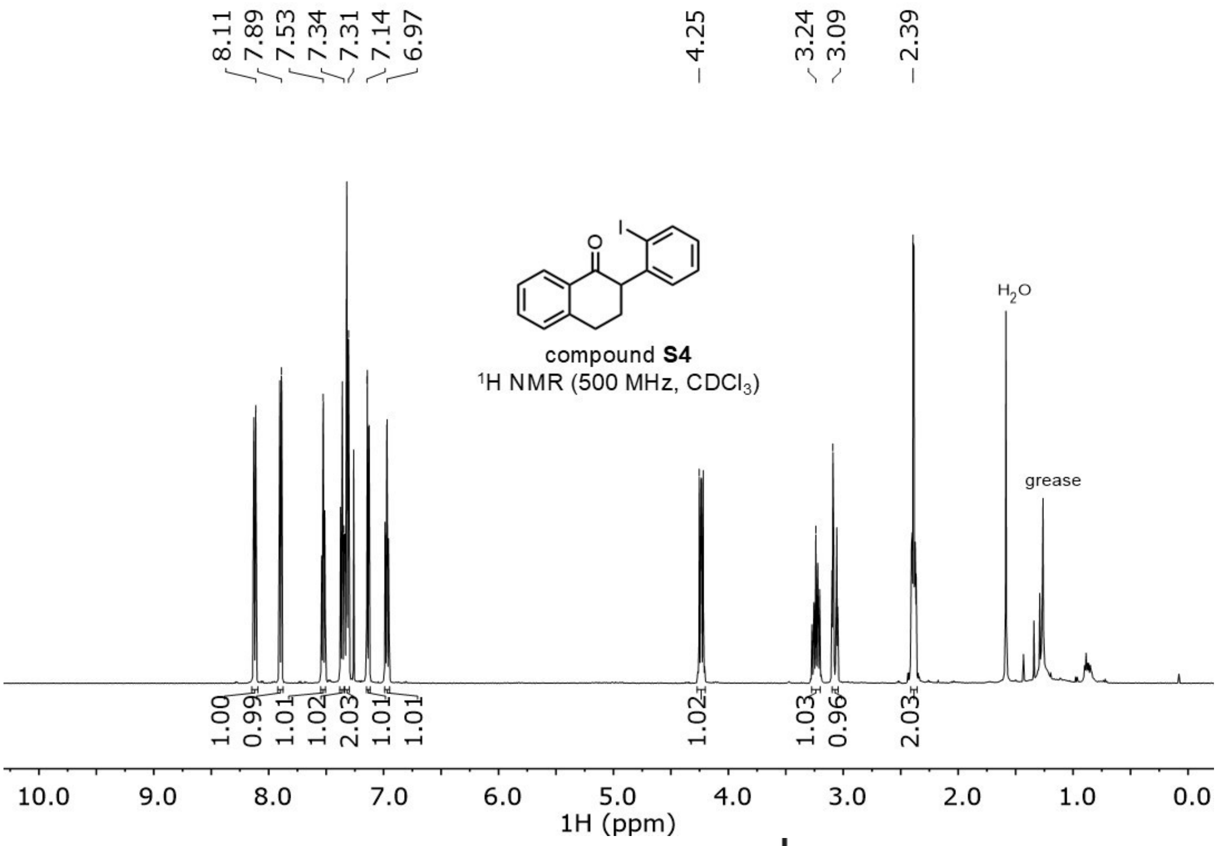
8.11
7.89
7.53
7.34
7.31
7.14
6.97
4.25
3.24
3.09
2.39
H2O
grease
compound S4
1H NMR (500 MHz, CDCl3)
1.00
0.99
1.01
1.02
2.03
1.01
1.01
1.02
1.03
0.96
2.03
10.0 9.0 8.0 7.0 6.0 5.0 4.0 3.0 2.0 1.0 0.0
1H (ppm)

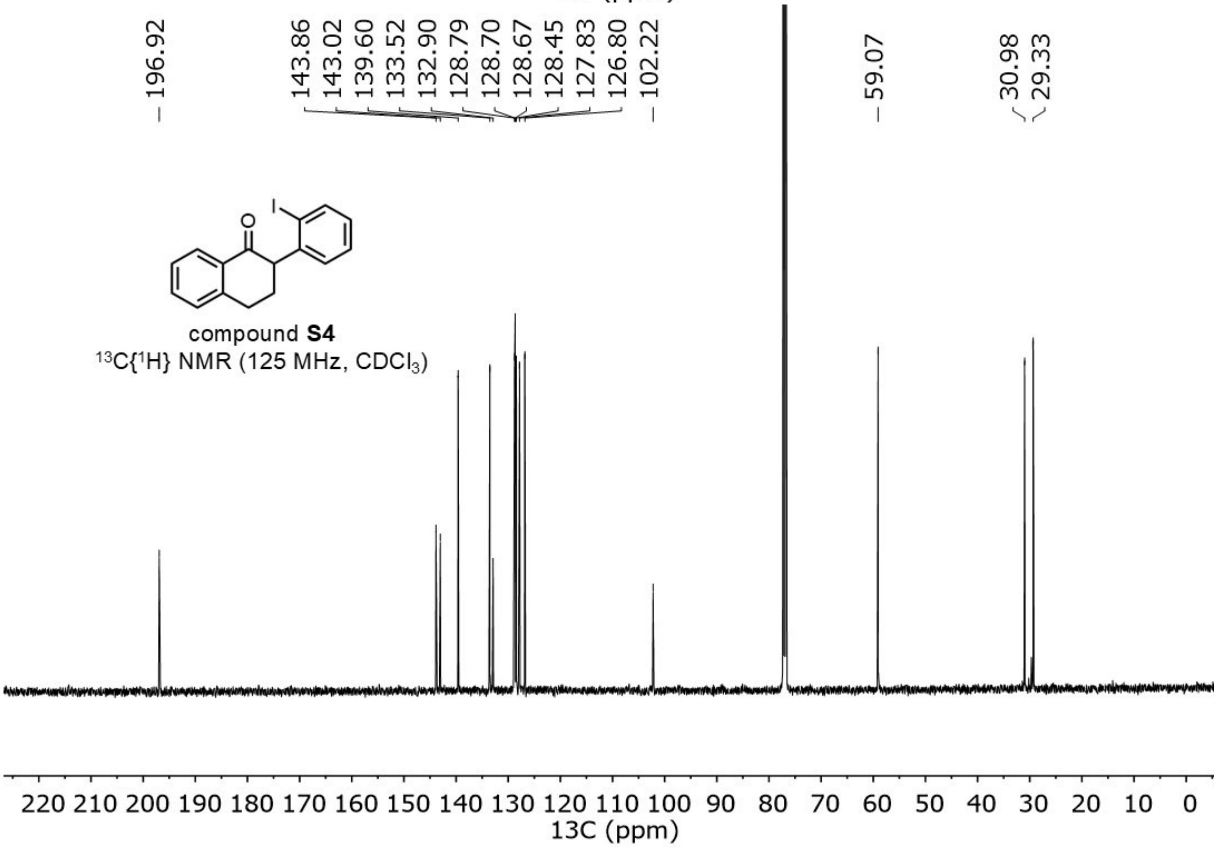
196.92
143.86
143.02
139.60
133.52
132.90
128.79
128.70
128.67
128.45
127.83
126.80
102.22
59.07
30.98
29.33
compound S4
13C{1H} NMR (125 MHz, CDCl3)
220 210 200 190 180 170 160 150 140 130 120 110 100 90 80 70 60 50 40 30 20 10 0
13C (ppm)

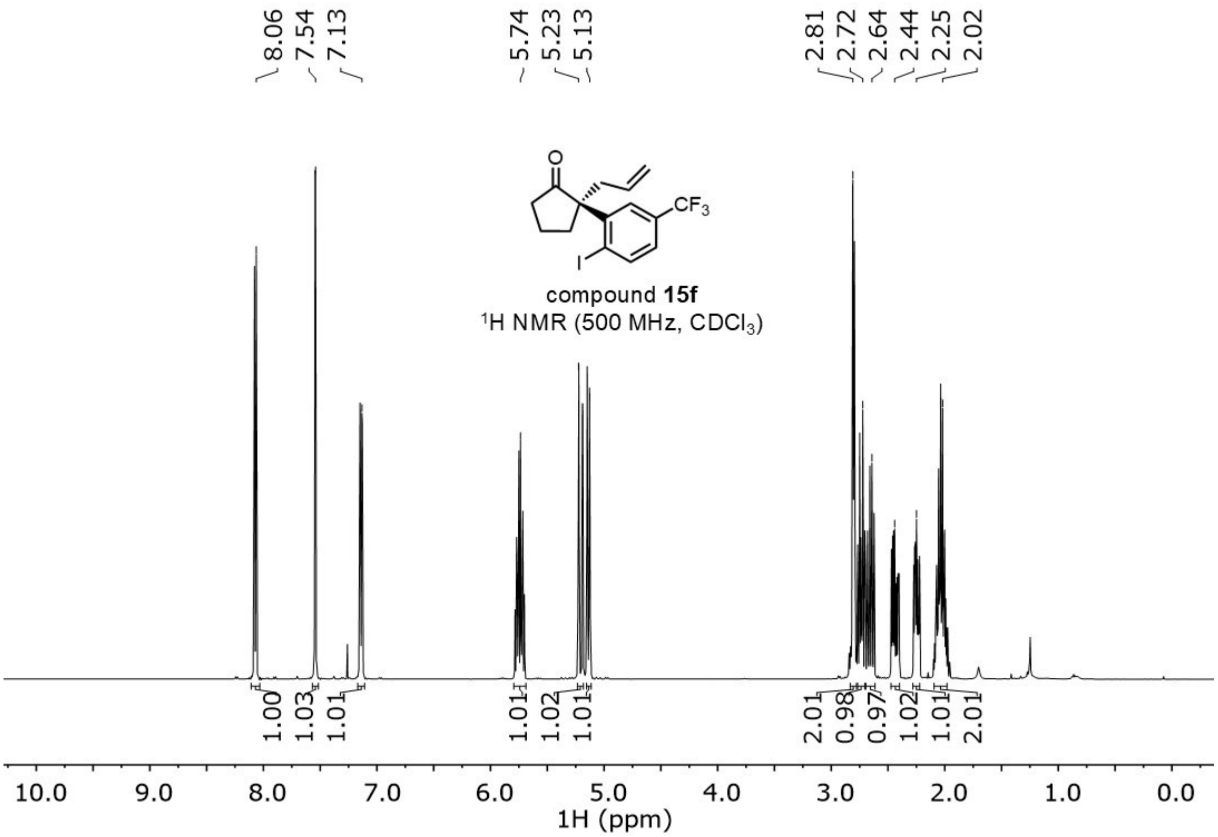
8.06
7.54
7.13
5.74
5.23
5.13
2.81
2.72
2.64
2.44
2.25
2.02
CF3
O
I
compound 15f
1H NMR (500 MHz, CDCl3)
1.00
1.03
1.01
1.01
1.02
1.01
2.01
0.98
0.97
1.02
1.01
2.01
10.0
9.0
8.0
7.0
6.0
5.0
4.0
3.0
2.0
1.0
0.0
1H (ppm)

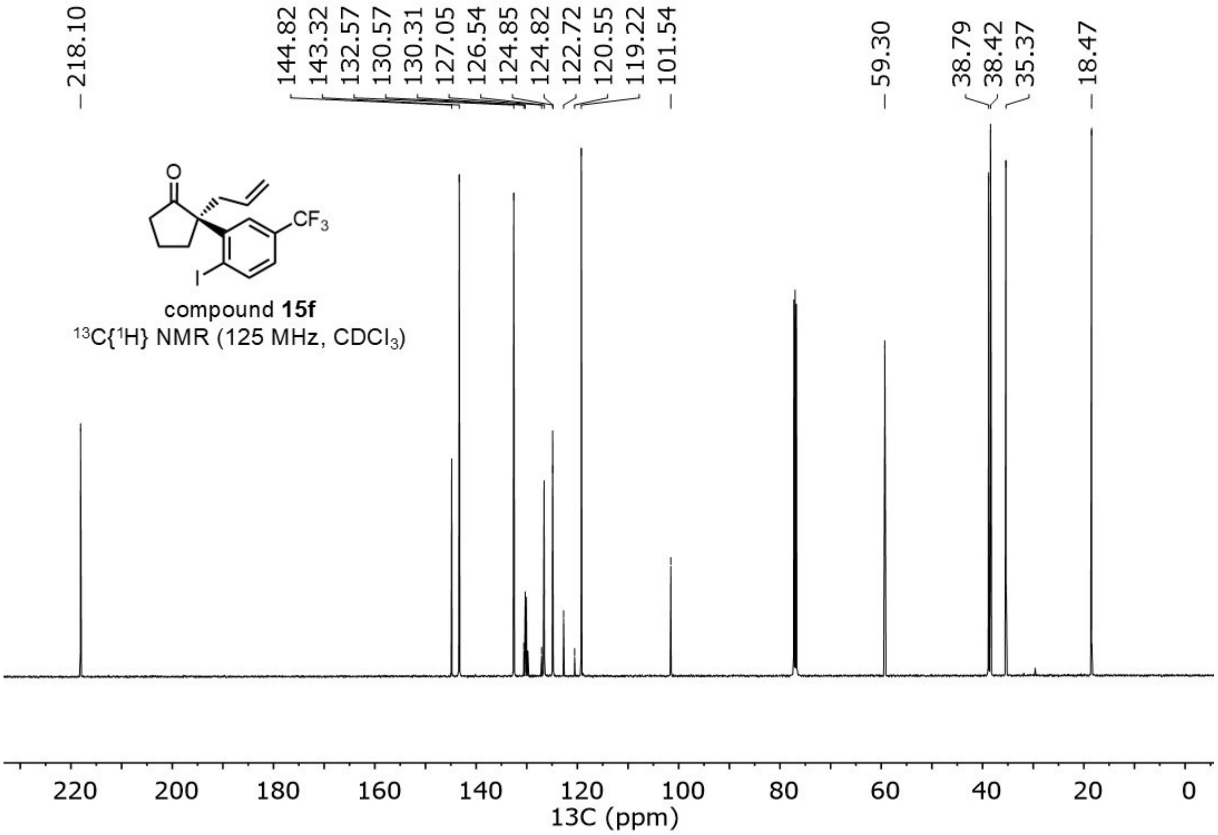
218.10
144.82
143.32
132.57
130.57
130.31
127.05
126.54
124.85
124.82
122.72
120.55
119.22
101.54
59.30
38.79
38.42
35.37
18.47
O
CF3
I
compound 15f
13C{1H} NMR (125 MHz, CDCl3)
220
200
180
160
140
120
100
80
60
40
20
0
13C (ppm)

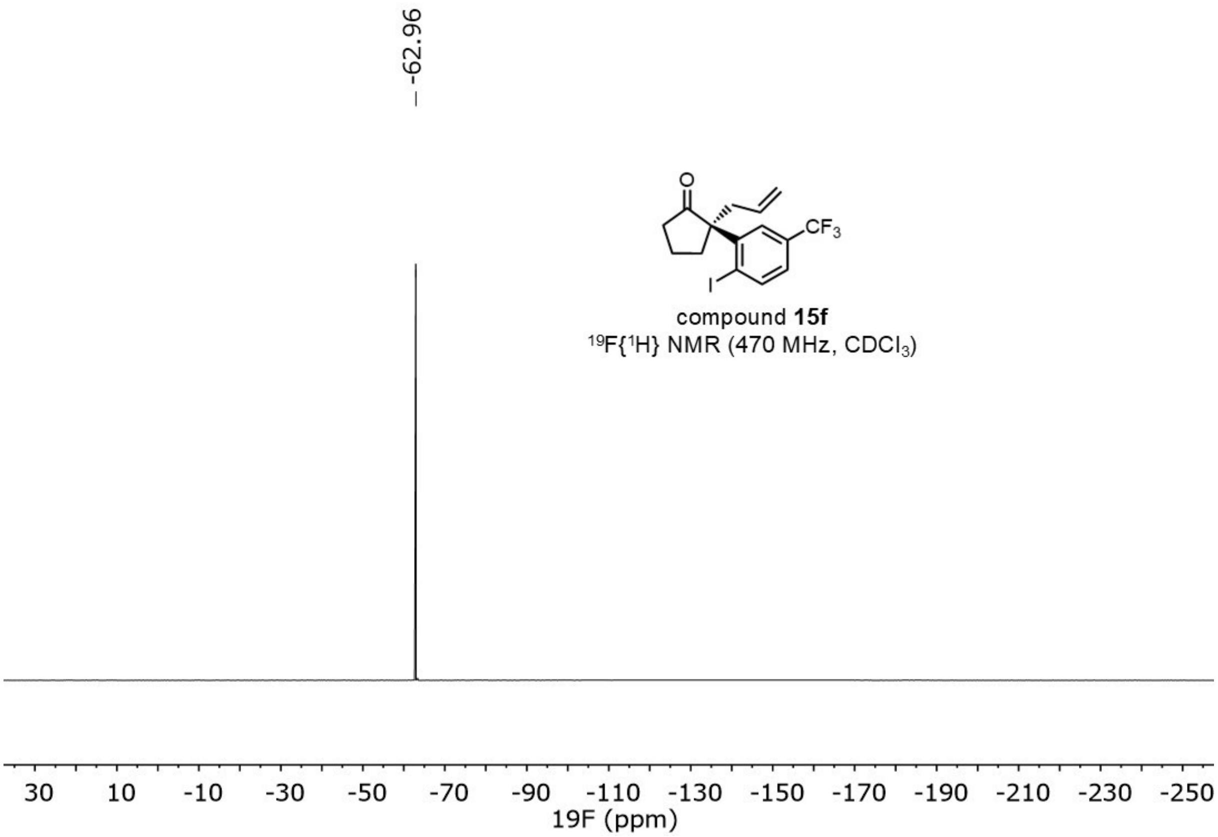

-62.96
CF3
O
I
compound 15f
19F{1H} NMR (470 MHz, CDCl3)
30 10 -10 -30 -50 -70 -90 -110 -130 -150 -170 -190 -210 -230 -250
19F (ppm)

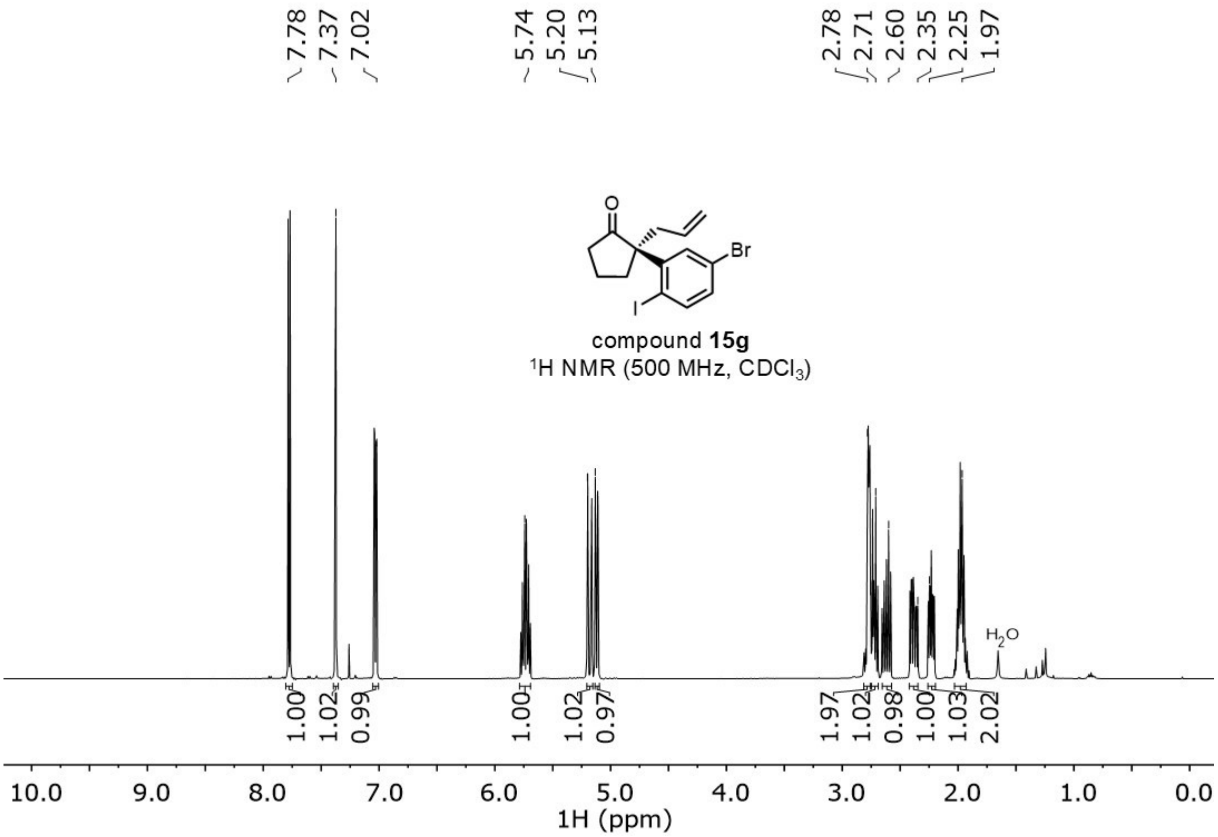
7.78
7.37
7.02
5.74
5.20
5.13
2.78
2.71
2.60
2.35
2.25
1.97
Br
I
O
compound 15g
1H NMR (500 MHz, CDCl3)
H2O
1.00
1.02
0.99
1.00
1.02
0.97
1.97
1.02
0.98
1.00
1.03
2.02
10.0
9.0
8.0
7.0
6.0
5.0
4.0
3.0
2.0
1.0
0.0
1H (ppm)

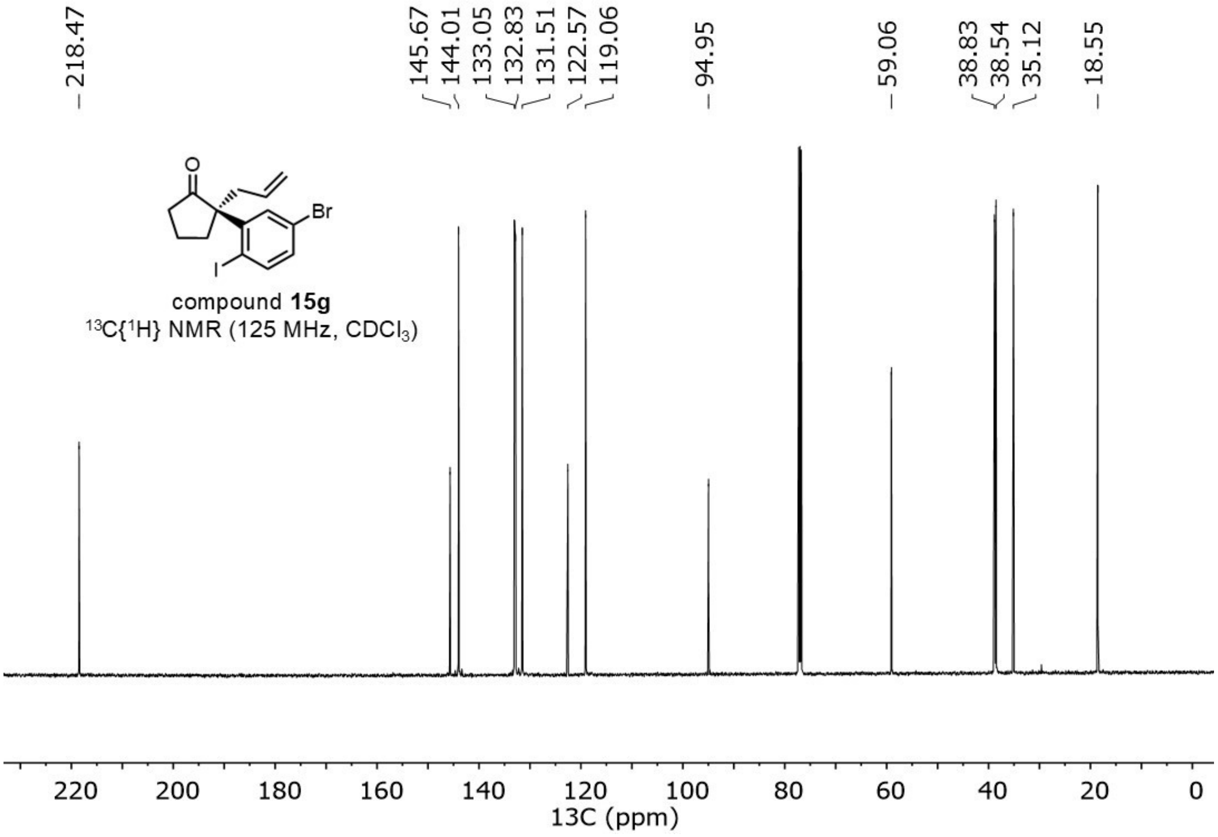
218.47
145.67
144.01
133.05
132.83
131.51
122.57
119.06
94.95
59.06
38.83
38.54
35.12
18.55
O
Br
I
compound 15g
13C{1H} NMR (125 MHz, CDCl3)
220
200
180
160
140
120
100
80
60
40
20
0
13C (ppm)

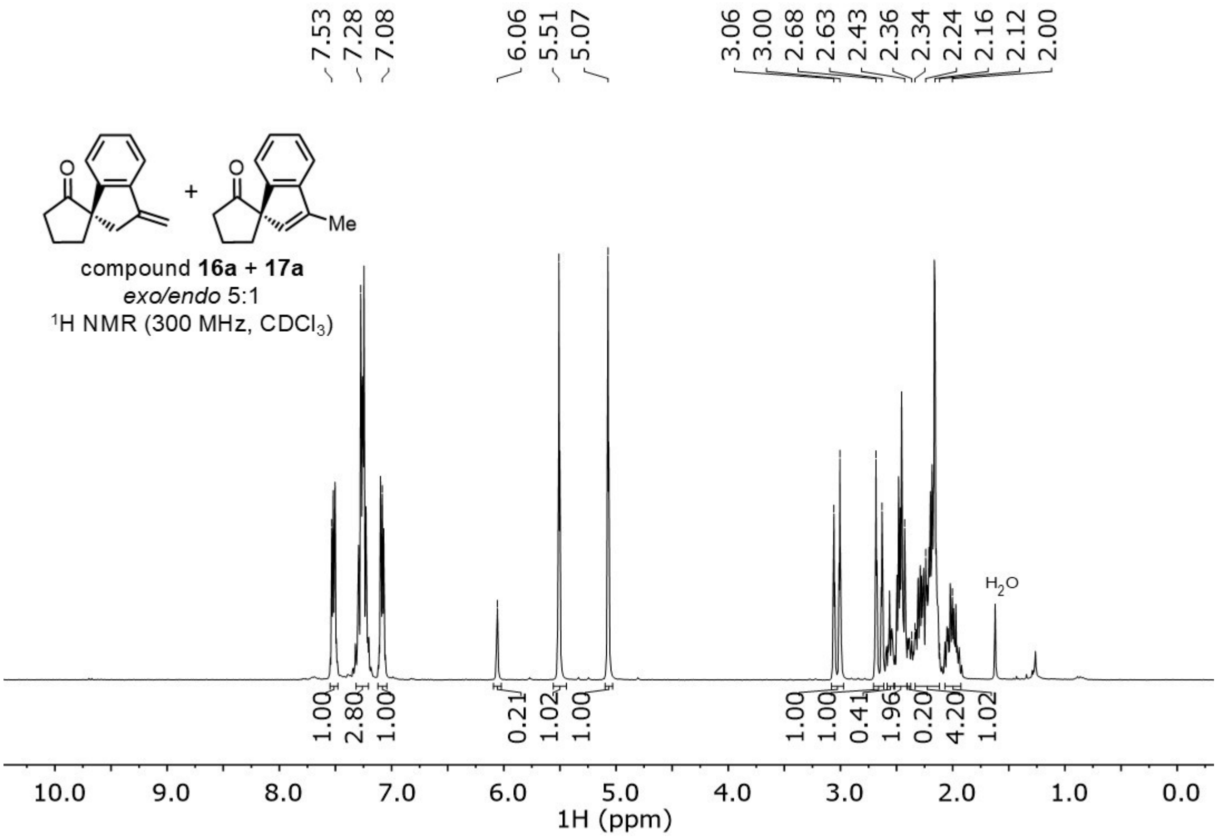

7.53
7.28
7.08
6.06
5.51
5.07
3.06
3.00
2.68
2.63
2.43
2.36
2.34
2.24
2.16
2.12
2.00
+
Me
compound **16a** + **17a**
exo/endo 5:1
^{1}H NMR (300 MHz, $CDCl_3$)
H_2O
1.00
2.80
1.00
0.21
1.02
1.00
1.00
1.00
0.41
1.96
0.20
4.20
1.02
10.0
9.0
8.0
7.0
6.0
5.0
4.0
3.0
2.0
1.0
0.0
1H (ppm)

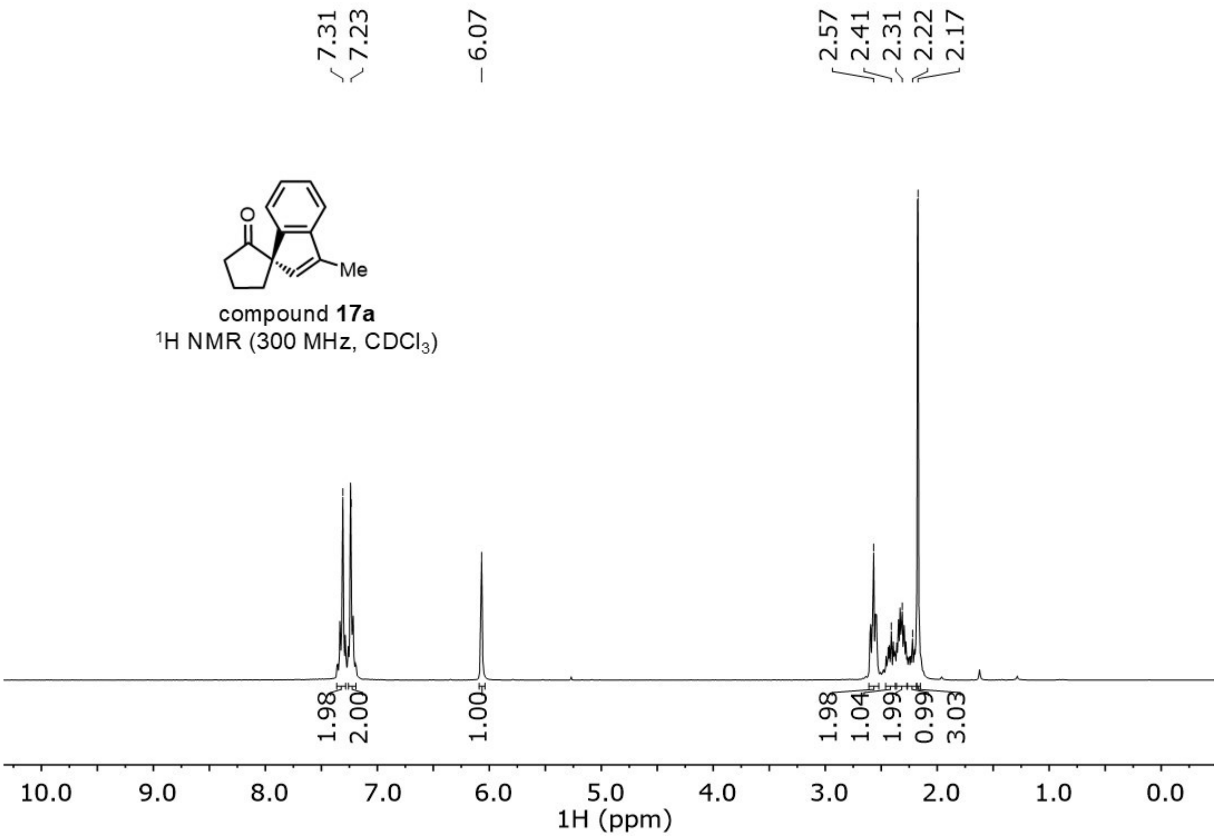
7.31
7.23
6.07
2.57
2.41
2.31
2.22
2.17
Me
O
compound **17a**
^{1}H NMR (300 MHz, $CDCl_3$)
1.98
2.00
1.00
1.98
1.04
1.99
0.99
3.03
10.0 9.0 8.0 7.0 6.0 5.0 4.0 3.0 2.0 1.0 0.0
1H (ppm)

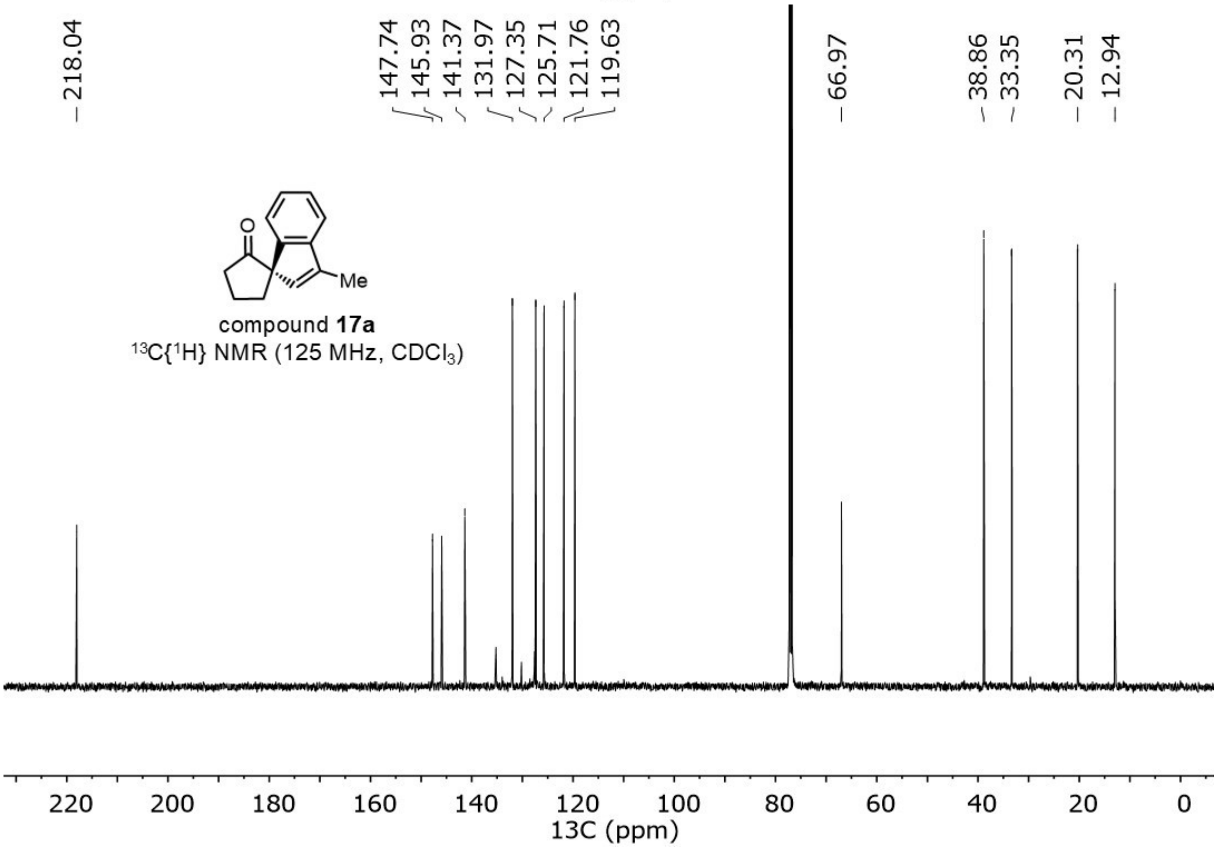
218.04
147.74
145.93
141.37
131.97
127.35
125.71
121.76
119.63
66.97
38.86
33.35
20.31
12.94
Me
O
compound **17a**
$^{13}C\{^1H\}$ NMR (125 MHz, $CDCl_3$)
220 200 180 160 140 120 100 80 60 40 20 0
13C (ppm)

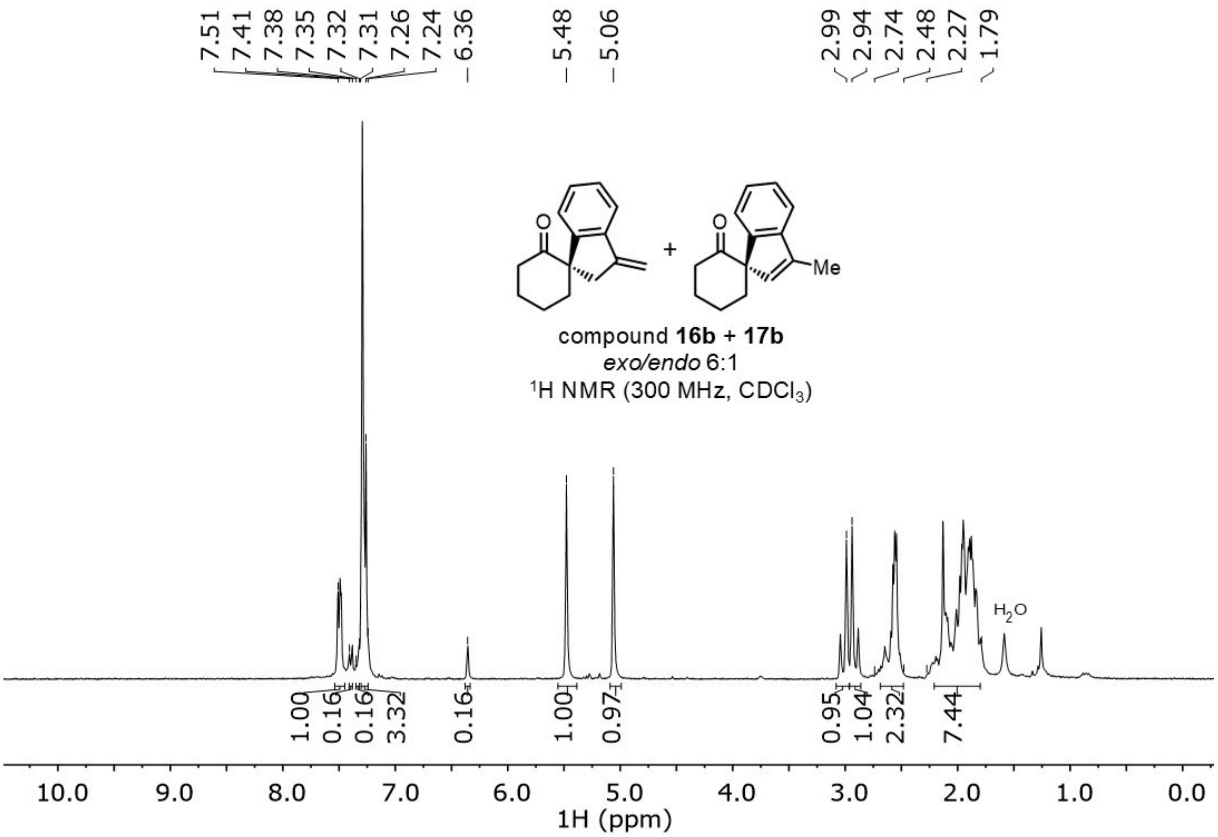
7.51
7.41
7.38
7.35
7.32
7.31
7.26
7.24
6.36
5.48
5.06
2.99
2.94
2.74
2.48
2.27
1.79
O
O
Me
+
compound **16b** + **17b**
exo/endo 6:1
^{1}H NMR (300 MHz, $CDCl_3$)
H_2O
1.00
0.16
0.16
3.32
0.16
1.00
0.97
0.95
1.04
2.32
7.44
10.0
9.0
8.0
7.0
6.0
5.0
4.0
3.0
2.0
1.0
0.0
1H (ppm)

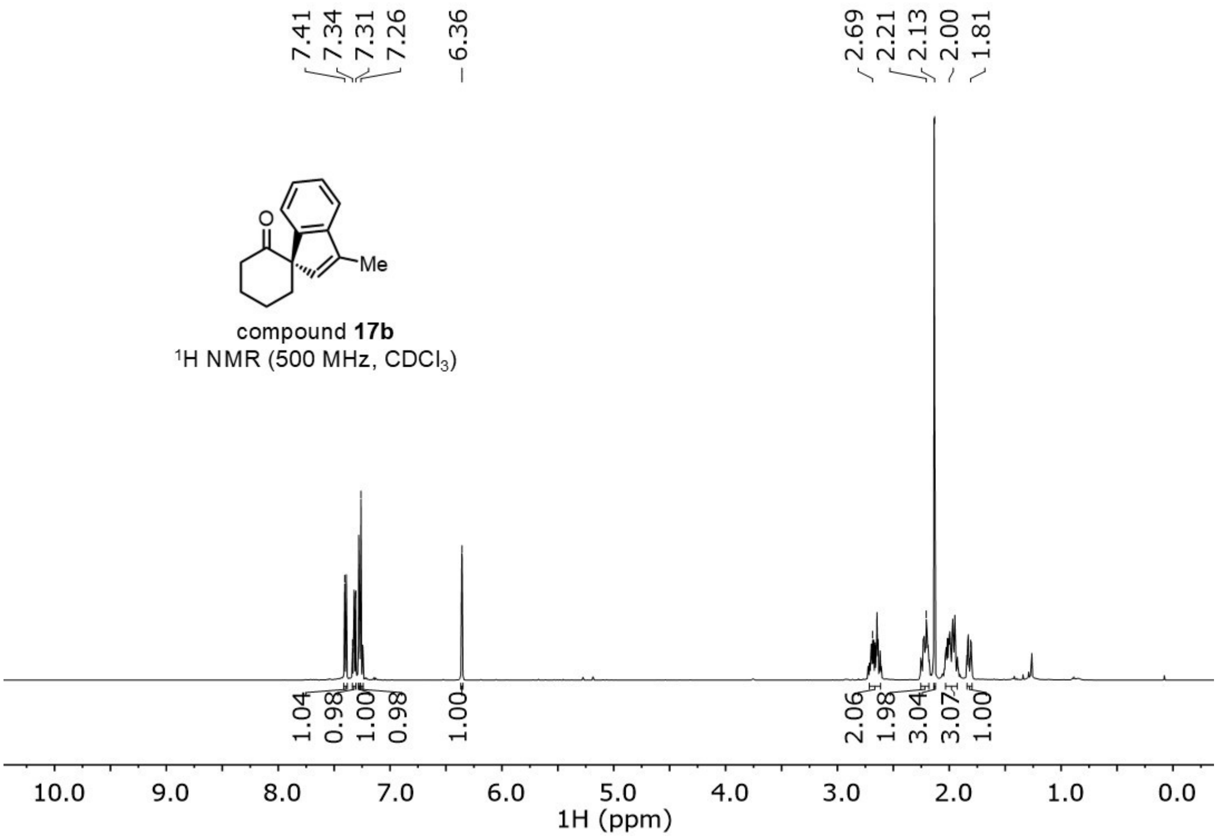
7.41
7.34
7.31
7.26
6.36
2.69
2.21
2.13
2.00
1.81
O
Me
compound 17b
1H NMR (500 MHz, CDCl3)
1.04
0.98
1.00
0.98
1.00
2.06
1.98
3.04
3.07
1.00
10.0 9.0 8.0 7.0 6.0 5.0 4.0 3.0 2.0 1.0 0.0
1H (ppm)

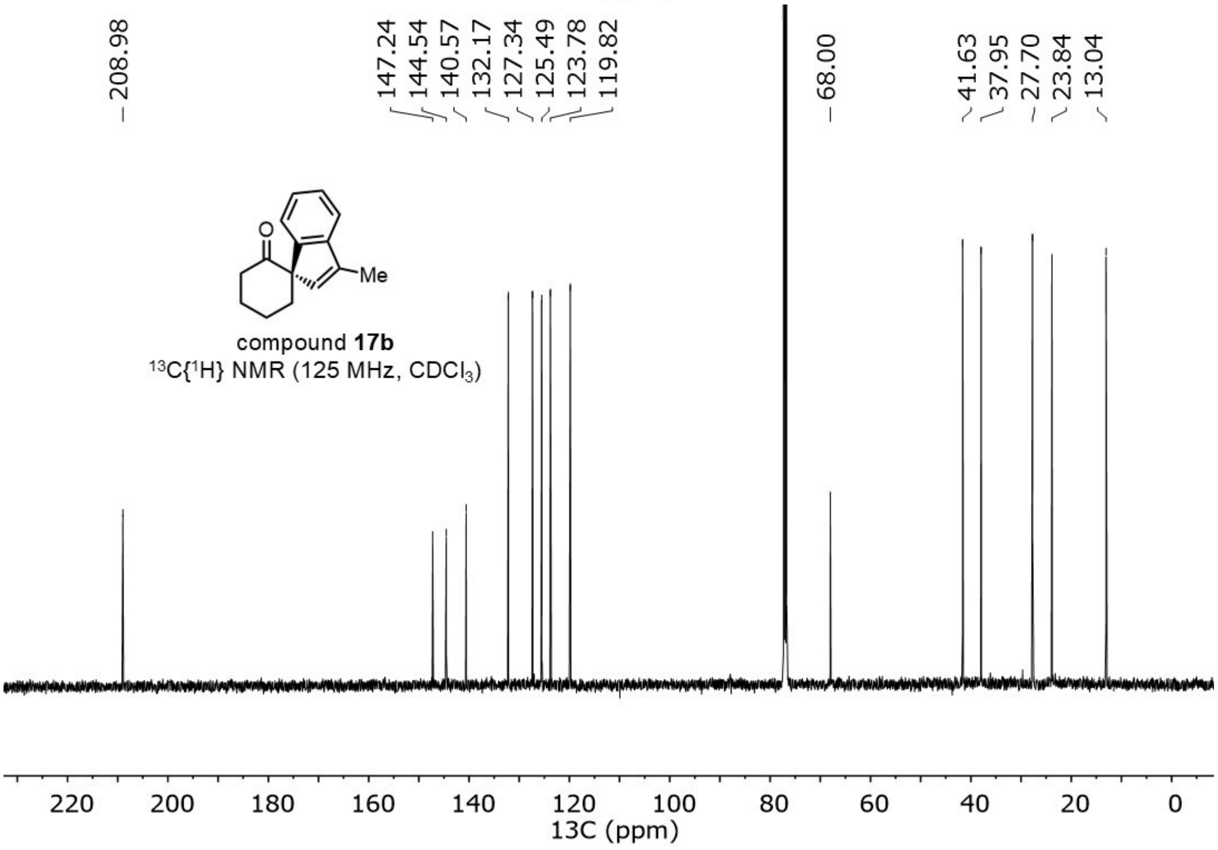
208.98
147.24
144.54
140.57
132.17
127.34
125.49
123.78
119.82
68.00
41.63
37.95
27.70
23.84
13.04
O
Me
compound 17b
13C{1H} NMR (125 MHz, CDCl3)
220 200 180 160 140 120 100 80 60 40 20 0
13C (ppm)

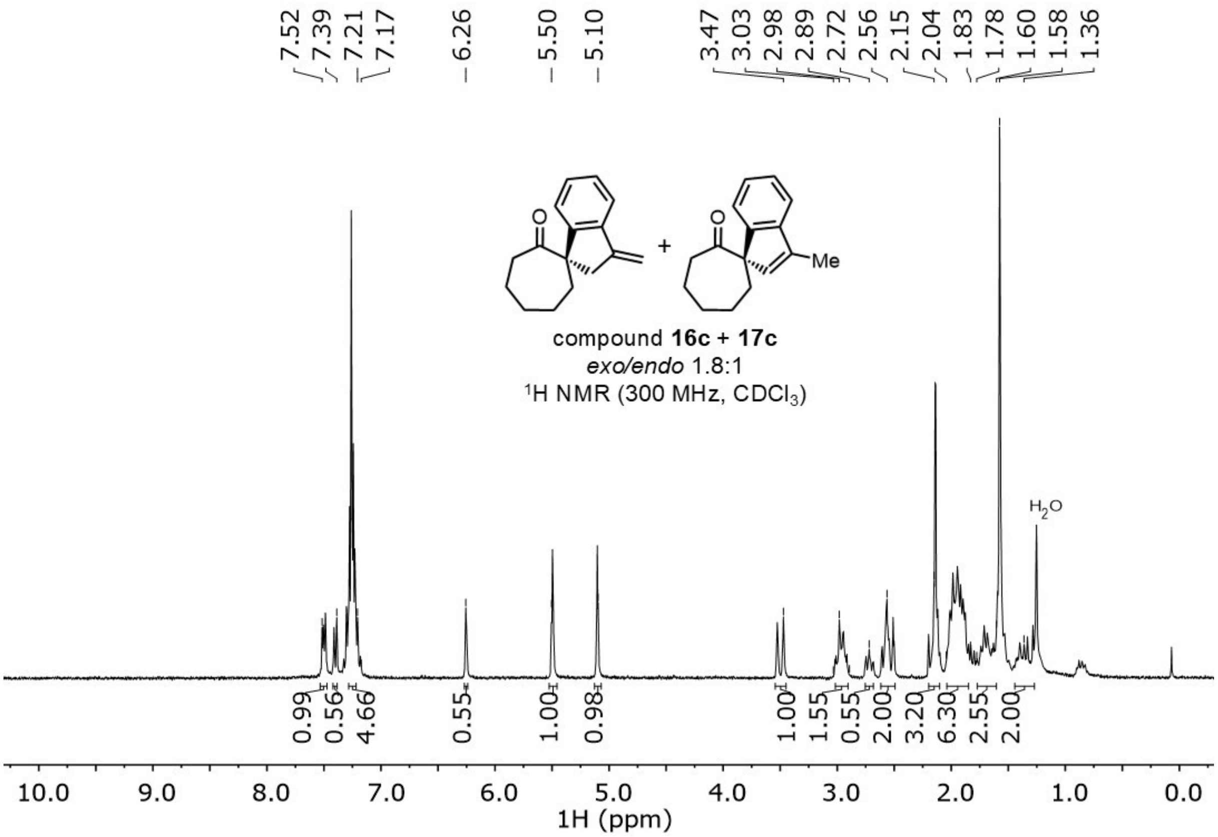
7.52
7.39
7.21
7.17
6.26
5.50
5.10
3.47
3.03
2.98
2.89
2.72
2.56
2.15
2.04
1.83
1.78
1.60
1.58
1.36
O
O
Me
compound **16c** + **17c**
exo/endo 1.8:1
^{1}H NMR (300 MHz, $CDCl_3$)
H_2O
0.99
0.56
4.66
0.55
1.00
0.98
1.00
1.55
0.55
2.00
3.20
6.30
2.55
2.00
10.0
9.0
8.0
7.0
6.0
5.0
4.0
3.0
2.0
1.0
0.0
1H (ppm)

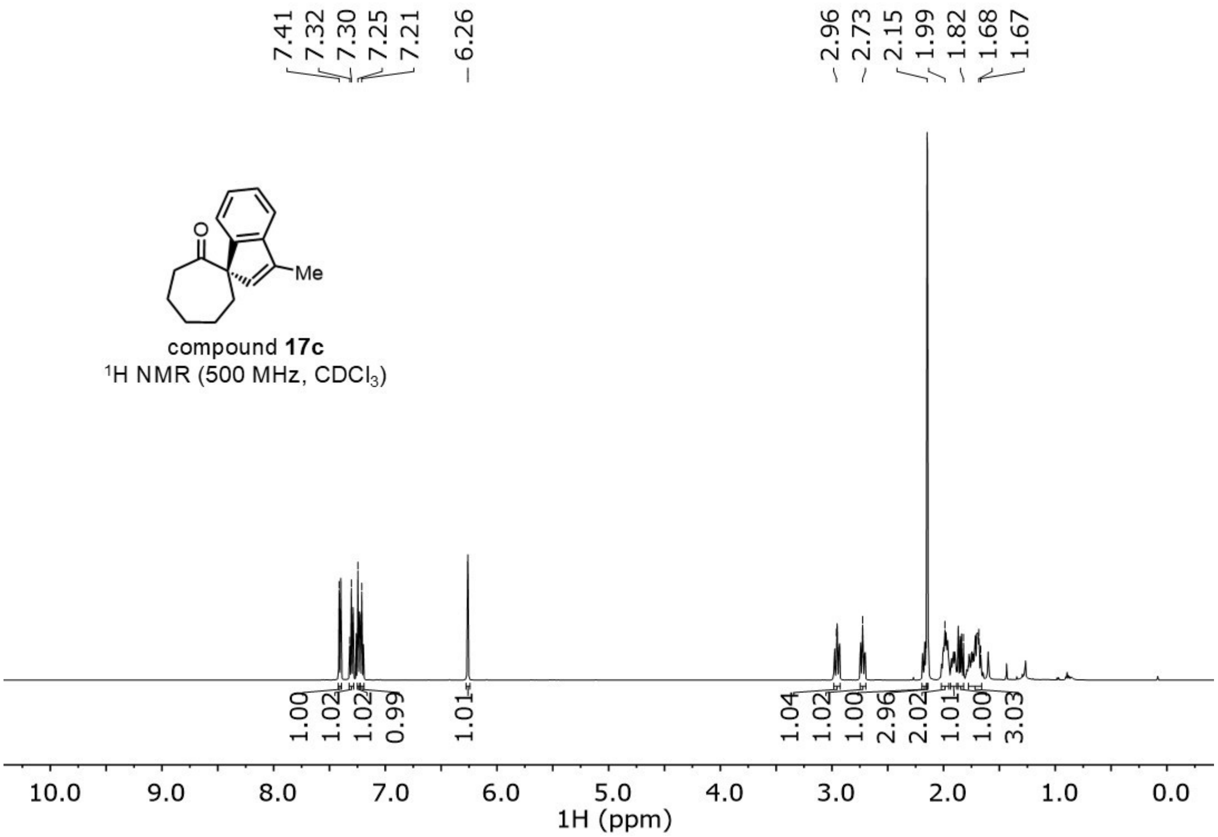
7.41
7.32
7.30
7.25
7.21
6.26
2.96
2.73
2.15
1.99
1.82
1.68
1.67
Me
compound 17c
1H NMR (500 MHz, CDCl3)
1.00
1.02
1.02
0.99
1.01
1.04
1.02
1.00
2.96
2.02
1.01
1.00
3.03
10.0
9.0
8.0
7.0
6.0
5.0
4.0
3.0
2.0
1.0
0.0
1H (ppm)

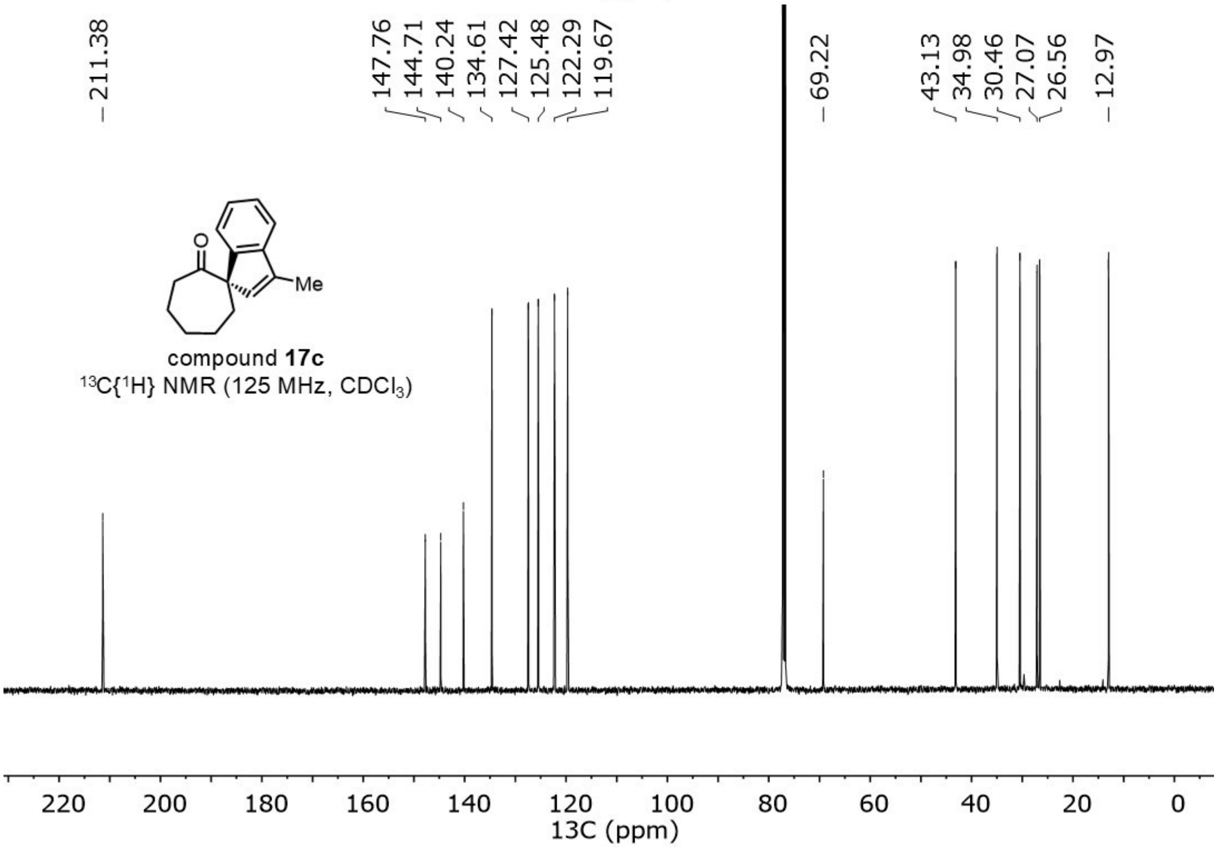
211.38
147.76
144.71
140.24
134.61
127.42
125.48
122.29
119.67
69.22
43.13
34.98
30.46
27.07
26.56
12.97
Me
compound 17c
13C{1H} NMR (125 MHz, CDCl3)
220
200
180
160
140
120
100
80
60
40
20
0
13C (ppm)

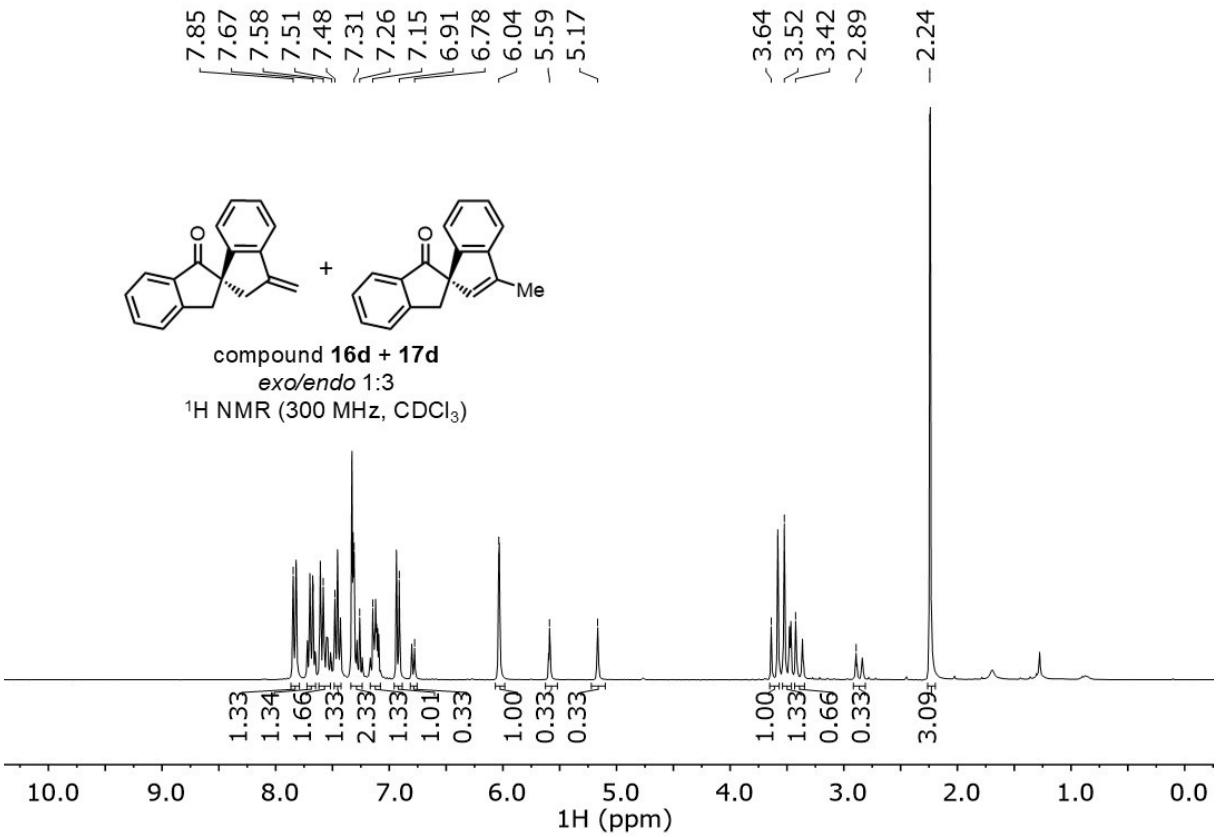
7.85
7.67
7.58
7.51
7.48
7.31
7.26
7.15
6.91
6.78
6.04
5.59
5.17
3.64
3.52
3.42
2.89
2.24
O
Me
compound **16d** + **17d**
exo/endo 1:3
^{1}H NMR (300 MHz, $CDCl_3$)
1.33
1.34
1.66
1.33
2.33
1.33
1.01
0.33
1.00
0.33
0.33
1.00
1.33
0.66
0.33
3.09
10.0
9.0
8.0
7.0
6.0
5.0
4.0
3.0
2.0
1.0
0.0
1H (ppm)

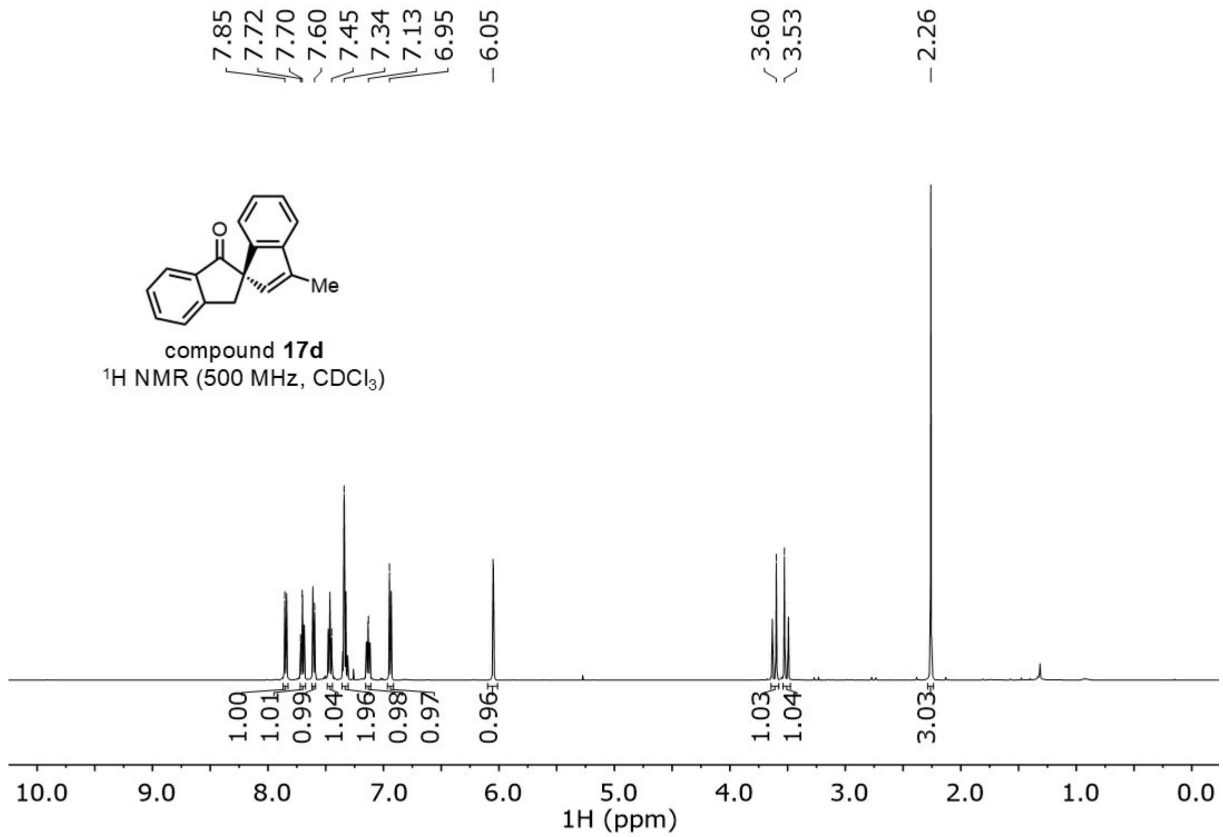
7.85
7.72
7.70
7.60
7.45
7.34
7.13
6.95
6.05
3.60
3.53
2.26
O
Me
compound **17d**
^{1}H NMR (500 MHz, $CDCl_3$)
1.00
1.01
0.99
1.04
1.96
0.98
0.97
0.96
1.03
1.04
3.03
10.0 9.0 8.0 7.0 6.0 5.0 4.0 3.0 2.0 1.0 0.0
1H (ppm)

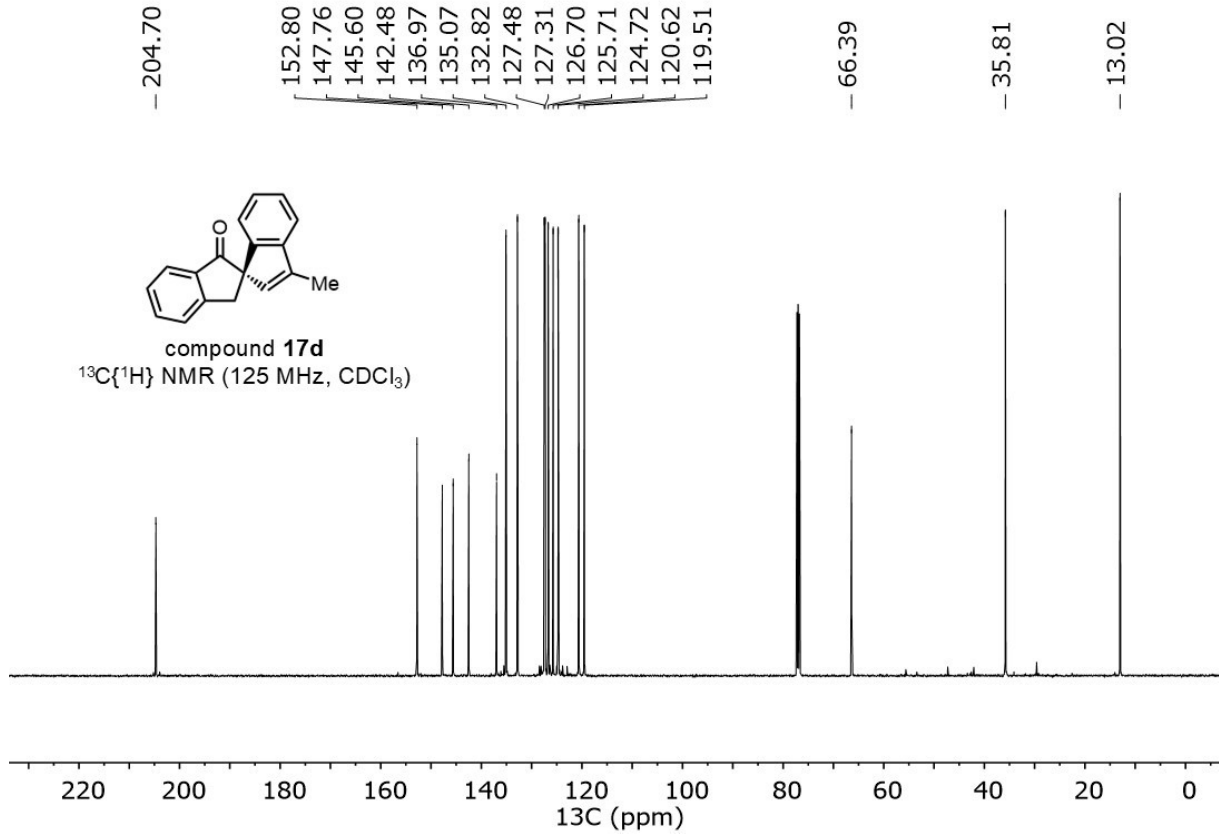
204.70
152.80
147.76
145.60
142.48
136.97
135.07
132.82
127.48
127.31
126.70
125.71
124.72
120.62
119.51
66.39
35.81
13.02
O
Me
compound **17d**
$^{13}C\{^{1}H\}$ NMR (125 MHz, $CDCl_3$)
220 200 180 160 140 120 100 80 60 40 20 0
13C (ppm)

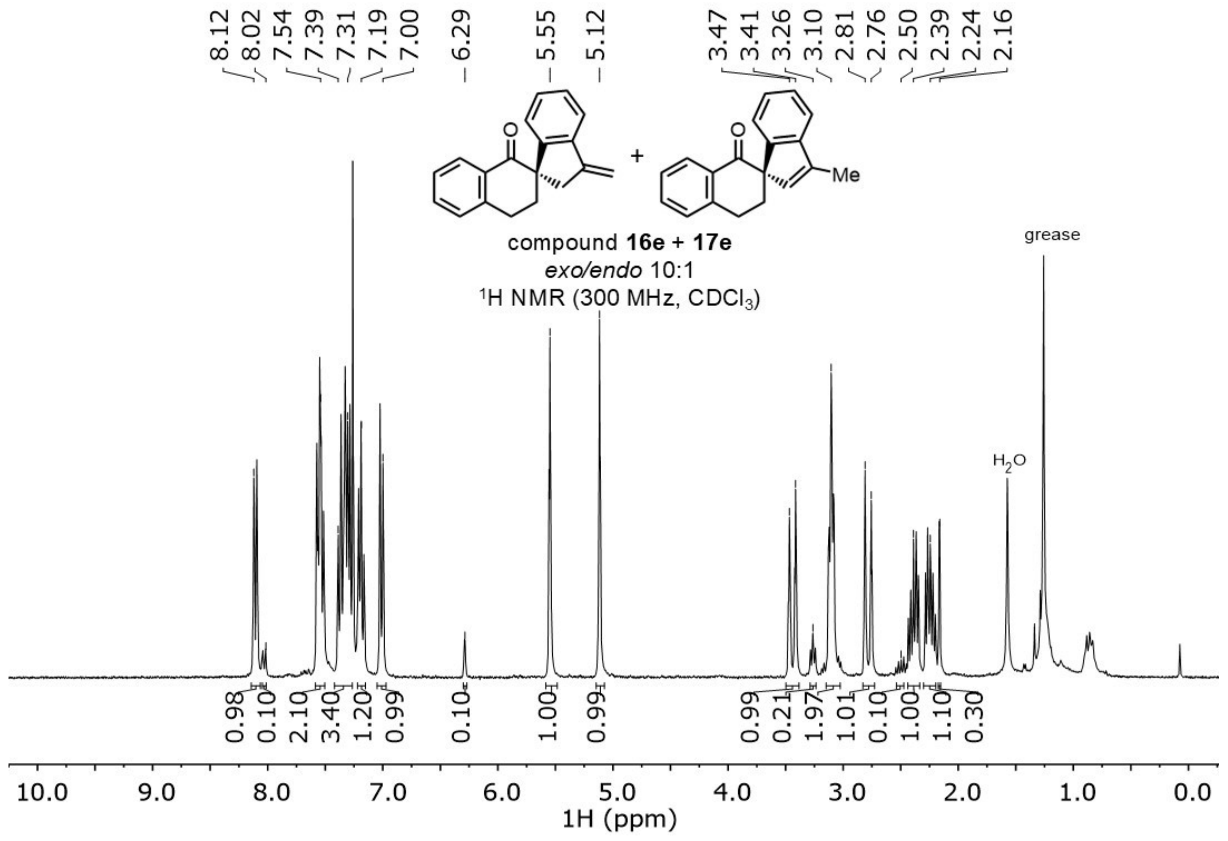
8.12
8.02
7.54
7.39
7.31
7.19
7.00
6.29
5.55
5.12
3.47
3.41
3.26
3.10
2.81
2.76
2.50
2.39
2.24
2.16
O
+
O
Me
compound 16e + 17e
exo/endo 10:1
1H NMR (300 MHz, CDCl3)
grease
H2O
0.98
0.10
2.10
3.40
1.20
0.99
0.10
1.00
0.99
0.99
0.21
1.97
1.01
0.10
1.00
1.10
0.30
10.0
9.0
8.0
7.0
6.0
5.0
4.0
3.0
2.0
1.0
0.0
1H (ppm)

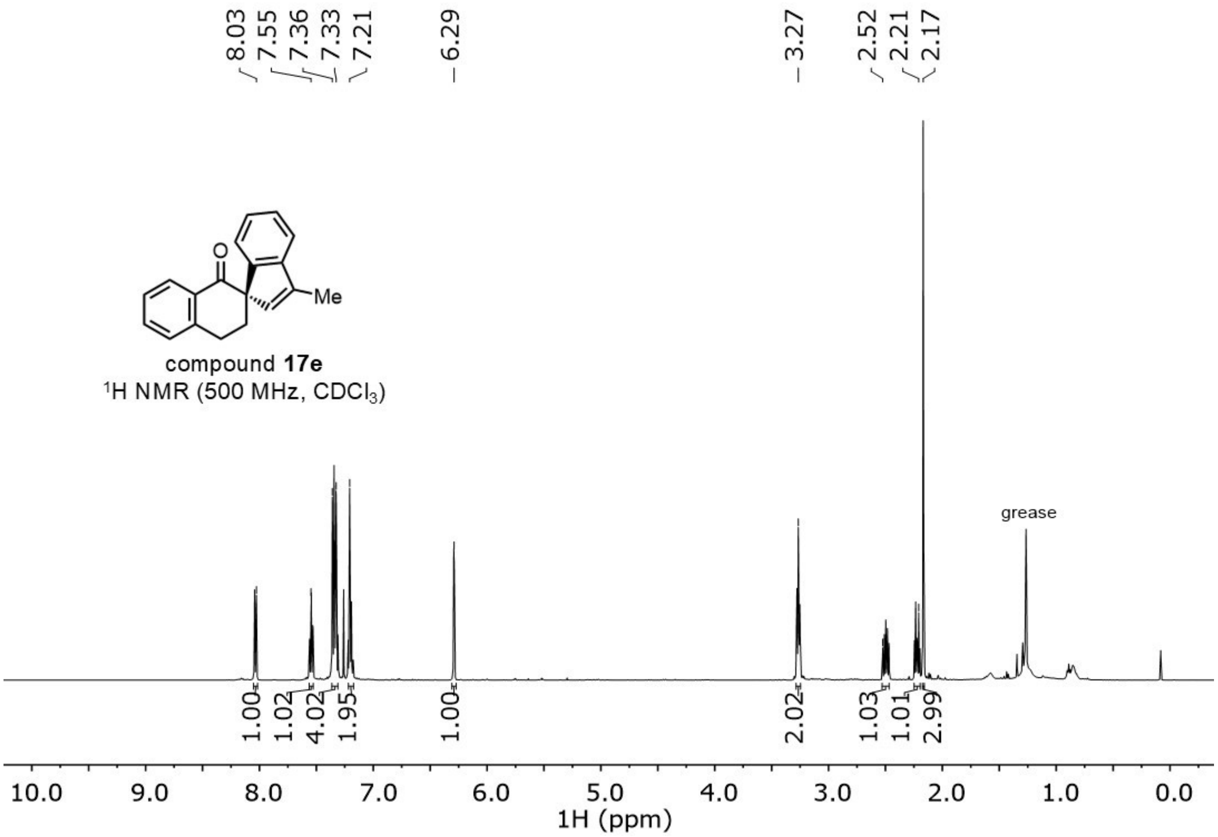
8.03
7.55
7.36
7.33
7.21
6.29
3.27
2.52
2.21
2.17
O
Me
compound 17e
1H NMR (500 MHz, CDCl3)
grease
1.00
1.02
4.02
1.95
1.00
2.02
1.03
1.01
2.99
10.0 9.0 8.0 7.0 6.0 5.0 4.0 3.0 2.0 1.0 0.0
1H (ppm)

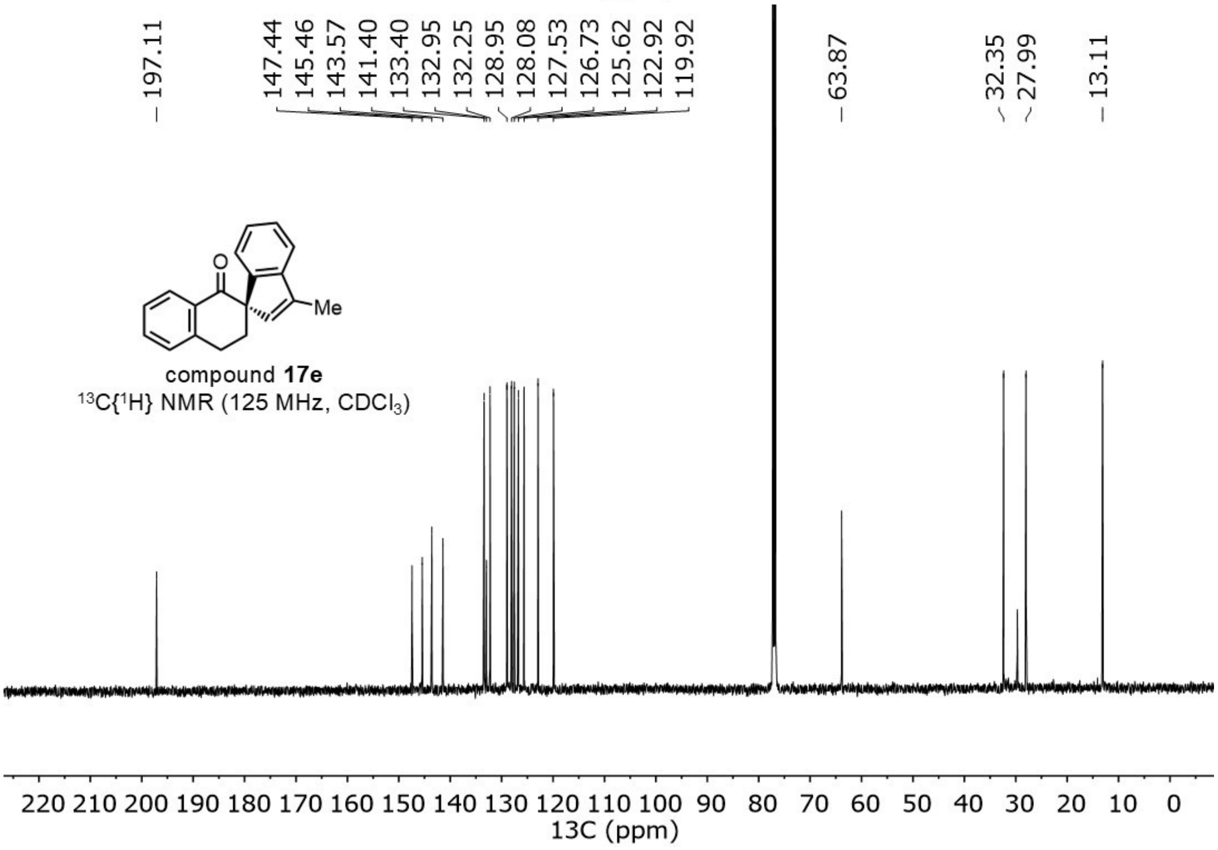
197.11
147.44
145.46
143.57
141.40
133.40
132.95
132.25
128.95
128.08
127.53
126.73
125.62
122.92
119.92
63.87
32.35
27.99
13.11
O
Me
compound 17e
13C{1H} NMR (125 MHz, CDCl3)
220 210 200 190 180 170 160 150 140 130 120 110 100 90 80 70 60 50 40 30 20 10 0
13C (ppm)

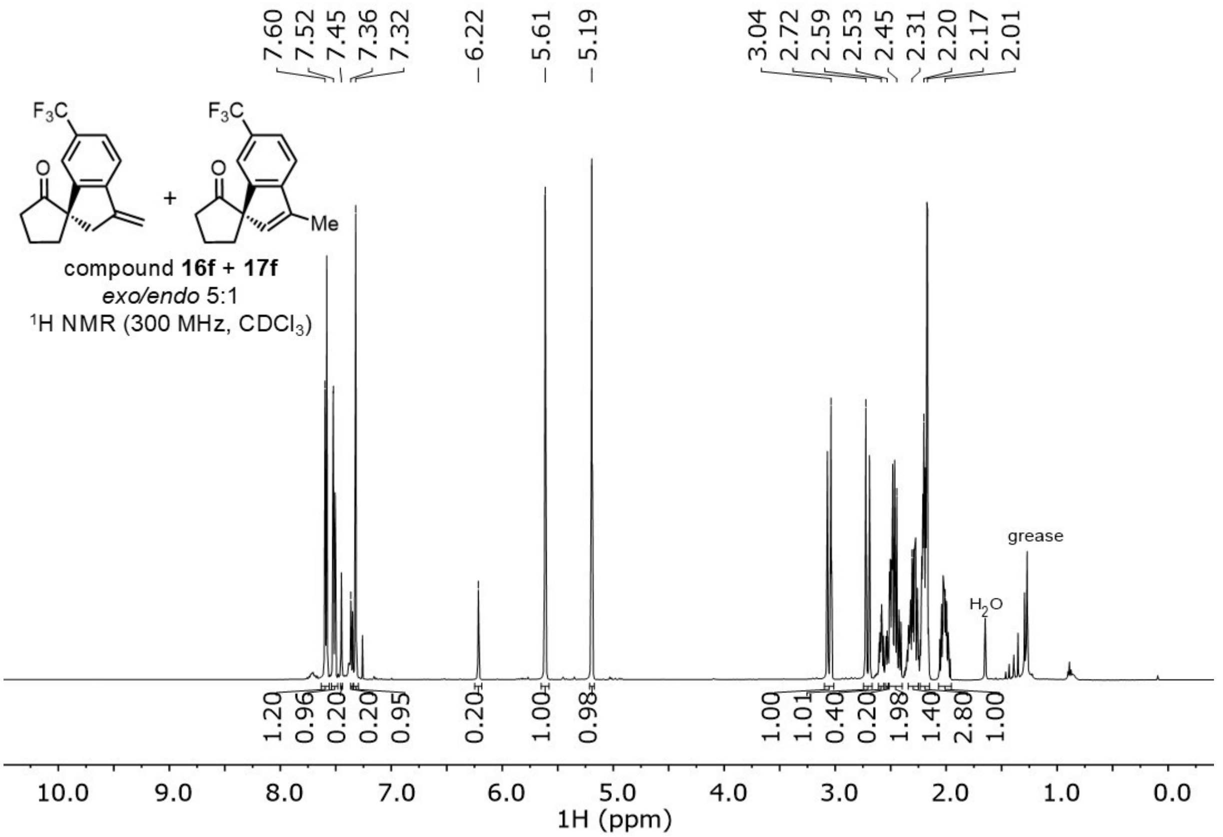
compound **16f** + **17f**
exo/endo 5:1
^{1}H NMR (300 MHz, CDCl3)
7.60 7.52 7.45 7.36 7.32 6.22 5.61 5.19 3.04 2.72 2.59 2.53 2.45 2.31 2.20 2.17 2.01
grease
H2O
1.20 0.96 0.20 0.20 0.95 0.20 1.00 0.98 1.00 1.01 0.40 0.20 1.98 1.40 2.80 1.00
10.0 9.0 8.0 7.0 6.0 5.0 4.0 3.0 2.0 1.0 0.0
1H (ppm)

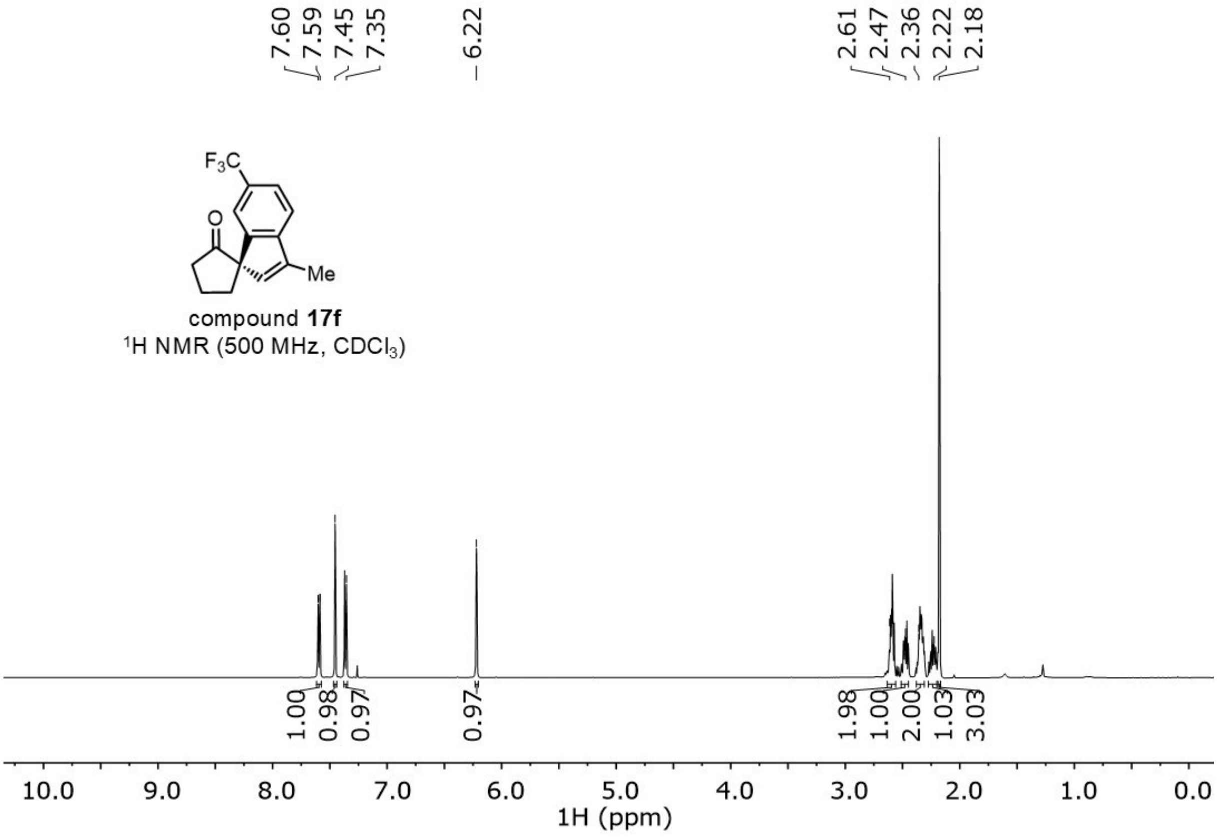
compound **17f**
^{1}H NMR (500 MHz, CDCl3)
7.60 7.59 7.45 7.35 6.22 2.61 2.47 2.36 2.22 2.18
1.00 0.98 0.97 0.97 1.98 1.00 2.00 1.03 3.03
10.0 9.0 8.0 7.0 6.0 5.0 4.0 3.0 2.0 1.0 0.0
1H (ppm)

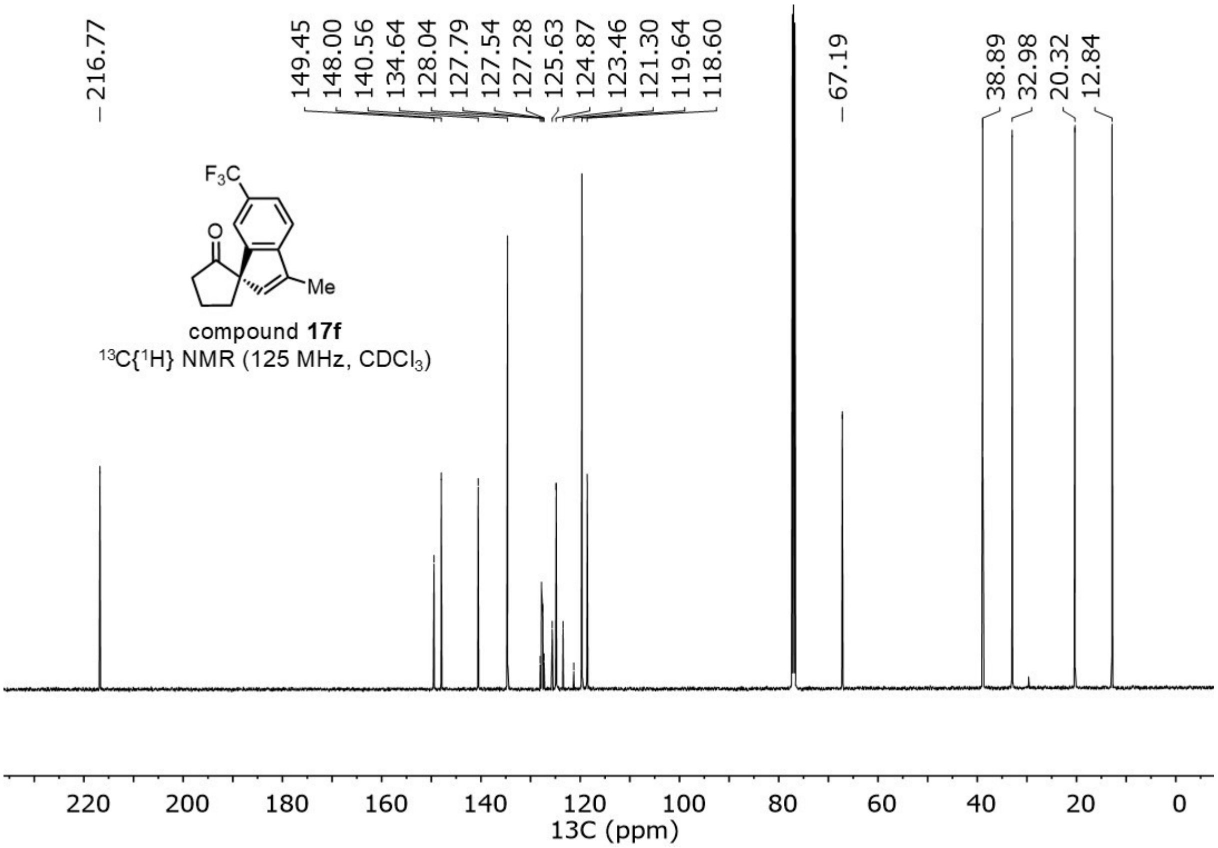
216.77
149.45
148.00
140.56
134.64
128.04
127.79
127.54
127.28
125.63
124.87
123.46
121.30
119.64
118.60
67.19
38.89
32.98
20.32
12.84
F3C
O
Me
compound 17f
13C{1H} NMR (125 MHz, CDCl3)
220 200 180 160 140 120 100 80 60 40 20 0
13C (ppm)

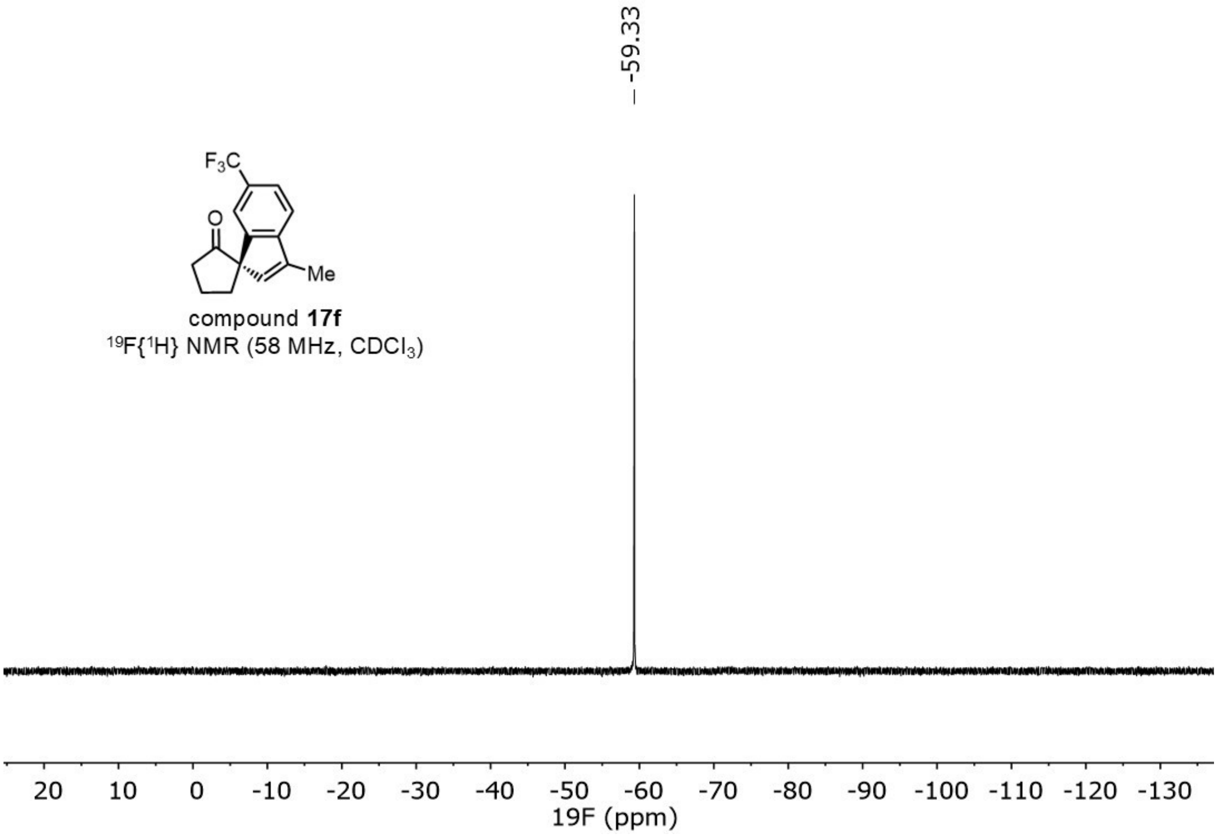
-59.33
F3C
O
Me
compound 17f
19F{1H} NMR (58 MHz, CDCl3)
20 10 0 -10 -20 -30 -40 -50 -60 -70 -80 -90 -100 -110 -120 -130
19F (ppm)

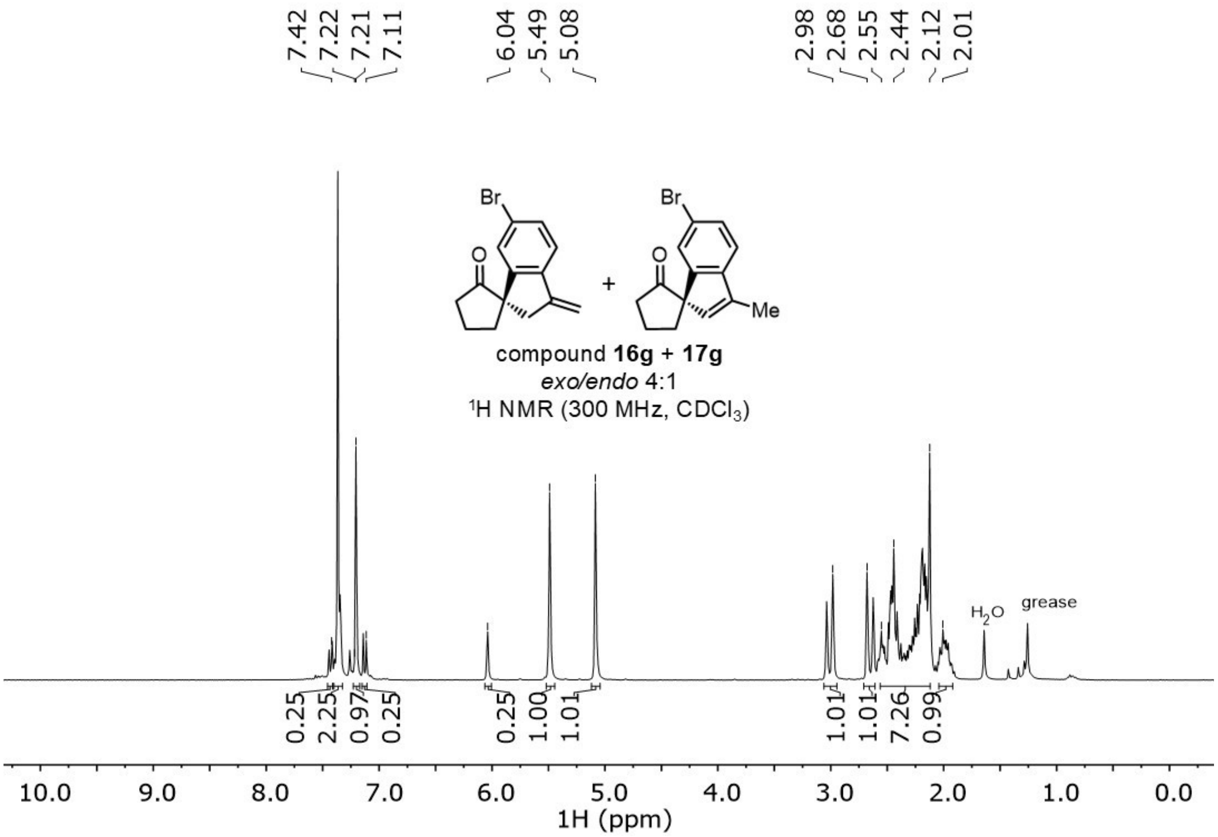
7.42
7.22
7.21
7.11
6.04
5.49
5.08
2.98
2.68
2.55
2.44
2.12
2.01
Br
O
+
Me
compound **16g** + **17g**
exo/endo 4:1
^{1}H NMR (300 MHz, $CDCl_3$)
H_2O
grease
0.25
2.25
0.97
0.25
0.25
1.00
1.01
1.01
1.01
7.26
0.99
10.0
9.0
8.0
7.0
6.0
5.0
4.0
3.0
2.0
1.0
0.0
1H (ppm)

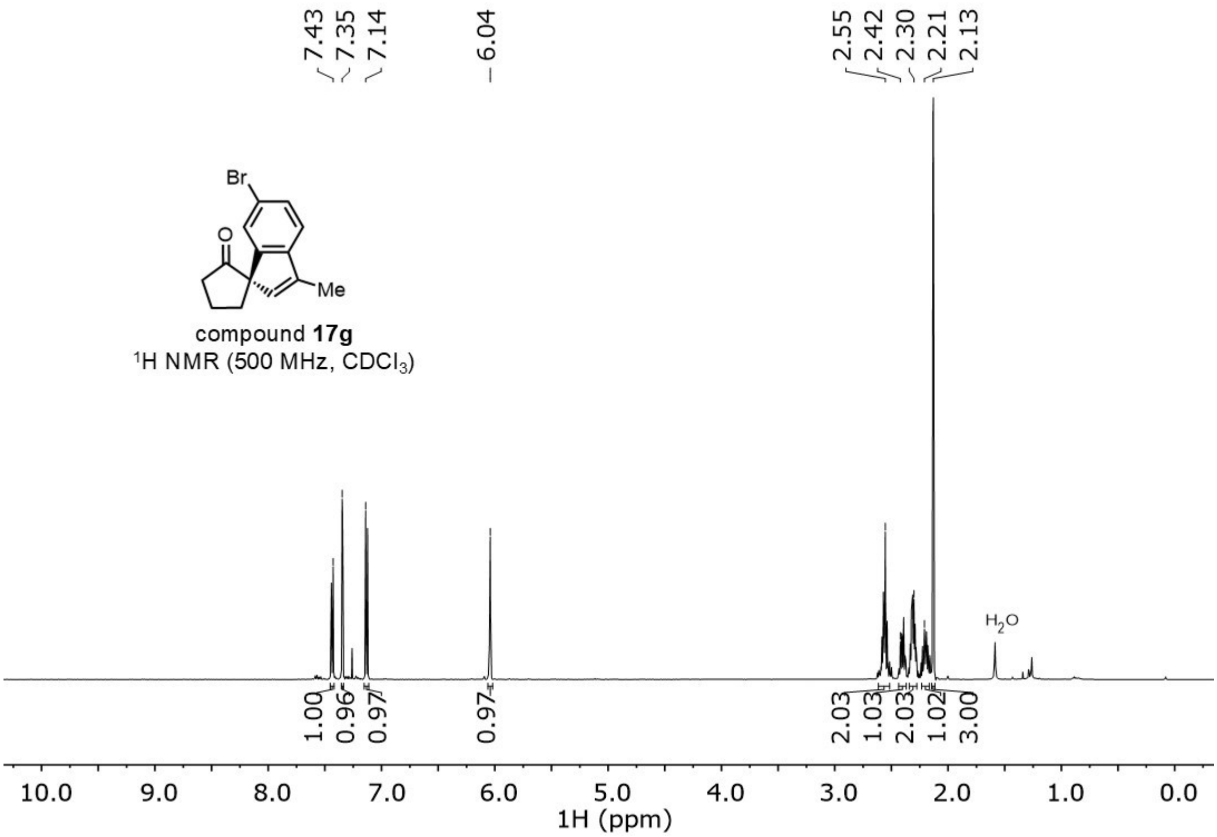
7.43
7.35
7.14
6.04
2.55
2.42
2.30
2.21
2.13
Br
O
Me
compound **17g**
1H NMR (500 MHz, CDCl3)
H2O
1.00
0.96
0.97
0.97
2.03
1.03
2.03
1.02
3.00
10.0
9.0
8.0
7.0
6.0
5.0
4.0
3.0
2.0
1.0
0.0
1H (ppm)

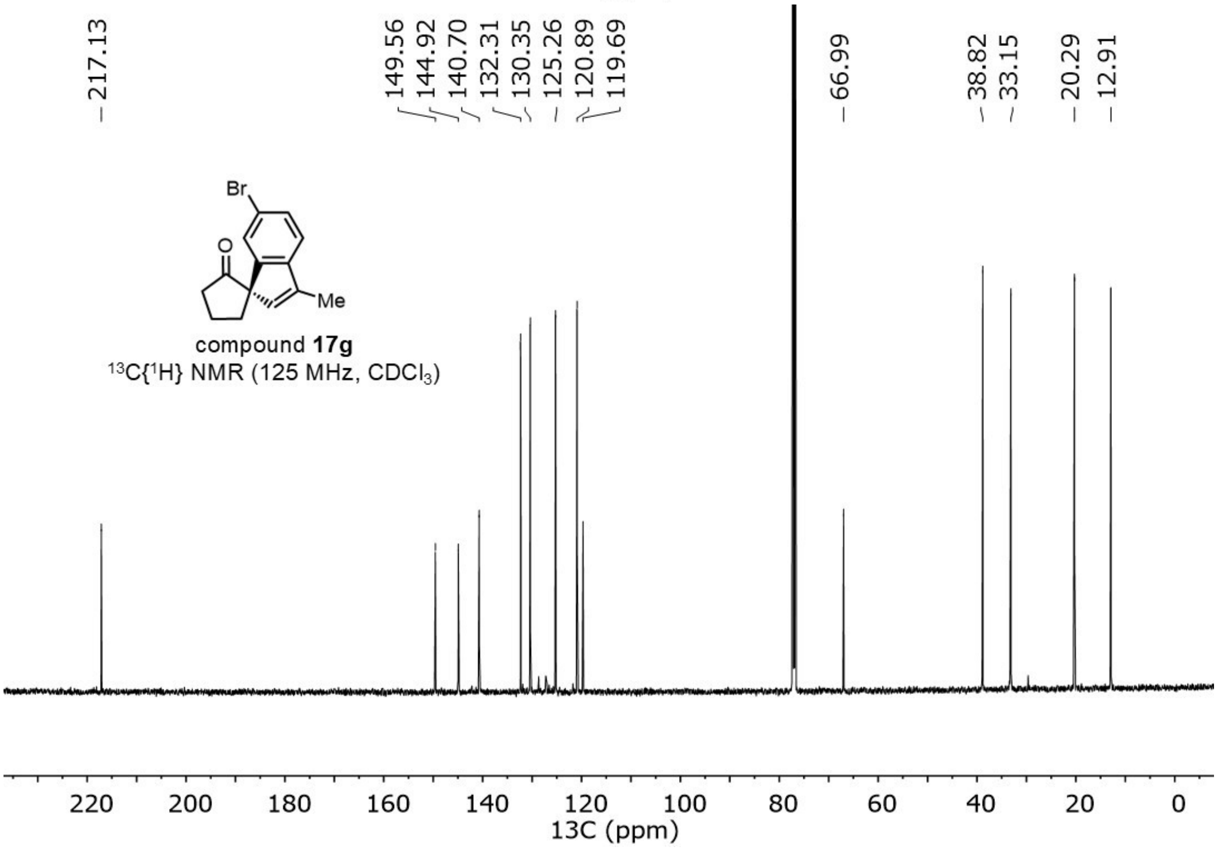
217.13
149.56
144.92
140.70
132.31
130.35
125.26
120.89
119.69
66.99
38.82
33.15
20.29
12.91
Br
O
Me
compound **17g**
13C{1H} NMR (125 MHz, CDCl3)
220
200
180
160
140
120
100
80
60
40
20
0
13C (ppm)

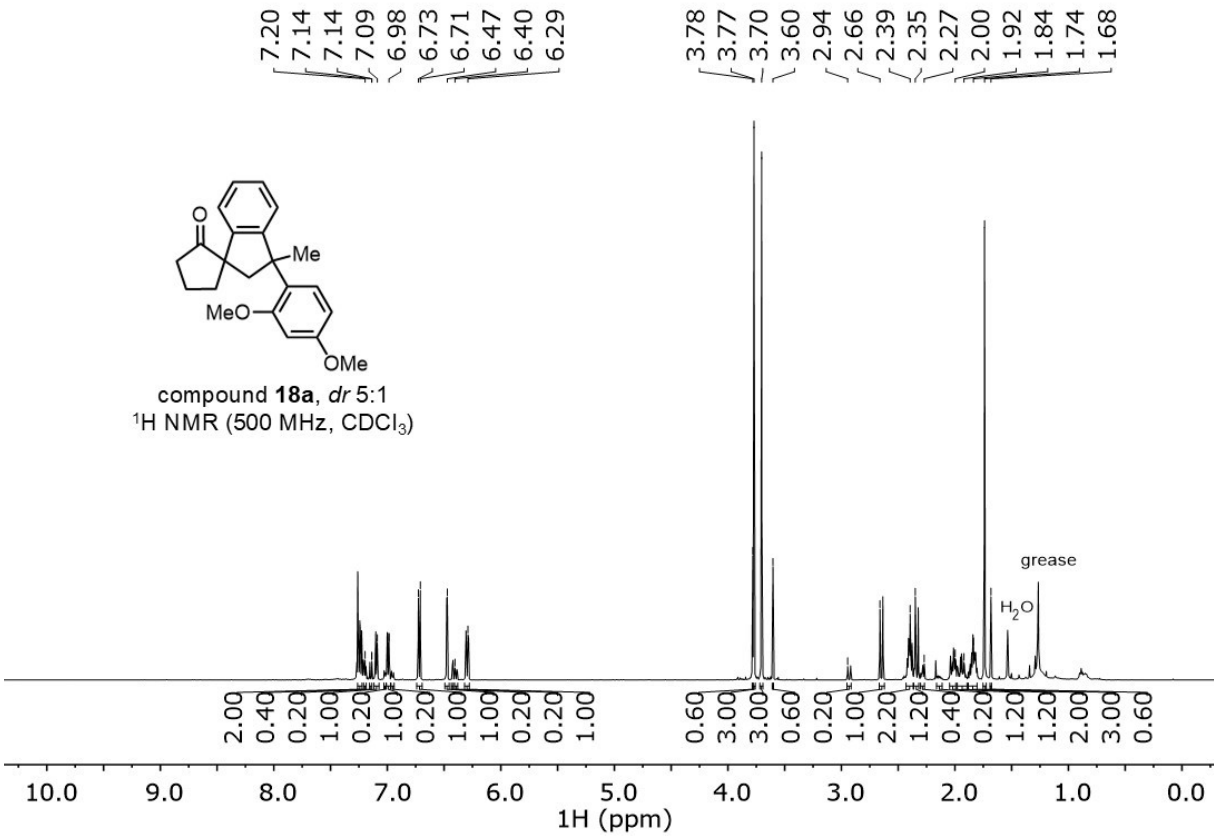
7.20 7.14 7.14 7.09 6.98 6.73 6.71 6.47 6.40 6.29
3.78 3.77 3.70 3.60 2.94 2.66 2.39 2.35 2.27 2.00 1.92 1.84 1.74 1.68
O
Me
MeO
OMe
compound **18a**, *dr* 5:1
^{1}H NMR (500 MHz, $CDCl_3$)
grease
H_2O
2.00 0.40 0.20 1.00 0.20 1.00 0.20 1.00 1.00 0.20 0.20 1.00
0.60 3.00 3.00 0.60 0.20 1.00 2.20 1.20 0.40 0.20 1.20 1.20 2.00 3.00 0.60
10.0 9.0 8.0 7.0 6.0 5.0 4.0 3.0 2.0 1.0 0.0
1H (ppm)

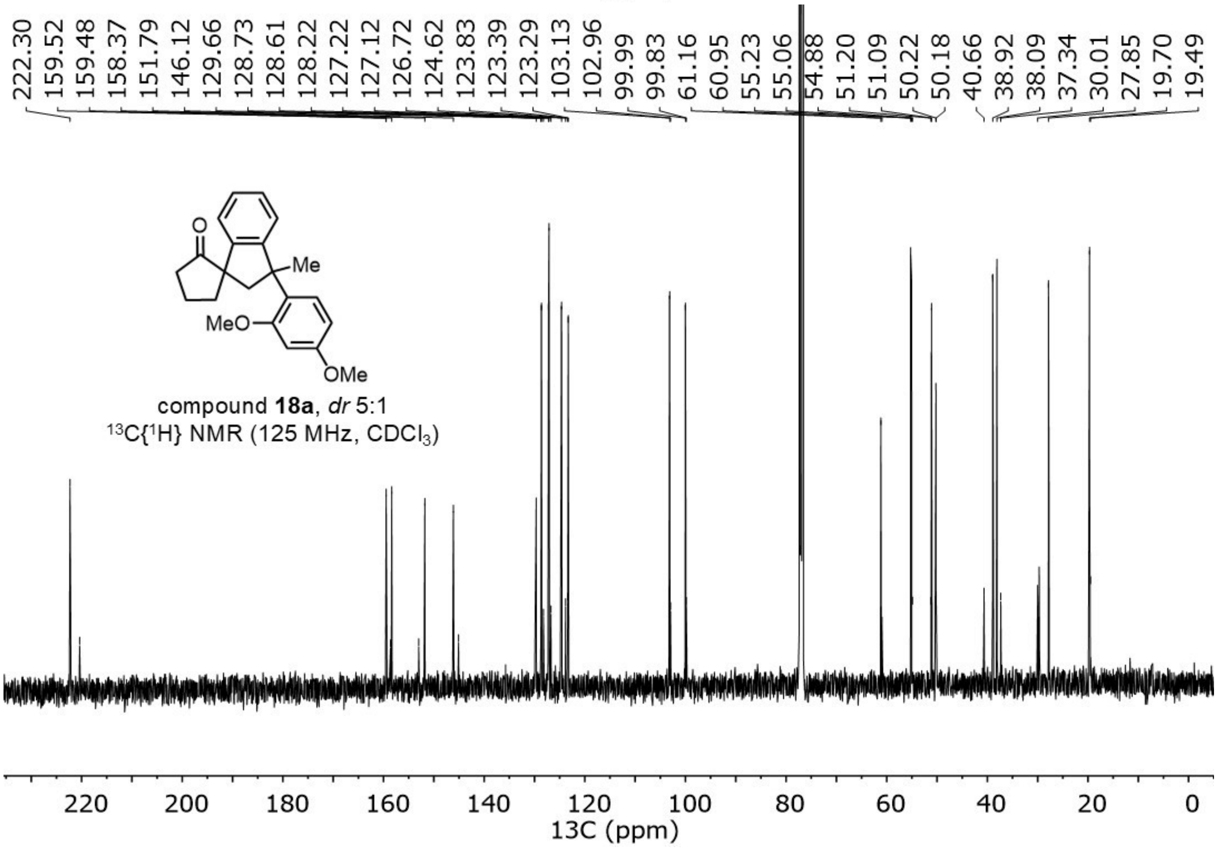
222.30 159.52 159.48 158.37 151.79 146.12 129.66 128.73 128.61 128.22 127.22 127.12 126.72 124.62 123.83 123.39 123.29 103.13 102.96 99.99 99.83 61.16 60.95 55.23 55.06 54.88 51.20 51.09 50.22 50.18 40.66 38.92 38.09 37.34 30.01 27.85 19.70 19.49
O
Me
MeO
OMe
compound **18a**, *dr* 5:1
^{13}C{^{1}H} NMR (125 MHz, $CDCl_3$)
220 200 180 160 140 120 100 80 60 40 20 0
13C (ppm)

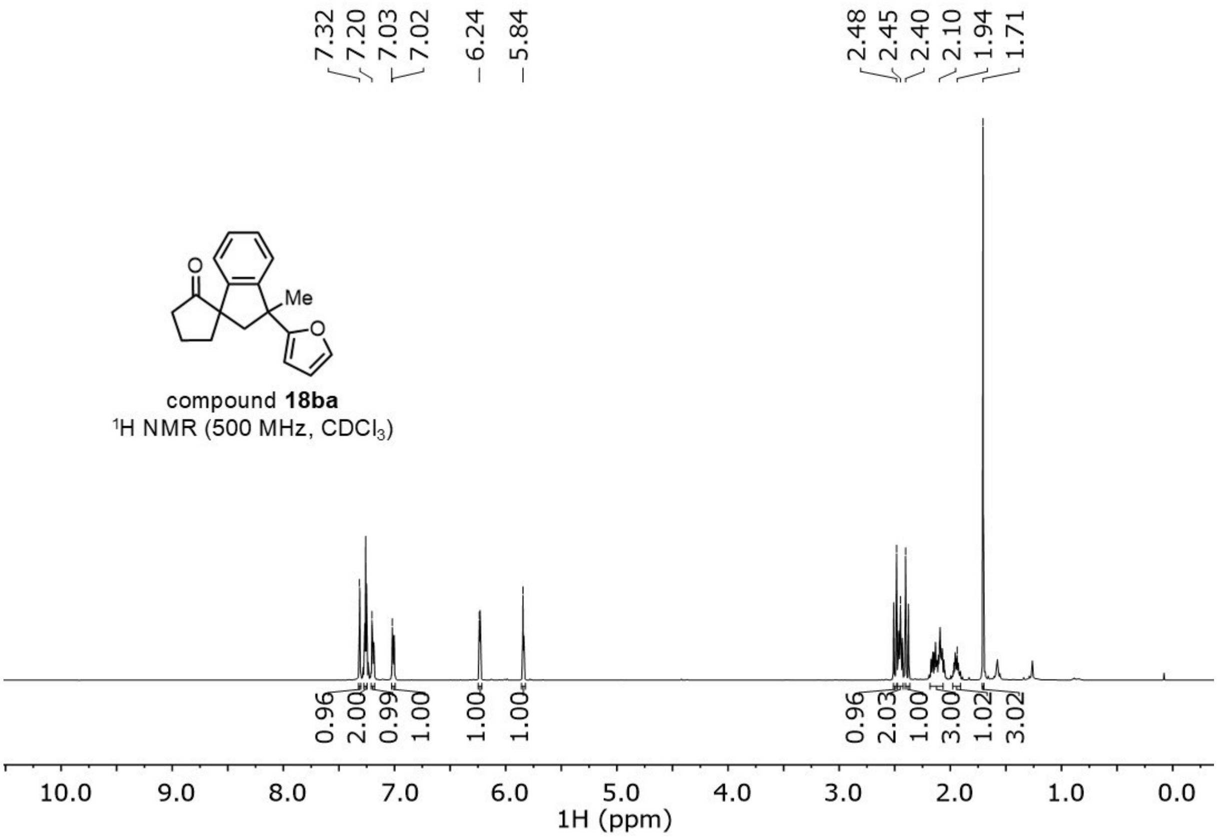
7.32
7.20
7.03
7.02
6.24
5.84
2.48
2.45
2.40
2.10
1.94
1.71
O
Me
O
compound **18ba**
^{1}H NMR (500 MHz, $CDCl_3$)
0.96
2.00
0.99
1.00
1.00
1.00
0.96
2.03
1.00
3.00
1.02
3.02
10.0
9.0
8.0
7.0
6.0
5.0
4.0
3.0
2.0
1.0
0.0
1H (ppm)

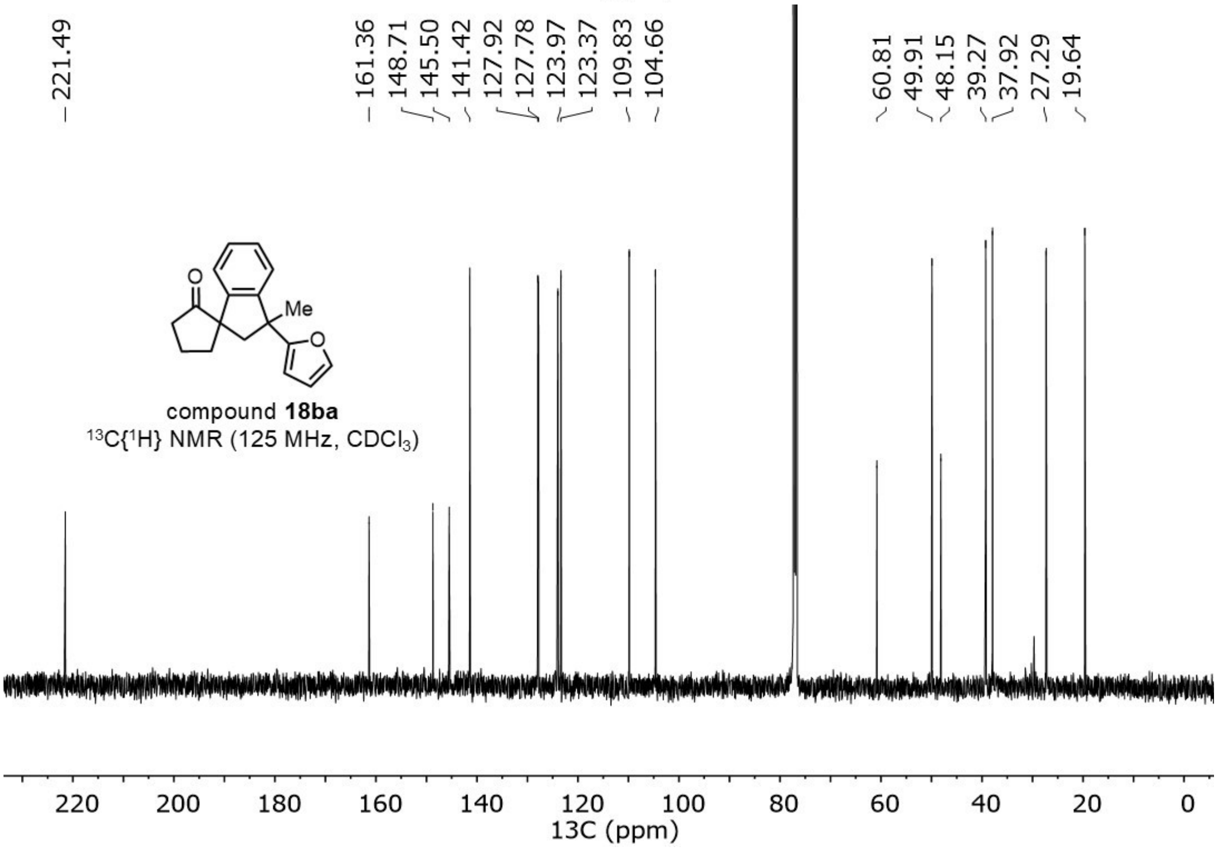
221.49
161.36
148.71
145.50
141.42
127.92
127.78
123.97
123.37
109.83
104.66
60.81
49.91
48.15
39.27
37.92
27.29
19.64
O
Me
O
compound **18ba**
$^{13}C\{^1H\}$ NMR (125 MHz, $CDCl_3$)
220
200
180
160
140
120
100
80
60
40
20
0
13C (ppm)

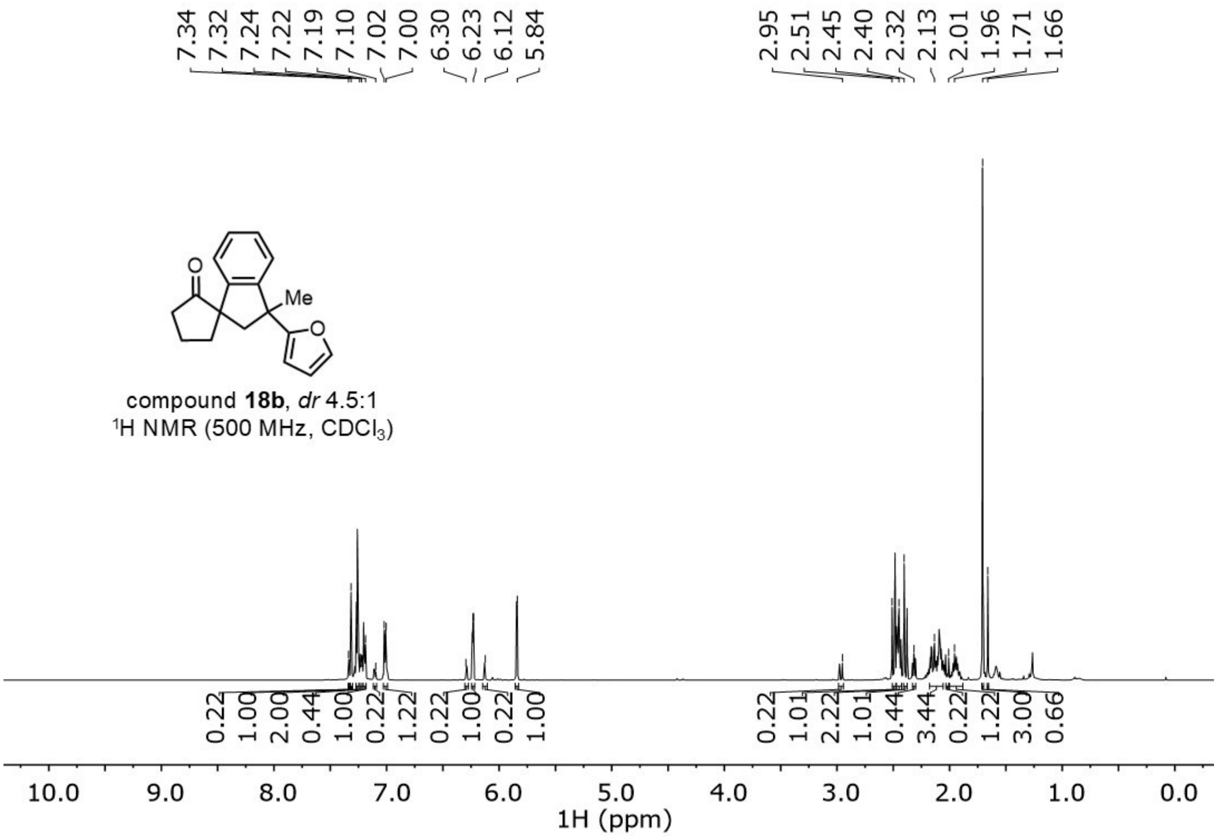
7.34
7.32
7.24
7.22
7.19
7.10
7.02
7.00
6.30
6.23
6.12
5.84
2.95
2.51
2.45
2.40
2.32
2.13
2.01
1.96
1.71
1.66
O
Me
O
compound 18b, dr 4.5:1
1H NMR (500 MHz, CDCl3)
0.22
1.00
2.00
0.44
1.00
0.22
1.22
0.22
1.00
0.22
1.00
0.22
1.01
2.22
1.01
0.44
3.44
0.22
1.22
3.00
0.66
10.0
9.0
8.0
7.0
6.0
5.0
4.0
3.0
2.0
1.0
0.0
1H (ppm)

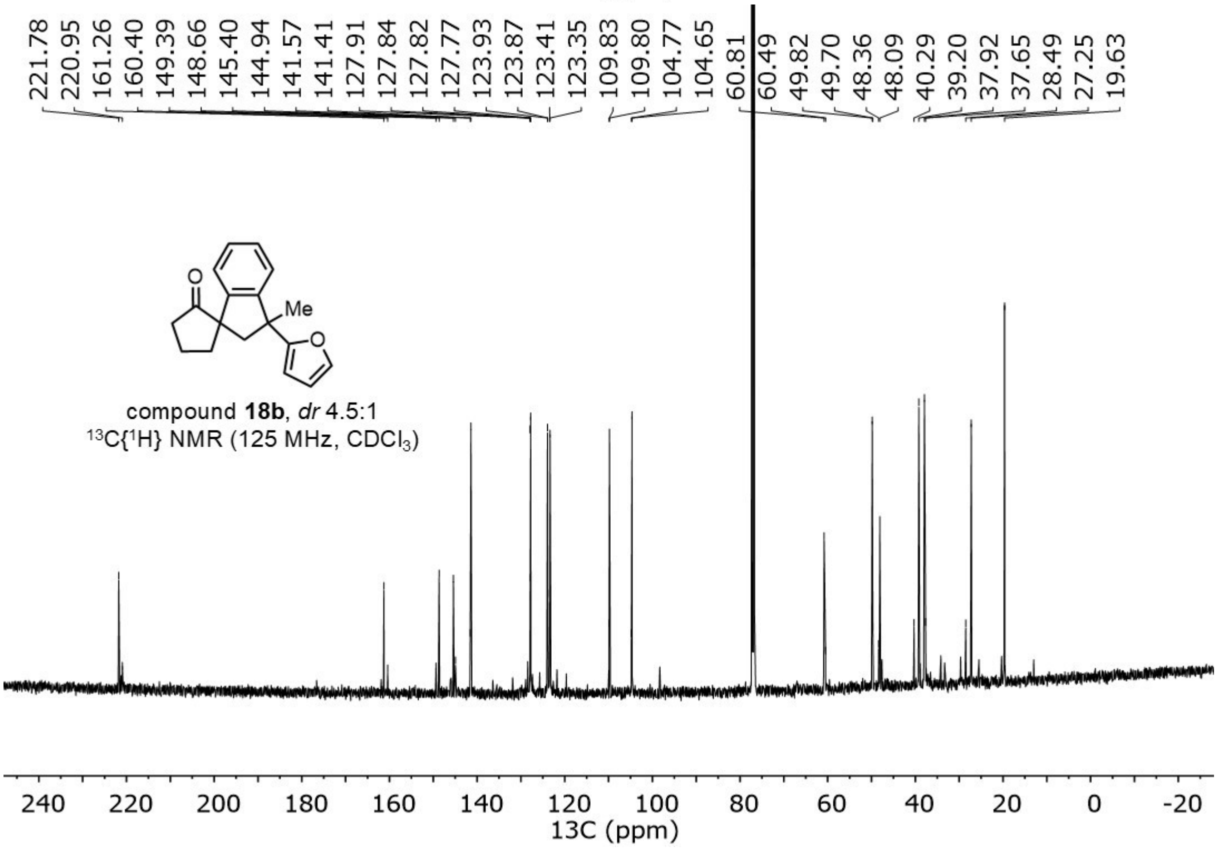
221.78
220.95
161.26
160.40
149.39
148.66
145.40
144.94
141.57
141.41
127.91
127.84
127.82
127.77
123.93
123.87
123.41
123.35
109.83
109.80
104.77
104.65
60.81
60.49
49.82
49.70
48.36
48.09
40.29
39.20
37.92
37.65
28.49
27.25
19.63
O
Me
O
compound 18b, dr 4.5:1
13C{1H} NMR (125 MHz, CDCl3)
240
220
200
180
160
140
120
100
80
60
40
20
0
-20
13C (ppm)

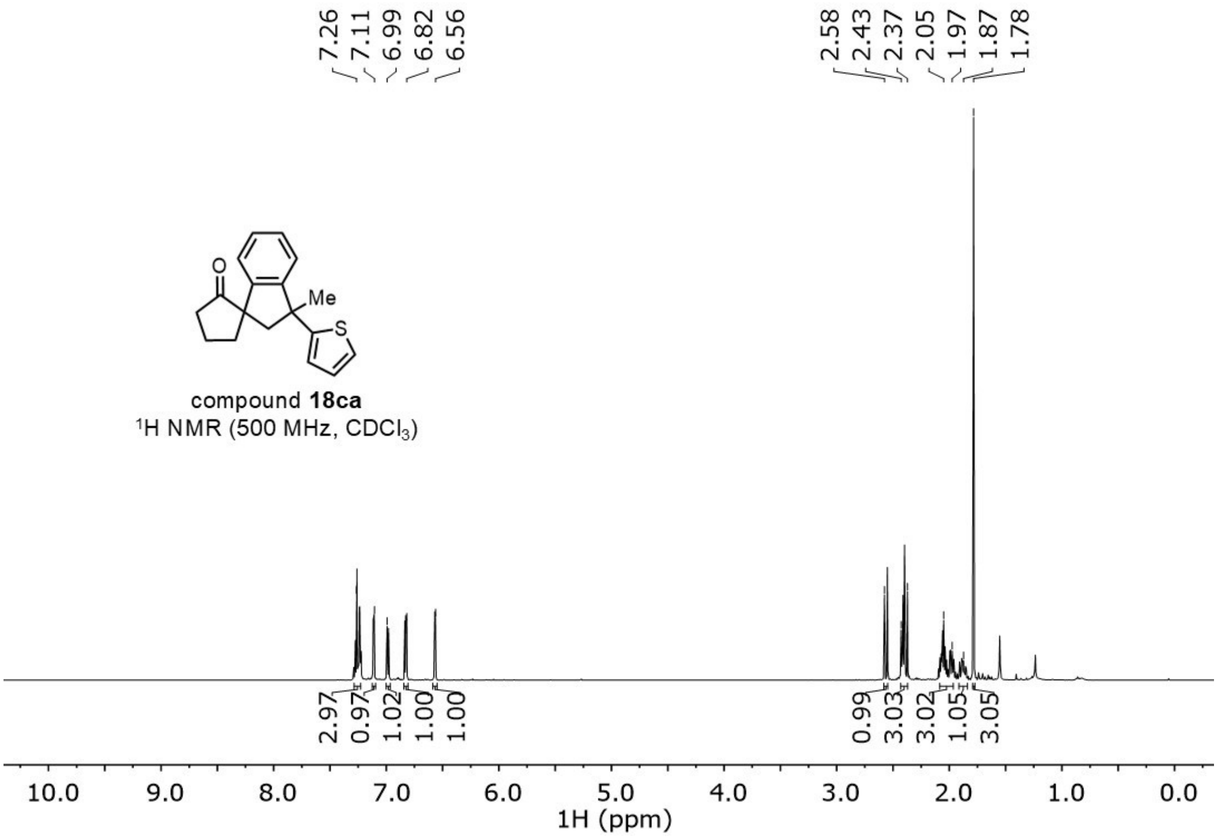
7.26
7.11
6.99
6.82
6.56
2.58
2.43
2.37
2.05
1.97
1.87
1.78
O
Me
S
compound **18ca**
^{1}H NMR (500 MHz, $CDCl_3$)
2.97
0.97
1.02
1.00
1.00
0.99
3.03
3.02
1.05
3.05
10.0
9.0
8.0
7.0
6.0
5.0
4.0
3.0
2.0
1.0
0.0
1H (ppm)

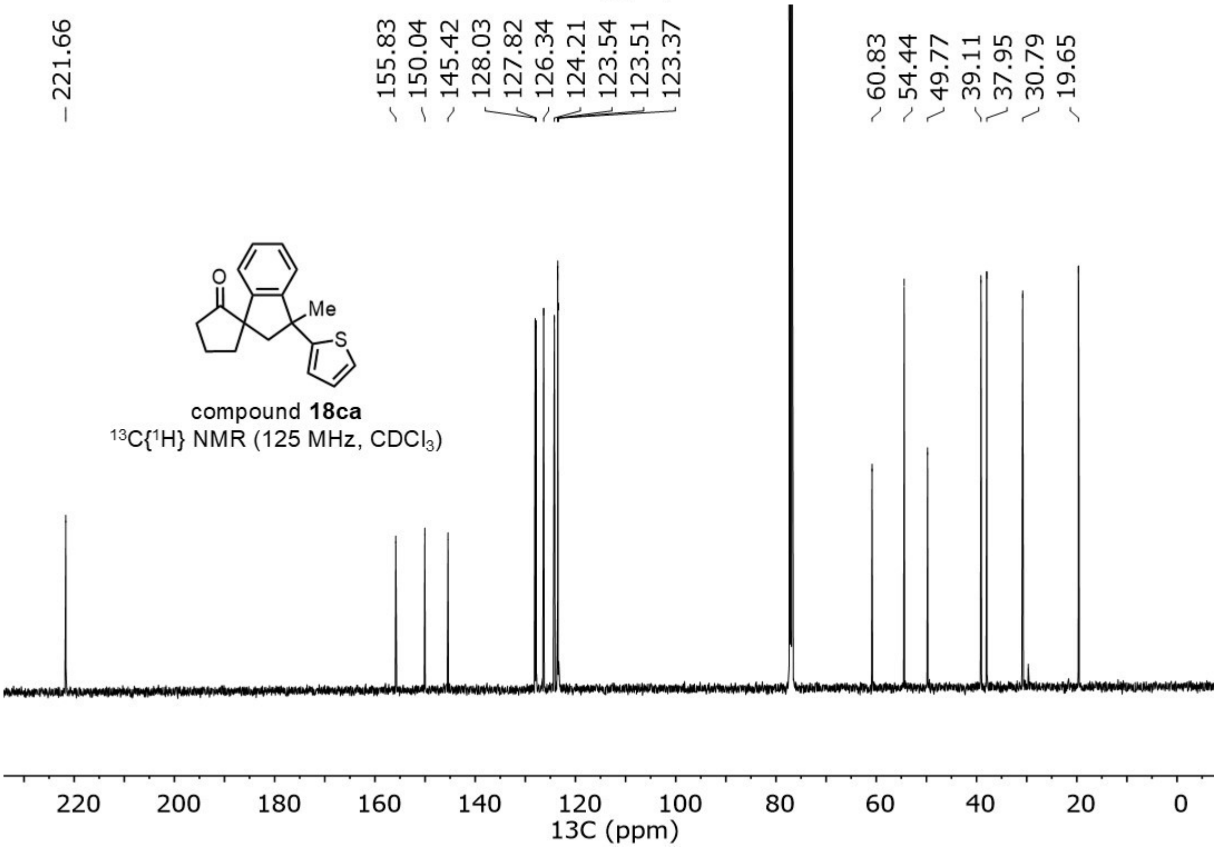
221.66
155.83
150.04
145.42
128.03
127.82
126.34
124.21
123.54
123.51
123.37
60.83
54.44
49.77
39.11
37.95
30.79
19.65
O
Me
S
compound **18ca**
$^{13}C\{^{1}H\}$ NMR (125 MHz, $CDCl_3$)
220
200
180
160
140
120
100
80
60
40
20
0
13C (ppm)

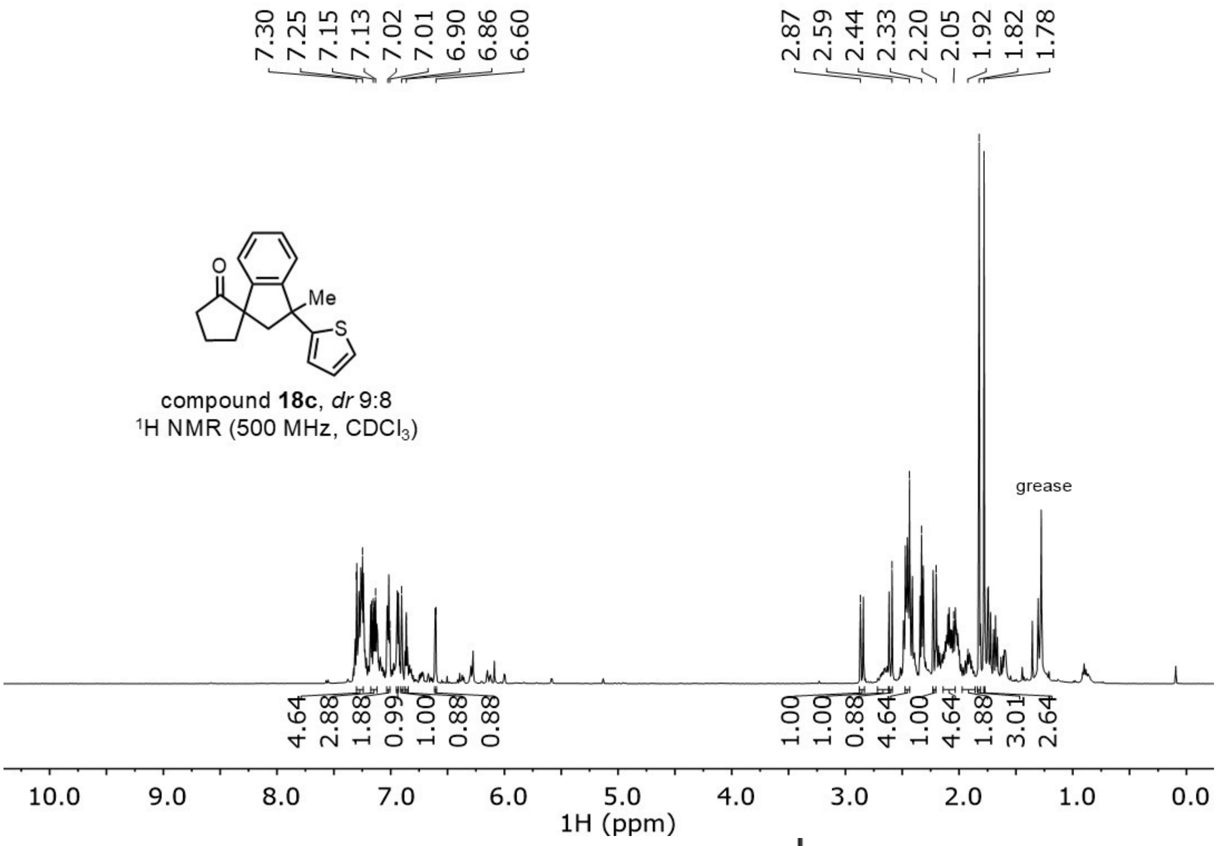

7.30 7.25 7.15 7.13 7.02 7.01 6.90 6.86 6.60
2.87 2.59 2.44 2.33 2.20 2.05 1.92 1.82 1.78
O
Me
S
compound **18c**, *dr* 9:8
^{1}H NMR (500 MHz, $CDCl_3$)
grease
4.64 2.88 1.88 0.99 1.00 0.88 0.88
1.00 1.00 0.88 4.64 1.00 4.64 1.88 3.01 2.64
10.0 9.0 8.0 7.0 6.0 5.0 4.0 3.0 2.0 1.0 0.0
1H (ppm)

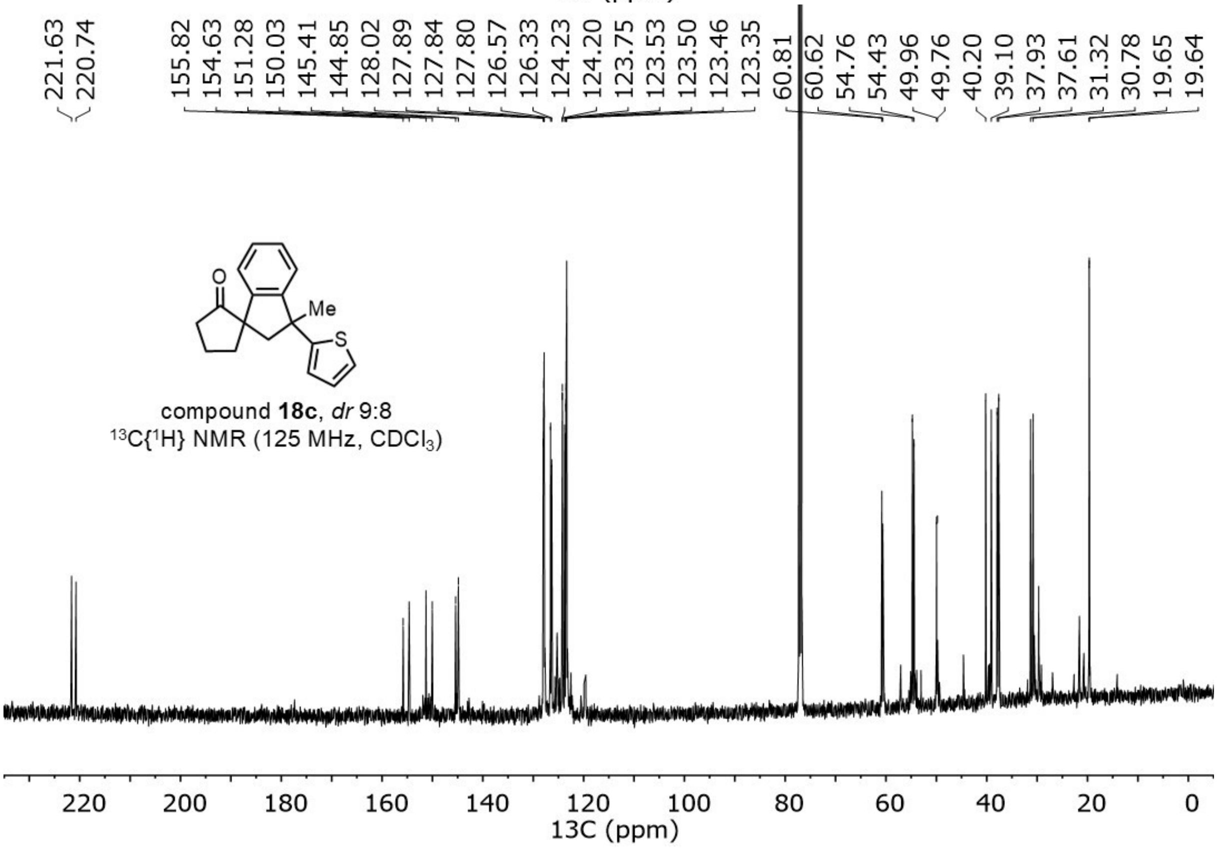

221.63 220.74
155.82 154.63 151.28 150.03 145.41 144.85 128.02 127.89 127.84 127.80 126.57 126.33 124.23 124.20 123.75 123.53 123.50 123.46 123.35
60.81 60.62 54.76 54.43 49.96 49.76 40.20 39.10 37.93 37.61 31.32 30.78 19.65 19.64
O
Me
S
compound **18c**, *dr* 9:8
^{13}C{^{1}H} NMR (125 MHz, $CDCl_3$)
220 200 180 160 140 120 100 80 60 40 20 0
13C (ppm)

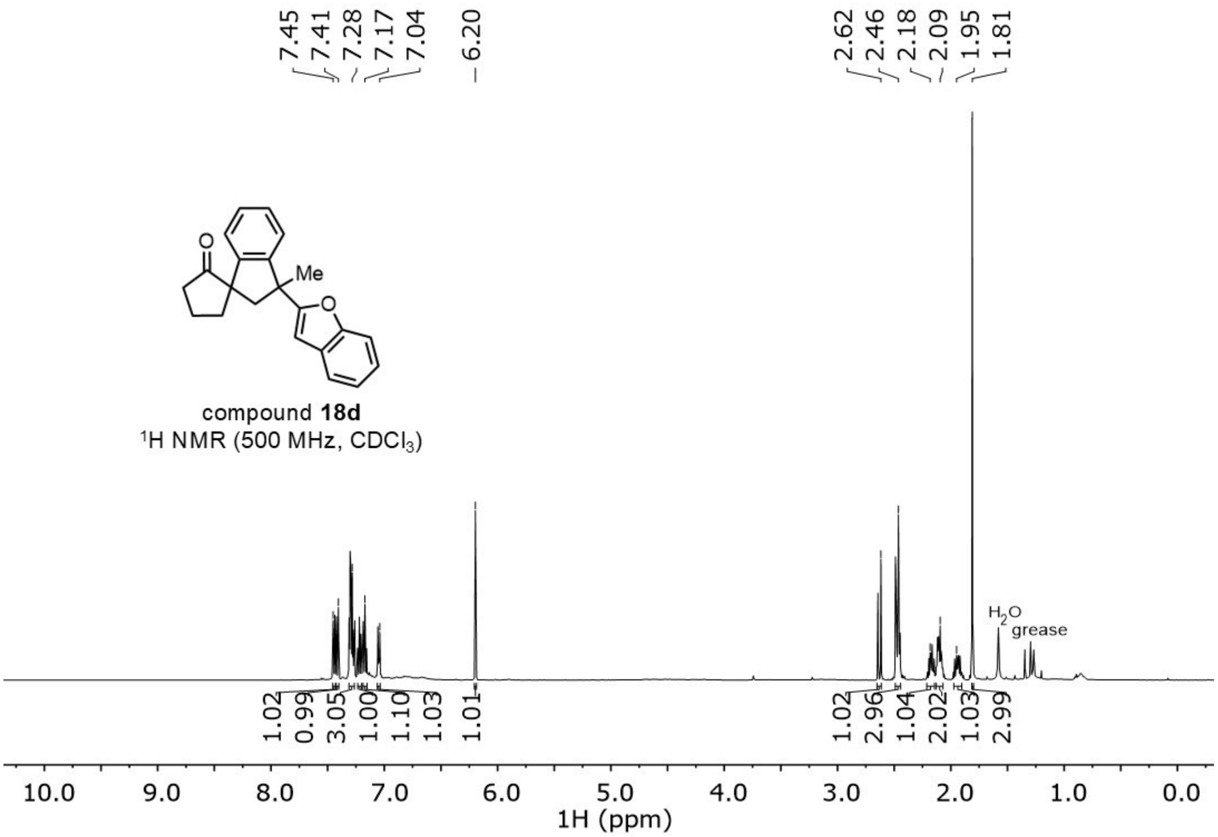
7.45
7.41
7.28
7.17
7.04
6.20
2.62
2.46
2.18
2.09
1.95
1.81
O
Me
O
compound **18d**
^{1}H NMR (500 MHz, $CDCl_3$)
H_2O
grease
1.02
0.99
3.05
1.00
1.10
1.03
1.01
1.02
2.96
1.04
2.02
1.03
2.99
10.0
9.0
8.0
7.0
6.0
5.0
4.0
3.0
2.0
1.0
0.0
1H (ppm)

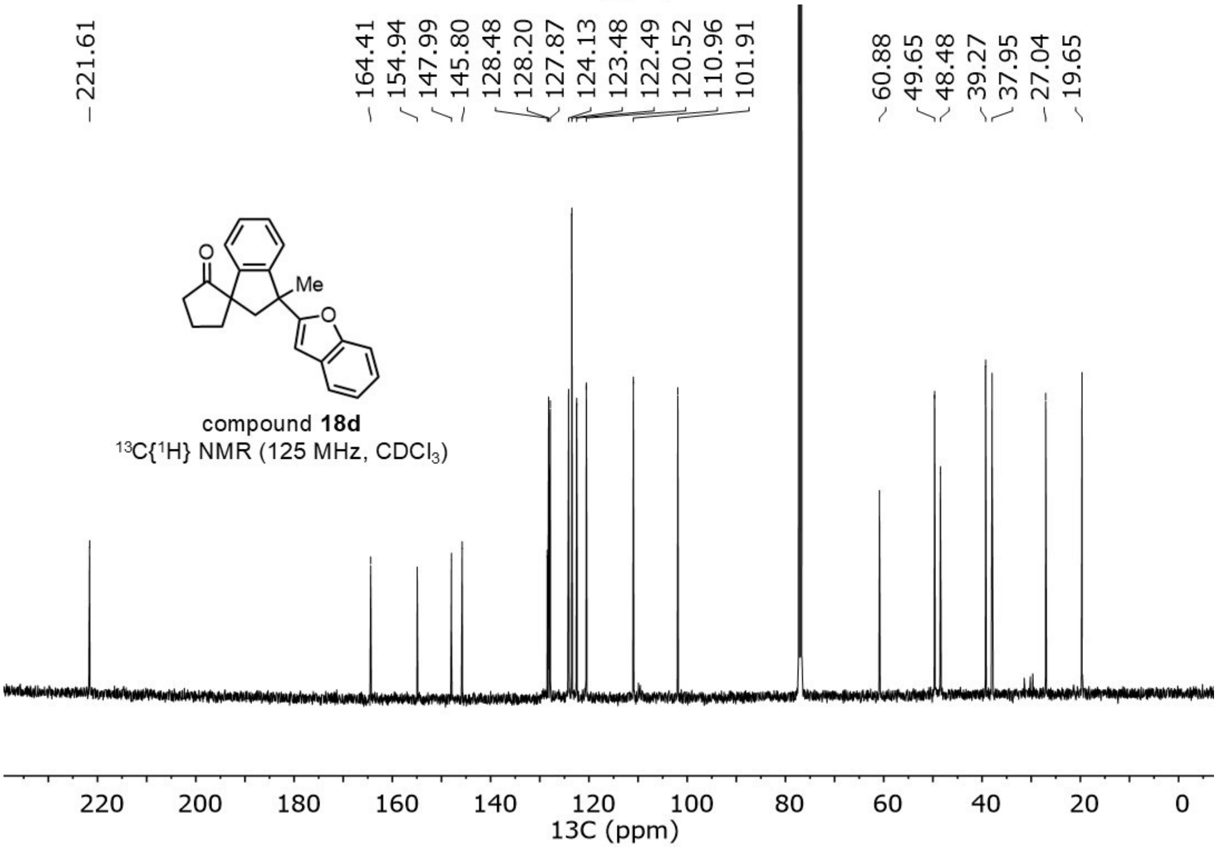
221.61
164.41
154.94
147.99
145.80
128.48
128.20
127.87
124.13
123.48
122.49
120.52
110.96
101.91
60.88
49.65
48.48
39.27
37.95
27.04
19.65
O
Me
O
compound **18d**
$^{13}C\{^{1}H\}$ NMR (125 MHz, $CDCl_3$)
220
200
180
160
140
120
100
80
60
40
20
0
13C (ppm)

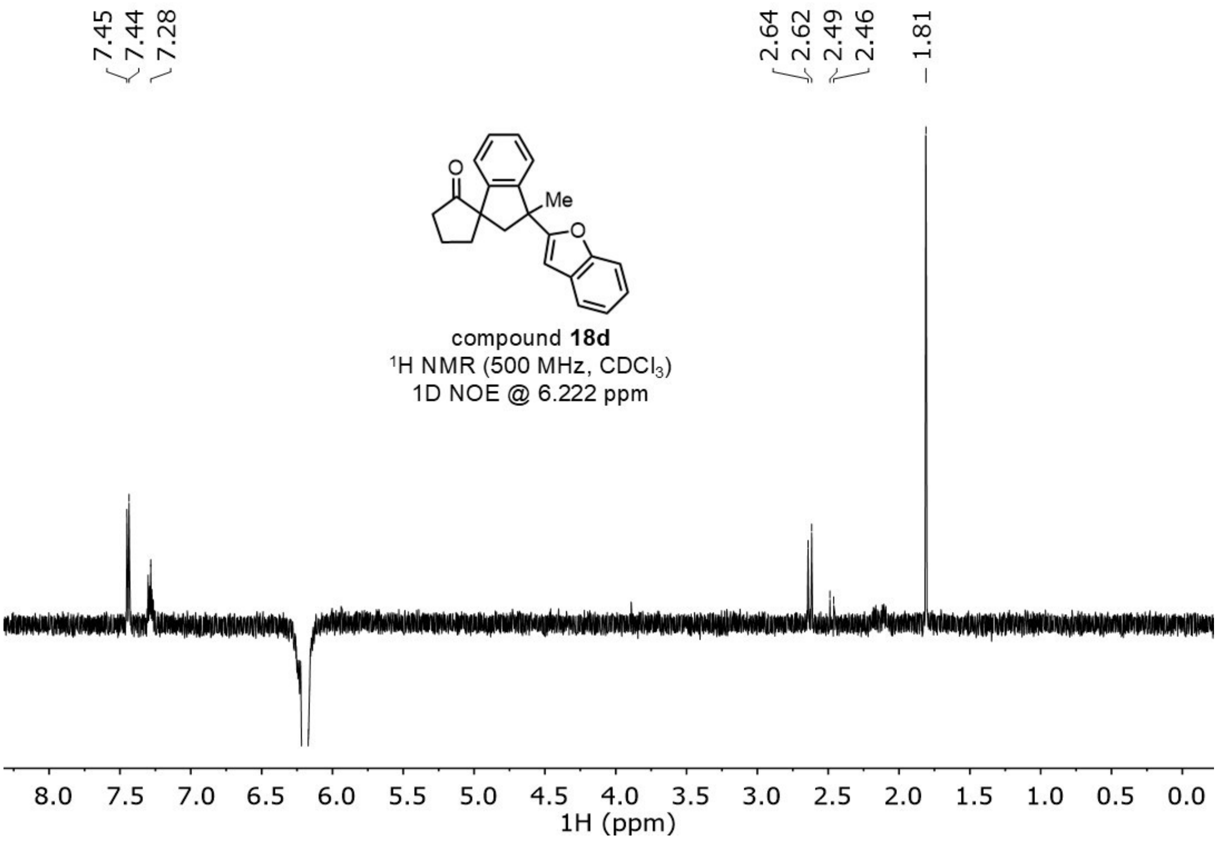
7.45
7.44
7.28
2.64
2.62
2.49
2.46
1.81
Me
O
O
compound 18d
1H NMR (500 MHz, CDCl3)
1D NOE @ 6.222 ppm
8.0 7.5 7.0 6.5 6.0 5.5 5.0 4.5 4.0 3.5 3.0 2.5 2.0 1.5 1.0 0.5 0.0
1H (ppm)

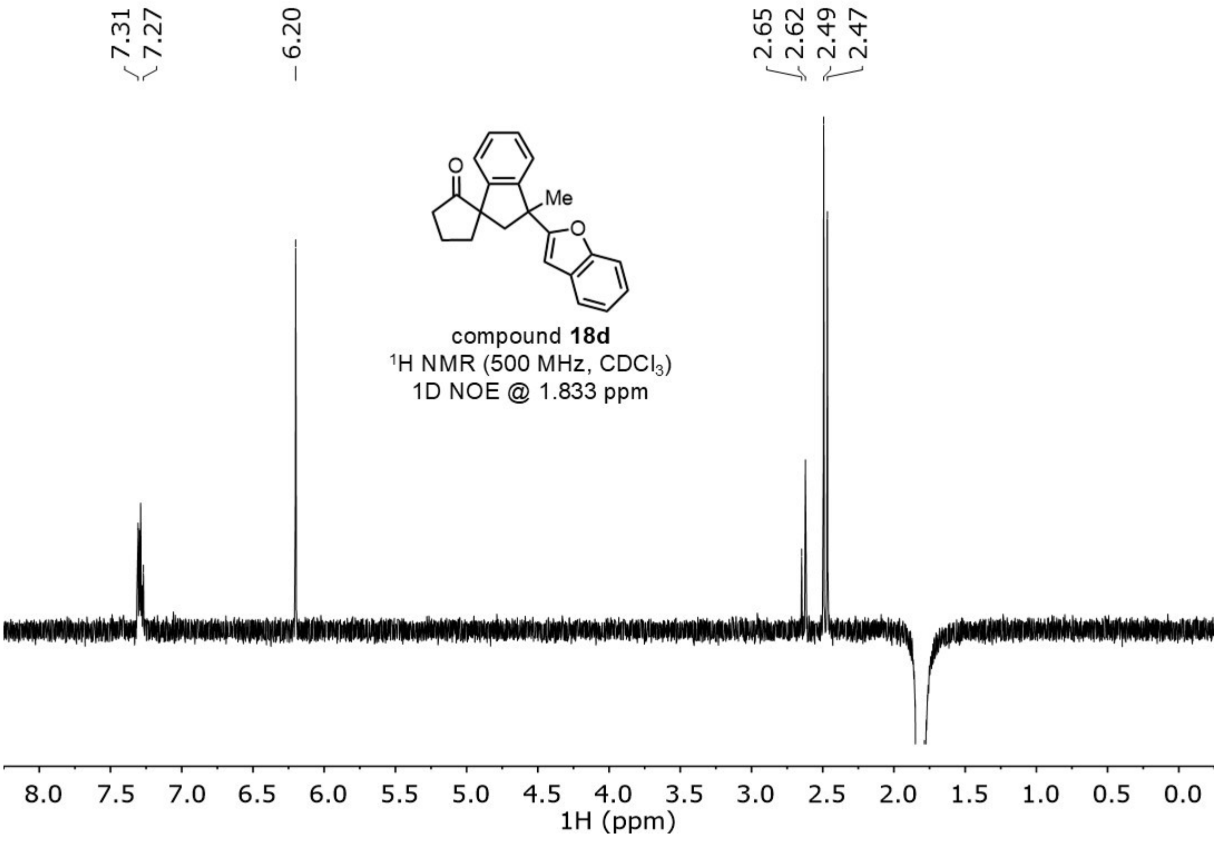
7.31
7.27
6.20
2.65
2.62
2.49
2.47
Me
O
O
compound 18d
1H NMR (500 MHz, CDCl3)
1D NOE @ 1.833 ppm
8.0 7.5 7.0 6.5 6.0 5.5 5.0 4.5 4.0 3.5 3.0 2.5 2.0 1.5 1.0 0.5 0.0
1H (ppm)

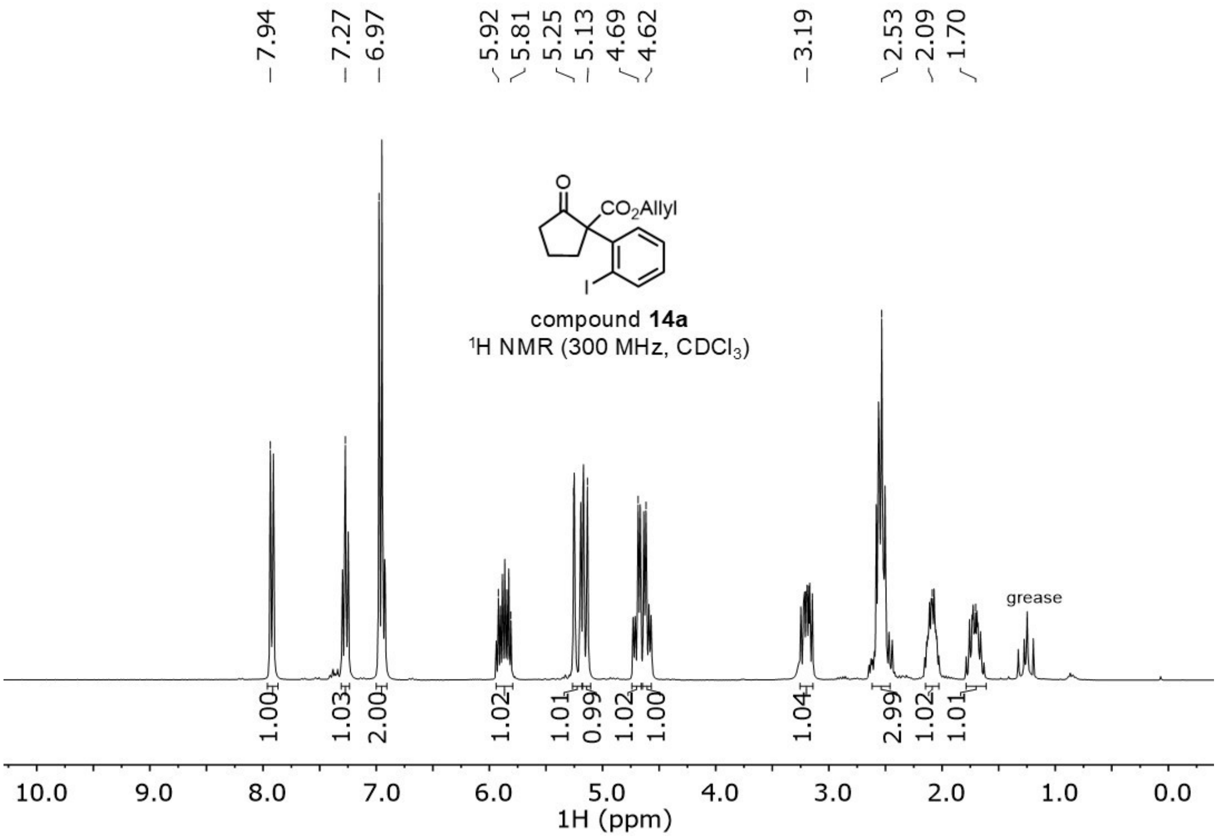
7.94
7.27
6.97
5.92
5.81
5.25
5.13
4.69
4.62
3.19
2.53
2.09
1.70
CO2Allyl
I
compound 14a
1H NMR (300 MHz, CDCl3)
grease
1.00
1.03
2.00
1.02
1.01
0.99
1.02
1.00
1.04
2.99
1.02
1.01
10.0
9.0
8.0
7.0
6.0
5.0
4.0
3.0
2.0
1.0
0.0
1H (ppm)

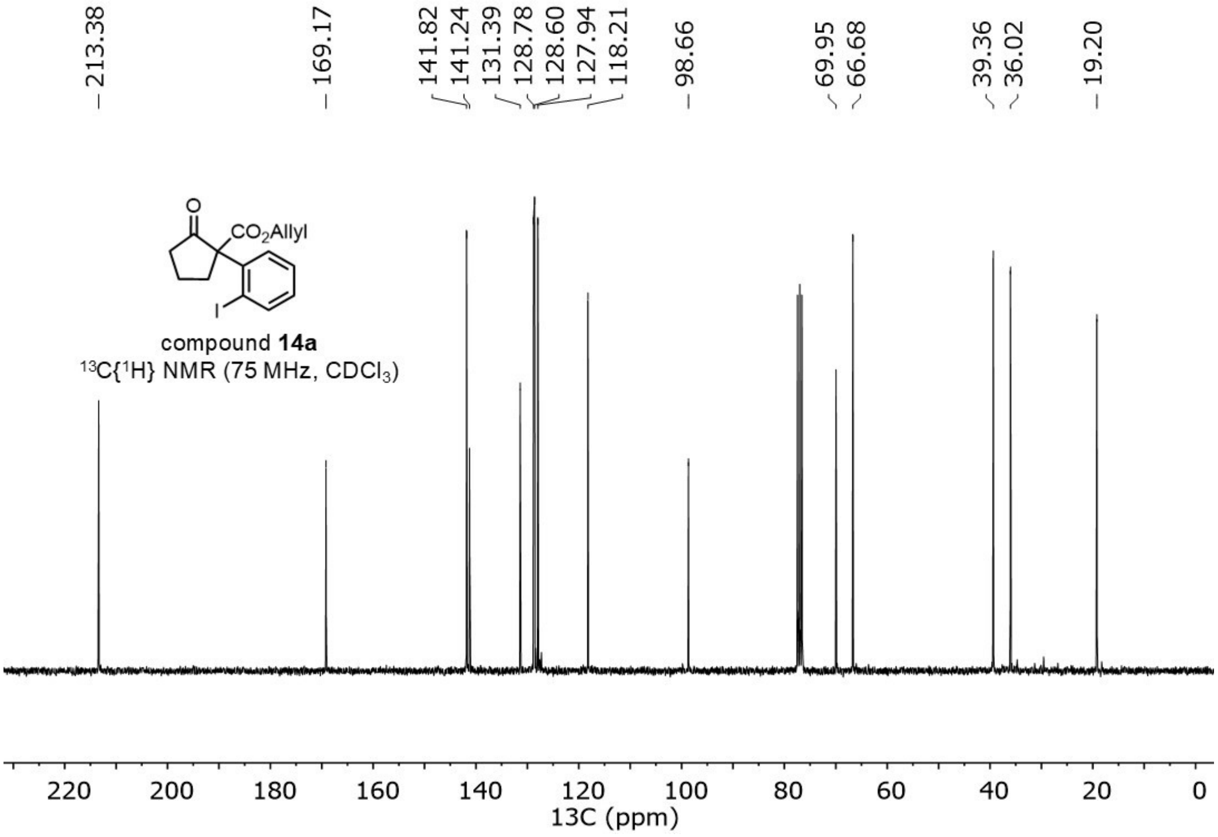
213.38
169.17
141.82
141.24
131.39
128.78
128.60
127.94
118.21
98.66
69.95
66.68
39.36
36.02
19.20
CO2Allyl
I
compound 14a
13C{1H} NMR (75 MHz, CDCl3)
220
200
180
160
140
120
100
80
60
40
20
0
13C (ppm)

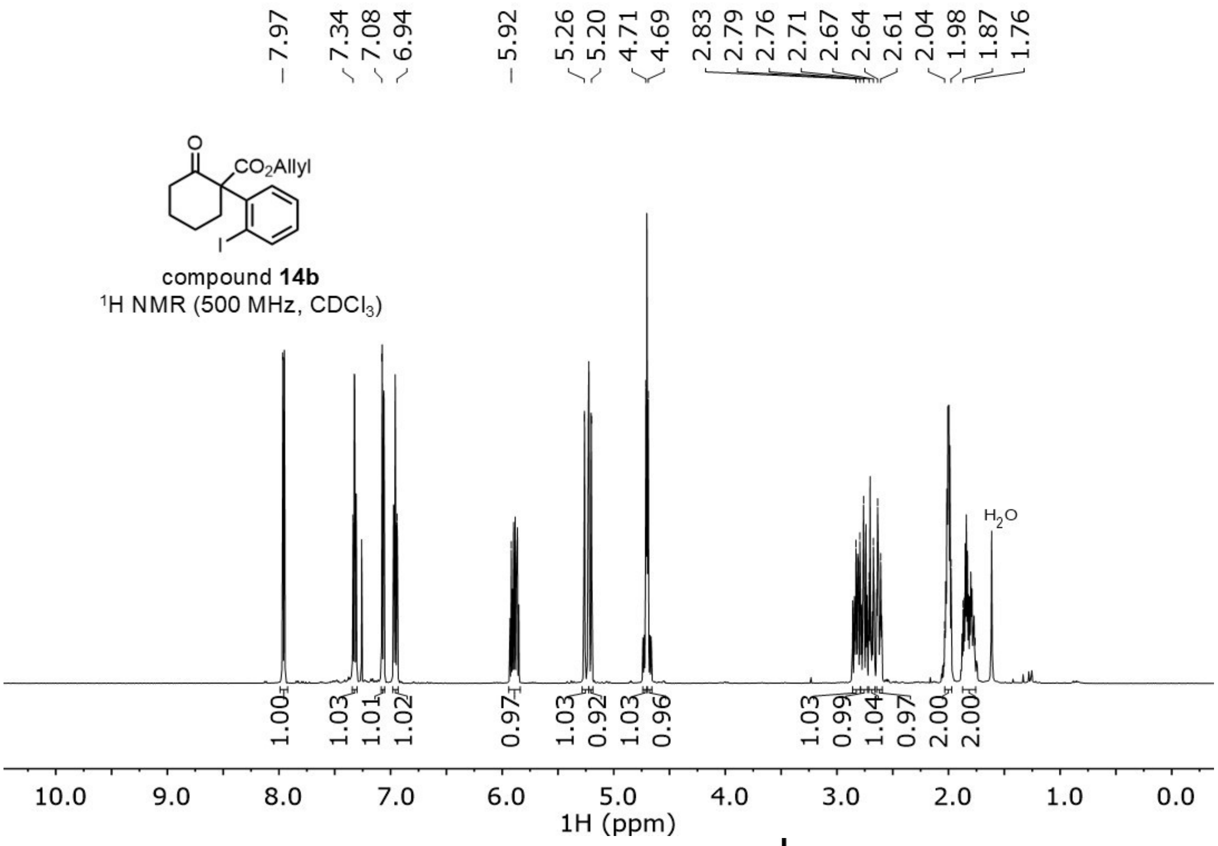
7.97 7.34 7.08 6.94 5.92 5.26 5.20 4.71 4.69 2.83 2.79 2.76 2.71 2.67 2.64 2.61 2.04 1.98 1.87 1.76
CO2Allyl
O
I
compound 14b
1H NMR (500 MHz, CDCl3)
H2O
1.00 1.03 1.01 1.02 0.97 1.03 0.92 1.03 0.96 1.03 0.99 1.04 0.97 2.00 2.00
10.0 9.0 8.0 7.0 6.0 5.0 4.0 3.0 2.0 1.0 0.0
1H (ppm)

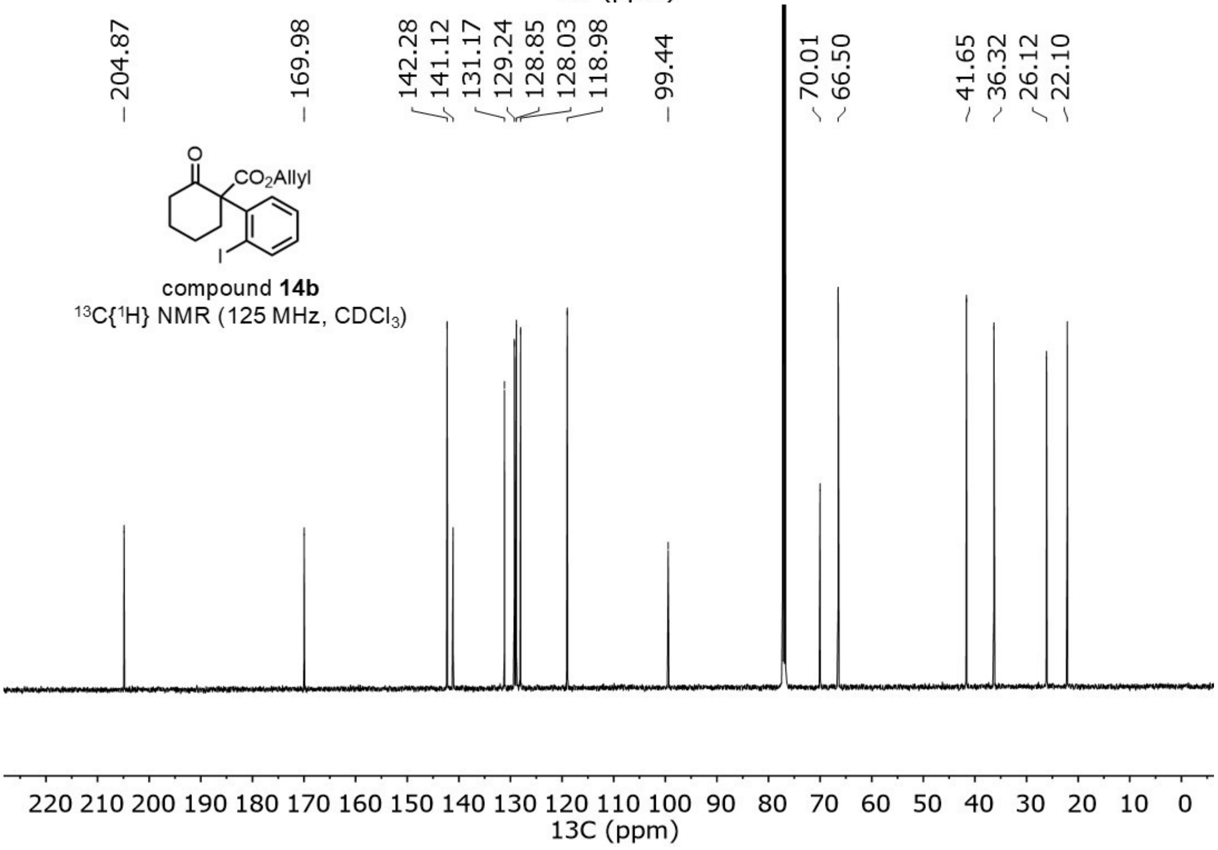
204.87 169.98 142.28 141.12 131.17 129.24 128.85 128.03 118.98 99.44 70.01 66.50 41.65 36.32 26.12 22.10
CO2Allyl
O
I
compound 14b
13C{1H} NMR (125 MHz, CDCl3)
220 210 200 190 180 170 160 150 140 130 120 110 100 90 80 70 60 50 40 30 20 10 0
13C (ppm)

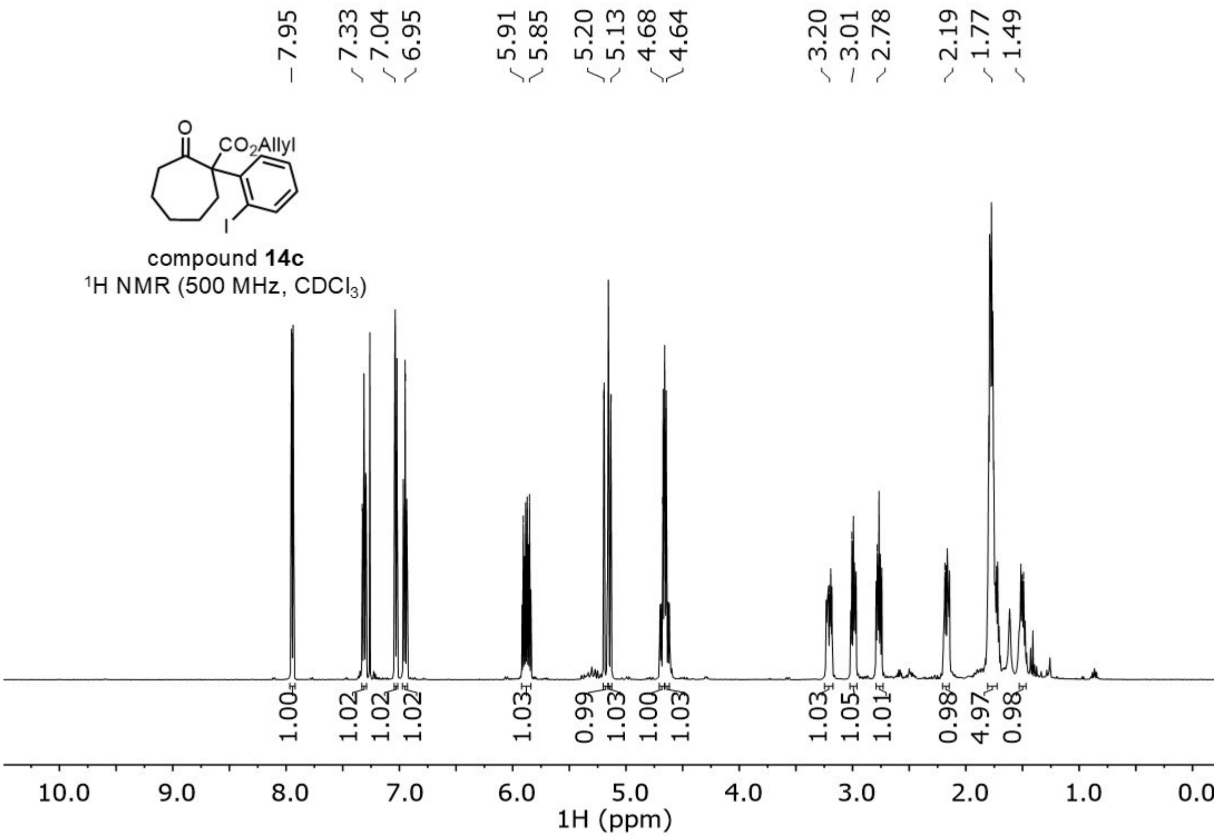
7.95
7.33
7.04
6.95
5.91
5.85
5.20
5.13
4.68
4.64
3.20
3.01
2.78
2.19
1.77
1.49
O
CO2Allyl
I
compound **14c**
1H NMR (500 MHz, CDCl3)
1.00
1.02
1.02
1.02
1.03
0.99
1.03
1.00
1.03
1.03
1.05
1.01
0.98
4.97
0.98
10.0
9.0
8.0
7.0
6.0
5.0
4.0
3.0
2.0
1.0
0.0
1H (ppm)

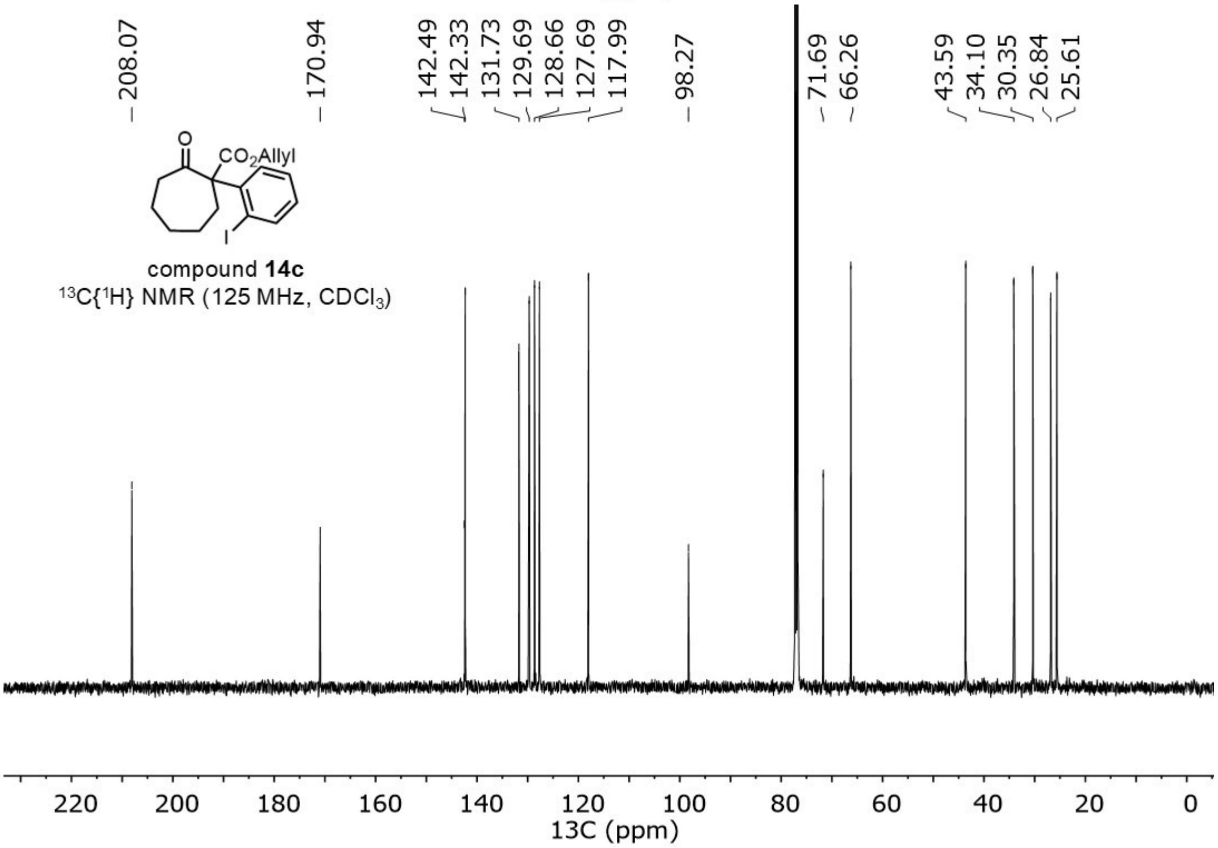
208.07
170.94
142.49
142.33
131.73
129.69
128.66
127.69
117.99
98.27
71.69
66.26
43.59
34.10
30.35
26.84
25.61
O
CO2Allyl
I
compound **14c**
13C{1H} NMR (125 MHz, CDCl3)
220
200
180
160
140
120
100
80
60
40
20
0
13C (ppm)

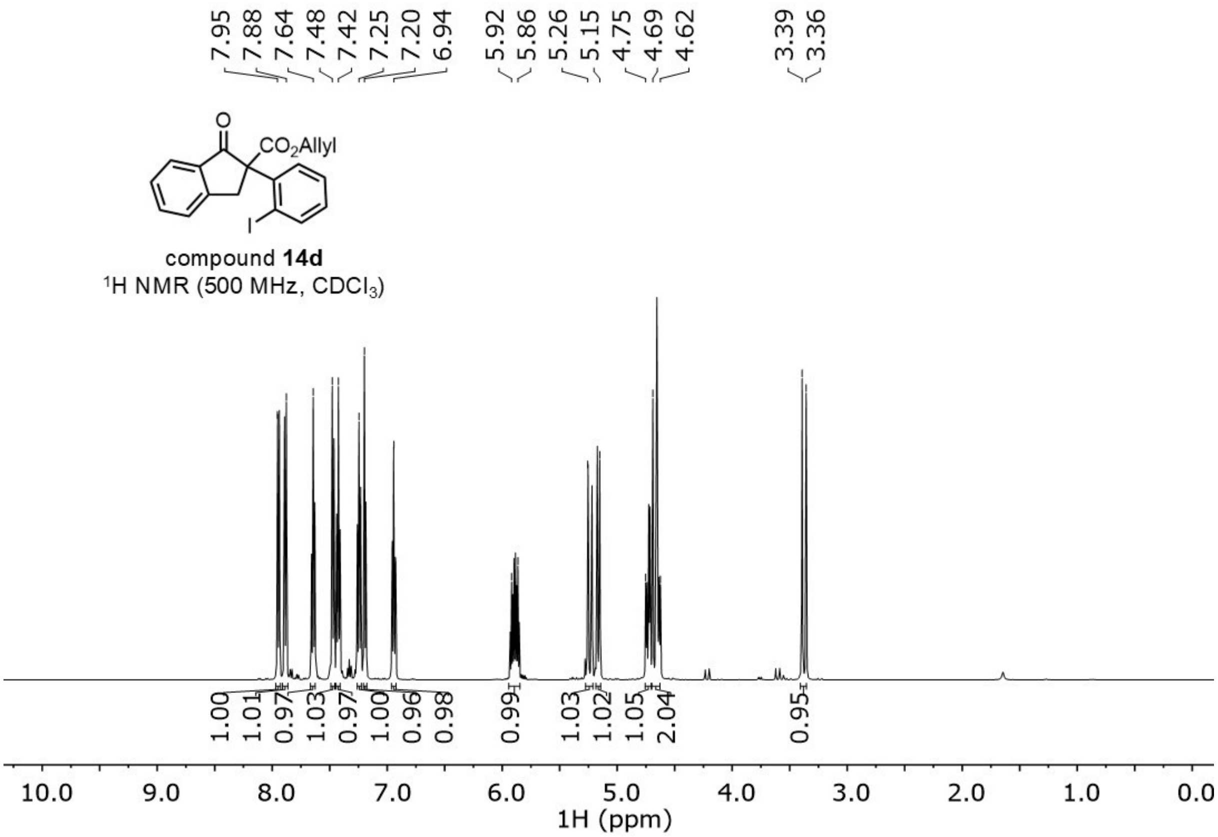
7.95
7.88
7.64
7.48
7.42
7.25
7.20
6.94
5.92
5.86
5.26
5.15
4.75
4.69
4.62
3.39
3.36
O
CO2Allyl
I
compound **14d**
1H NMR (500 MHz, CDCl3)
1.00
1.01
0.97
1.03
0.97
1.00
0.96
0.98
0.99
1.03
1.02
1.05
2.04
0.95
10.0
9.0
8.0
7.0
6.0
5.0
4.0
3.0
2.0
1.0
0.0
1H (ppm)

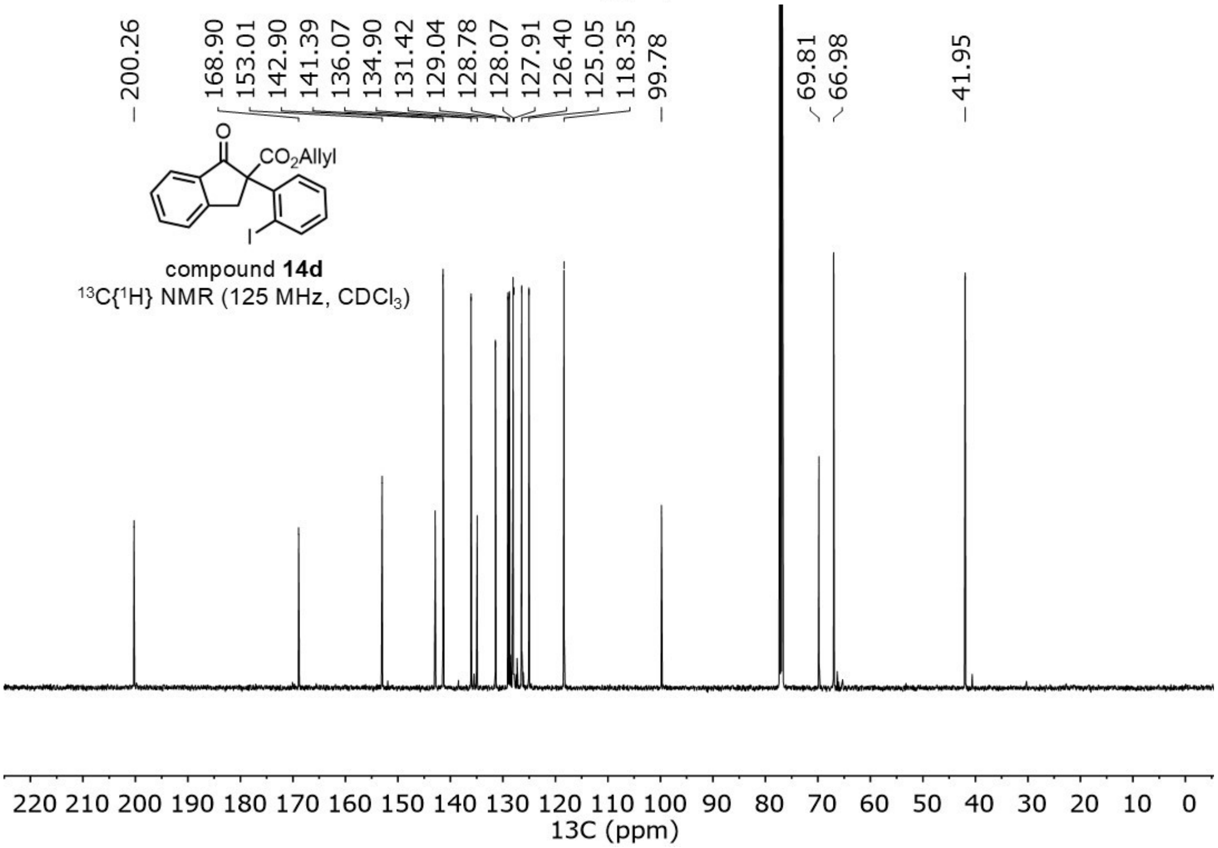
200.26
168.90
153.01
142.90
141.39
136.07
134.90
131.42
129.04
128.78
128.07
127.91
126.40
125.05
118.35
99.78
69.81
66.98
41.95
O
CO2Allyl
I
compound **14d**
13C{1H} NMR (125 MHz, CDCl3)
220 210 200 190 180 170 160 150 140 130 120 110 100 90 80 70 60 50 40 30 20 10 0
13C (ppm)

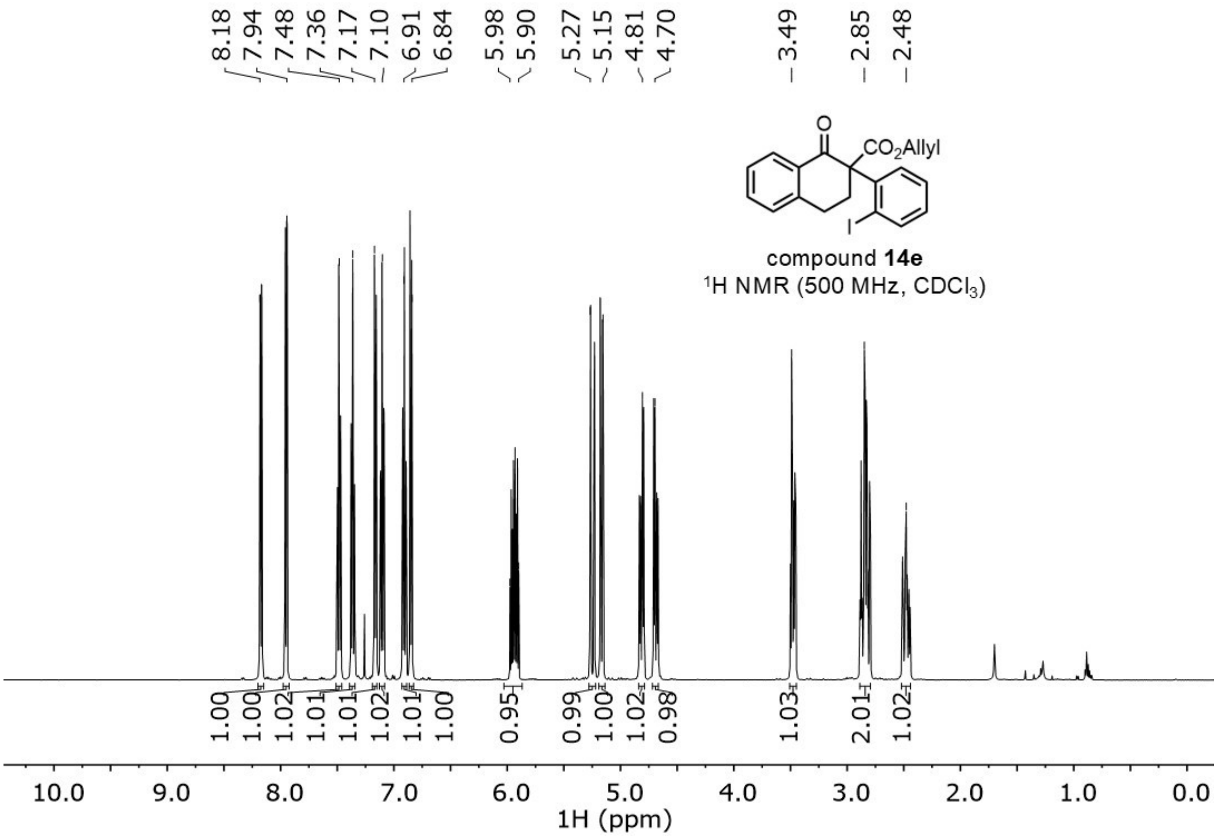
8.18
7.94
7.48
7.36
7.17
7.10
6.91
6.84
5.98
5.90
5.27
5.15
4.81
4.70
3.49
2.85
2.48
CO2Allyl
I
compound 14e
1H NMR (500 MHz, CDCl3)
1.00
1.00
1.02
1.01
1.01
1.02
1.01
1.00
0.95
0.99
1.00
1.02
0.98
1.03
2.01
1.02
10.0
9.0
8.0
7.0
6.0
5.0
4.0
3.0
2.0
1.0
0.0
1H (ppm)

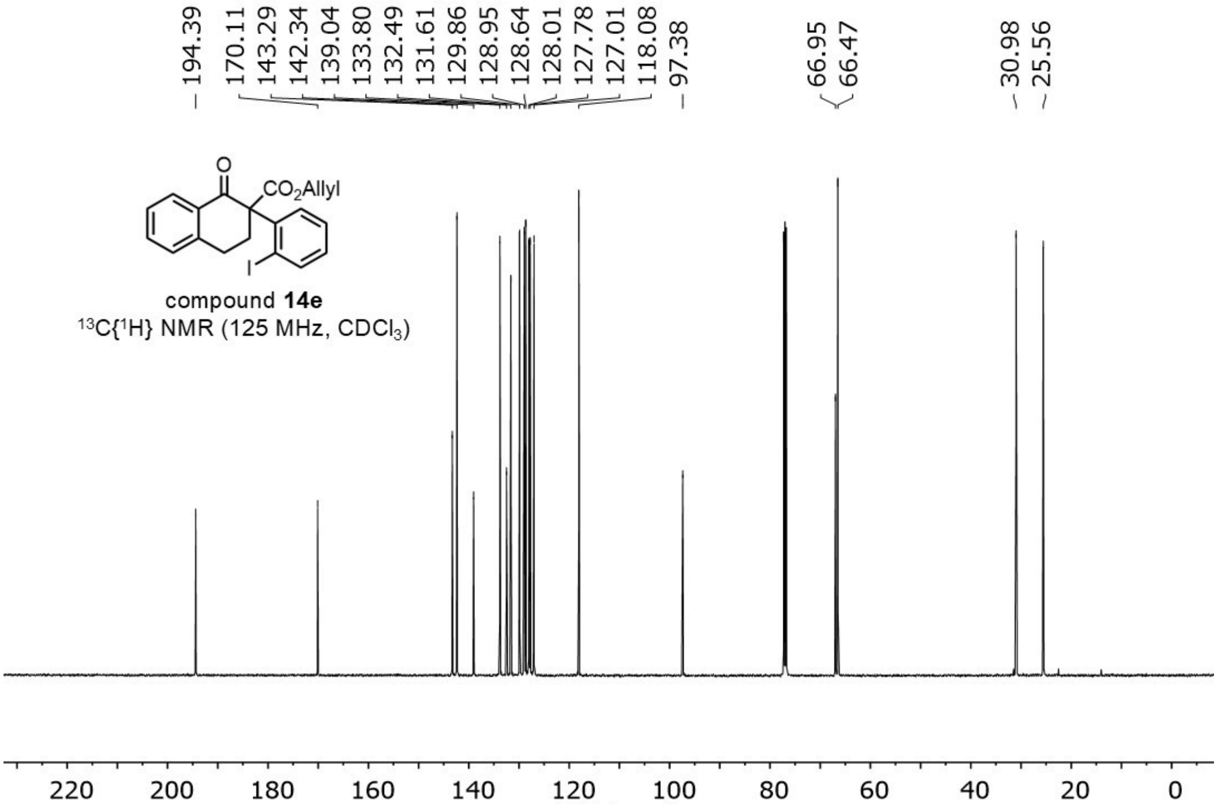
194.39
170.11
143.29
142.34
139.04
133.80
132.49
131.61
129.86
128.95
128.64
128.01
127.78
127.01
118.08
97.38
66.95
66.47
30.98
25.56
CO2Allyl
I
compound 14e
13C{1H} NMR (125 MHz, CDCl3)
220
200
180
160
140
120
100
80
60
40
20
0
13C (ppm)

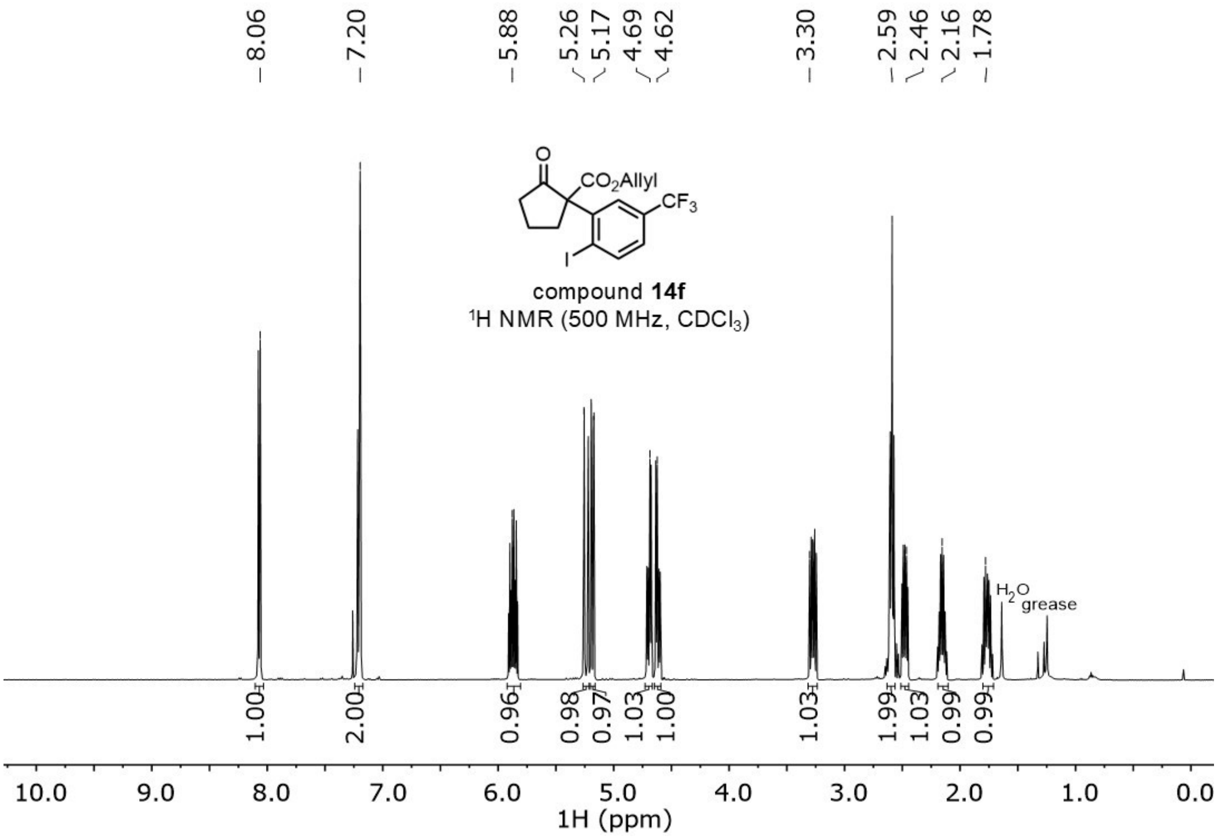
8.06
7.20
5.88
5.26
5.17
4.69
4.62
3.30
2.59
2.46
2.16
1.78
O
CO2Allyl
CF3
I
compound 14f
1H NMR (500 MHz, CDCl3)
H2O
grease
1.00
2.00
0.96
0.98
0.97
1.03
1.00
1.03
1.99
1.03
0.99
0.99
10.0
9.0
8.0
7.0
6.0
5.0
4.0
3.0
2.0
1.0
0.0
1H (ppm)

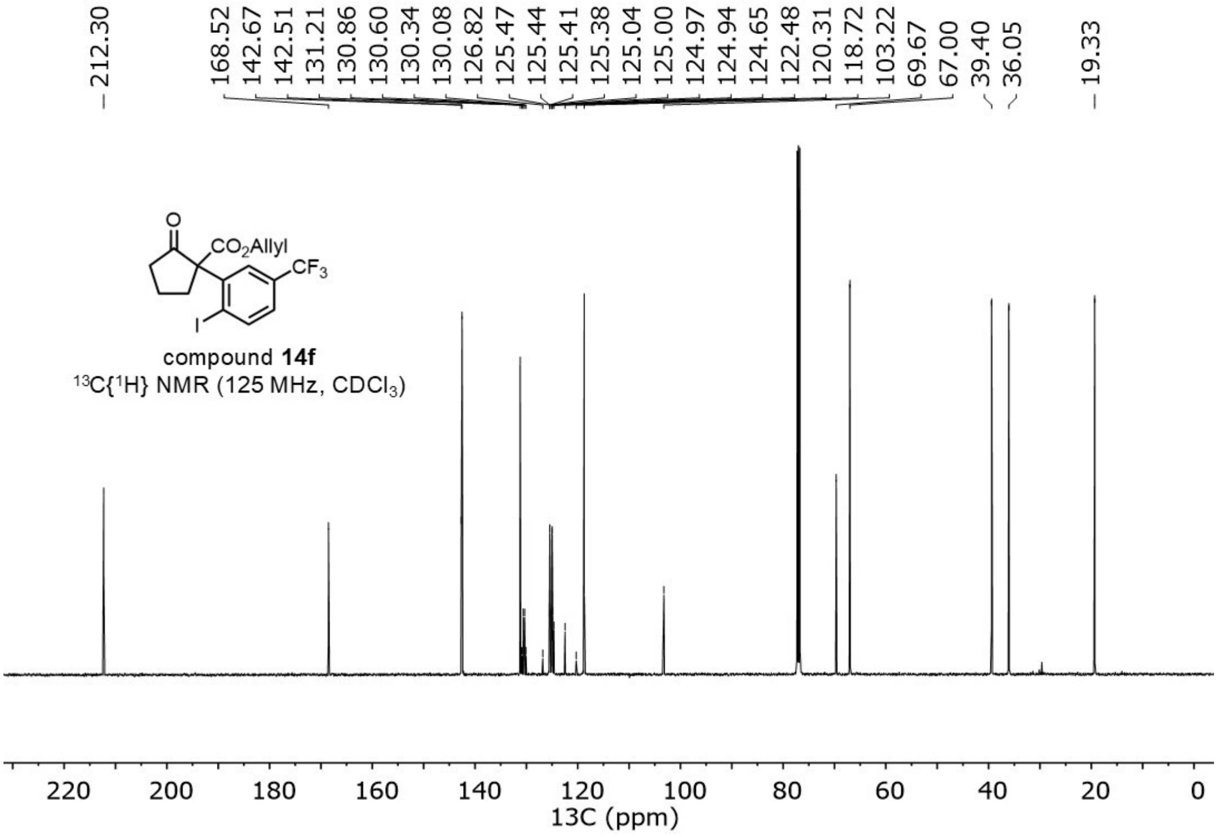
212.30
168.52
142.67
142.51
131.21
130.86
130.60
130.34
130.08
126.82
125.47
125.44
125.41
125.38
125.04
125.00
124.97
124.94
124.65
122.48
120.31
118.72
103.22
69.67
67.00
39.40
36.05
19.33
O
CO2Allyl
CF3
I
compound 14f
13C{1H} NMR (125 MHz, CDCl3)
220
200
180
160
140
120
100
80
60
40
20
0
13C (ppm)

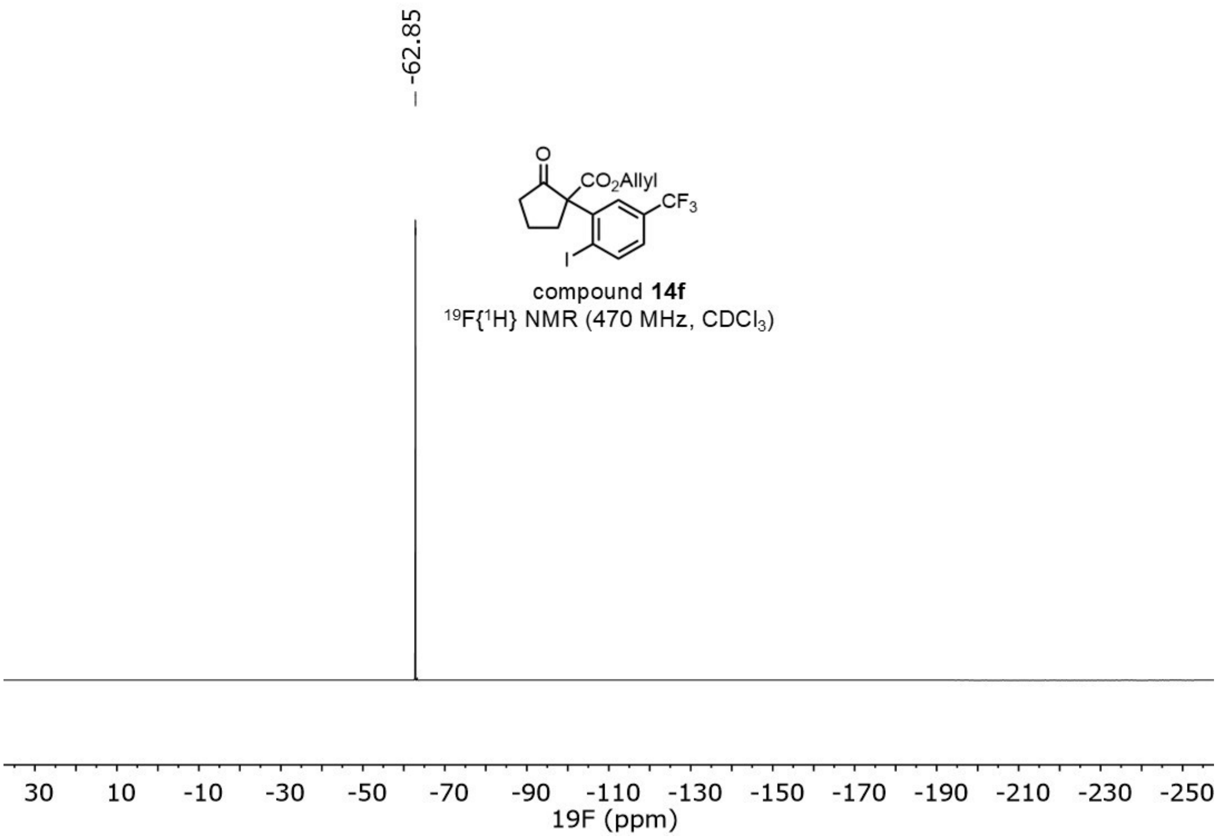

-62.85
O
CO2Allyl
CF3
I
compound 14f
19F{1H} NMR (470 MHz, CDCl3)
30 10 -10 -30 -50 -70 -90 -110 -130 -150 -170 -190 -210 -230 -250
19F (ppm)

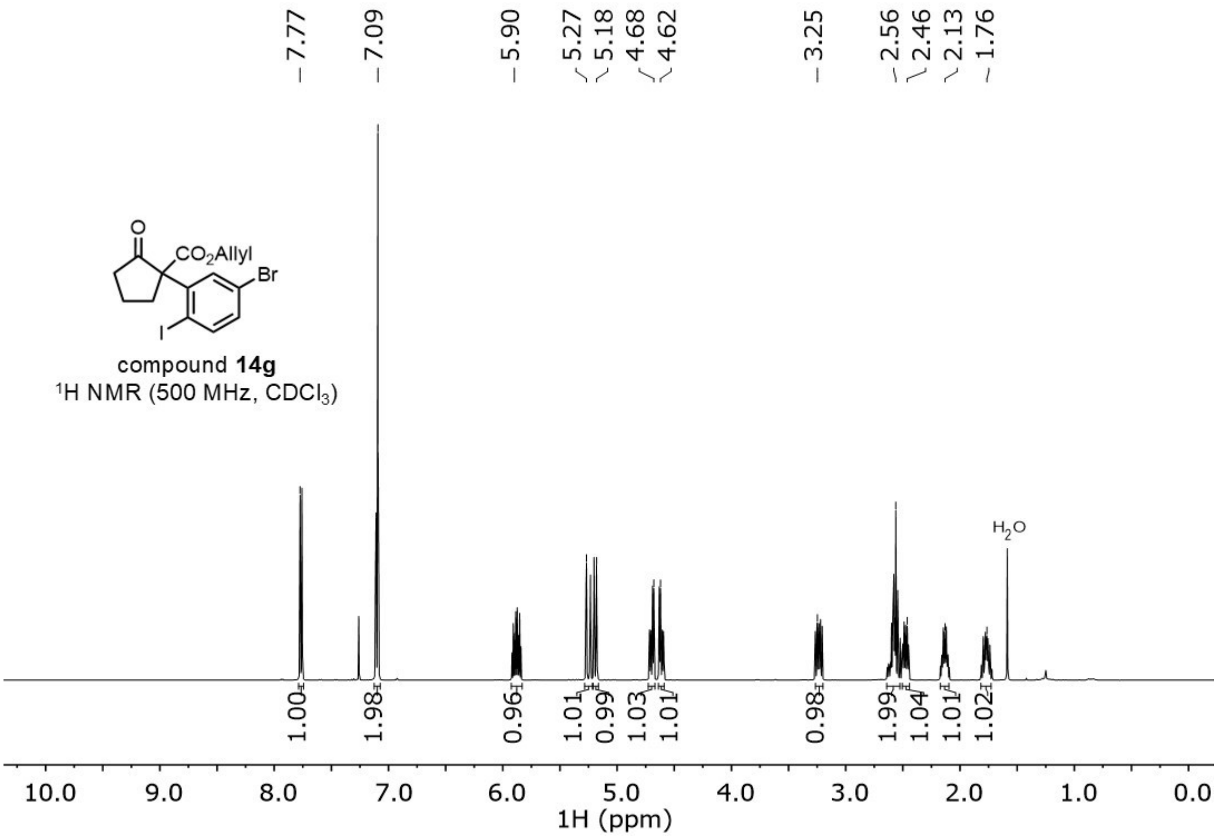
7.77
7.09
5.90
5.27
5.18
4.68
4.62
3.25
2.56
2.46
2.13
1.76
CO2Allyl
Br
I
compound 14g
1H NMR (500 MHz, CDCl3)
H2O
1.00
1.98
0.96
1.01
0.99
1.03
1.01
0.98
1.99
1.04
1.01
1.02
10.0
9.0
8.0
7.0
6.0
5.0
4.0
3.0
2.0
1.0
0.0
1H (ppm)

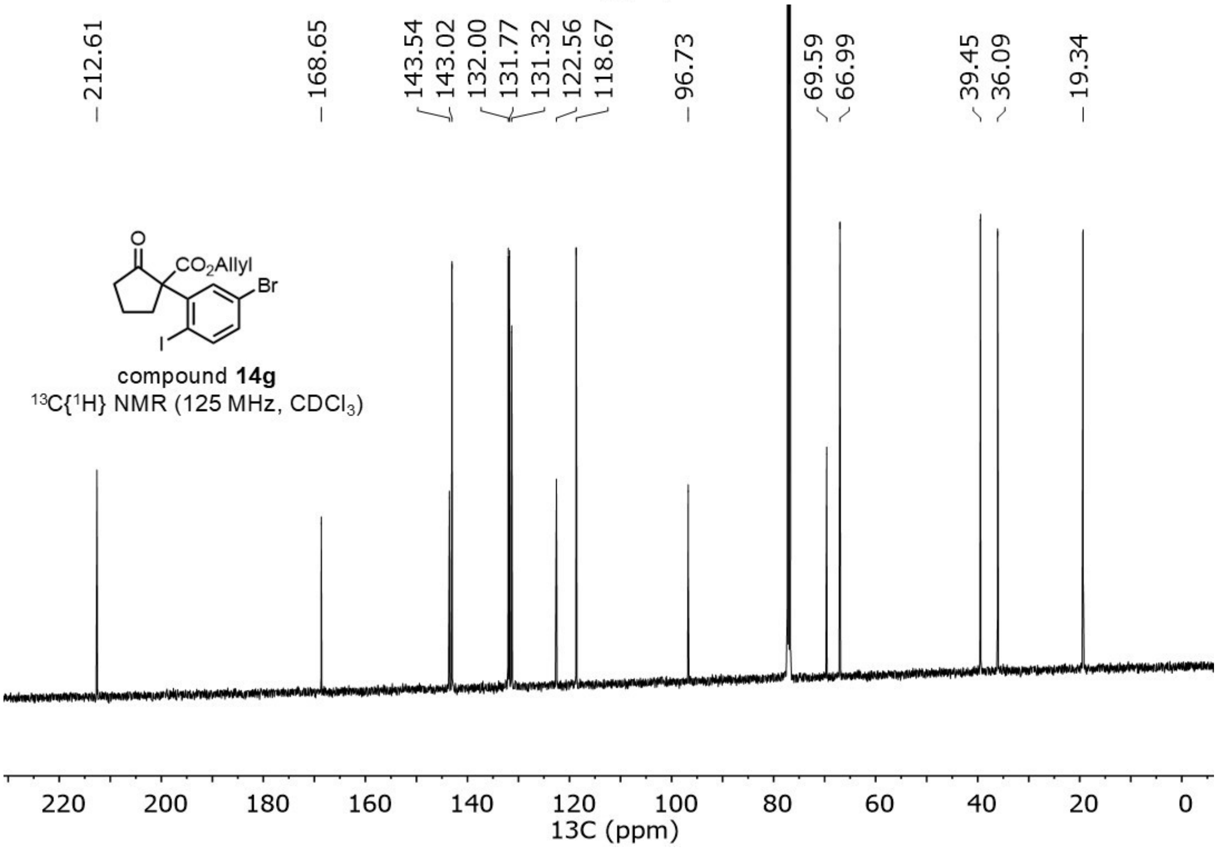
212.61
168.65
143.54
143.02
132.00
131.77
131.32
122.56
118.67
96.73
69.59
66.99
39.45
36.09
19.34
CO2Allyl
Br
I
compound 14g
13C{1H} NMR (125 MHz, CDCl3)
220
200
180
160
140
120
100
80
60
40
20
0
13C (ppm)

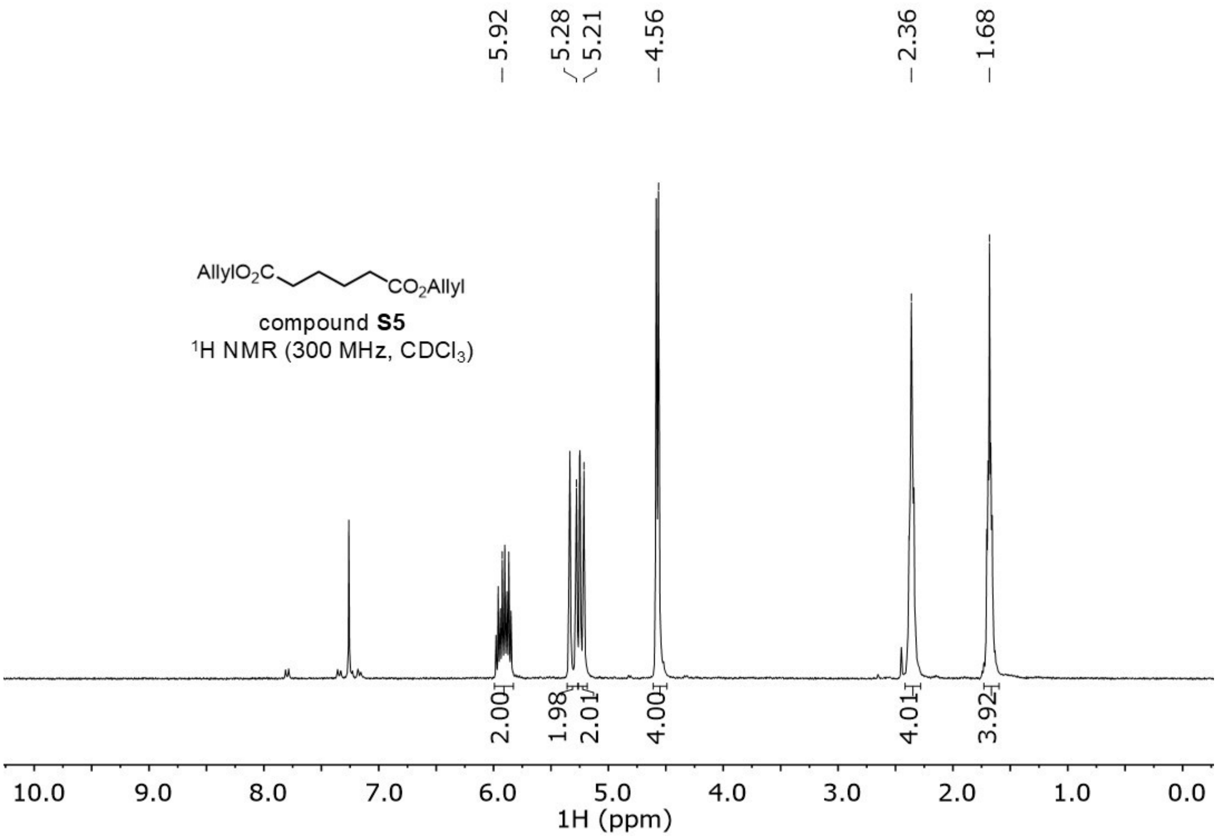
5.92
5.28
5.21
4.56
2.36
1.68
AllylO2C
CO2Allyl
compound S5
1H NMR (300 MHz, CDCl3)
2.00
1.98
2.01
4.00
4.01
3.92
10.0 9.0 8.0 7.0 6.0 5.0 4.0 3.0 2.0 1.0 0.0
1H (ppm)

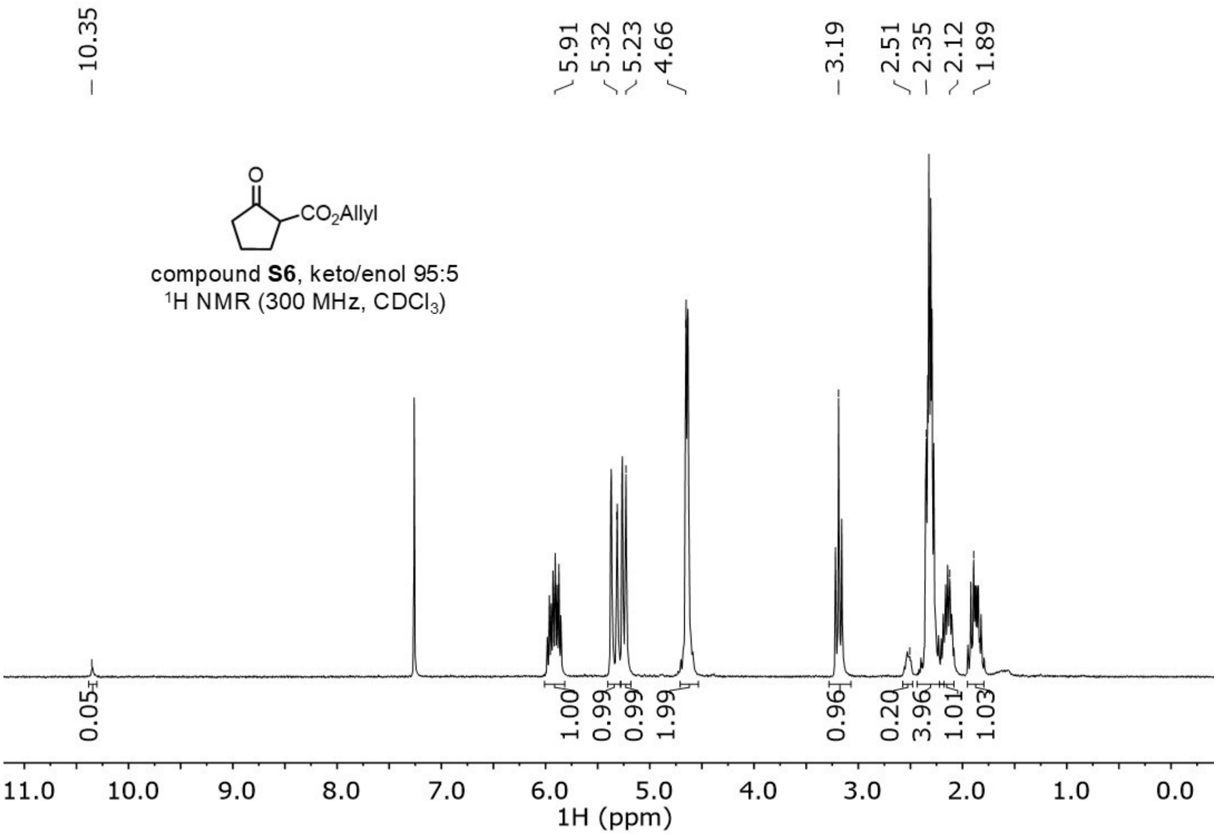
10.35
5.91
5.32
5.23
4.66
3.19
2.51
2.35
2.12
1.89
O
CO2Allyl
compound S6, keto/enol 95:5
1H NMR (300 MHz, CDCl3)
0.05
1.00
0.99
0.99
1.99
0.96
0.20
3.96
1.01
1.03
11.0 10.0 9.0 8.0 7.0 6.0 5.0 4.0 3.0 2.0 1.0 0.0
1H (ppm)

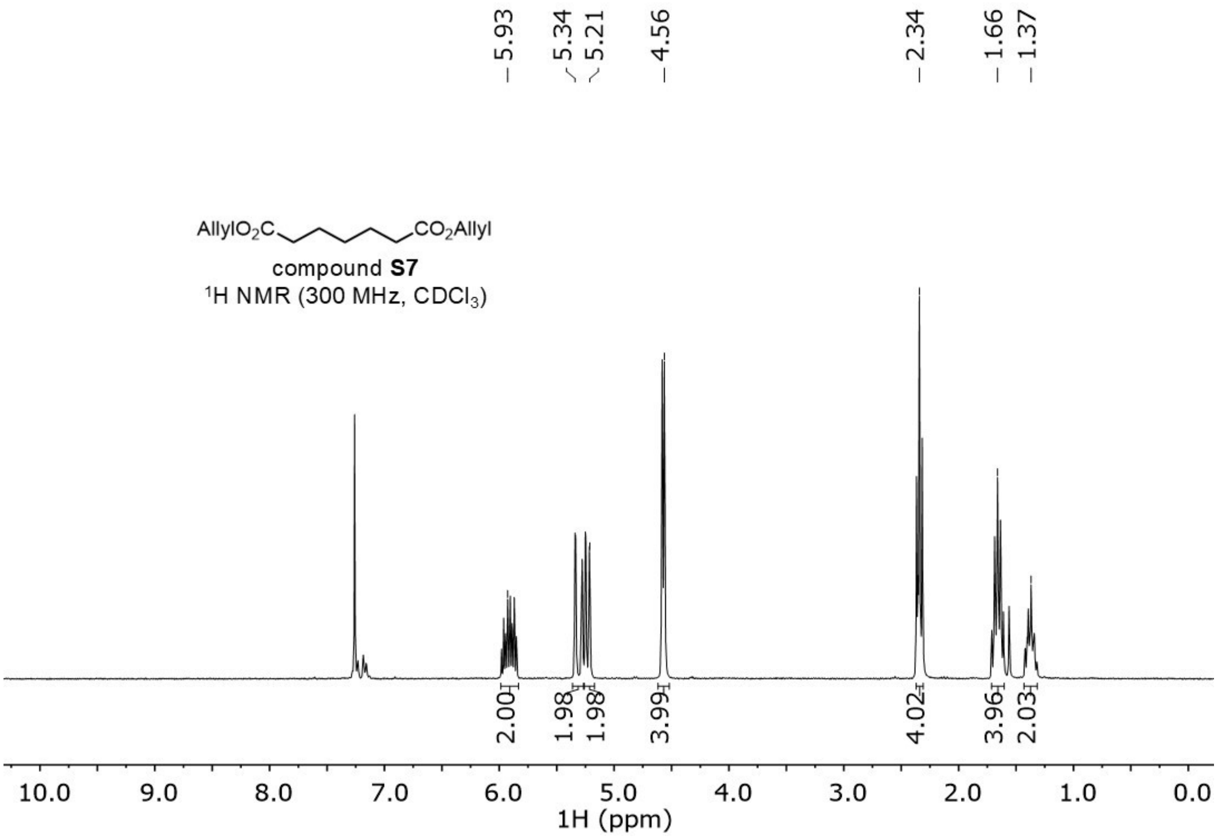
5.93
5.34
5.21
4.56
2.34
1.66
1.37
AllylO2C CO2Allyl
compound S7
1H NMR (300 MHz, CDCl3)
2.00
1.98
1.98
3.99
4.02
3.96
2.03
10.0 9.0 8.0 7.0 6.0 5.0 4.0 3.0 2.0 1.0 0.0
1H (ppm)

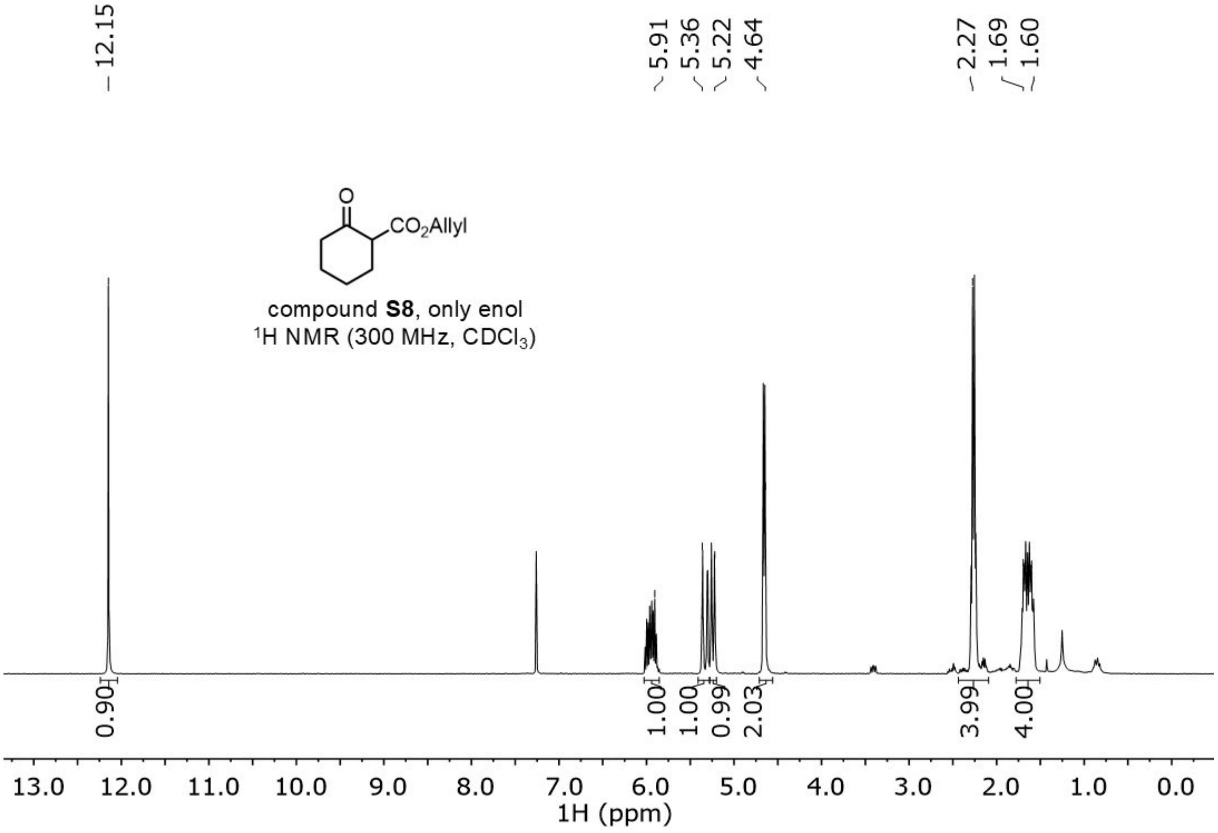
12.15
5.91
5.36
5.22
4.64
2.27
1.69
1.60
CO2Allyl
compound S8, only enol
1H NMR (300 MHz, CDCl3)
0.90
1.00
1.00
0.99
2.03
3.99
4.00
13.0 12.0 11.0 10.0 9.0 8.0 7.0 6.0 5.0 4.0 3.0 2.0 1.0 0.0
1H (ppm)

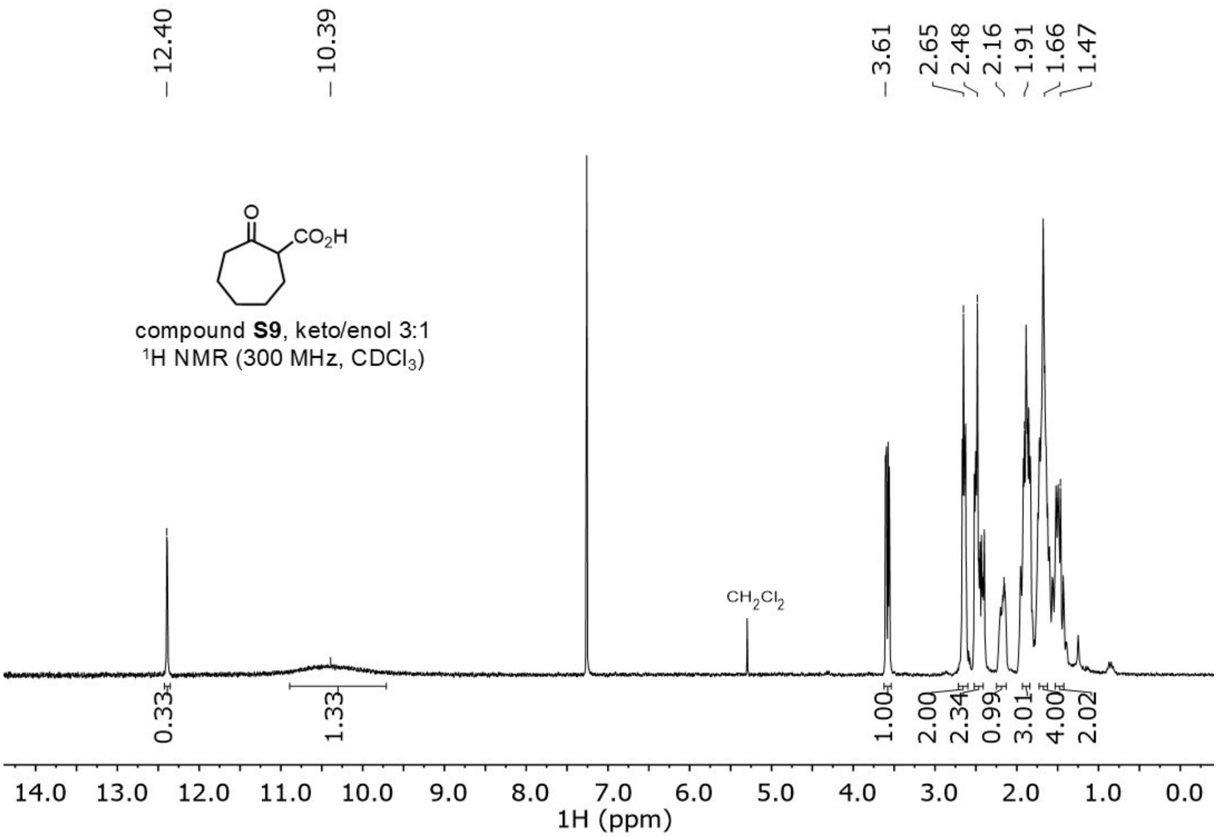
CO2H
compound S9, keto/enol 3:1
1H NMR (300 MHz, CDCl3)
CH2Cl2
12.40
10.39
3.61
2.65
2.48
2.16
1.91
1.66
1.47
0.33
1.33
1.00
2.00
2.34
0.99
3.01
4.00
2.02
1H (ppm)

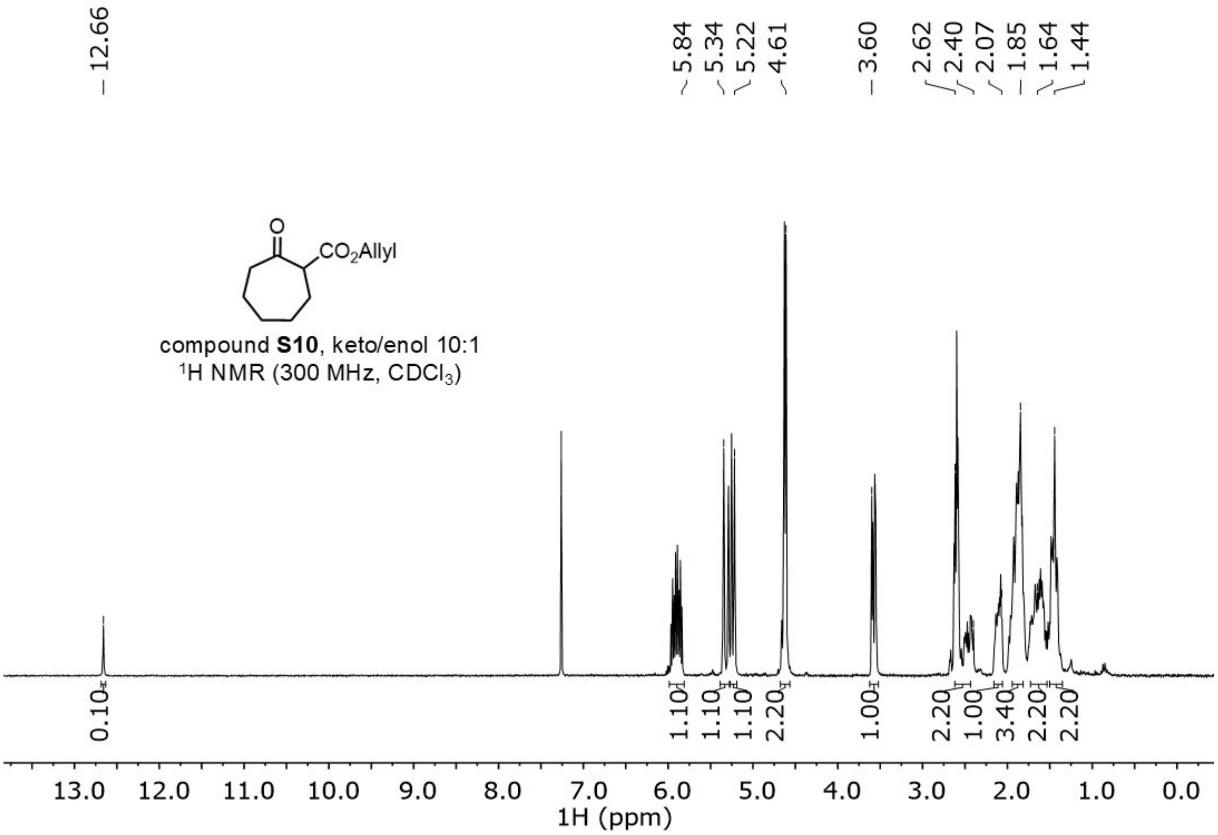
CO2Allyl
compound S10, keto/enol 10:1
1H NMR (300 MHz, CDCl3)
12.66
5.84
5.34
5.22
4.61
3.60
2.62
2.40
2.07
1.85
1.64
1.44
0.10
1.10
1.10
1.10
2.20
1.00
2.20
1.00
3.40
2.20
2.20
1H (ppm)

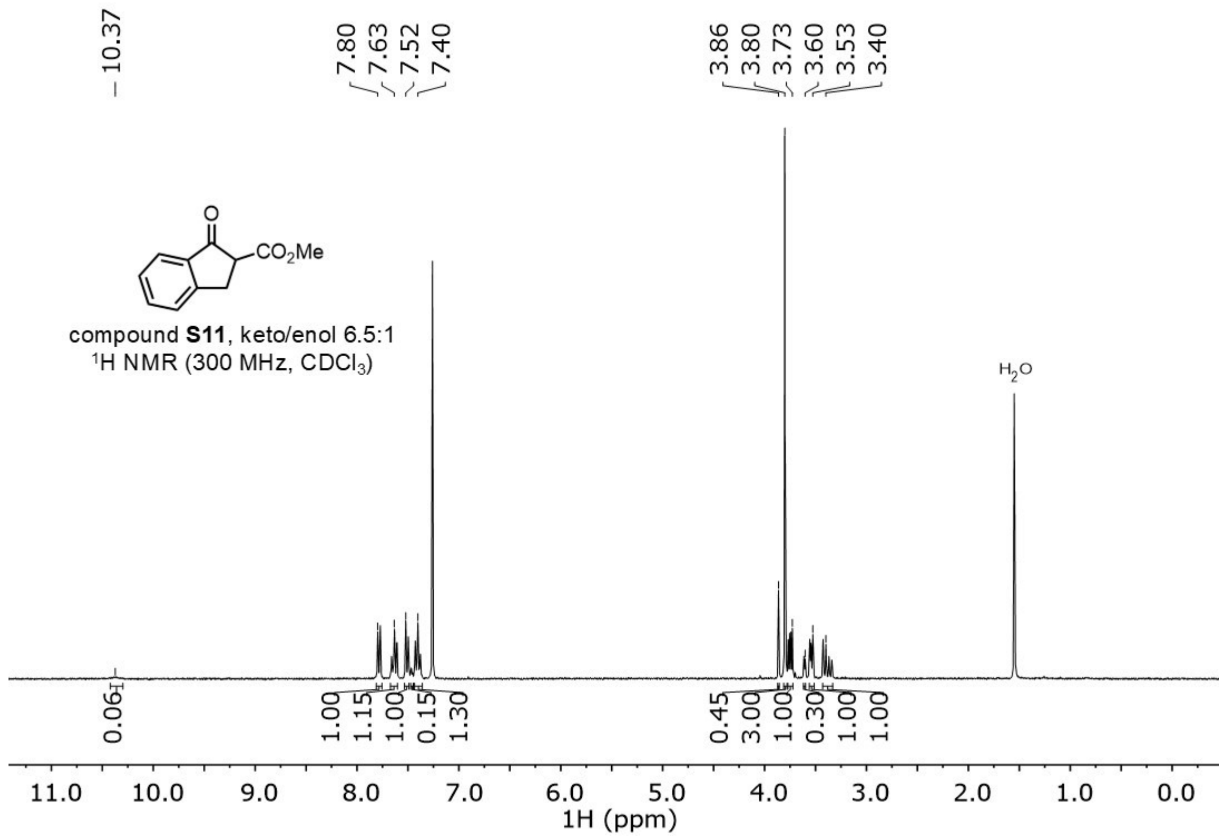
10.37
7.80
7.63
7.52
7.40
3.86
3.80
3.73
3.60
3.53
3.40
O
CO2Me
compound S11, keto/enol 6.5:1
1H NMR (300 MHz, CDCl3)
H2O
0.06
1.00
1.15
1.00
0.15
1.30
0.45
3.00
1.00
0.30
1.00
1.00
11.0
10.0
9.0
8.0
7.0
6.0
5.0
4.0
3.0
2.0
1.0
0.0
1H (ppm)

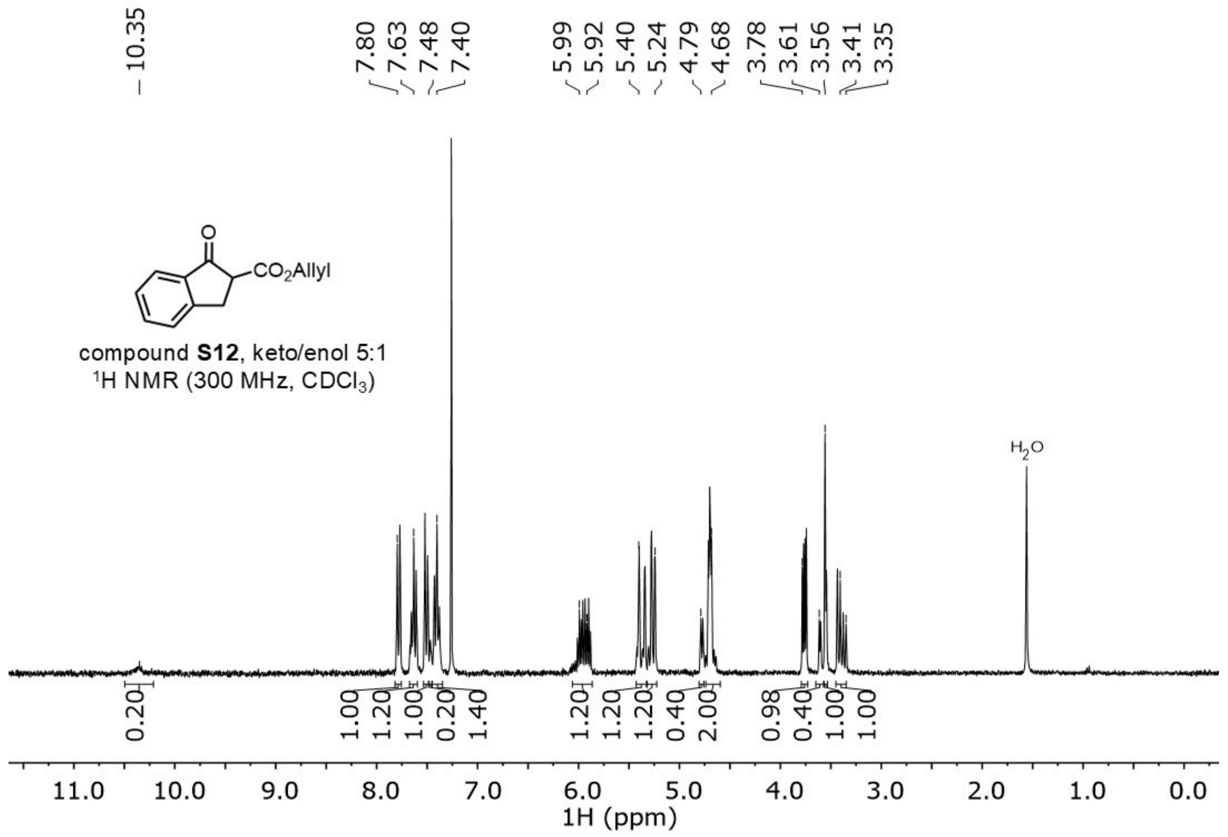
10.35
7.80
7.63
7.48
7.40
5.99
5.92
5.40
5.24
4.79
4.68
3.78
3.61
3.56
3.41
3.35
O
CO2Allyl
compound S12, keto/enol 5:1
1H NMR (300 MHz, CDCl3)
H2O
0.20
1.00
1.20
1.00
0.20
1.40
1.20
1.20
1.20
0.40
2.00
0.98
0.40
1.00
1.00
11.0
10.0
9.0
8.0
7.0
6.0
5.0
4.0
3.0
2.0
1.0
0.0
1H (ppm)

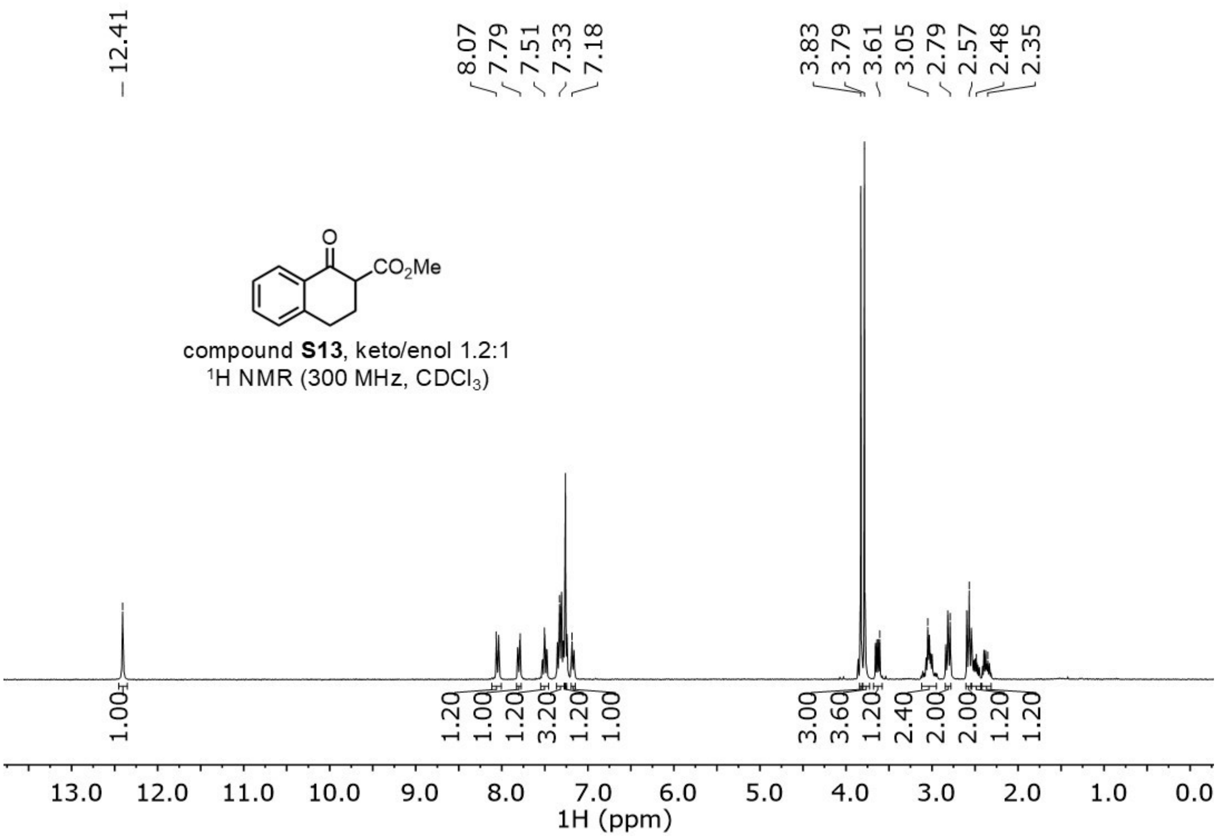
12.41
8.07 7.79 7.51 7.33 7.18
3.83 3.79 3.61 3.05 2.79 2.57 2.48 2.35
CO_2Me
compound **S13**, keto/enol 1.2:1
^{1}H NMR (300 MHz, $CDCl_3$)
1.00
1.20 1.00 1.20 3.20 1.20 1.00
3.00 3.60 1.20 2.40 2.00 2.00 1.20 1.20
13.0 12.0 11.0 10.0 9.0 8.0 7.0 6.0 5.0 4.0 3.0 2.0 1.0 0.0
1H (ppm)

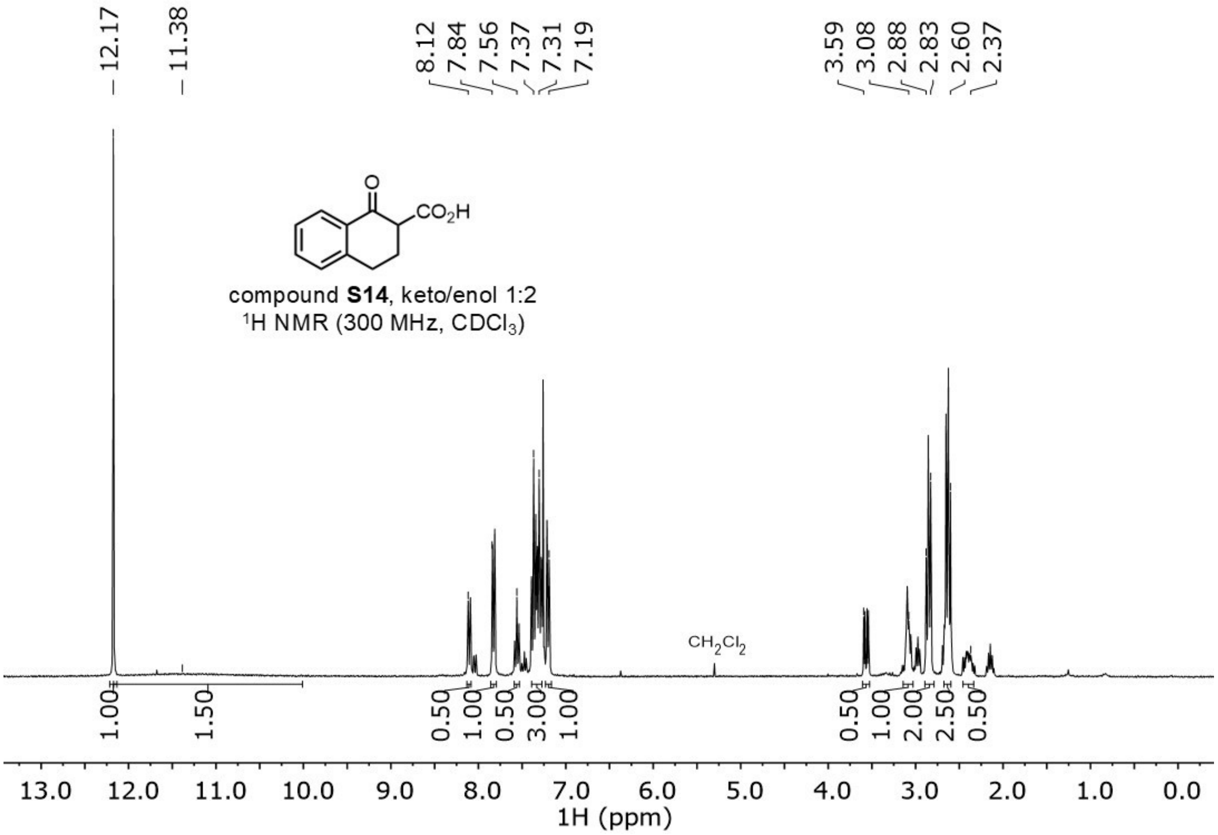
12.17
11.38
8.12 7.84 7.56 7.37 7.31 7.19
3.59 3.08 2.88 2.83 2.60 2.37
CO_2H
compound **S14**, keto/enol 1:2
^{1}H NMR (300 MHz, $CDCl_3$)
CH_2Cl_2
1.00 1.50
0.50 1.00 0.50 3.00 1.00
0.50 1.00 2.00 2.50 0.50
13.0 12.0 11.0 10.0 9.0 8.0 7.0 6.0 5.0 4.0 3.0 2.0 1.0 0.0
1H (ppm)

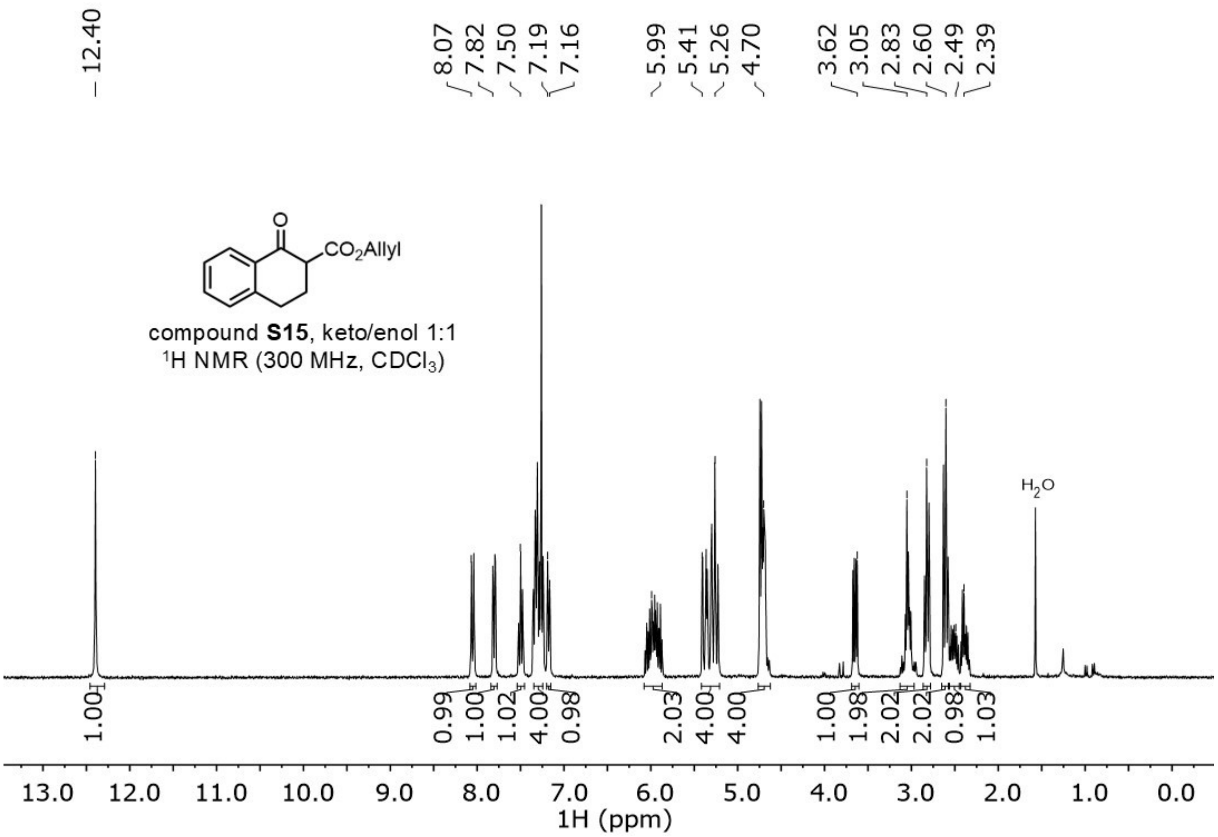

12.40
8.07
7.82
7.50
7.19
7.16
5.99
5.41
5.26
4.70
3.62
3.05
2.83
2.60
2.49
2.39
CO2Allyl
compound S15, keto/enol 1:1
1H NMR (300 MHz, CDCl3)
H2O
1.00
0.99
1.00
1.02
4.00
0.98
2.03
4.00
4.00
1.00
1.98
2.02
2.02
0.98
1.03
13.0
12.0
11.0
10.0
9.0
8.0
7.0
6.0
5.0
4.0
3.0
2.0
1.0
0.0
1H (ppm)

10. GLC on a Chiral Phase

<Chromatogram> Compound **15a**

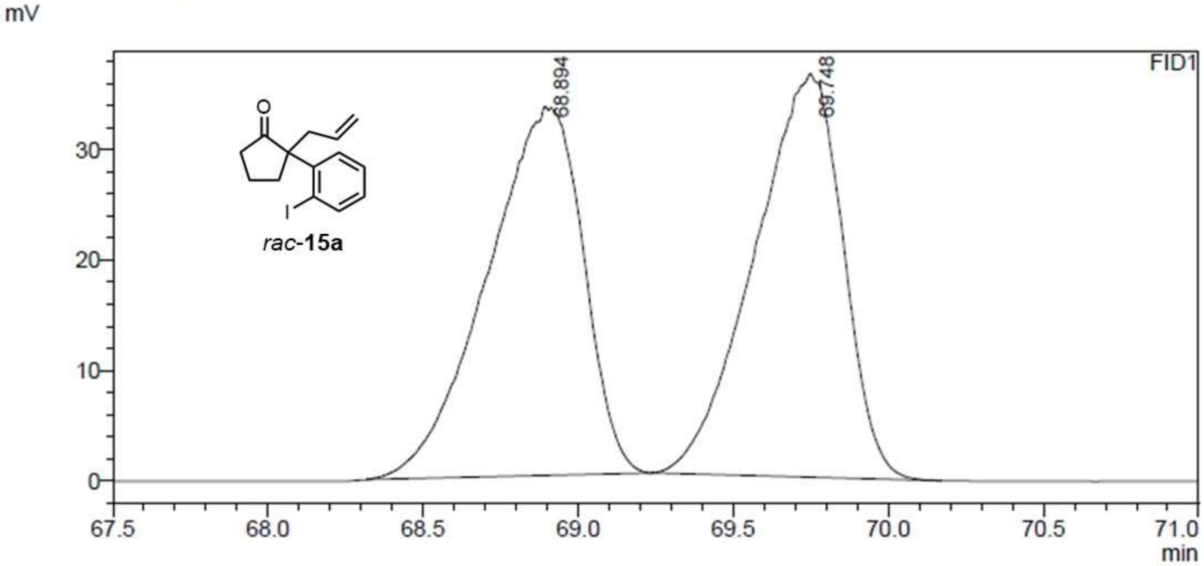

<Peak Table>

FID1

Peak#	Ret. Time	Area	Height	Conc.
1	68.894	720165	33383	49.833
2	69.748	724996	36460	50.167
Total		1445161	69843	

<Chromatogram> Compound **15a**

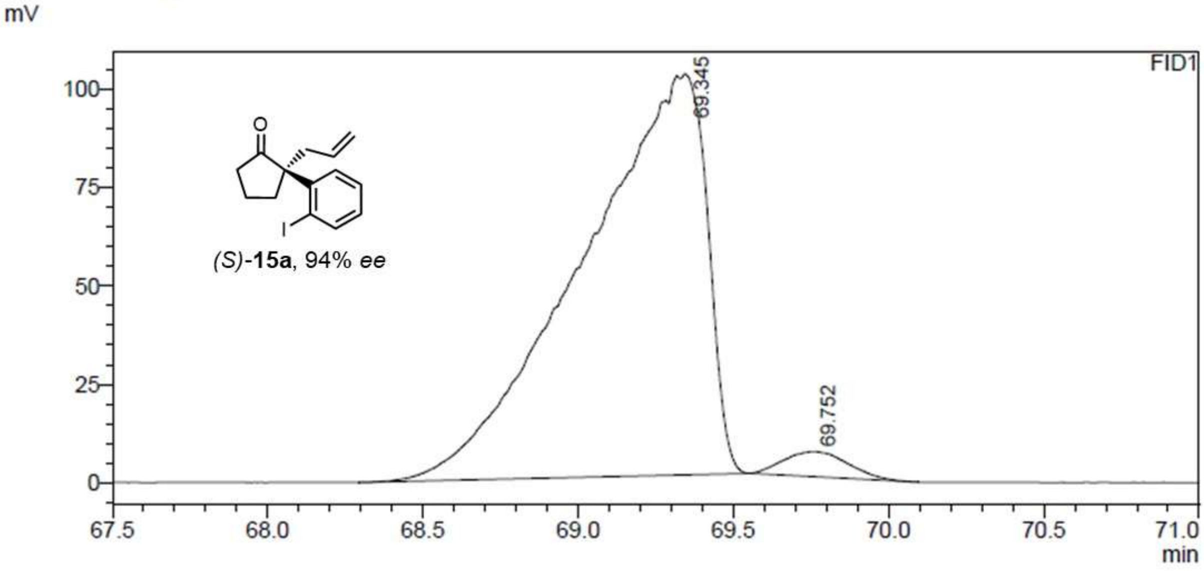

<Peak Table>

FID1

Peak#	Ret. Time	Area	Height	Conc.
1	69.345	2841001	101690	96.886
2	69.752	91324	6344	3.114
Total		2932325	108034	

<Chromatogram>

Compound **15b**

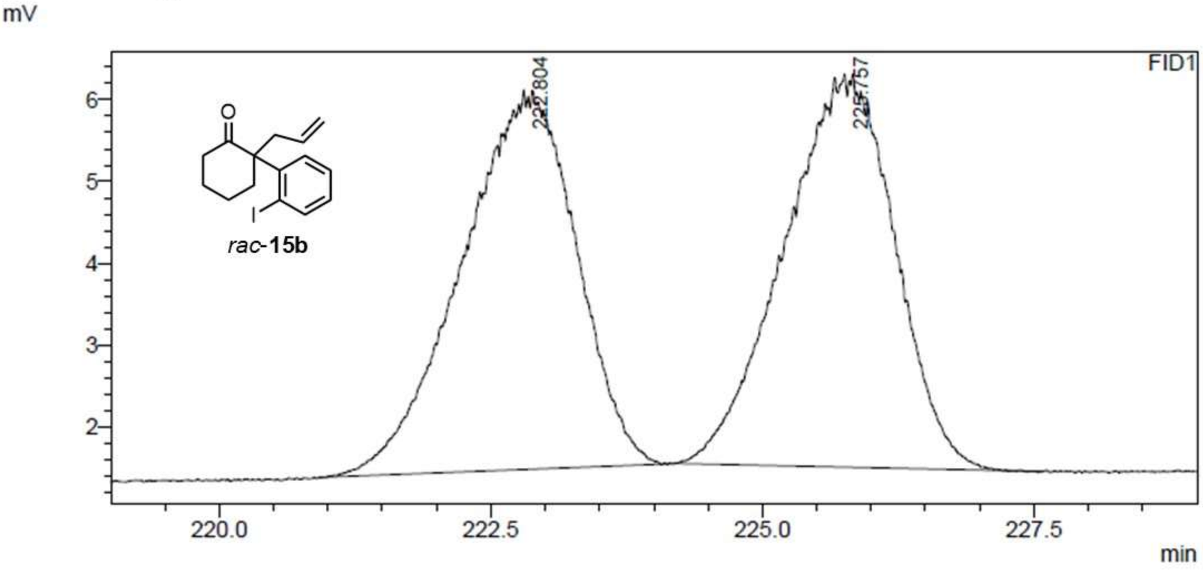

<Peak Table>

FID1

Peak#	Ret. Time	Area	Height	Conc.
1	222.804	339273	4635	49.947
2	225.757	339994	4802	50.053
Total		679266	9437	

<Chromatogram>

Compound **15b**

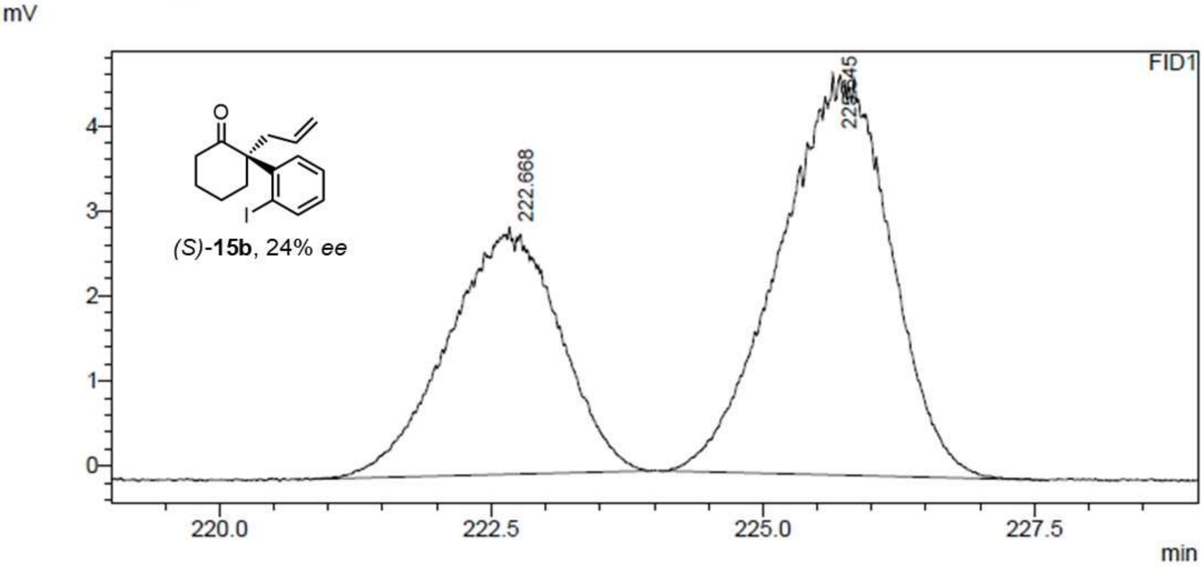

<Peak Table>

FID1

Peak#	Ret. Time	Area	Height	Conc.
1	222.668	205449	2903	38.093
2	225.645	333886	4710	61.907
Total		539335	7612	

<Chromatogram> Compound **15c**

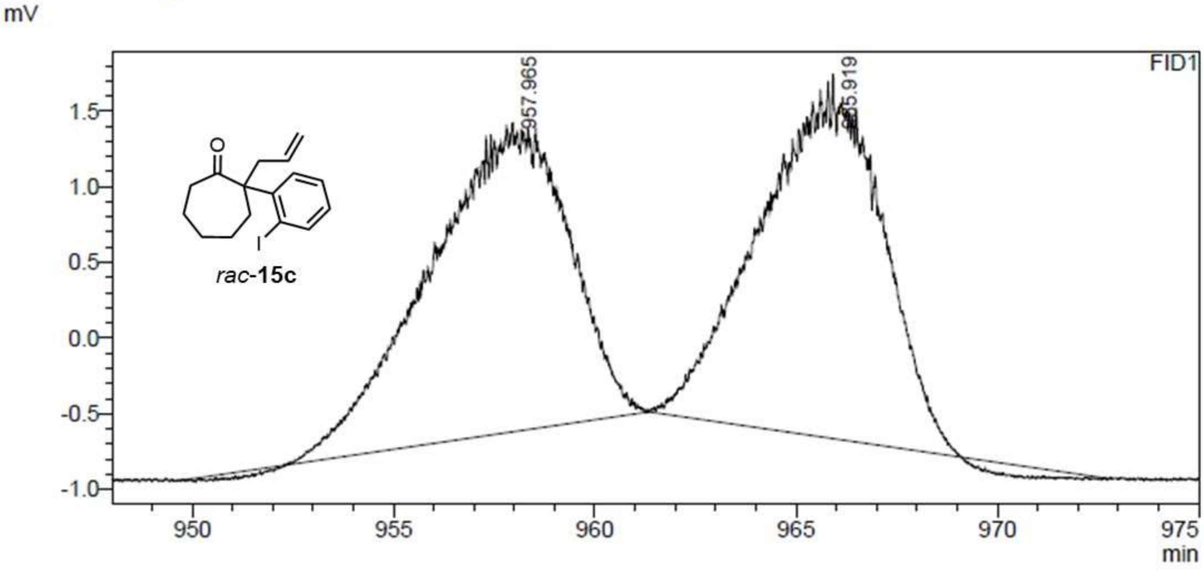

<Peak Table>

FID1

Peak#	Ret. Time	Area	Height	Conc.
1	957.965	487239	2043	50.042
2	965.919	486414	2409	49.958
Total		973653	4452	

<Chromatogram> Compound **15c**

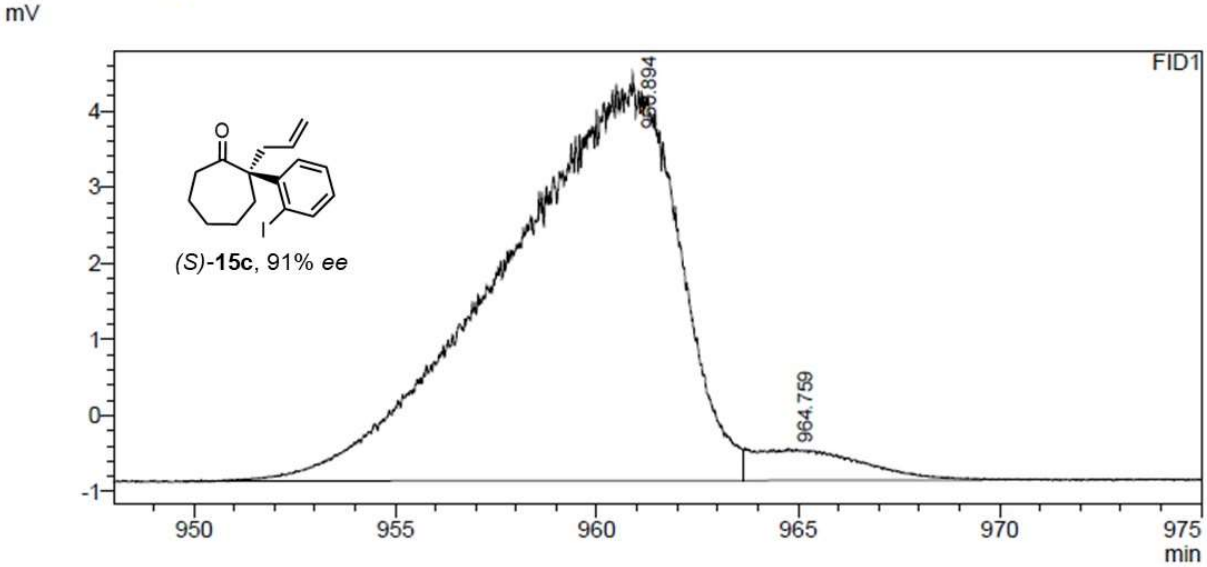

<Peak Table>

FID1

Peak#	Ret. Time	Area	Height	Conc.
1	960.894	1577608	5342	95.301
2	964.759	77789	418	4.699
Total		1655397	5760	

<Chromatogram> Compound **15d**

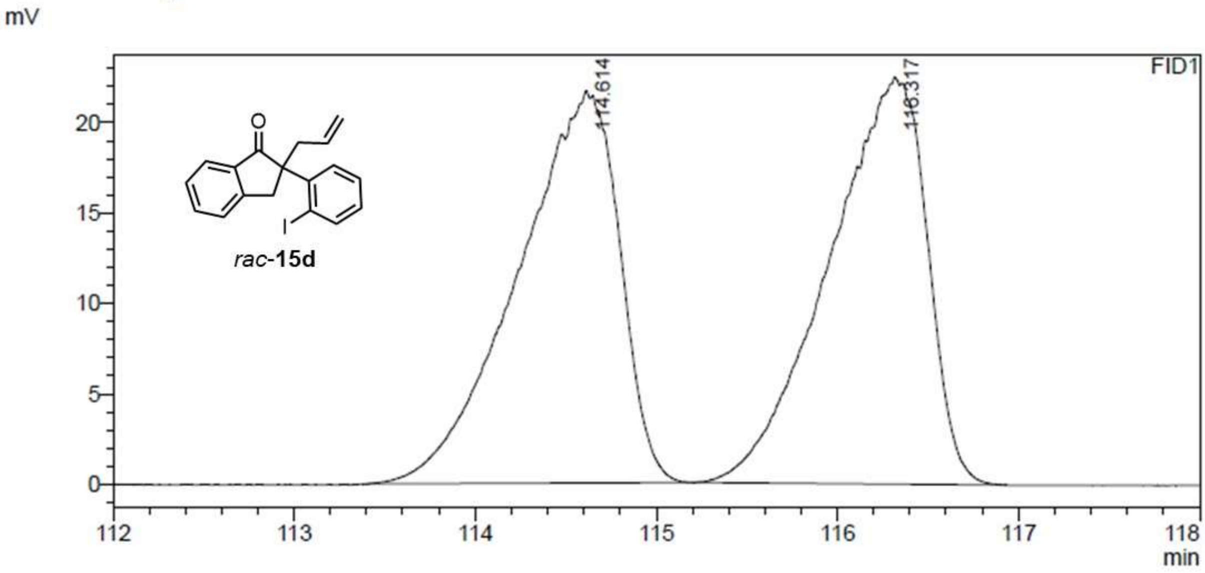

<Peak Table>

FID1

Peak#	Ret. Time	Area	Height	Conc.
1	114.614	845283	21653	49.917
2	116.317	848094	22477	50.083
Total		1693377	44130	

<Chromatogram> Compound **15d**

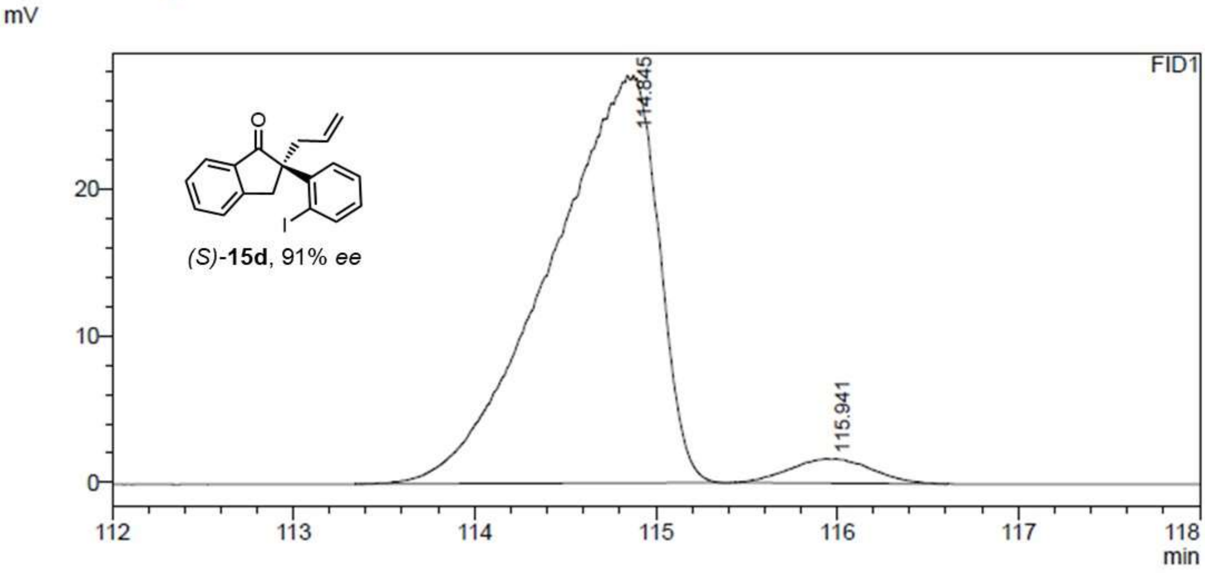

<Peak Table>

FID1

Peak#	Ret. Time	Area	Height	Conc.
1	114.845	1152532	27731	95.663
2	115.941	52250	1675	4.337
Total		1204782	29406	

<Chromatogram>

Compound **15e**

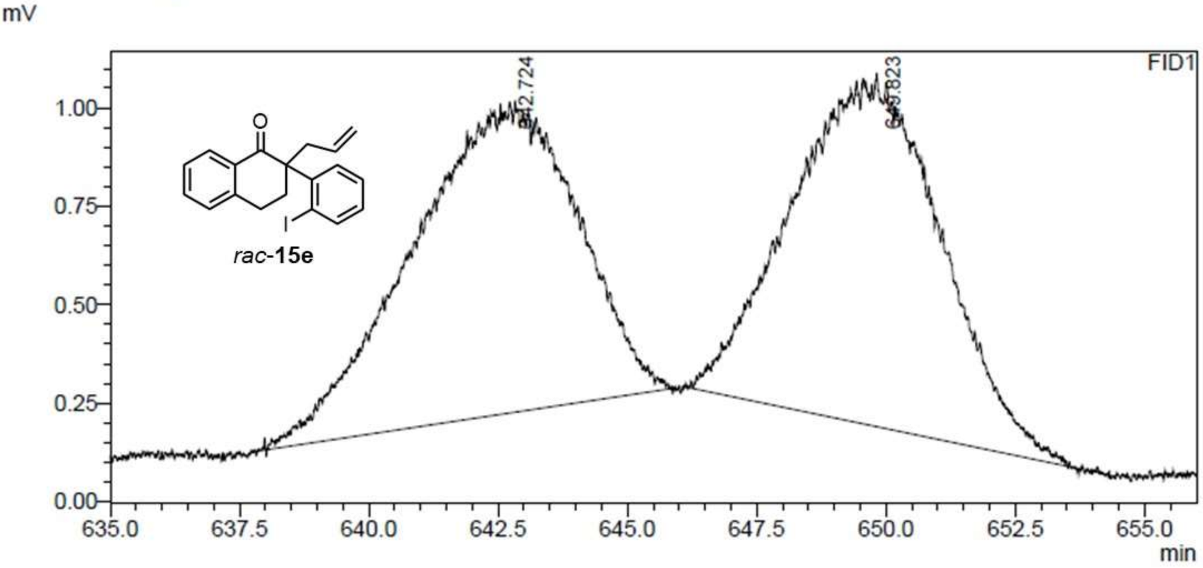

<Peak Table>

FID1

Peak#	Ret. Time	Area	Height	Conc.
1	642.724	175716	790	49.561
2	649.823	178829	896	50.439
Total		354545	1686	

<Chromatogram>

Compound **15e**

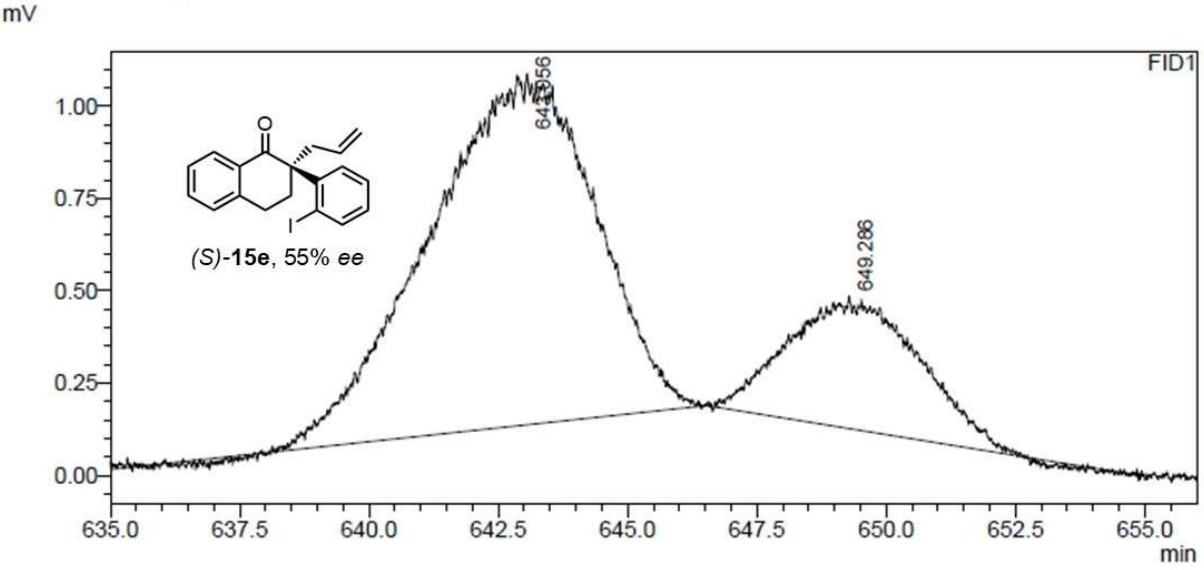

<Peak Table>

FID1

Peak#	Ret. Time	Area	Height	Conc.
1	643.056	211728	950	77.325
2	649.286	62089	358	22.675
Total		273818	1308	

<Chromatogram> Compound **15f**

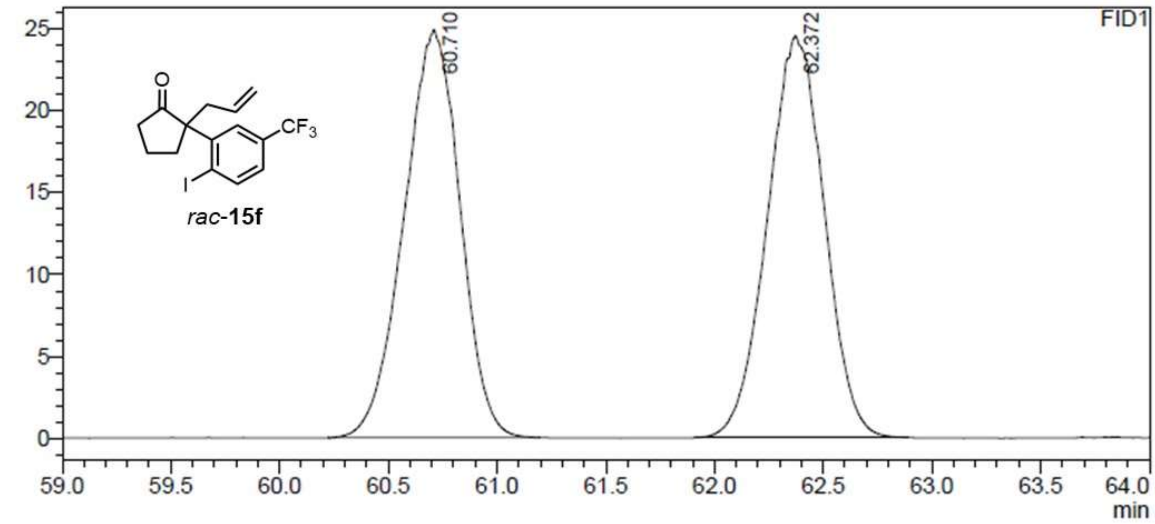

<Peak Table>

FID1

Peak#	Ret. Time	Area	Height	Conc.
1	60.710	445796	24815	50.022
2	62.372	445411	24477	49.978
Total		891208	49292	

<Chromatogram> Compound **15f**

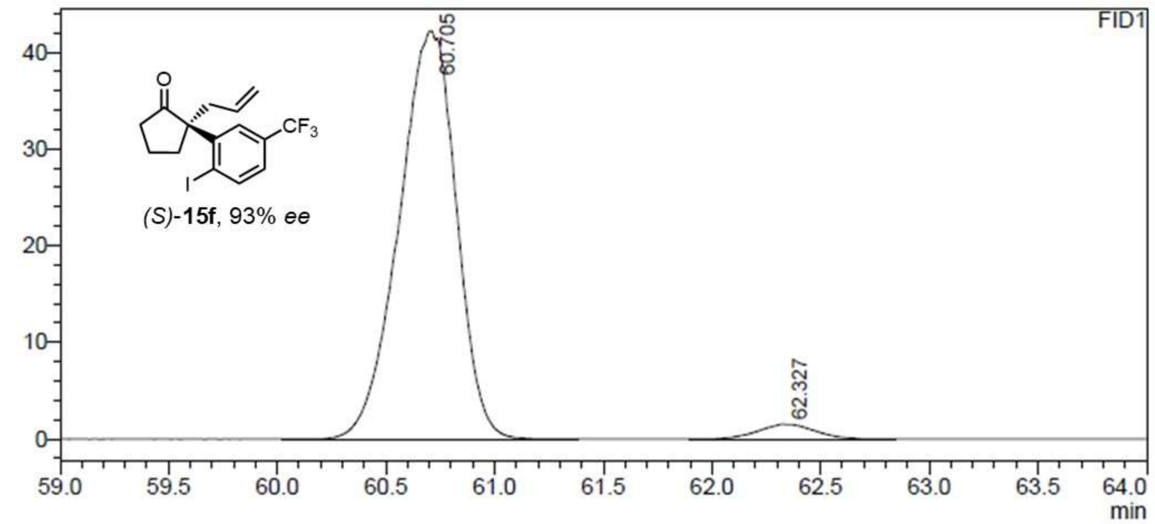

<Peak Table>

FID1

Peak#	Ret. Time	Area	Height	Conc.
1	60.705	775773	42193	96.576
2	62.327	27503	1517	3.424
Total		803276	43710	

<Chromatogram> Compound **15g**

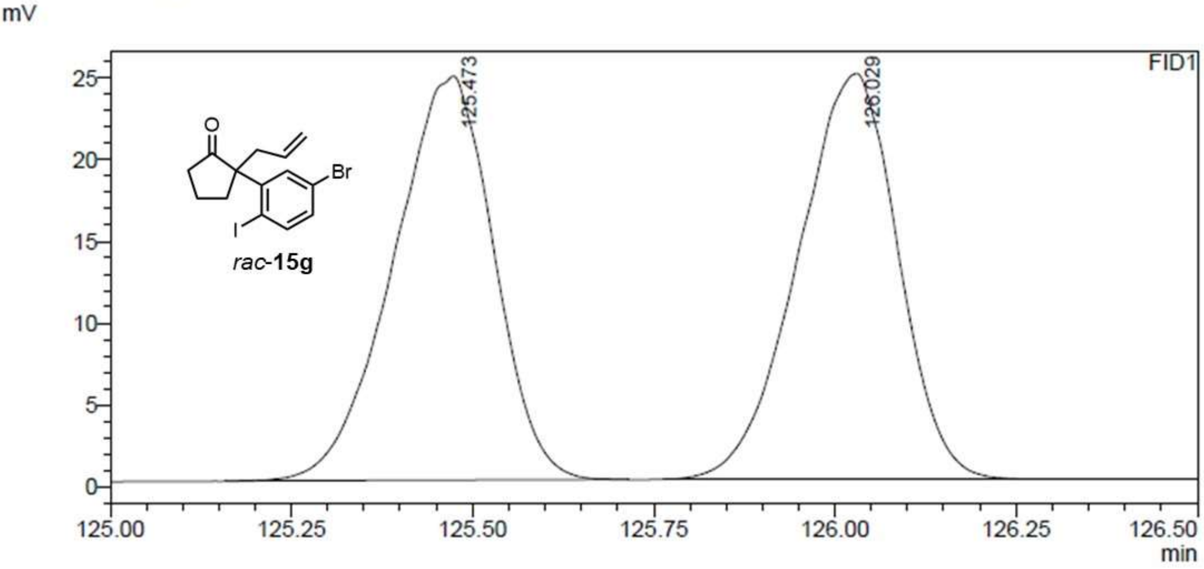

<Peak Table>

FID1

Peak#	Ret. Time	Area	Height	Conc.
1	125.473	238647	24689	50.059
2	126.029	238086	24806	49.941
Total		476732	49495	

<Chromatogram> Compound **15g**

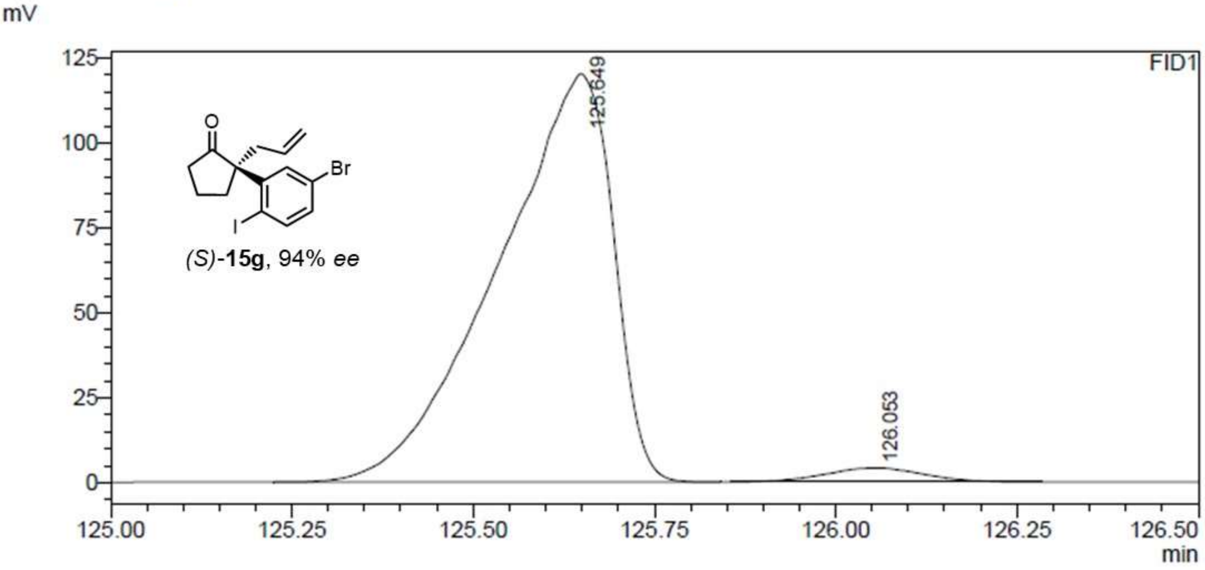

<Peak Table>

FID1

Peak#	Ret. Time	Area	Height	Conc.
1	125.649	1324677	120082	97.183
2	126.053	38391	4109	2.817
Total		1363068	124191	

<Chromatogram> Compound **17a**

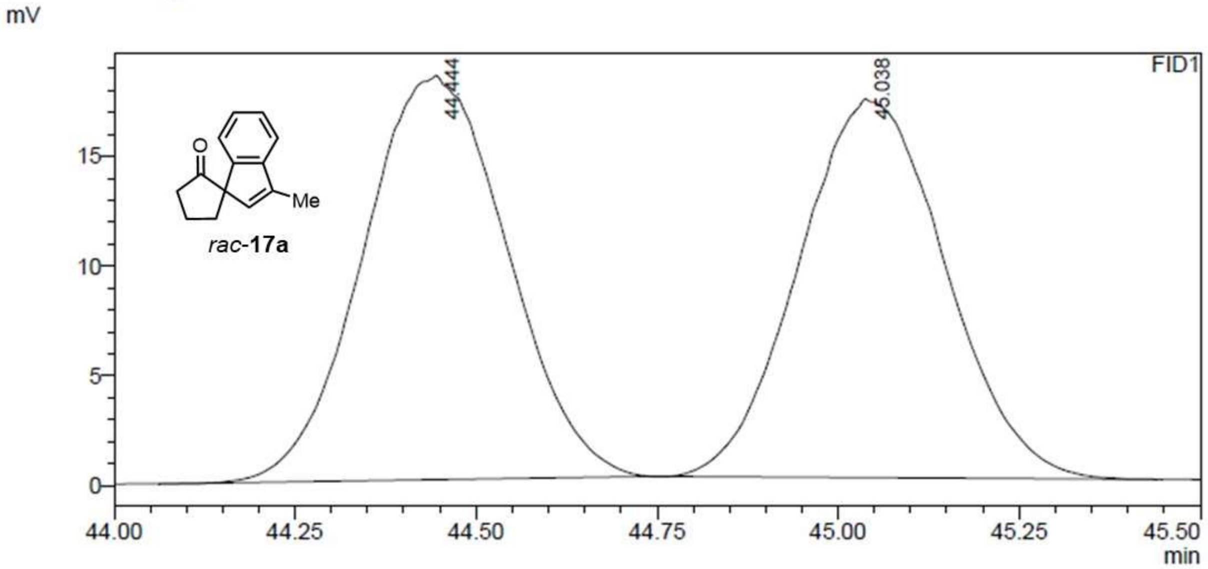

<Peak Table>

FID1

Peak#	Ret. Time	Area	Height	Conc.
1	44.444	255187	18389	51.215
2	45.038	243082	17222	48.785
Total		498269	35610	

<Chromatogram> Compound **17a**

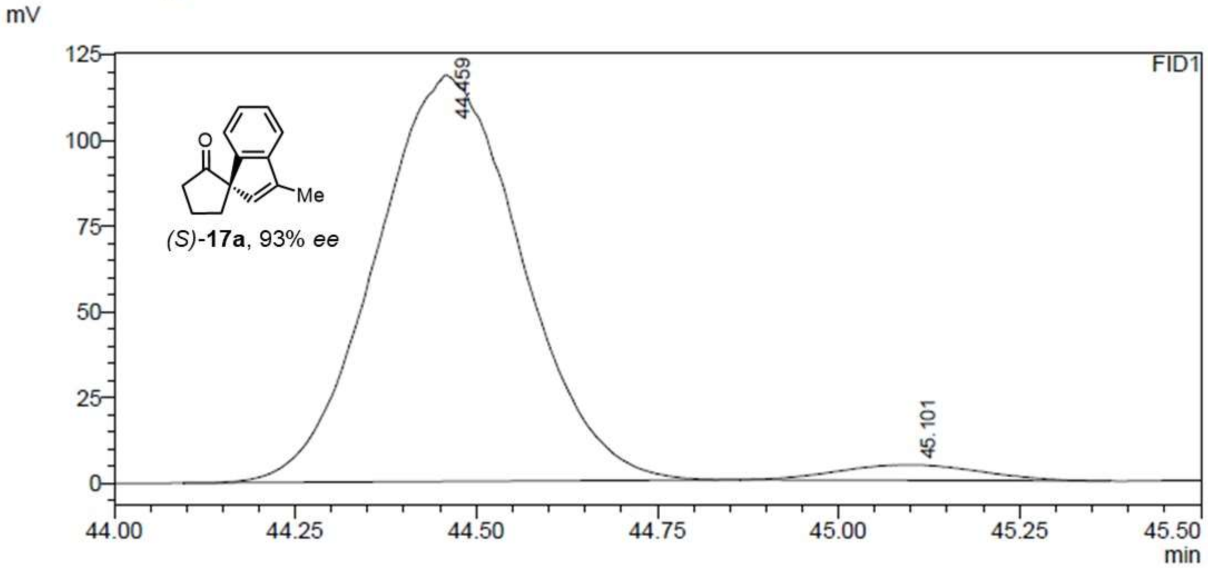

<Peak Table>

FID1

Peak#	Ret. Time	Area	Height	Conc.
1	44.459	1658773	118470	96.407
2	45.101	61823	4562	3.593
Total		1720596	123032	

<Chromatogram>

Compound **17b**

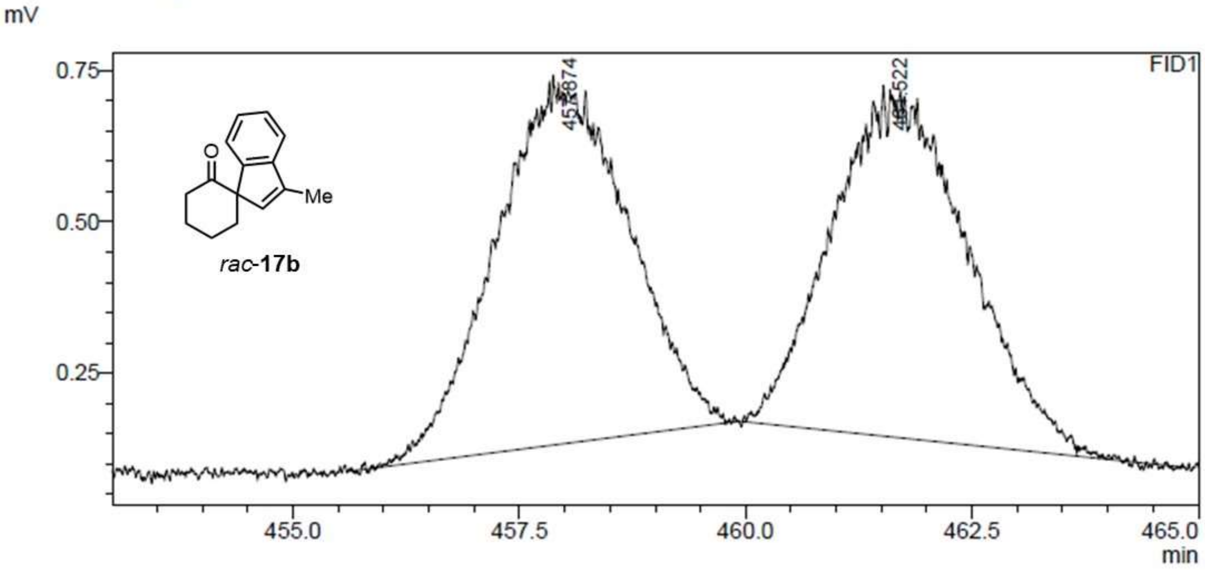

<Peak Table>

FID1

Peak#	Ret. Time	Area	Height	Conc.
1	457.874	61269	609	50.553
2	461.522	59929	578	49.447
Total		121199	1188	

<Chromatogram>

Compound **17b**

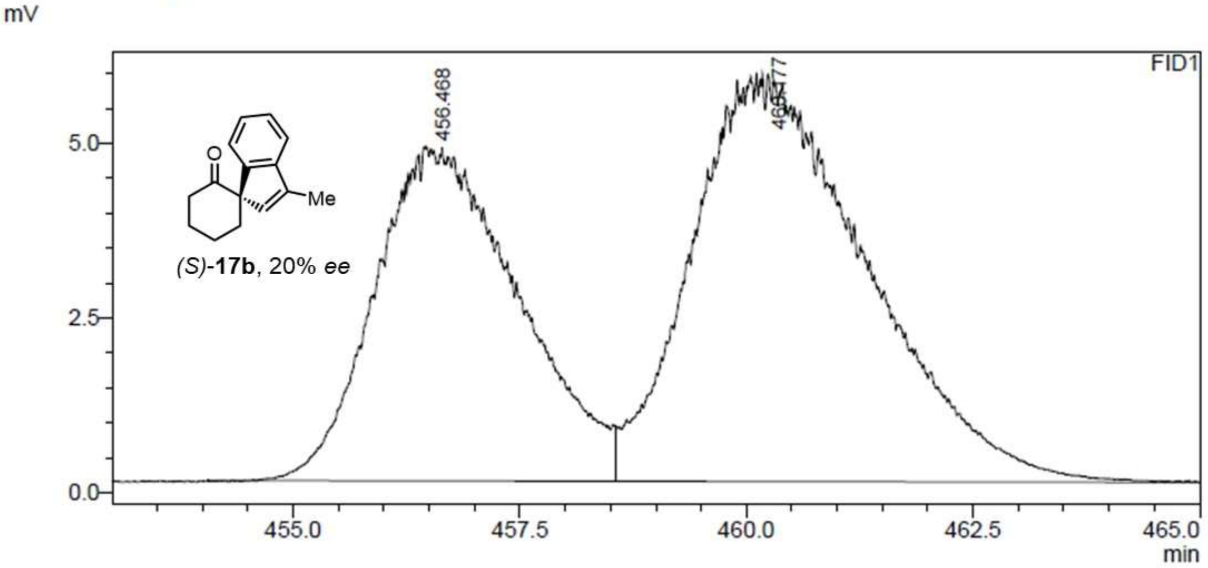

<Peak Table>

FID1

Peak#	Ret. Time	Area	Height	Conc.
1	456.468	525811	4790	40.250
2	460.177	780552	5829	59.750
Total		1306363	10618	

<Chromatogram> Compound **17c**

mV

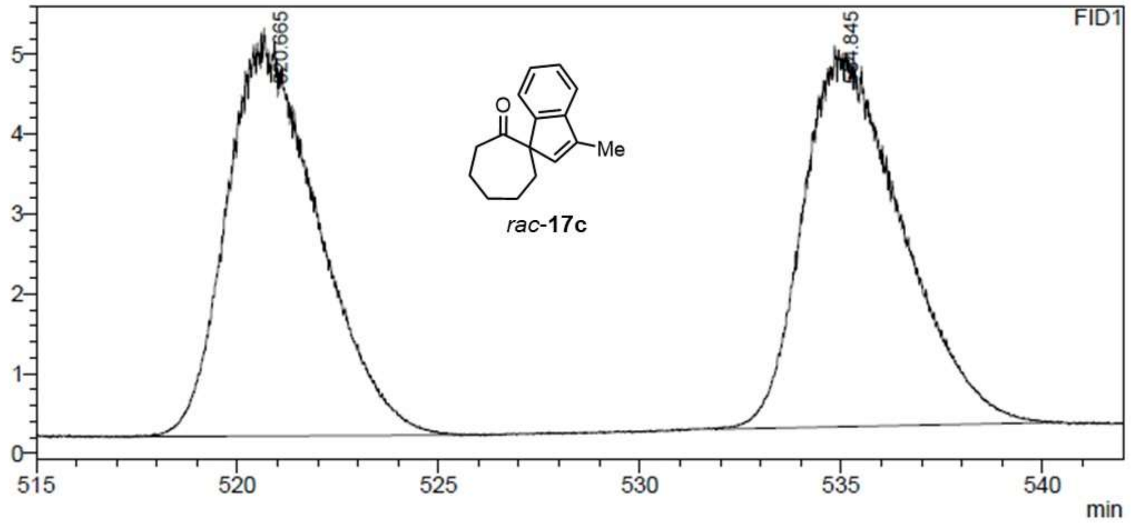

<Peak Table>

FID1

Peak#	Ret. Time	Area	Height	Conc.
1	520.665	787666	5100	49.575
2	534.845	801158	4771	50.425
Total		1588824	9870	

<Chromatogram> Compound **17c**

mV

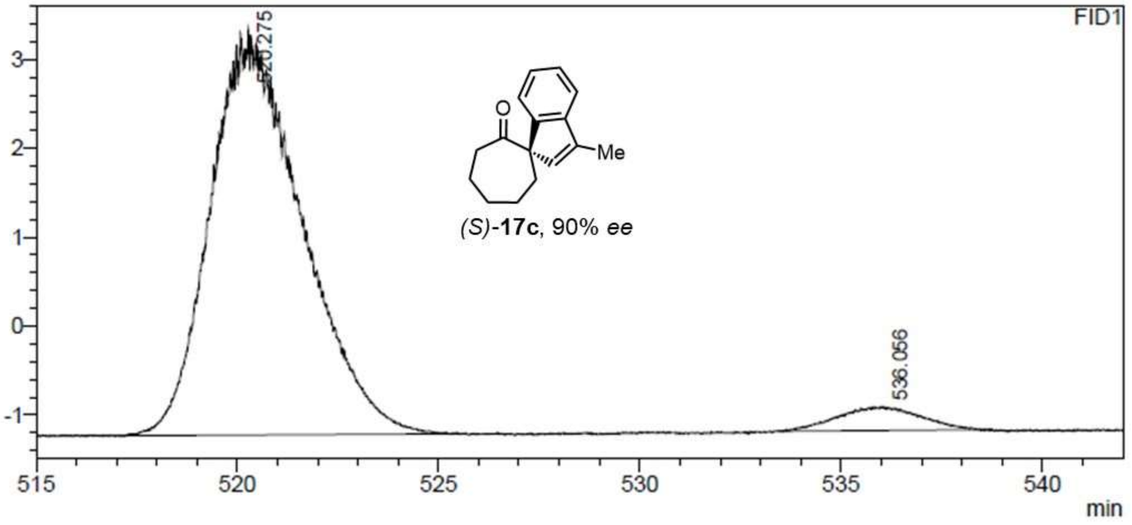

<Peak Table>

FID1

Peak#	Ret. Time	Area	Height	Conc.
1	520.275	712779	4574	94.940
2	536.056	37991	269	5.060
Total		750770	4843	

<Chromatogram> Compound **17d**

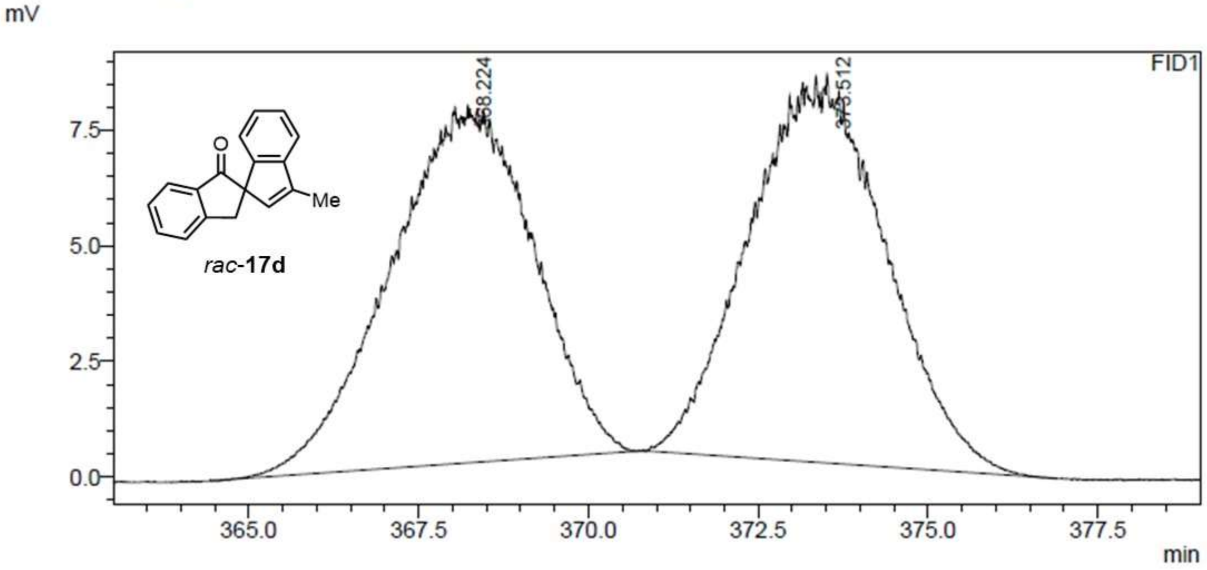

<Peak Table>

FID1

Peak#	Ret. Time	Area	Height	Conc.
1	368.224	1127719	7721	49.167
2	373.512	1165931	8404	50.833
Total		2293650	16125	

<Chromatogram> Compound **17d**

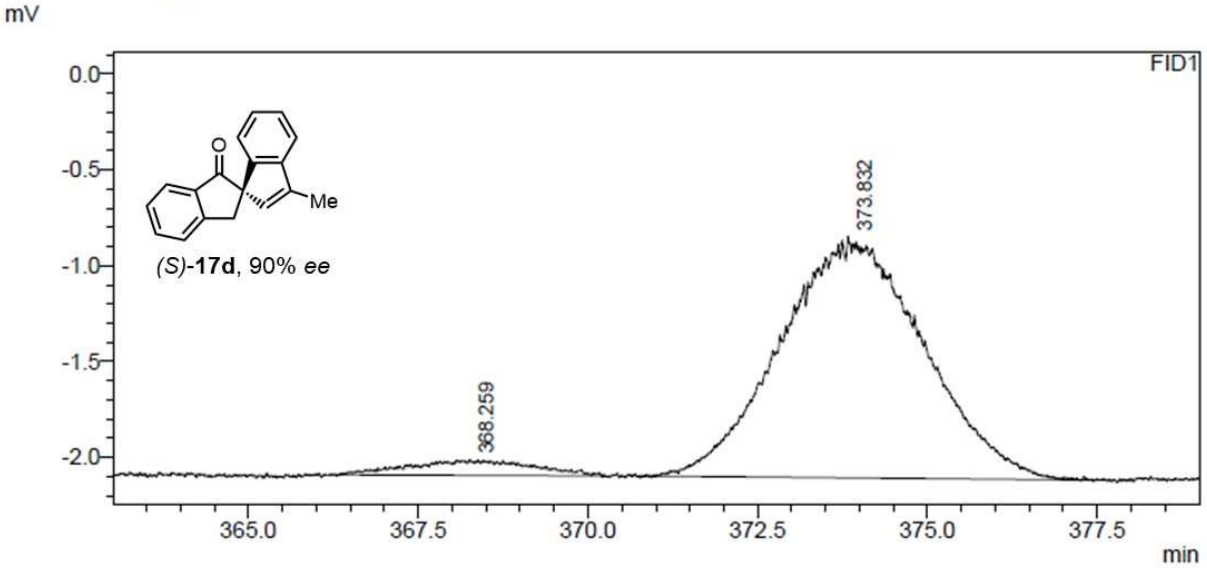

<Peak Table>

FID1

Peak#	Ret. Time	Area	Height	Conc.
1	368.259	10032	83	5.218
2	373.832	182237	1259	94.782
Total		192269	1343	

<Chromatogram>

Compound **17e**

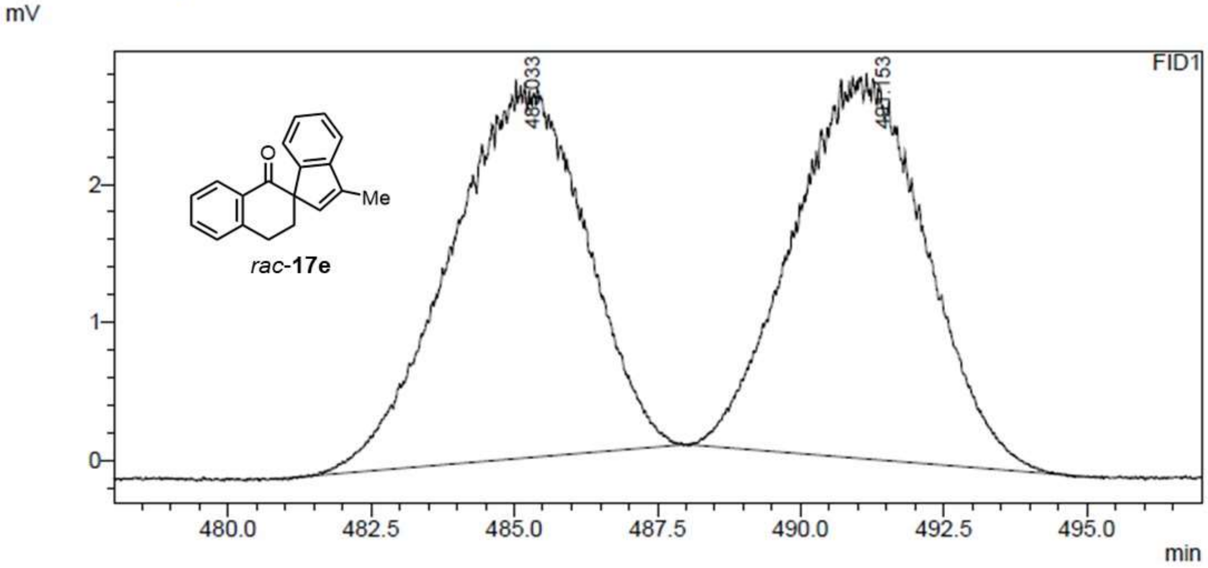

<Peak Table>

FID1

Peak#	Ret. Time	Area	Height	Conc.
1	485.033	443843	2749	49.831
2	491.153	446849	2791	50.169
Total		890692	5540	

<Chromatogram>

Compound **17e**

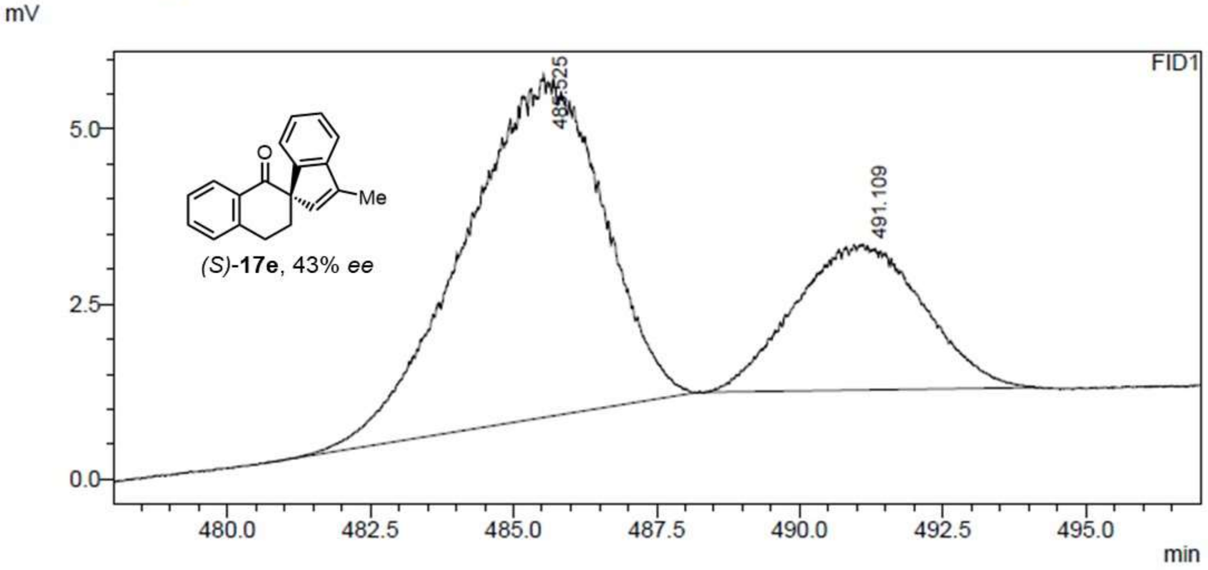

<Peak Table>

FID1

Peak#	Ret. Time	Area	Height	Conc.
1	485.525	817106	4900	71.514
2	491.109	325475	2085	28.486
Total		1142581	6985	

<Chromatogram>

Compound **17f**

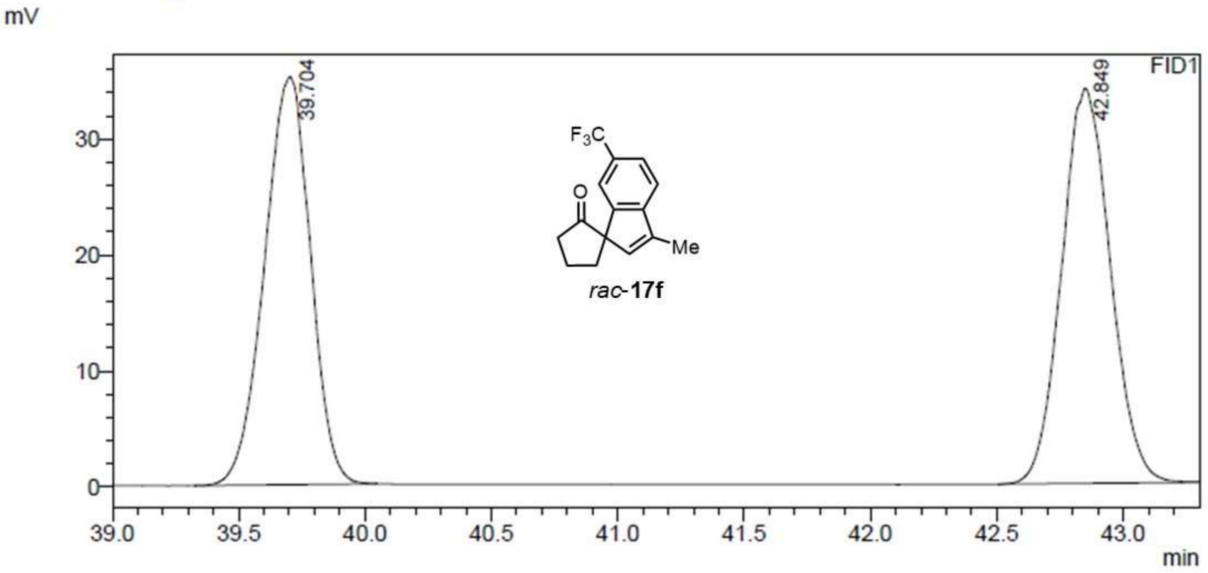

<Peak Table>

FID1

Peak#	Ret. Time	Area	Height	Conc.
1	39.704	445625	35118	49.306
2	42.849	458176	34037	50.694
Total		903802	69156	

<Chromatogram>

Compound **17f**

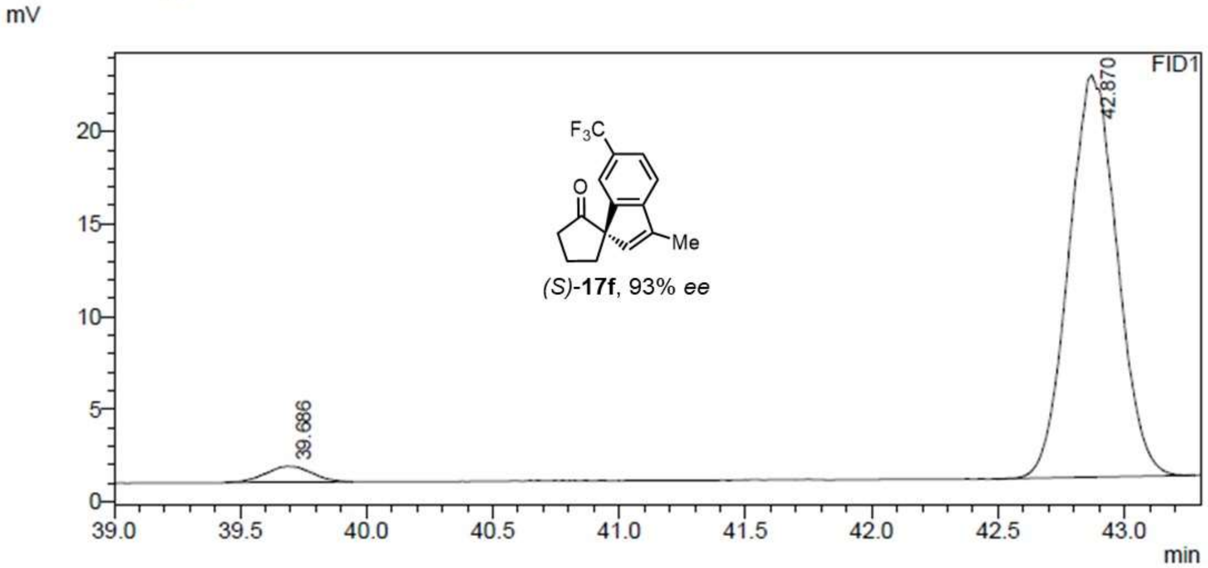

<Peak Table>

FID1

Peak#	Ret. Time	Area	Height	Conc.
1	39.686	10672	846	3.555
2	42.870	289533	21712	96.445
Total		300206	22558	

<Chromatogram>

Compound **17g**

mV

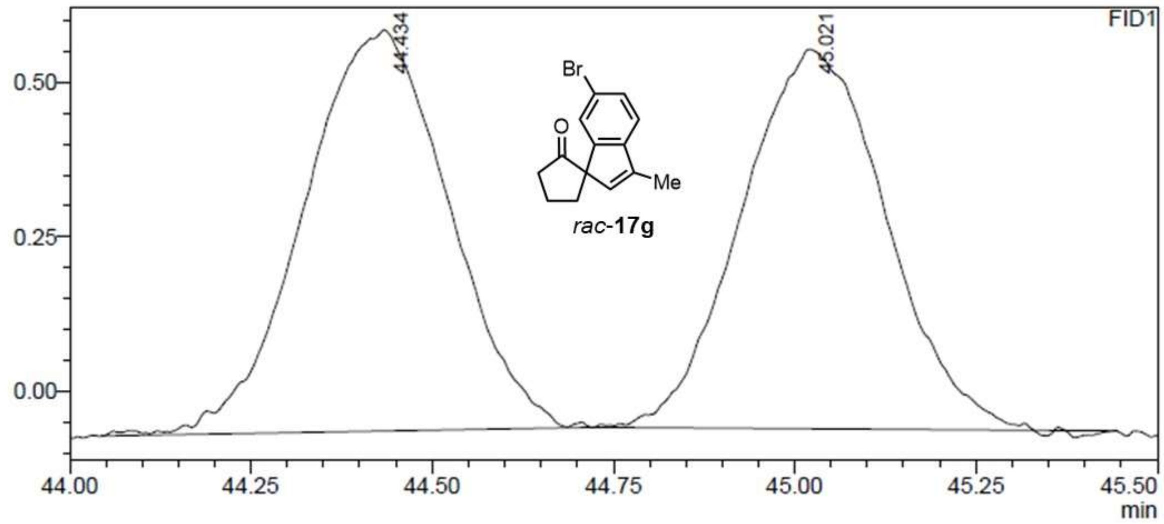

<Peak Table>

FID1

Peak#	Ret. Time	Area	Height	Conc.
1	44.434	9018	649	51.488
2	45.021	8497	614	48.512
Total		17515	1262	

<Chromatogram>

Compound **17g**

mV

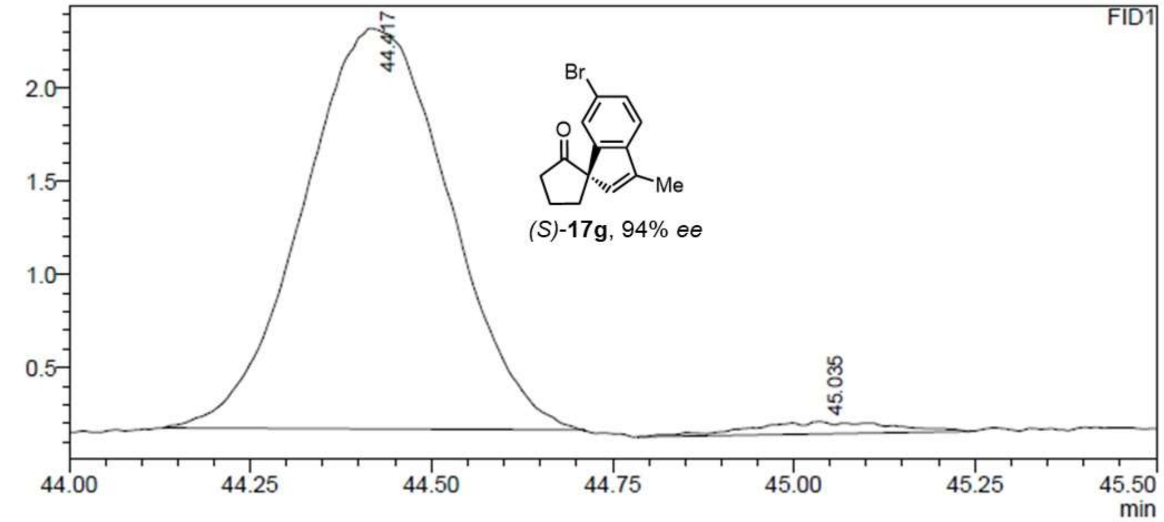

<Peak Table>

FID1

Peak#	Ret. Time	Area	Height	Conc.
1	44.417	30096	2148	97.182
2	45.035	873	66	2.818
Total		30969	2214	
